# Preparation of T_8_ and double-decker silsesquioxane-based Janus-type molecules: molecular modeling and DFT insights

**DOI:** 10.1038/s41598-024-69481-6

**Published:** 2024-08-09

**Authors:** Julia Duszczak-Kaczmarek, Katarzyna Mituła-Chmielowiec, Monika Rzonsowska, Wojciech Jankowski, Marcin Hoffmann, Jędrzej Walkowiak, Beata Dudziec

**Affiliations:** 1 Faculty of Chemistry, Adam Mickiewicz University in Poznan, Uniwersytetu Poznanskiego 8, 61-614 Poznan, Poland; 2 Center for Advanced Technologies, Adam Mickiewicz University in Poznan, Uniwersytetu Poznanskiego 10, 61-614 Poznan, Poland

**Keywords:** Silsesquioxane, DDSQ, Janus compounds, Catalysis, Hydrosilylation, Molecular modeling, DFT, Organic-inorganic nanostructures, Chemistry, Organometallic chemistry

## Abstract

We present a methodology for the synthesis of inorganic-organic Janus-type molecules based on mono-T_8_ and difunctionalized double-decker silsesquioxanes (DDSQs) *via* hydrosilylation reactions, achieving exceptionally high yields and selectivities. The synthesized compounds were extensively characterized using various spectroscopic techniques, and their sizes and spatial arrangements were predicted through molecular modelling and density functional theory (DFT) calculations. Quantum chemical calculations were employed to examine the interactions among four molecules of the synthesized compounds. These computational results allowed us to determine the propensity for molecular aggregation, identify the functional groups involved in these interactions, and understand the changes in interatomic distances during aggregation. Understanding the aggregation behaviour of silsesquioxane molecules is crucial for tailoring their properties for specific applications, such as nanocomposites, surface coatings, drug delivery systems, and catalysts. Through a combination of experimental and computational approaches, this study provides valuable insights into the design and optimization of silsesquioxane-based Janus-type molecules for enhanced performance across various fields.

## Introduction

Polyhedral Oligomeric Silsesquioxanes (SQs, POSS^®^)^1^ belong to a large and diverse class of specific organosilicon frameworks with inorganic Si-O-Si core and organic coronae, constituting hybrid molecules of the general formula (RSiO_3/2_)_n_ up to n=18. They exhibit diverse architectures, from random *via* ladder to partially or closed caged ones^2,3^. Still, from the application point of view, the most important are cube-like T_8_ derivatives but also double-decker silsesquioxanes (DDSQ)^4–6^. SQs for their unique physicochemical properties have found application in many branches of chemistry, e.g. in materials (building blocks or polymer modifiers)^7,8^, optoelectronics (e.g. sensors, OLED devices)^9^, heterogeneous catalysis^10^, or in medicine (e.g. drug delivery systems)^11,12^.

Janus particles have been known in the literature for nearly twenty years. Nonetheless, they are still arousing scientific interest. These systems are characterized by exclusive properties due to their asymmetrical structure that constitutes two kinds of surfaces with diverse physico-chemical features. In 1989, Casagrande and Veyssie were the firsts to describe spherical glass particles with one of the hemisphere hydrophobic and another hydrophilic. In their report, the amphiphilic beads were synthesized by protecting one of the hemispheres with cellulose varnish and treating another part of the molecule with a silane reagent (octadecyl trichlorosilane)^13^. The unique surface of Janus molecule allows two distinct types of chemistry and physics to occur on the same particle^14^. Until now, in the scientific literature, Janus molecules have been based mainly on: polystyrene and poly (methyl methacrylate), polyacrylic acid, gold and iron oxides, fibers, allyl alcohol, protein cages, and pigments^15–17^. Thanks to the fact that they possess two distinct “faces” in their structure, it makes them a particular class of materials among microparticles^16^. Janus molecules can be divided into a few groups, according to their architecture and dimensionality e.g. spherical, two types of disc-like particles and two types of cylinders^16^. Due to such different physical and chemical properties in one structure, the synthesis of Janus particles has remained challenging for a long time. It requires the ability to create (selectively and with high yield) each hemisphere particle with individual features in a unique way. Currently, few major methods have been applied in the synthesis of these systems, e.g. masking, self-assembly, phase separation^14^. One of the most popular and important strategies for the Janus particles synthesis is to temporarily immobilize one face of a particle for symmetry-breaking. On the other hand, masking involves the protection of one side of a nanoparticle followed by the modification of the unprotected side and the removal of the protection^14^.

Since the last decade, Janus-type molecules have found numerous applications, among others as nanocorals^18^, water-repellent fibers^15^, catalyst, stabilizers in emulsions^19^, batteries^20^, nanocomposites, membranes, novel compatibilizer for PS/PMMA polymer blends^21,22^, and as an amphiphilic colloidal surfactants^13,14^. The use of spherical Janus molecules has allowed increasing work efficiency of optical probes for biological interactions or rheological measurements in confined space (compared to ordinary molecules). With this strategy, it is possible to create devices ranging from precise nanoviscosimeters to nanothermometers^23–25^.

The extension of the synthetic approach towards Janus particles has been also reflected in the use of specific organosilicon frameworks, i.e. functionalized silsesquioxanes (SQs). In recent years, Professor Unno research group has reported the formation of Janus-type inorganic-organic nanoparticles based on cubic T_8_ silsesquioxane containing four phenyl groups and four *iso*-butyl groups in the cage^26^. The other results refer to the synthesis of the same cubic structure with a different kind of inert groups affecting the physical properties of these molecules^27–29^. The synthesis of Janus-type nanoparticles based on silsesquioxanes involves the formation of organic-inorganic hybrids which properties become dual. The inorganic core of silsesquioxane provides, among others very good thermal and mechanical stability, dielectric properties, oxidation resistance as well as non-flammability. A properly selected organic inert group (alkyl and aryl type) can positively influence, e.g. solubility in organic solvents, amphiphilic or optoelectronic properties. By combining two silsesquioxane (SQ) compounds with different organic substituents into one molecule, one can enhance the functional versatility of the resulting system. This may enable modulation of its solubility in organic and/or aqueous solvents, potential self-assembly capabilities, and diversification of chemical reactivity.

Their further application is also possible, e.g. as complex metals and anions, used as a selective sensor for the recognition of ions and biomolecules^30^. Janus-type particles containing silsesquioxanes may form amphiphilic molecules to obtain improved colloidal surfactants or solvent separation membranes^31–33^. The use of silsesquioxanes for the synthesis of Janus molecules improves the compatibility properties in polymer mixing, reduces the size of domains and structural defects as well as enhances the mechanical properties of the entire system (e.g. in Young’s modulus and strain at the break or hardness)^31,34^. The scientific literature concerning synthetic methodology using silsesquioxanes for the formation of Janus-type nanoparticles is still limited and mainly based on mono- and octafunctionalized silsesquioxanes^20,21, 30, 31, 34–39^. Moreover, there are no literature reports on the use of DDSQs that are still quite novel class of organosilicon Si-O-Si frameworks.

**Figure 1:**
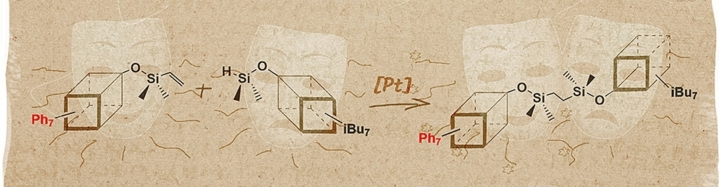
Selected example of elaborated Silsesquioxane-Based Janus-type structures.

The proposed research fulfills the idea of basic research as it concerns the original experimental work undertaken to acquire new information on the synthesis and finally the properties of Janus-type molecules containing silsesquioxanes with diverse organic coronae. The synthesis of Janus-type molecules utilizing silsesquioxanes and DDSQ-type system and selection of catalytic protocols to yield those systems, represents a significant advancement in this field of chemistry. By leveraging the structural versatility and reactivity of double-decker silsesquioxanes alongside the precise control afforded by hydrosilylation chemistry, this research paves the way for tailored Janus architectures with enhanced functionality and tunable properties. The scope of this study includes an application of hydrosilylation in presence of TM complexes, e.g. platinum, for the preparation of products (Fig. 1). Hydrosilylation has been a fundamental synthetic path leading to saturated or unsaturated organosilicon compounds^40^. It is a convenient synthetic tool of fundamental importance in the industry towards the formation of molecular and macromolecular organosilicon compounds of desirable physical and chemical properties^41^. In addition to experimental synthesis, this research integrates computational molecular modeling and Density Functional Theory (DFT) calculations to provide crucial insights into the structural properties and electronic behaviors of the designed Janus molecules. By combining experimental findings with computational modeling, a comprehensive understanding of the structure-function relationships and potential applications of the synthesized Janus molecules can be achieved. This synergistic approach not only validates experimental observations but also enables predictive modeling and facilitates further refinement of molecular design strategies, offering a holistic framework for advancing the field of Janus molecule synthesis and applications.

## Experimental section

### Materials

The chemicals were purchased from the following sources: Sigma-Aldrich for toluene, chloroform, dichloromethane-*d*, THF, chloroform-*d***,** Karstedt’s catalyst ([Pt_2_(dvs)_3_]) (2% xylene solution), Chloro(1,5-cyclooctadiene)iridium(I) dimer ([Ir(cod)Cl]_2_) and silica gel 60. AmBeed for 2-Dicyclohexylphosphino-2′,4′,6′-triisopropylbiphenyl (XPhos). Thermo Scientific Acros for PtO_2_. Pt/SDB was prepared according to the previously reported method^42^. The following silsesquioxanes: DDSQ-2SiOSiVi and DDSQ-2SiVi and 1-dimethylsiloxy-3,5,7,9,11,13,15-hepta(ethyl)pentacyclo[9.5.1.1^3,9^.1^5,15^.1^7,13^]octasiloxane (**Et**_**7**_**T**_**8**_**H**), 1-dimethylvinylsiloxy-3,5,7,9,11,13,15-hepta(phenyl)pentacyclo[9.5.1.1^3,9^.1^5,15^.1^7,13^]octasiloxane (**Ph**_**7**_**T**_**8**_**Vi**), 1-dimethylsiloxy-3,5,7,9,11,13,15-hepta(isobutyl)pentacyclo[9.5.1.1^3,9^.1^5,15^.1^7,13^]octasiloxane (**iBu**_**7**_**T**_**8**_**H**), 1-dimethylvinylsiloxy-3,5,7,9,11,13,15-hepta(isobutyl)pentacyclo[9.5.1.1^3,9^.1^5,15^.1^7,13^]octasiloxane (**iBu**_**7**_**T**_**8**_**Vi**), 1-dimethylsiloxy-3,5,7,9,11,13,15-hepta(isooctyl)pentacyclo[9.5.1.1^3,9^.1^5,15^.1^7,13^]octasiloxane (**iOc**_**7**_**T**_**8**_**H**), 1-dimethylsiloxy-3,5,7,9,11,13,15-hepta(phenyl)pentacyclo[9.5.1.1^3,9^.1^5,15^.1^7,13^]octasiloxane (**Ph**_**7**_**T**_**8**_**H**) were prepared according to the literatures procedure^43–46^. All solvents were dried over CaH_2_ prior to use and stored under argon over 4Å molecular sieves. The liquid substrates were dried and degassed by bulb-to-bulb distillation. All syntheses were conducted under argon atmosphere using standard Schlenk-line and vacuum techniques.

### Measurements

#### Nuclear magnetic resonance (NMR)

^1^H, ^13^C, and ^29^Si Nuclear Magnetic Resonance (NMR) were performed on Brucker Ultra Shield 400 and 300 spectrometers using CD_2_Cl_2_ or CDCl_3_ as a solvent. Chemical shifts are reported in ppm with reference to the residual solvent (CD_2_Cl_2_ or CDCl_3_) peaks for ^1^H and ^13^C and to TMS for ^29^Si NMR.

#### FT-IR spectroscopy

Fourier Transform-Infrared (FT-IR) spectra were recorded on a Nicolet iS5 (Thermo Scientific) spectrophotometer equipped with a diamond ATR unit. In all cases, 16 scans at a resolution of 2 cm^−1^ were collected, to record the spectra in a range of 4000-450 cm^−1^.

#### Elemental analyses

Elemental analyses were performed using a Vario EL III instrument.

#### Density functional theory (DFT) calculations

For both investigated compounds **Ph**_**7**_**T**_**8**_-**T**_**8**_**Et**_**7**_ and **Ph**_**7**_**T**_**8**_-**T**_**8**_***i*Bu**_**7**_ four molecules were used to create system for simulations. Our goal was to find possible interactions which will suggest aggregation of those structures. We also wanted to check how interatomic distances changes during simulation and if those distances affect the energy of the obtained clusters. The CHARMM36 force field was selected to perform Molecular Dynamics simulations as it is often used for this type of simulations^47^. Each molecule was parametrized with the use of PARATOOL^48^ program which is a plug-in for the molecular viewer VMD^49^. The molecular geometry of each compound was optimized at B3LYP/6-31+G(d)^50,51^ level of theory. Sets of point charges and a Cartesian Hessian matrices were obtained from natural population (NPA) and frequency analyses^52^ for each molecule studied. The calculations were performed with the Gaussian 09 programme^53^. Molecular Dynamics simulations were performed in chloroform box obtained with use of VMD Solvate Plugin^54^. Electrostatic and van der Waals interactions were treated with a cut-off of 12 Å as it was suggested for non-bonded interactions in simulations with CHARMM parameters^55^. The first step of each simulation was minimization. Energy minimization was followed by simulated annealing for 1.4 ns with temperature rising from 250 to 600 K^56,57^ and then decreasing back to 250 K. The last step of simulation was the production run during which temperature was kept at 298 K by applying to all heavy atoms the Langevin forces with damping coefficient of 1ps^−1^^58^. Production run lasted 60 ns. All simulations were performed in NAMD 2.9^59^. To assess the stability of the investigated systems we calculated RMSD (Root- mean-square deviation)^60^. Calculated RMSD were used to group conformers into eight clusters, with use of VMD Clustering plug-in, which is using quality threshold algorithm^61,62^. After completing simulations and grouping conformers to eight clusters, for each cluster average structures were obtained. Electronic energies of those structures were then computed with B3LYP^63^ method and 6-31++G(d,p)^64^ basis set which were also used for computational studies of similar compounds^65–67^. To take into account the effect of chloroform solvent we used the Polarizable Continuum Model (PCM)^68^.

### Synthetic procedures

#### General synthetic procedure for Janus-type organic-inorganic molecules based on monofunctionalized silsesquioxanes obtained via hydrosilylation reaction

The procedure for the synthesis of **Ph**_**7**_**T**_**8**_-**T**_**8**_***i*Bu**_**7**_ is described as an example. Into a two-neck round-bottom flask equipped with a magnetic stirrer **Ph**_**7**_**T**_**8**_**Vi** (0.047 g, 0.044 mmol), an anhydrous toluene (6 mL) and 1.0 equiv. of ***i*Bu**_**7**_**T**_**8**_**H** (0.04 g, 0.044 mmol) were added. The reaction mixture was heated up to 95 ℃ and 1×10^−3^ equiv. of [Pt_2_(dvs)_3_] (4.4×10^−8 ^mol) was added. Reactions were carried out until >99% conversion of ***i*Bu**_**7**_**T**_**8**_**H**, that was precisely controlled by FT-IR spectroscopy. After cooling it to room temperature, the excess of solvent was evaporated under vacuum and crude product was transferred onto chromatographic column (silica gel 60) using chloroform as an eluent. Evaporation of eluent gave an analytically pure sample of **Ph**_**7**_**T**_**8**_-**T**_**8**_***i*Bu**_**7**_ with 94% yield.

#### General synthetic procedure for Janus-type organic-inorganic molecules based on monofunctionalized silsesquioxane and difunctional DDSQ compounds obtained via hydrosilylation reaction

The procedure for the synthesis of DDSQ-2Si-(T8iBu7)2 is described as an example. Into a two-neck round-bottom flask equipped with a magnetic stirrer, **DDSQ-2SiVi** (0.034 g, 0.029 mmol), an anhydrous toluene (6 mL) and 2.0 equiv. of *i*Bu_7_POSS-OSiMe_2_H (***i*Bu**_**7**_**T**_**8**_**H**) (0.05 g, 0.058 mmol) were added respectively. The reaction mixture was heated up to 95 ℃ and 2×10^−3^ equiv. of [Pt_2_(dvs)_3_] (5.8 ×10^−8 ^mol) was added. Reactions were carried out until >99% conversion of ***i*Bu**_**7**_**T**_**8**_**H**, that was precisely controlled by FT-IR spectroscopy, analysing the disappearance of signal at 2139 cm^−1^ (from Si-H bond). After cooling the reaction mixture to room temperature, the excess of solvent was evaporated under vacuum and crude product was transferred onto chromatographic column (silica gel 60) using chloroform as an eluent. Evaporation of eluent gave an analytically pure sample of **DDSQ-2Si-(T**_**8**_***i*Bu**_**7**_**)**_**2**_ with 93% yield.

#### Additional tests for hydrosilylation reaction of DDSQ-2SiVi with iOc_7_T8H in the presence of different catalysts

##### [IrCl(cod)]_2_^69^

**DDSQ-2SiVi** (30 mg, 0.025 mmol), [IrCl(cod)]_2_ (0.7 mg, 2×10^−6 ^mol) and anhydrous toluene (2 mL) were introduced into Schlenk reactor purged with argon and equipped with a magnetic stirrer. Afterwards **iOc**_**7**_**T**_**8**_**H** (64 mg, 0.05 mmol) in 3 mL of anhydrous toluene was added dropwise. The reaction mixture was heated up to 95 ℃ and carried out for 24 h. After cooling the reaction mixture to room temperature, the excess of solvent was evaporated under vacuum and crude product was transferred onto chromatographic column (silica gel 60) using chloroform as an eluent. Evaporation of eluent gave an analytically pure sample.

##### PtO_2_/XPhos^70^

PtO_2_ (0.02 mg, 6.5×10^−7 ^mol) and XPhos (0.6 mg, 1.3×10^−6 ^mol) and anhydrous THF (1 mL) were introduced into Schlenk reactor purged with argon and equipped with a magnetic stirrer. The reaction mixture was heated up to 60 ℃. Afterwards **iOc**_**7**_**T**_**8**_**H** (64 mg, 0.05 mmol) in 2 mL of THF and **DDSQ-2SiVi** (30 mg, 0.025 mmol) in 2 mL of THF were added dropwise. The reaction was carried out for 24 h. After cooling the reaction mixture to room temperature, the excess of solvent was evaporated under vacuum and crude product was transferred onto chromatographic column (silica gel 60) using chloroform as an eluent. Evaporation of eluent gave an analytically pure sample.

##### Pt/SDB^42^

**DDSQ-2SiVi** (30 mg, 0.025 mmol) and anhydrous toluene (2 mL) were introduced into Schlenk reactor purged with argon and equipped with a magnetic stirrer. Afterwards **iOc**_**7**_**T**_**8**_**H** (64 mg, 0.05 mmol) in 3 mL of anhydrous toluene was added dropwise. The reaction mixture was heated up to 90 ℃ and Pt/SDB was added (0.3 mg, 1.7×10^−6 ^mol). Reaction was carried out for 24 h. After cooling the reaction mixture to room temperature, the excess of solvent was evaporated under vacuum and crude product was transferred onto chromatographic column (silica gel 60) using chloroform as an eluent. Evaporation of eluent gave an analytically pure sample.

## Result and discussion

The reports on Janus-type compounds based on silsesquioxanes has been still limited. As a result, there was a scientific necessity to explore this area of chemistry. It should be underlined that the combinations of SQs structures with inert and functional groups anchored onto Si-O-Si core constitute a vast library of Janus-type organic-inorganic compounds of complex structural motifs that may possess various and interesting physical and chemical properties. This concept is an expansion of the up to date research in the area of SQ chemistry.

**Figure 2:**
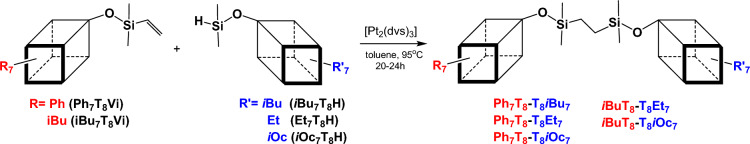
The synthetic procedure to obtain SQs-based Janus *via* hydrosilylation of two monofunctionalized T_8_-type SQs.

The first part of presented research was focused on hydrosilylation reaction of two monofunctionalized T_8_-type SQs possessing different inert groups anchored onto their cores (Ph, *i*Bu, Et, *i*Oc). Inert groups have been selected in great detail due to the differences in their physical and chemical properties (thermal stability, solubility, state of matter, modulus values, creep-recovery, reactivity, melting and crystallization temperatures, tensile strength and elongation) but also based on their availability^44,71, 72^. As pointed by Naka, physical properties (solubility, the state of matter, and their thermal behavior) are determined by, e.g. the type of chemically inert organic substituents present in the structure of SQ. It was observed that the thermal stability of the compounds increased with inert groups in a row: iBu < iOc < Ph^44^. Silsesquioxanes compounds with isobutyl groups yielded greater modulus values than their phenyl counterparts. Similar trends were observed with tensile strength and elongation at yield. Compounds with isobutyl groups also experienced less creep than their phenyl counterparts^71^. The main plot of the reaction along with the structures of exploited SQs structures is presented below (Fig. 2).

**Figure 3:**
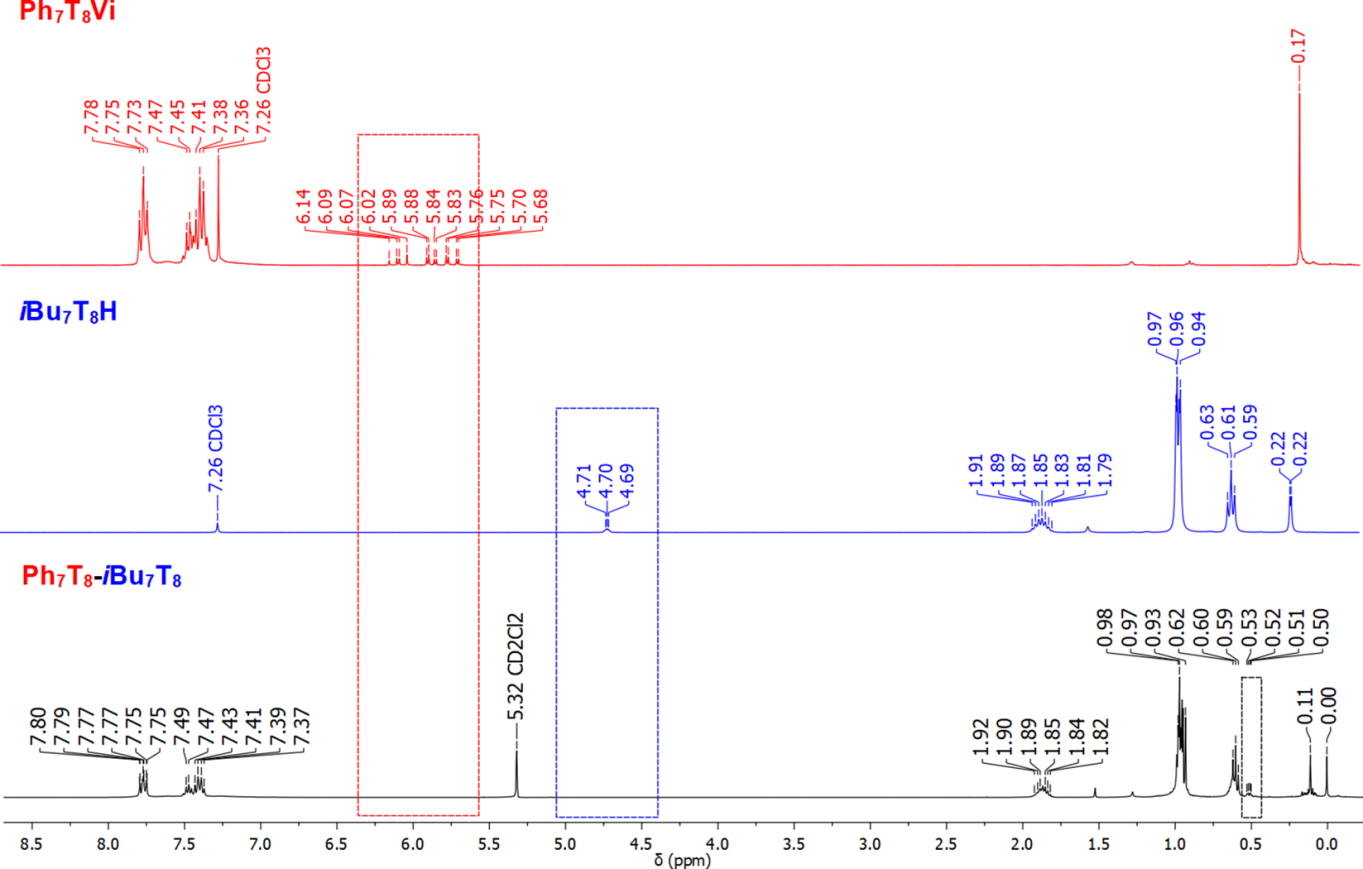
Stacked ^1^H NMR spectra of substrates **Ph**_**7**_**T**_**8**_**Vi**, ***i*Bu**_**7**_**T**_**8**_**H** and resulting product **Ph**_**7**_**T**_**8**_-**T**_**8**_***i*Bu**_**7**_.

As an example, we present a Janus-type organic-inorganic molecule based on two monofunctionalized silsesquioxanes (containing two different types of groups: phenyl and *iso-*Butyl). Hydrosilylation reaction was performed using Karstedt’s catalyst, 10^−3^ mol per 1 mol of Si-H group were established basing on literature for molecular compounds. The reaction was monitored *via* FT-IR to detect complete conversion of ***i*Bu**_**7**_**T**_**8**_**H** (Si-H bond disappearance at ῡ = 2138 and 900 cm^−1^). What is more, the process was strictly controlled by the ^1^H NMR with Si-H (4.71–4.69 ppm) as well as Si-HC=CH_2_ (6.14–5.68 ppm) chemical shifts disappearance from ***i*Bu**_**7**_**T**_**8**_**H** and **Ph**_**7**_**T**_**8**_**Vi** respectively (Fig. 3). Also, the exclusive formation of the *β*-hydrosilylation product was confirmed, i.e. CH_2_ moiety presence was observed (0.53–0.50 ppm, Fig. 3) along with the absence of CH and CH_3_ groups, characteristics for *α*-hydrosilylation. The reaction product **Ph**_**7**_**T**_**8**_**-T**_**8**_***i*Bu**_**7**_ was isolated with 94% yield.

**Figure 4:**
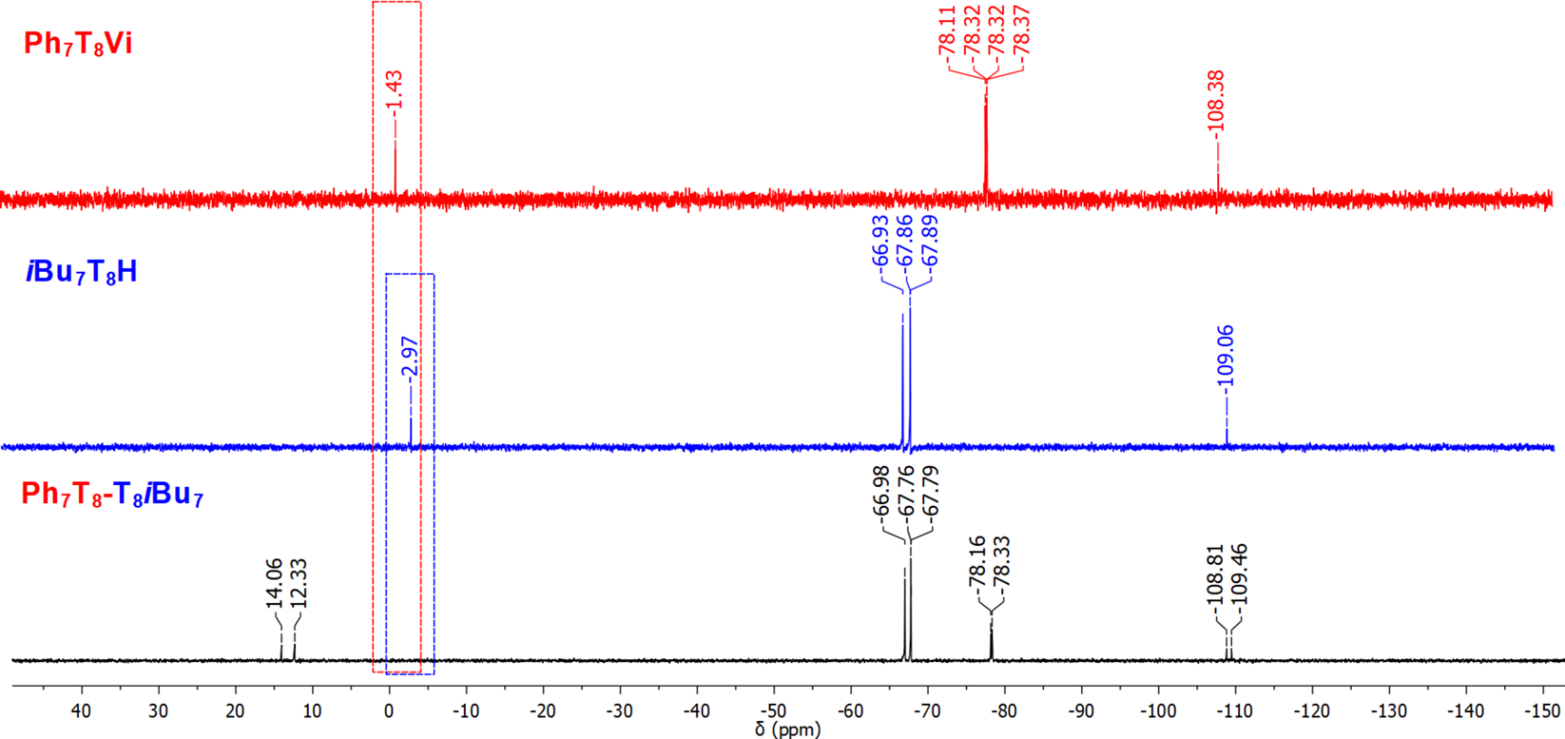
Stacked ^29^Si NMR spectra of substrates **Ph**_**7**_**T**_**8**_**Vi**, ***i*Bu**_**7**_**T**_**8**_**H** and resulting product **Ph**_**7**_**T**_**8**_-**T**_**8**_***i*Bu**_**7**_.

Formation of the desired product was also confirmed by ^29^Si NMR analysis. Nine signals, at 14.06, 12.33, −66.98, −67.76, −67.79, −78.16, −78.33, −108.81 and −109.46 ppm in the case of **Ph**_**7**_**T**_**8**_-**T**_**8**_***i*Bu**_**7**_ can be discerned (Fig. 4). The first two signals might be assigned to the Si^M^ atoms shifted significantly in comparison to the respectively signals of Si^M^ atoms of the substrates (14.06 and 12.33 ppm *vs* -2.97 and −1.43 ppm). Signals −66.98, −67.76 and −67.79 ppm are assigned to the Si^T^ (from silsesquioxanes with *i*Bu moieties), −78.16 and −78.33 are assigned to the Si^T^ (from silsesquioxanes with Ph moieties), −108.81 is assigned to the Si^Q^ (from silsesquioxanes with Ph moieties) and the last one −109.46 is assigned to the Si^Q^ (from silsesquioxanes with *i*Bu moieties). The integrated areas of these signals are in excellent agreement with the expected values.

All obtained products based on monofunctionalized silsesquioxanes are air-stable solids. They are soluble in organic solvents like DCM, CDCl_3_, THF and toluene but not in, e.g. methanol, MeCN. They were isolated an characterized by means of spectroscopic methods (NMR, FT-IR spectroscopy) and elemental analysis.

**Figure 5:**
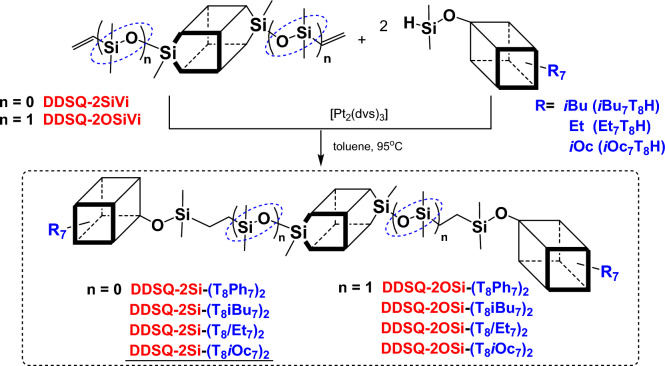
Janus-type organic-inorganic molecules based on monofunctionalized T_8_ SQs and difunctionalized DDSQs.

Structures of SQs may vary and even in the family of well-defined cages, so-called double-decker silsesquioxanes (DDSQ) are crucially different from T_8_-type systems. The structure of DDSQ includes two “decks” of cyclosiloxane rings stacked on top of one another with four phenyl groups at the silicon atoms of each ring (joined by oxygen bridges)^4^. Recent years have shown that the concept of synthesis of molecular and macromolecular organosilicon compounds based on DDSQ have led to two major trends in their development and our understanding of their interesting physicochemical properties. DDSQ might possess two or four reactive groups, therefore it is possible to obtain Janus-type molecular compounds differently silsesquioxanes. We focused our research on difunctional DDSQ and joined it with T_8_ SQs possessing different groups attached to their core (phenyl-Ph, *iso-*Butyl-*i*Bu, Ethyl-Et, *iso-*Octyl-*i*Oct) (Fig. 5). As a result a new type of Janus compounds based on silsesquioxanes differing in their Si-O-Si structures, resembling a dumbbell shape, were successfully obtained. To our knowledge this is the first example of DDSQs exploitation in formation of Janus-type compounds.

**Figure 6:**
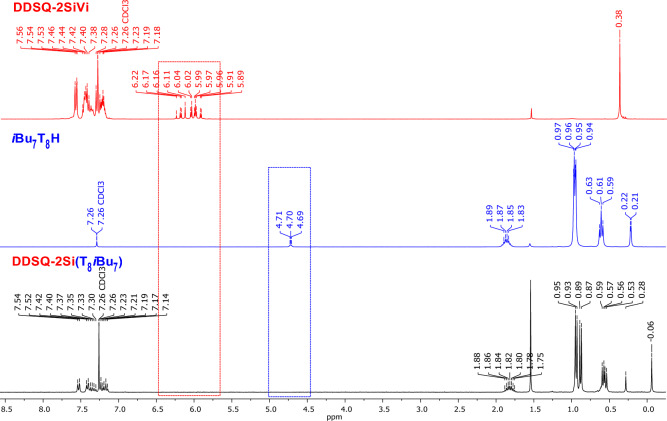
Stacked ^1^H NMR spectra of the substrates (**DDSQ-2SiVi**, ***i*Bu**_**7**_**T**_**8**_**H**) and final product **DDSQ-2Si**-**(T**_**8**_***i*Bu**_**7**_**)**_**2**_.

Reaction path for the hydrosilylation experiments of **DDSQ-2SiVi** with ***i*Bu**_**7**_**T**_**8**_**H** (to yield product **DDSQ-2Si**-**(T**_**8**_***i*Bu**_**7**_**)**_**2**_) is described as an example. 2×10^−3 ^mol of Karstedt’s catalyst per 1 mol of Si-H group was established based on literature reports. Estimated time for the reactions for all Janus-type organic-inorganic molecules was approximately 20–24 h. The reaction was monitored *via* FT-IR to detect complete conversion of ***i*Bu**_**7**_**T**_**8**_**H** (Si-H bond disappearance at ῡ = 2138 and 900 cm^-1^). According to the state of the art, addition sequence and stoichiometry of reagents are important for proper course of the reaction, i.e. 2 equiv. of ***i*Bu**_**7**_**T**_**8**_**H** per 1 equiv. of **DDSQ-2SiVi**. The reaction progress was also monitored by ^1^H NMR analyses. The disappearance of chemical shifts characteristic for Si-H (4.71–4.69 ppm) and Si-HC=CH_2_ groups (6.22–5.89 ppm) during hydrosilylation was observed (Fig. 6). What is more, the exclusive formation of the *β*-hydrosilylation product was confirmed, i.e. CH_2_ moiety presence (signal overlapped with signal from the CH_2_ of isobutyl group, i.e. 0.53–0.59 ppm) was observed along with the absence of CH and CH_3_ groups, characteristics for *α*-hydrosilylation. The reaction product **DDSQ-2Si**-**(T**_**8**_***i*Bu**_**7**_**)**_**2**_ was isolated with 93% yield.

**Figure 7:**
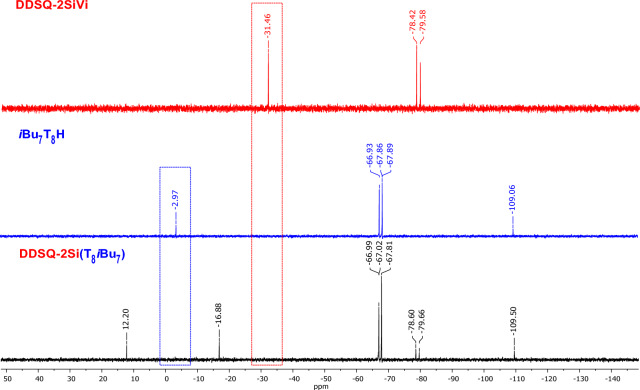
Stacked ^29^Si NMR spectra of the substrates (**DDSQ-2SiVi**, ***i*Bu**_**7**_**T**_**8**_**H**) and final product **DDSQ-2Si**-**(T**_**8**_***i*Bu**_**7**_**)**_**2**_.

Formation of the desired product was also confirmed by ^29^Si NMR analysis. Eight signals, at 12.20, −16.88, −66.99, −67.02, −67.81, −78.60, −79.60, and −109.50 ppm in the case of **DDSQ-2Si**-**(T**_**8**_***i*Bu**_**7**_**)**_**2**_ can be discerned (Fig. 7). The first signal might be assigned to the Si^M^ (from SQ with *i*Bu moieties), while the second is assigned to the Si^D^ (from DDSQ), signals −66.99, −67.02 and −67.81 ppm are assigned to the Si^T^ (from SQ with *i*Bu moieties), while −78.60 and −79.60 ppm are assigned to the Si^T^ (from DDSQ). The last one (−109.50 ppm) is assigned to the Si^Q^ (from SQ with *i*Bu moieties). The integrated areas of these signals are in excellent agreement with the expected values.

Obtained products are air-stable solids and can be synthesis on multigram scale with high yields. They are soluble in organic solvents like DCM, THF and toluene but not in, e.g. methanol, MeCN. They were isolated an characterized by means of spectroscopic methods and elemental analysis.

It is worth mentioning that hydrosilylation may not proceed with a complete selectivity control towards the desired product and side reactions may occur. There is one exception from the *β*-addition product exclusive formation during hydrosilylation reaction of DDSQ moieties with T_8_ derivatives, i.e. the reaction of **DDSQ-2SiVi** with ***i*Oc**_**7**_**T**_**8**_**H**. Careful evaluation of ^29^Si NMR analysis of **DDSQ-2Si-(T**_**8**_***i*Oc**_**7**_**)**_**2**_ revealed multiplied signals for Si^M^ and Si^Q^ from ***i*Oc**_**7**_**T**_**8**_ and also Si^D^ from **DDSQ-2Si** (See supplementary materials, Fig. S27) which is characteristics for *α*-hydrosilylation product formation. This might be connected with the steric hindrance of bulky *i*Oc groups. However, this was not observed for the hydrosilylation of ***i*Oc**_**7**_**T**_**8**_**H** with **DDSQ-2OSiVi**. Therefore presence of *α*-hydrosilylation product in the case of **DDSQ-2Si-(T**_**8**_***i*Oc**_**7**_**)**_**2**_ might be related with other steric but also electron aspects of the reagent. Vinyl groups in **DDSQ-2SiVi** are connected directly to the DDSQ core while in **DDSQ-2OSiVi** they are separated by additional siloxane linkers (-OSiMe_2_-). This might explain the formation of only *β*-addition product in the case of **DDSQ-2OSi-(T**_**8**_**iOc**_**7**_**)**_**2**_. For that reason, additional tests of ***i*Oc**_**7**_**T**_**8**_**H** hydrosilylation reaction with **DDSQ-2SiVi** with different catalysts known for their reactivity in that process, i.e. [IrCl(cod)]_2_^69^, PtO_2_/XPhos^70^, Pt/SDB^42^ were performed (see ESI). Unfortunately none of them did ensure the complete conversion of Si-H bond.

### Computational studies

Our simulations allowed us to find potential energy minima corresponding to the studied systems consisted of four molecules of **Ph**_**7**_**T**_**8**_**-T**_**8**_**Et**_**7**_ compound. Clustering analysis helped identify eight clusters based on simulation trajectory (coordinates of average structures of those systems are included in supplementary information Table S1–S8). Clusters numbering depend on how many structures from simulation trajectory fit to specific cluster (1 is for the biggest number of structures, 8 is for the lowest number of structures). These structures were used to obtain energies of the systems^62^.

**Figure 8:**
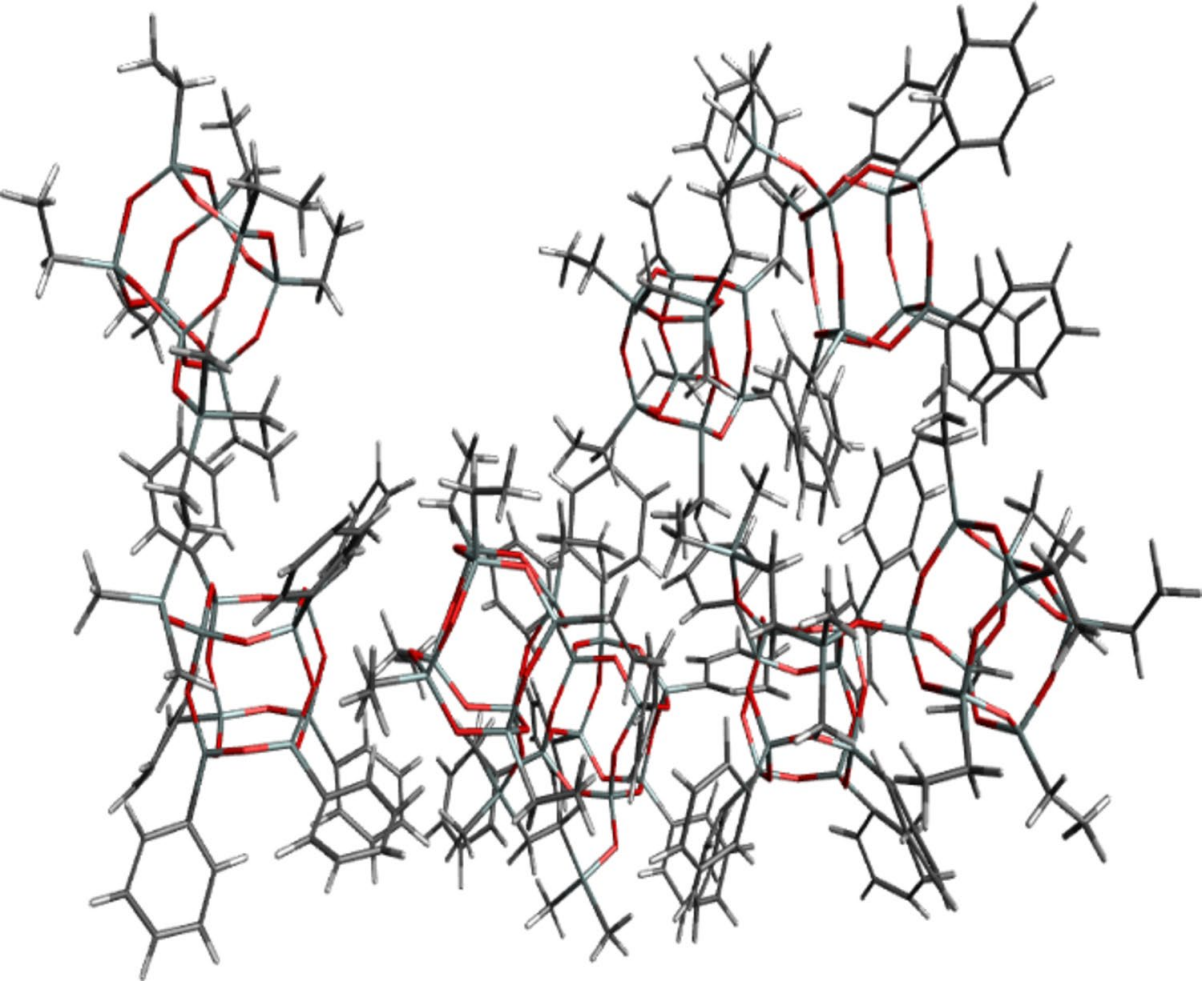
Structure of cluster **2** system consisted of four **Ph**_**7**_**T**_**8**_**-T**_**8**_**Et**_**7**_ compounds.

The energetically favored cluster **2** (see Table 1) consisted of four molecules of **Ph**_**7**_**T**_**8**_**-T**_**8**_**Et**_**7**_; it is depicted in Fig. 8.

**Table 1:** Relative energy of average structures of cluster obtained from simulations with four **Ph**_**7**_**T**_**8**_**-T**_**8**_**Et**_**7**_ molecules.

System obtained from four **Ph**_**7**_**T**_**8**_**-T**_**8**_**Et**_**7**_ structures	Relative energy [kcal/mol]
Cluster **1**	121.1
Cluster **2**	0.0^a^
Cluster **3**	79.0
Cluster **4**	406.4
Cluster **5**	170.1
Cluster **6**	225.1
Cluster **7**	112.0
Cluster **8**	197.1

Absolute energy [hartree]: ^a^ −38337.7352919.

**Figure 9:**
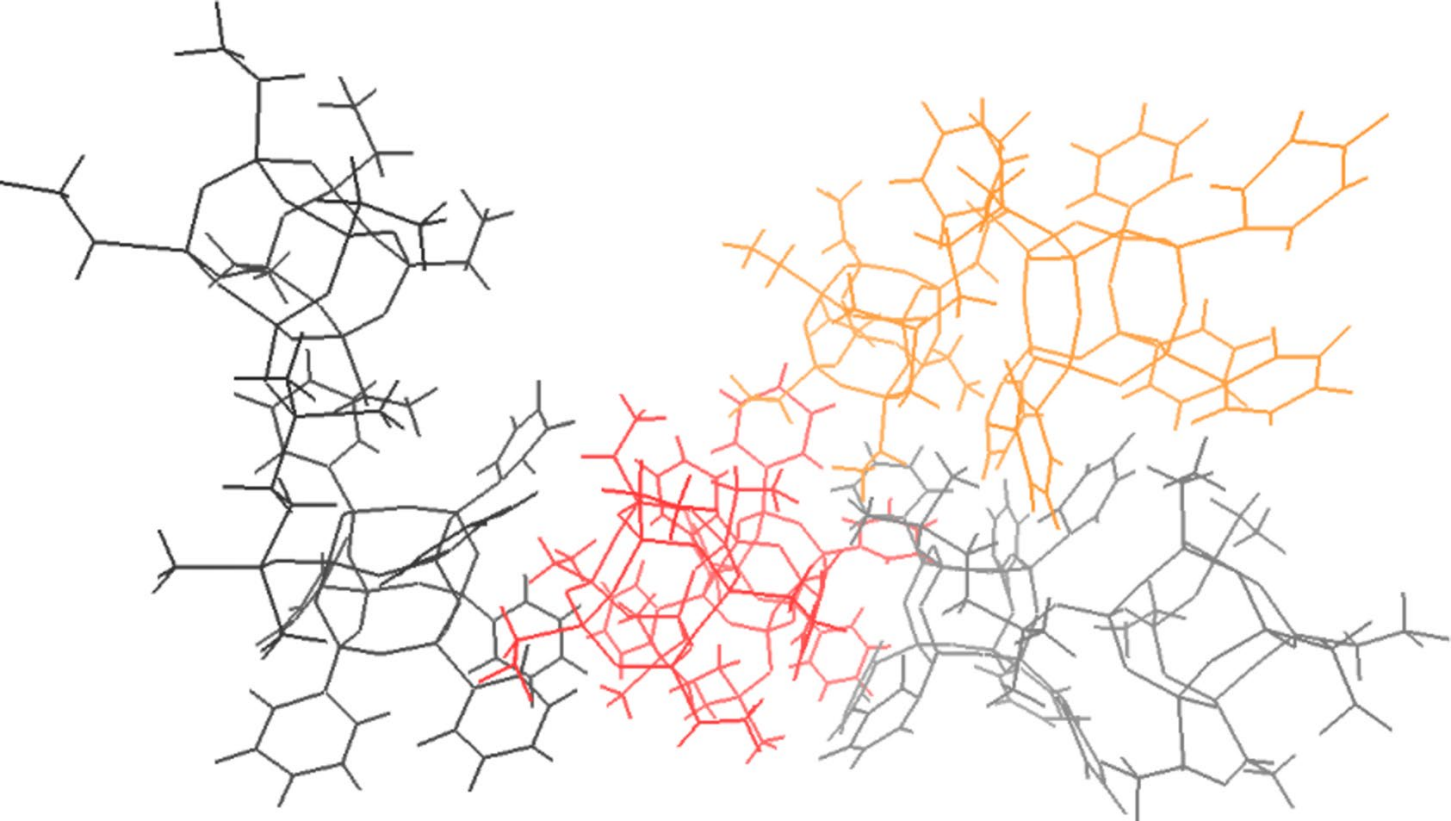
Structure of cluster **2** system consisted of four **Ph**_**7**_**T**_**8**_**-T**_**8**_**Et**_**7**_ compounds coloured in red, grey, orange and black.

As it can be observed the aggregation of **Ph**_**7**_**T**_**8**_**-T**_**8**_**Et**_**7**_ molecules is so strong that it is hard to see four molecules separately. Figure 9 shows those four molecules coloured in red, grey, orange and black.

It can be observed that ‘red’ molecule of **Ph**_**7**_**T**_**8**_**-T**_**8**_**Et**_**7**_ is oriented with its ethyl groups to phenyl groups of black molecule. For orange and grey molecules it was observed that their ethyl groups are oriented to their phenyl groups. Ethyl groups of orange molecule and phenyl groups of grey molecule are also oriented to phenyl groups of red molecule.

Interatomic distances of each of obtained structures of **Ph**_**7**_**T**_**8**_**-T**_**8**_**Et**_**7**_ molecule were calculated on the basis of their coordinates. For cluster 2 with lowest energy four molecules of **Ph**_**7**_**T**_**8**_**-T**_**8**_**Et**_**7**_ had the interatomic distance respectively 22.72, 19.91, 19.79 and 20.46 Å. (See supplementary materials Table S4). The biggest interatomic distances for **Ph**_**7**_**T**_**8**_**-T**_**8**_**Et**_**7**_ molecule had cluster 5 (23.56 Å) and the lowest had cluster 8 (19.22 Å). From all of the obtained data it can be noticed that the lowest energy cluster is consisted of three **Ph**_**7**_**T**_**8**_**-T**_**8**_**Et**_**7**_ molecules with smaller interatomic distances (19.91, 19.79 and 20.46 Å) and one more extended conformation of the molecule (22.72 Å).

**Figure 10:**
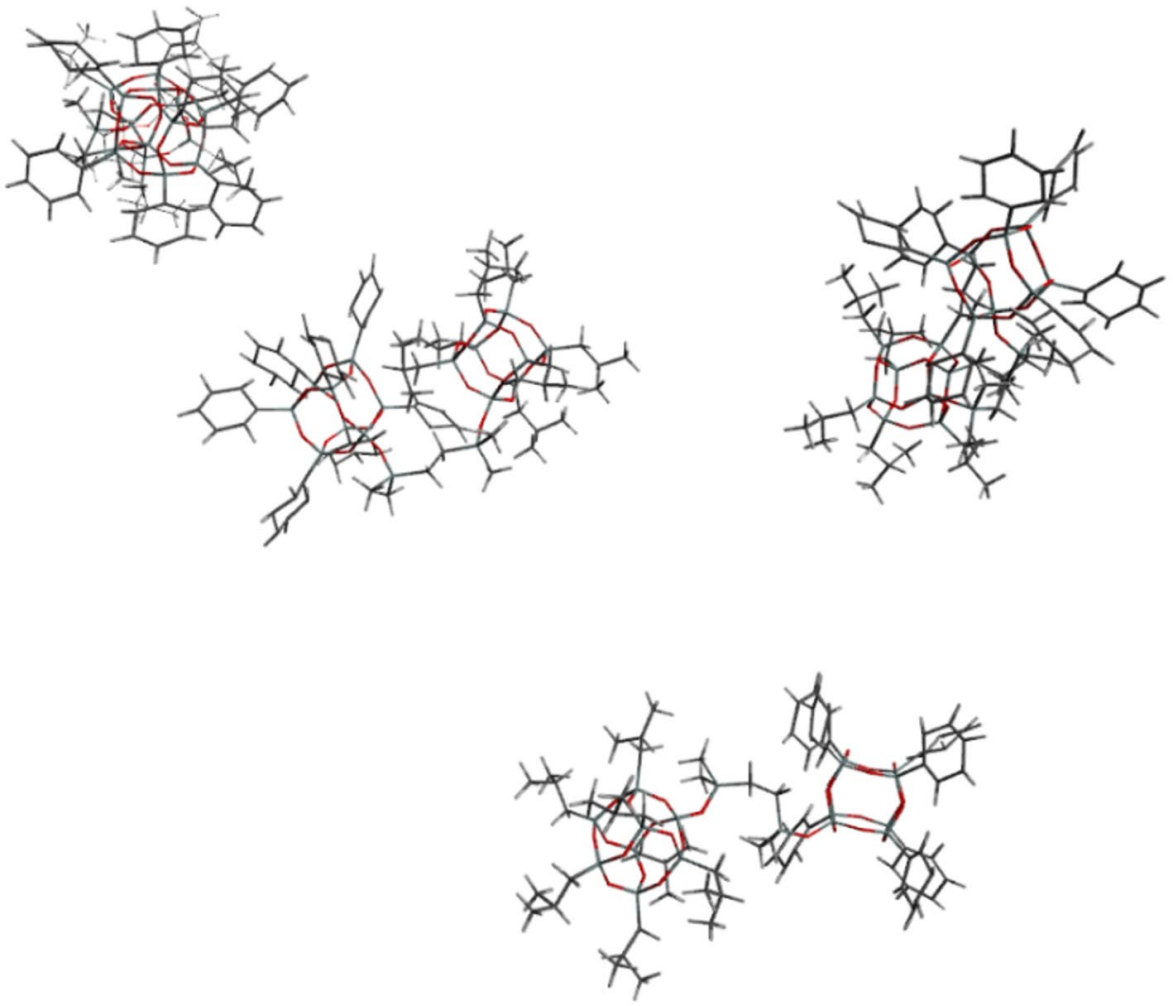
Structure of cluster **5** system consisted of four **Ph**_**7**_**T**_**8**_**-T**_**8**_**iBu**_**7**_ compounds.

From all investigated systems (coordinates of average structures of those systems are included in supplementary information Table S5–S7) consisted of four molecules of **Ph**_**7**_**T**_**8**_**-T**_**8**_***i*Bu**_**7**_ the lowest energy had cluster 5 (see Table 2). Structure of cluster 5 system is depicted in Fig. 10.

**Table 2:** Relative energy of average structures of cluster obtained from simulations with four **Ph**_**7**_**T**_**8**_**-T**_**8**_**iBu**_**7**_ molecules.

System obtained from four **Ph**_**7**_**T**_**8**_**-T**_**8**_***i*Bu**_**7**_ structures	Relative energy [kcal/mol]
Cluster **1**	214.7
Cluster **2**	29.5
Cluster **3**	75.3
Cluster **4**	496.1
Cluster **5**	0.0^b^
Cluster **6**	172.8
Cluster **7**	292.3
Cluster **8**	262.9

Absolute energy [hartree]: ^b^ −40537.0979796.

In comparison to clusters observed for **Ph**_**7**_**T**_**8**_**-T**_**8**_**Et**_**7**_ in the case of **Ph**_**7**_**T**_**8**_**-T**_**8**_***i*Bu**_**7**_ clusters do not show interaction between four **Ph**_**7**_**T**_**8**_**-T**_**8**_***i*Bu**_**7**_ molecules. In all eight obtained clusters of **Ph**_**7**_**T**_**8**_**-T**_**8**_***i*Bu**_**7**_ the molecules interact with solvent molecules (chloroform) but not with one another - they are not close enough each other to say that they are aggregating.

From all structures of **Ph**_**7**_**T**_**8**_**-T**_**8**_***i*Bu**_**7**_ the largest interatomic distance was observed in cluster 3 (28.44Å). The smallest one in cluster 2 (23.21 Å). Cluster 5 with the lowest energy had structures with largest interatomic distances of 24.79, 28.07, 24.03 and 24.05 Å. Similarly as it was observed for **Ph**_**7**_**T**_**8**_**-T**_**8**_**Et**_**7**_ compound the energetically favoured cluster had three molecules of **Ph**_**7**_**T**_**8**_**-T**_**8**_***i*Bu**_**7**_ in the most compact conformation while the forth molecule was present in the extended conformation (See supplementary materials Table S8).

## Conclusions

This study provides insights through experimental and computational methods on understanding silsesquioxane molecule aggregation for tailoring their future properties as nanocomposites, coatings, drug delivery systems, and catalysts.

In summary, we have developed an efficient and selective approach to synthesize Janus-type inorganic-organic molecules based on monofunctionalized silsesquioxanes (R_7_T_8_, where R = Et, Ph, *i*Bu, *i*Oc) and difunctional double-decker symmetric systems (DDSQ-2SiVi and DDSQ-2OSiVi). We revealed simple and effective methods for their synthesis and isolation, achieving high purity and thorough characterization. This work represents the first demonstration of the catalytic reactivity of monofunctionalized silsesquioxanes in hydrosilylation reactions with other monofunctional silsesquioxanes (leading to the formation of R_7_T_8_-T_8_R’_7_) and of di-substituted DDSQs with various monofunctional silsesquioxanes (resulting in DDSQ-2Si-(T_8_R_7_)_2_ and DDSQ-2OSi-(T_8_R_7_)_2_). All compounds were comprehensively investigated using NMR, FT-IR spectroscopy, and elemental analysis. Looking ahead, we anticipate developing new Janus compounds by modifying phenyl groups attached to the Si-O-Si core through halogenation and catalytic reactions such as Heck, Sonogashira, and Suzuki coupling. Computational investigations revealed the aggregation behavior of the synthesized molecules and enabled visualization of the most energetically preferred structures. Molecular modeling and DFT calculations provided detailed information on the interatomic distances within the compounds, depending on their interactions within studied clusters. This study offers valuable understanding through both experimental and computational methods, enhancing our comprehension of silsesquioxane molecule aggregation. These insights are crucial for tailoring their properties for future applications in nanocomposites, coatings, drug delivery systems, and catalysts.

### Supplementary Information

Supplementary Information.

## Data Availability

The authors declare that the data supporting the findings of this study are available within the paper and its Supplementary Information files. Should any raw data files be needed in another format they are available from the corresponding author upon reasonable request.

## References

[1] POSS - Hybrid Plastics. *Regist. trademark*.

[2] Cordes, D. B., Lickiss, P. D. & Rataboul, F. Recent developments in the chemistry of cubic polyhedral. *Chem. Rev.* **110**, 2081–2173 (2010). 20225901 10.1021/cr900201r

[3] Laird, M. *et al.* Large polyhedral oligomeric silsesquioxane cages: The isolation of functionalized POSS with an unprecedented Si18O27 core. *Angew. Chem. Int. Ed.* **60**, 3022–3027 (2021). 10.1002/anie.202010458 33043577

[4] Dudziec, B. & Marciniec, B. Double-decker silsesquioxanes: Current chemistry and applications. *Curr. Org. Chem.* **21**, 2794–2813 (2017).

[5] Wang, M., Chi, H., Joshy, K. S. & Wang, F. Progress in the synthesis of bifunctionalized polyhedral oligomeric silsesquioxane. *Polymers (Basel)* **11**, 2098–2118 (2019). 31847358 10.3390/polym11122098 PMC6960853

[6] Li, L., Wang, H. & Zheng, S. Well-defined difunctional POSS macromers and related organic–inorganic polymers: Precision synthesis, structure and properties. *J. Polym. Sci.*10.1002/pol.20230428 (2023). 10.1002/pol.20230428

[7] Ye, Q., Zhou, H. & Xu, J. Cubic polyhedral oligomeric silsesquioxane based functional materials: Synthesis, assembly, and applications. *Chem. Asian J.* **11**, 1322–1337 (2016). 26879136 10.1002/asia.201501445

[8] Zhou, H., Ye, Q. & Xu, J. Polyhedral oligomeric silsesquioxane-based hybrid materials and their applications. *Mater. Chem. Front.* **1**, 212–230 (2017). 10.1039/C6QM00062B

[9] Gon, M., Tanaka, K. & Chujo, Y. Recent progress on designable hybrids with stimuli-responsive optical properties originating from molecular assembly concerning polyhedral oligomeric silsesquioxane. *Chem. Asian J.* **17**, e202200144 (2022). 35322576 10.1002/asia.202200144

[10] Calabrese, C., Aprile, C., Gruttadauria, M. & Giacalone, F. POSS nanostructures in catalysis. *Catal. Sci. Technol.* **10**, 7415–7447 (2020). 10.1039/D0CY01407A

[11] Wang, L. *et al.* Multi-stimuli-responsive nanoparticles formed of POSS-PEG for the delivery of boronic acid-containing therapeutics. *Biomacromolecules* **24**, 5071–5082 (2023). 37691317 10.1021/acs.biomac.3c00677

[12] Jafari, M. *et al.* Dendritic hybrid materials comprising polyhedral oligomeric silsesquioxane (POSS) and hyperbranched polyglycerol for effective antifungal drug delivery and therapy in systemic candidiasis. *Nanoscale* **15**, 16163–16177 (2023). 37772640 10.1039/D3NR04321E

[13] Poggi, E. & Gohy, J. F. Janus particles: From synthesis to application. *Colloid Polym. Sci.* **295**, 2083–2108 (2017). 10.1007/s00396-017-4192-8

[14] Walther, A. & Mu, A. H. E. Janus particles: Synthesis, self-assembly, physical properties, and applications. *Chem. Rev.*10.1021/cr300089t (2013). 23557169 10.1021/cr300089t

[15] Synytska, A., Khanum, R., Ionov, L., Cherif, C. & Bellmann, C. Water-repellent textile via decorating fibers with amphiphilic Janus Particles. *ACS Appl. Mater. Interfaces* **3**, 1216–1220 (2011). 21366338 10.1021/am200033u

[16] Walther, A. & Müller, A. H. E. Janus particles. *Soft Matter* **4**, 663–668 (2008). 32907169 10.1039/b718131k

[17] Xu, L., Pradhan, S. & Chen, S. Adhesion force studies of Janus nanoparticles. *Langmuir*10.1021/la700774g (2007). 17595125 10.1021/la700774g

[18] Wu, L. Y., Ross, B. M., Hong, S. & Lee, L. P. Bioinspired nanocorals with decoupled cellular targeting and sensing functionality **. *Small*10.1002/smll.200901604 (2010). 20108232 10.1002/smll.200901604

[19] Valadares, L. F. *et al.* Catalytic nanomotors: Self-propelled sphere dimers. *Small*10.1002/smll.200901976 (2010). 20108240 10.1002/smll.200901976

[20] Chinnam, P. R. & Wunder, S. L. Polyoctahedral silsesquioxane-nanoparticle electrolytes for lithium batteries: POSS-lithium salts and POSS-PEGs. *Chem. Mater.* **23**, 5111–5121 (2011). 10.1021/cm2015675

[21] Han, D., Zhang, Q., Chen, F. & Fu, Q. RSC advances using POSS—C 60 giant molecules as a novel compatibilizer for PS / PMMA polymer blends †. *RSC Adv.* **6**, 18924–18928 (2016). 10.1039/C6RA00218H

[22] Han, D. *et al.* AC SC. *Polymer (Guildf).* **136**, 84–91 (2018). 10.1016/j.polymer.2017.12.050

[23] Anker, J. N., Behrend, C. J., Huang, H. & Kopelman, R. Magnetically-modulated optical nanoprobes (MagMOONs) and systems. *J. Magn. Magn. Mater.* **293**, 655–662 (2005). 10.1016/j.jmmm.2005.01.031

[24] Xu, H., Aylott, J. W., Kopelman, R., Miller, T. J. & Philbert, M. A. A real-time ratiometric method for the determination of molecular oxygen inside living cells using sol-gel-based spherical optical nanosensors with applications to rat C6 glioma. *Anal. Chem.* **73**, 4124–4133 (2001). 11569801 10.1021/ac0102718

[25] Behrend, C. J. *et al.* Metal-capped Brownian and magnetically modulated optical nanoprobes (MOONs): Micromechanics in chemical and biological microenvironments †. *J. Phys. Chem. B* **108**, 10408–10414 (2004). 10.1021/jp040125g

[26] Tanaka, T., Hasegawa, Y., Kawamori, T., Kunthom, R. & Takeda, N. Synthesis of double-decker silsesquioxanes from substituted difluorosilane. *Organometallics*10.1021/acs.organomet.8b00896 (2018). 10.1021/acs.organomet.8b00896

[27] Asuncion, M. Z., Ronchi, M., Abu-Seir, H. & Laine, R. M. Synthesis, functionalization and properties of incompletely condensed ‘half cube’ silsesquioxanes as a potential route to nanoscale Janus particles. *Comptes Rendus Chim.* **13**, 270–281 (2010). 10.1016/j.crci.2009.10.007

[28] Oguri, N., Egawa, Y., Takeda, N. & Unno, M. Janus-cube octasilsesquioxane: Facile synthesis and structure elucidation. *Angew. Chem. Int. Ed.* **55**, 9336–9339 (2016). 10.1002/anie.201602413 27225052

[29] Shiba, H., Yoshikawa, M., Wada, H., Shimojima, A. & Kuroda, K. Synthesis of polycyclic and cage siloxanes by hydrolysis and intramolecular condensation of alkoxysilylated cyclosiloxanes. *Chem. Eur. J.*10.1002/chem.201805942 (2019). 30600848 10.1002/chem.201805942

[30] Blázquez-Moraleja, A., Pérez-Ojeda, M. E., Ramón Suárez, J., Jimeno, M. L. & Chiara, J. L. Chemical communications. *Chem. Commun.* **52**, 5792–5795 (2016). 10.1039/C6CC00896H 26948377

[31] Chen, X. *et al.* Science of the total environment single step synthesis of Janus nano-composite membranes by atmospheric aerosol plasma polymerization for solvents separation. *Sci. Total Environ.* **645**, 22–33 (2018). 30015115 10.1016/j.scitotenv.2018.06.343

[32] Meng, Y., Li, W., Kunthom, R., Liu, H. Rational Design and Application of Superhydrophobic Fluorine-Free Coating Basedon Double-Decker Silsesquioxane for Oil-Water Separation. Polymer 304, 127143 (2024) 10.1016/j.polymer.2024.127143

[33] Li, W.; Liu, H. Rational Design and Facile Preparation of Hybrid Superhydrophobic Epoxy Coatings Modified byFluorinated Silsesquioxane-Based Giant Molecules via Photo-Initiated Thiol-Ene Click Reaction with Potential Applications.Chem. Eng. J. 480, 147943 (2024) 10.1016/j.cej.2023.147943

[34] Laine, R. M. *et al.* Perfect and nearly perfect silsesquioxane (SQs) nanoconstruction sites and Janus SQs. *J. Sol Gel Sci. Technol.* **46**, 335–347 (2008). 10.1007/s10971-008-1700-9

[35] Liu, H. *et al.* Unraveling the self-assembly of hetero-cluster Janus dumbbells into hybrid cubosomes with internal double diamond structure. *J. Am. Chem. Soc.*10.1021/jacs.8b08016 (2018). 30501178 10.1021/jacs.8b08016

[36] Ma, C. *et al.* A filled-honeycomb-structured crystal formed by self-assembly of a Janus polyoxometalate – silsesquioxane (POM – POSS) co-cluster Angewandte. *Angew. Chem. Int. Ed. Engl.* **54**, 15699–15704 (2015). 26563587 10.1002/anie.201507237

[37] Wang, F., Phonthammachai, N., Mya, K. Y., Tjiu, W. W. & He, C. PEG-POSS assisted facile preparation of amphiphilic gold nanoparticles and interface formation of Janus nanoparticles. *Chem. Commun.* **47**, 767–769 (2011). 10.1039/C0CC02082F 21069220

[38] Liu, H. *et al.* Manipulation of self-assembled nanostructure dimensions in molecular Janus particles. *ACS Nano*10.1021/acsnano.6b01336 (2016). 27337531 10.1021/acsnano.6b01336

[39] Liu, H. *et al.* Two-dimensional nanocrystals of molecular Janus particles. *J. Am. Chem. Soc.* **136**, 10691–10699 (2014). 25029032 10.1021/ja504497h

[40] Marciniec, B., Pietraszuk, C., Pawluć, P. & Maciejewski, H. Inorganometallics (transition metal-metalloid complexes) and catalysis. *Chem. Rev.* **122**, 3996–4090 (2022). 34967210 10.1021/acs.chemrev.1c00417 PMC8832401

[41] Troegel, D. & Stohrer, J. Recent advances and actual challenges in late transition metal catalyzed hydrosilylation of olefins from an industrial point of view. *Coord. Chem. Rev.* **255**, 1440–1459 (2011). 10.1016/j.ccr.2010.12.025

[42] Walczak, M. *et al.* Hydrosilylation of alkenes and alkynes with silsesquioxane (HSiMe2O)(i-Bu)7Si8O12 catalyzed by Pt supported on a styrene-divinylbenzene copolymer. *J. Catal.* **367**, 1–6 (2018). 10.1016/j.jcat.2018.08.012

[43] Walczak, M. *et al.* Unusual cis- and trans- architecture of dihydrofunctional double-decker shaped silsesquioxane – design and construction of its ethyl bridged π-conjugated arene derivatives. *New J. Chem.* **41**, 3290–3296 (2017). 10.1039/C7NJ00255F

[44] Mituła, K., Dutkiewicz, M., Dudziec, B., Marciniec, B. & Czaja, K. A library of monoalkenylsilsesquioxanes as potential comonomers for synthesis of hybrid materials. *J. Therm. Anal. Calorim.* **132**, 1545–1555 (2018). 10.1007/s10973-018-7121-2

[45] Duszczak, J. *et al.* Distinct insight into the use of difunctional double-decker silsesquioxanes as building blocks for alternating A-B type macromolecular frameworks. *Inorg. Chem. Front.* **10**, 888–899 (2022). 10.1039/D2QI02161G

[46] Mrzygłód, A., Rzonsowska, M. & Dudziec, B. Exploring polyol-functionalized dendrimers with silsesquioxane cores. *Inorg. Chem.* **62**, 21343–21352 (2023). 38055955 10.1021/acs.inorgchem.3c03427

[47] Best, R. B. *et al.* Optimization of the additive CHARMM all-atom protein force field targeting improved sampling of the backbone φ, ψ and side-chain χ1 and χ2 dihedral angles. *J. Chem. Theory Comput.* **8**, 3257–3273 (2012). 23341755 10.1021/ct300400x PMC3549273

[48] Saam, J., Ivanov, I., Walther, M., Holzhütter, H. G. & Kuhn, H. Molecular dioxygen enters the active site of 12/15-lipoxygenase via dynamic oxygen access channels. *Proc. Natl. Acad. Sci. U. S. A.* **104**, 13319–13324 (2007). 17675410 10.1073/pnas.0702401104 PMC1948941

[49] Humphrey, W., Dalke, A. & Schulten, K. VMD: Visual molecular dynamics. *Proc. Natl. Acad. Sci. U. S. A.* **104**, 13319–13324 (2007). 8744570 10.1016/0263-7855(96)00018-5

[50] Becke, A. D. Density-functional exchange-energy approximation with correct asymptotic behavior. *Phys. Rev. Lett.* **38**, 3098–3100 (1988). 10.1103/physreva.38.3098 9900728

[51] Ditchfield, R., Hehre, W. J. & Pople, J. A. Self-consistent molecular-orbital methods. IX. An extended Gaussian-type basis for molecular-orbital studies of organic molecules. *J. Chem. Phys.* **54**, 720–723 (1971). 10.1063/1.1674902

[52] Reed, A. E., Weinstock, R. B. & Weinhold, F. Natural population analysis. *J. Chem. Phys.* **83**, 735–746 (1985). 10.1063/1.449486

[53] Frish, M. J. *et al.**Gaussian 09, Revision A.1* (Gaussian Inc., 2009).

[54] *Solvate Plugin, Version 1.5.* https://www.ks.uiuc.edu/Research/vmd/plugins/solva at (2021).

[55] *The Energy Function - CHARMM tutorial*. https://www.charmmtutorial.org/index.php/The_Energ at (2021).

[56] Kirkpatrick, S., Gelatt, C. D. & Vecchi, M. P. Optimization by simulated annealing. *Science* **220**, 671–680 (1983). 17813860 10.1126/science.220.4598.671

[57] Franz, A., Hoffmann, K. H. & Salamon, P. Best possible strategy for finding ground states. *Phys. Rev. Lett.* **86**, 5219–5222 (2001). 11384462 10.1103/PhysRevLett.86.5219

[58] Allen, M. P. & Tildesley, D. J. *Computer Simulation of Liquids* (Clarendon Press, 1989).

[59] Phillips, J. C. *et al.* Scalable molecular dynamics with NAMD. *J. Comput. Chem.* **26**, 1781–1802 (2005). 16222654 10.1002/jcc.20289 PMC2486339

[60] Coutsias, E. A., Seok, C. & Dill, K. A. Using quaternions to calculate RMSD. *J. Comput. Chem.* **25**, 1849–1857 (2004). 15376254 10.1002/jcc.20110

[61] Heyer, L. J., Kruglyak, S. & Yooseph, S. Exploring expression data identification and analysis of coexpressed genes. *Genome Res.* **9**, 1106–1115 (1999). 10568750 10.1101/gr.9.11.1106 PMC310826

[62] *Clustering plugin for VMD.* http://physiology.med.cornell.edu/faculty/hweinste at (2019).

[63] Becke, A. D. Density-functional thermochemistry. III. The role of exact exchange. *J. Chem. Phys.* **98**, 5648–5652 (1993). 10.1063/1.464913

[64] Petersson, G. A., Mohammad, A. & Laham, A. A complete basis set model chemistry. II. The total energies of open-shell atoms and hydrides of the first-row atoms. *J. Chem. Phys.* **9**, 6081–6090 (1991). 10.1063/1.460447

[65] Asaduzzaman, A., Runge, K., Muralidharan, K., Deymier, P. A. & Zhang, L. Energetics of substituted polyhedral oligomeric silsesquioxanes: A DFT study. *MRS Commun.* **5**, 519–524 (2015). 10.1557/mrc.2015.58

[66] Asaduzzaman, A., Runge, K., Deymier, P. A. & Muralidharan, K. The role of aluminum substitution on the stability of substituted polyhedral oligomeric silsesquioxanes. *Zeitschrift Fur Phys. Chem.* **230**, 1005–1014 (2016). 10.1515/zpch-2015-0706

[67] Muya, J. T., Ceulemans, A., Gopakumar, G. & Parish, C. A. Jahn-teller distortion in polyoligomeric silsesquioxane (POSS) cations. *J. Phys. Chem. A* **119**, 4237–4243 (2015). 25831095 10.1021/acs.jpca.5b01787

[68] Tomasi, J., Mennucci, B. & Cammi, R. Quantum mechanical continuum solvation models. *Chem. Rev.* **105**, 2999–3093 (2005). 16092826 10.1021/cr9904009

[69] Sokolnicki, T., Franczyk, A., Janowski, B. & Walkowiak, J. Synthesis of bio-based silane coupling agents by the modification of eugenol. *Adv. Synth. Catal.* **363**, 5493–5500 (2021). 10.1002/adsc.202101178

[70] Stefanowska, K. *et al.* Selective hydrosilylation of alkynes with octaspherosilicate (HSiMe2O)8Si8O12. *Chem. Asian J.* **13**, 2101–2108 (2018). 10.1002/asia.201800726 29874414

[71] Spoljaric, S. & Shanks, R. A. Poly (styrene- b -butadiene- b -styrene)—dye-coupled polyhedral oligomeric silsesquioxanes. *Adv. Mater. Res.* **125**, 169–172 (2010). 10.4028/www.scientific.net/AMR.123-125.169

[72] Yuasa, S., Sato, Y., Imoto, H. & Naka, K. Thermal properties of open-cage silsesquioxanes: The effect of substituents at the corners and opening moieties. *Bulletin Chem. Soc. Jpn.* **92**, 127–132 (2019). 10.1246/bcsj.20180257

