# Supplementary material for: Preparation of T8 and double-decker silsesquioxane-based Janus-type molecules: molecular modeling and DFT insights
Source: Sci Rep. 2024 Aug 9;14:18527. doi: 10.1038/s41598-024-69481-6 (PMC11316061; doi:10.1038/s41598-024-69481-6)
Supplement: Supplementary file 1 — Supplementary Information. [file 41598_2024_69481_MOESM1_ESM.pdf]

Preparation of T_8 and Double-Decker Silsesquioxane-Based Janus-Type Molecules: Molecular Modeling and DFT Insights

Julia Duszczak-Kaczmarek[1,2], Katarzyna Mituła-Chmielowiec[1,2], Monika Rzonsowska[1,2], Wojciech Jankowski[1], Marcin Hoffmann[1], Jędrzej Walkowiak[2], Beata Dudziec[1,2,*]

[1]Faculty of Chemistry, Adam Mickiewicz University in Poznan,
Uniwersytetu Poznanskiego 8, 61-614 Poznan, Poland.
[2]Center for Advanced Technologies, Adam Mickiewicz University in Poznan,
Uniwersytetu Poznanskiego 10, 61-614 Poznan, Poland.
***beata.dudziec@gmail.com**

Table of Contents:

1. Table of isolated compounds………………………………………………………… ••• S-2
2. Data characterizing the obtained products (^{1}H, ^{13}C, ^{29}Si NMR spectra)………... ••• S-4-29
3. DFT Calculations…………………………………………………………………... ••• S-30-145

1 Table of isolated compounds:

Structure	Compound Abbrev.	NMR spectra page:
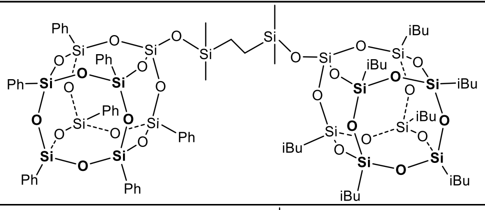	**Ph_7T_8-T_8*i*Bu_7**	S-4-5
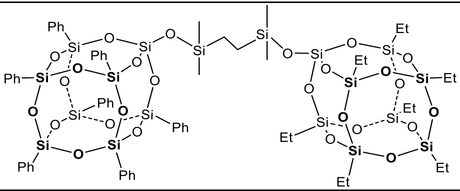	**Ph_7T_8-T_8Et_7**	S-6-7
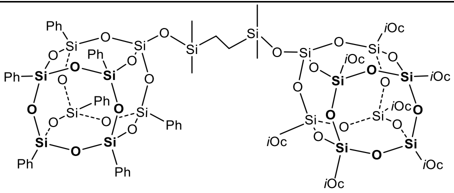	**Ph_7T_8-T_8*i*Oc_7**	S-8-9
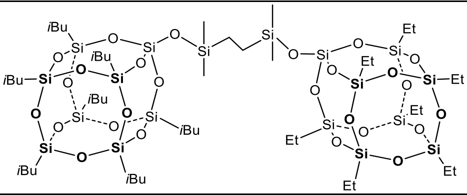	***i*Bu_7T_8-T_8Et_7**	S-10-11
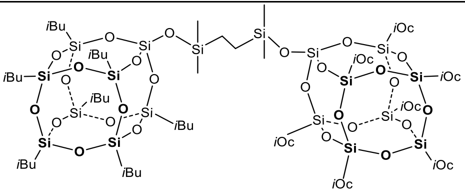	***i*Bu_7T_8-T_8*i*Oc_7**	S-12-13
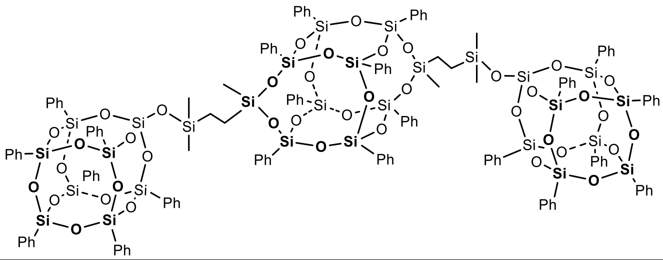	**DDSQ-2Si-(T_8Ph_7)$_2$**	S-14-15
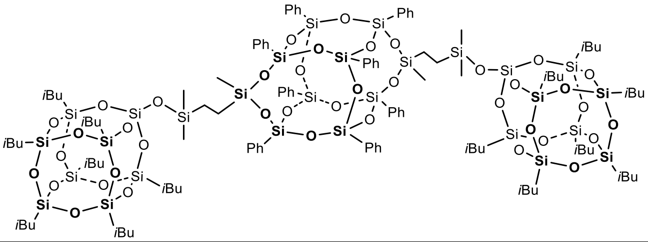	**DDSQ-2Si-(T_8*i*Bu_7)$_2$**	S-16-17

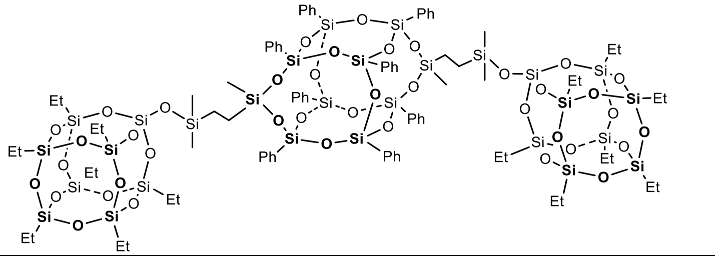	DDSQ-2Si-$(T_8Et_7)_2$	S-18-19
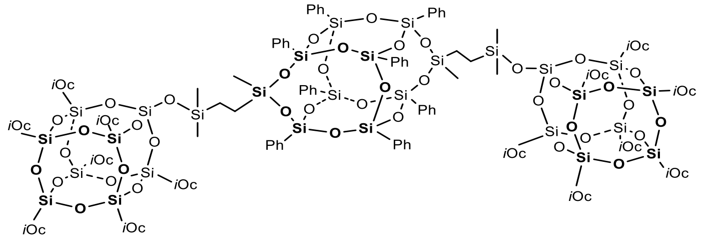	DDSQ-2Si-$(T_8\textit{i}Oc_7)_2$	S-20-21
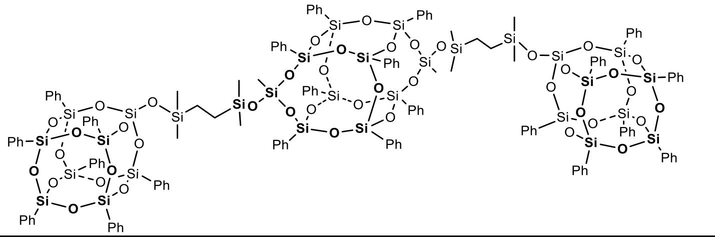	DDSQ-2OSi-$(T_8Ph_7)_2$	S-22-23
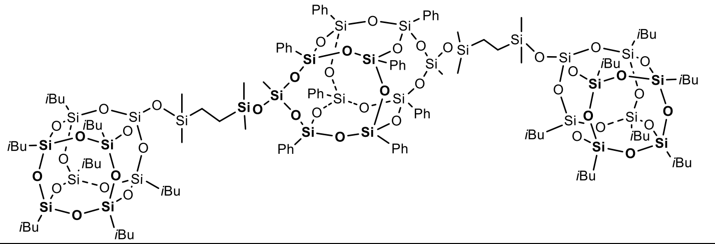	DDSQ-2OSi-$(T_8\textit{i}Bu_7)_2$	S-24-25
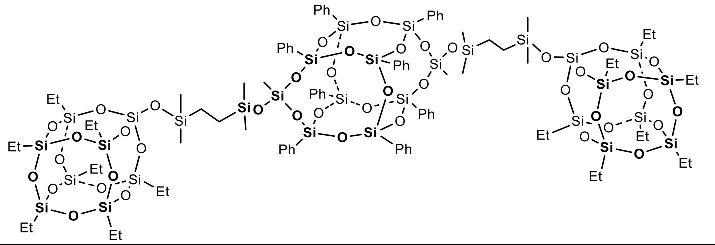	DDSQ-2OSi-$(T_8Et_7)_2$	S-26-27
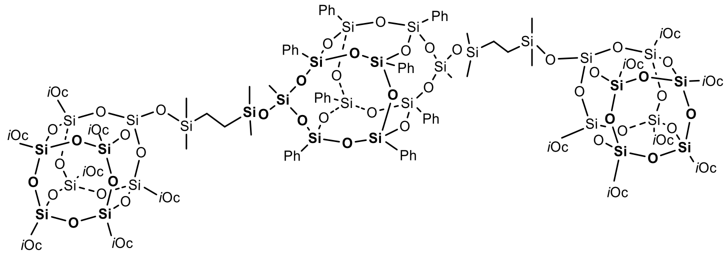	DDSQ-2OSi-$(T_8\textit{i}Oc_7)_2$	S-28-29

2 Characterization data of the hydrosilylation products:

Ph_7T_8-T_8iBu_7

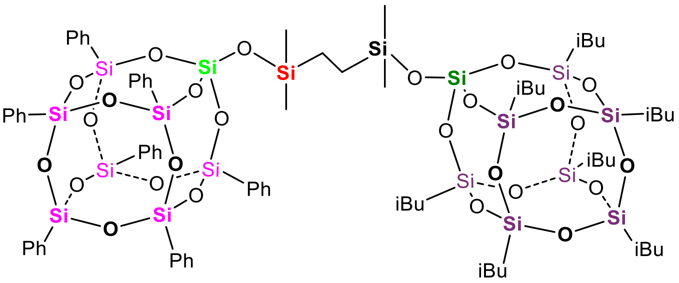

White solid. Isolated Yield 94%

^{1}H NMR (300 MHz, CD_2Cl_2, ppm): δ = 0.00 (s, 6H, $Si(CH_3)_2$), 0.11 (s, 6H, $Si(CH_3)_2$, 0.49-0.53 (m, 4H, $-CH_2-$), 0.60-0.62 (m, 14H, $-CH_2-$ *i*Bu), 0.93-0.98 (m, 42H, $-CH_3$ *i*Bu), 1.82-1.89 (m, 7H, -CH- *i*Bu), 7.37-7.80 (m, 35H, Ph);
^{13}C NMR (101 MHz, CD_2Cl_2, ppm): δ = -0.81 $(Si-CH_3)_2$, -0.70 $(Si-CH_3)_2$, 9.27 ($-CH_2-$), 22.88, 22.93, 24.46, 26.05 (*i*Bu), 128.55, 130.66, 131.49, 134.60 (Ph);
^{29}Si NMR (79 MHz, CD_2Cl_2, ppm): δ = 14.06 (Si^M), 12.33 (Si^M), -66.98, -67.76, -67.79 (Si^T-*i*Bu), -78.16, -78.33 (Si^T-Ph), -108.81 (Si^Q), -109.46 (Si^Q);
FT-IR (ATR, cm^{-1}): 3074.53 (C-H phenyl), 2954.34 (C-H), 2923.79, 2870.00 (C-H), 1466.42 (C-H), 1431.11 (C=C phenyl), 1229.14 (Si-C), 1079.35, 1028.78 (Si-O-Si), 997.92 (C-H phenyl).
EA: Anal. calcd for $C_{76}H_{114}O_{26}Si_{18}$ (%): C, 46.83, H, 5.90; found: C, 46.85; H, 5.91.

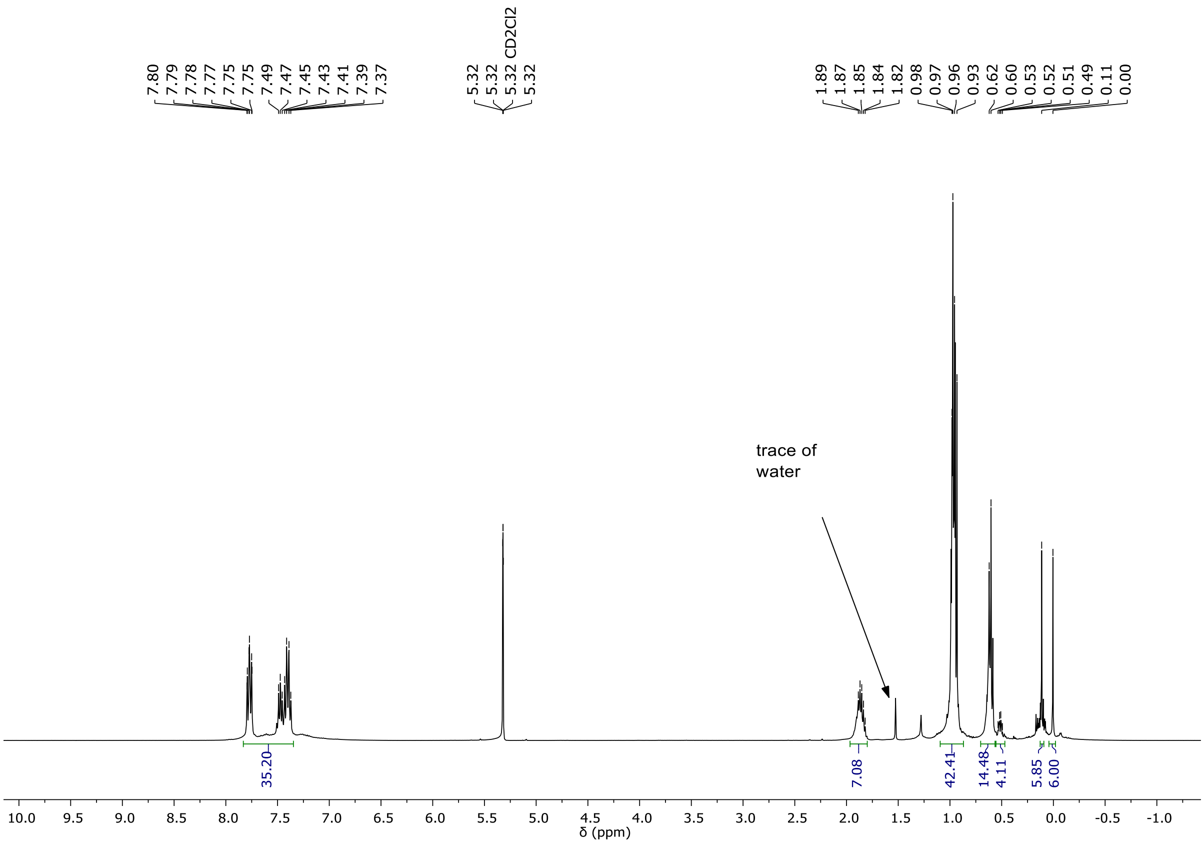

*Figure S1 ^{1}H NMR (300 MHz, CD_2Cl_2) spectrum of **Ph_7T_8-T_8iBu_7**.*

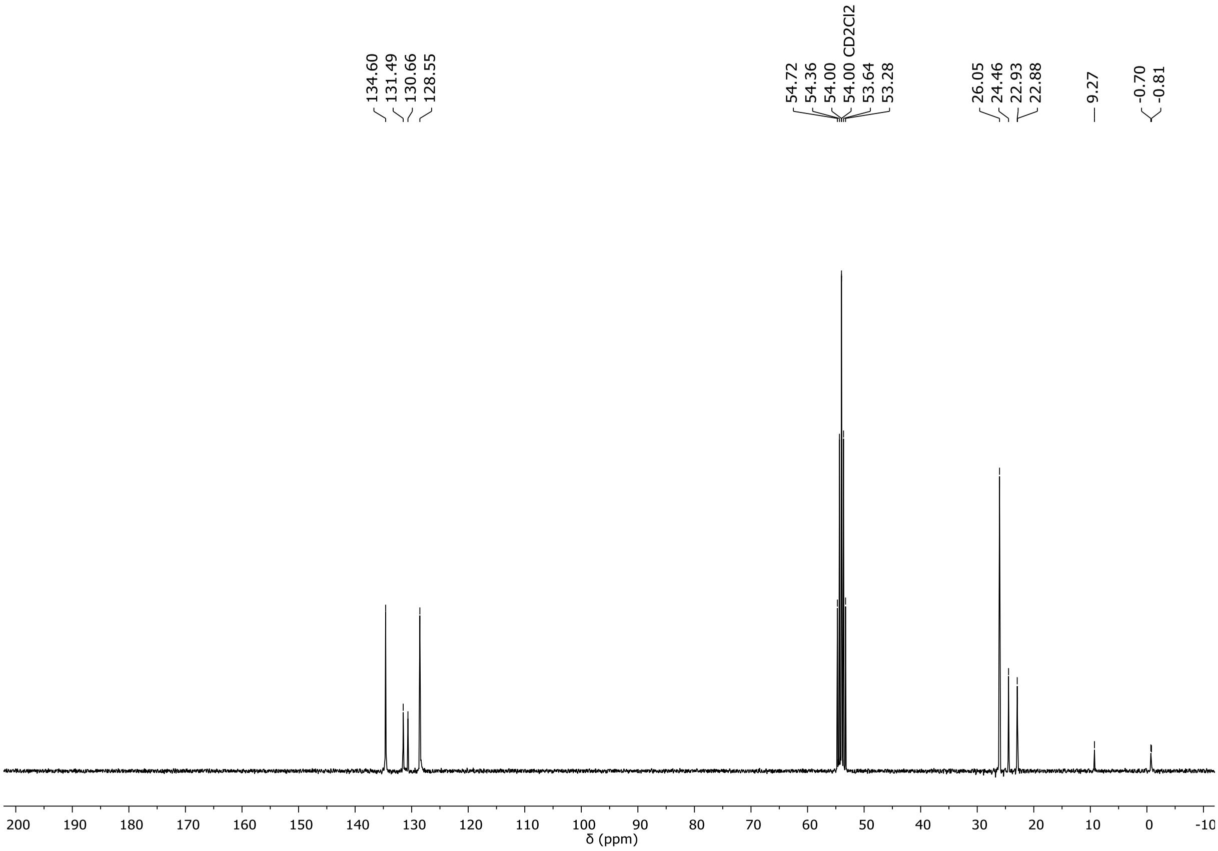

Figure S2 ^{13}*C NMR (101 MHz,* CD_2Cl_2*) spectrum of* ***Ph₇T₈-T₈iBu₇****.*

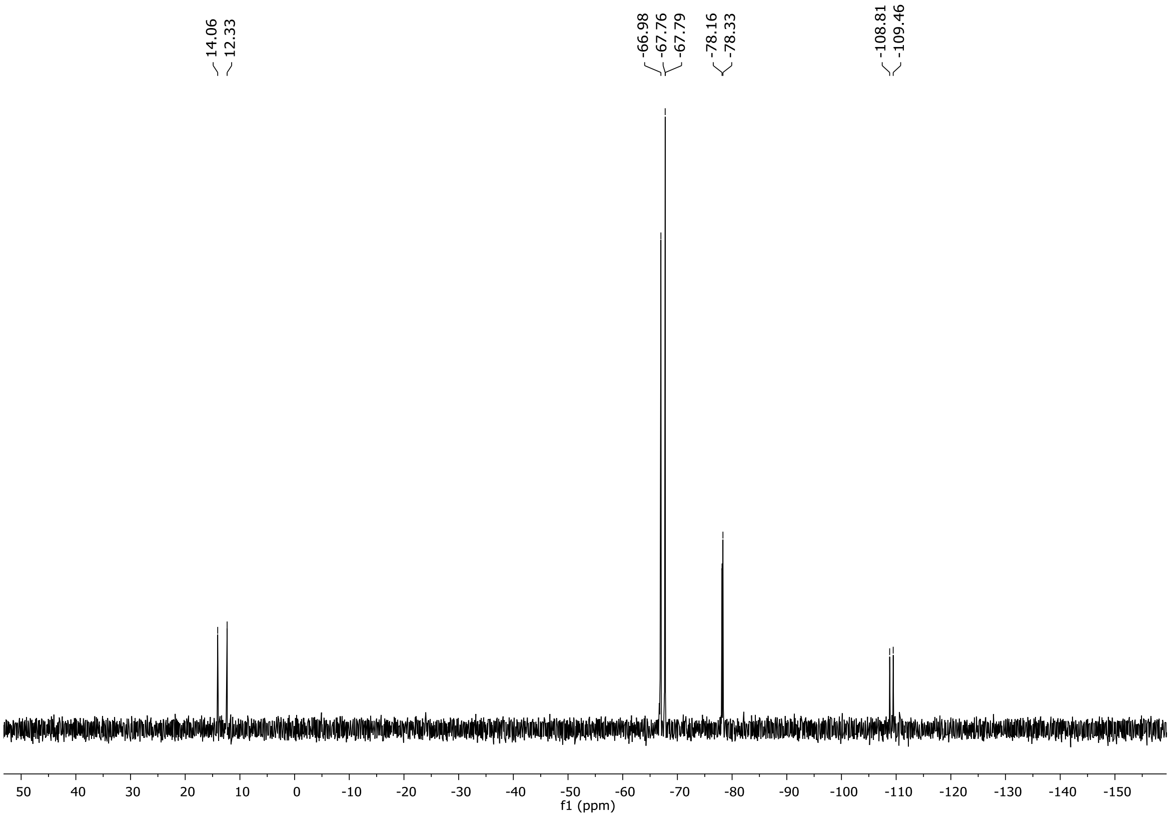

Figure S3 ^{29}*Si NMR (79 MHz,* CD_2Cl_2*) spectrum of* ***Ph₇T₈-T₈iBu₇****.*

Ph₇T₈-T₈Et₇

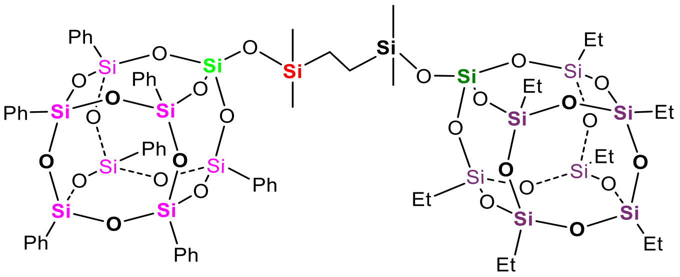

White solid. Isolated Yield 92%

^{1}H NMR (300 MHz, CD_2Cl_2, ppm): δ = -0.01 (s, 6H, $Si(CH_3)_2$), 0.10 (s, 6H, $Si(CH_3)_2$), 0.56-0.60 (m, 18H, $-CH_2-$, $-CH_2-$ Et), 0.94-1.01 (m, 21H, $-CH_3$ Et), 7.14-7.78 (m, 35H, Ph);
^{13}C NMR (101 MHz, CD_2Cl_2, ppm): δ = -0.83 (Si-$(CH_3)_2$), -0.74 (Si-$(CH_3)_2$), 4.50-4.53 ($-CH_3$ Et), 6.81-6.84 ($-CH_2-$ Et), 9.32 ($-CH_2-$), 128.53-128.76, 129.56, 130.70, 131.51, 134.63 (Ph);
^{29}Si NMR (79 MHz, CD_2Cl_2, ppm): δ = 14.06 (Si^M), 12.79 (Si^M), -65.10, -65.62 (Si^T-Et), -78.13, -78.31 (Si^T-Ph), -108.55 (Si^Q), -108.80 (Si^Q);
FT-IR (ATR, cm^{-1}): 3073.84, 3051.91 (C-H phenyl), 3029.10 (=C-H) 2961.67, 2880.15 (C-H), 1594.36, 1460.73 (C-H), 1430.73 (C=C phenyl), 1252.92 (Si-C), 1065.06 (Si-O-Si), 1011.23 (C-H phenyl).
EA: Anal. calcd for $C_{62}H_{86}O_{26}Si_{18}$ (%): C, 42.48, H, 4.95; found: C, 42.51; H, 4.93.

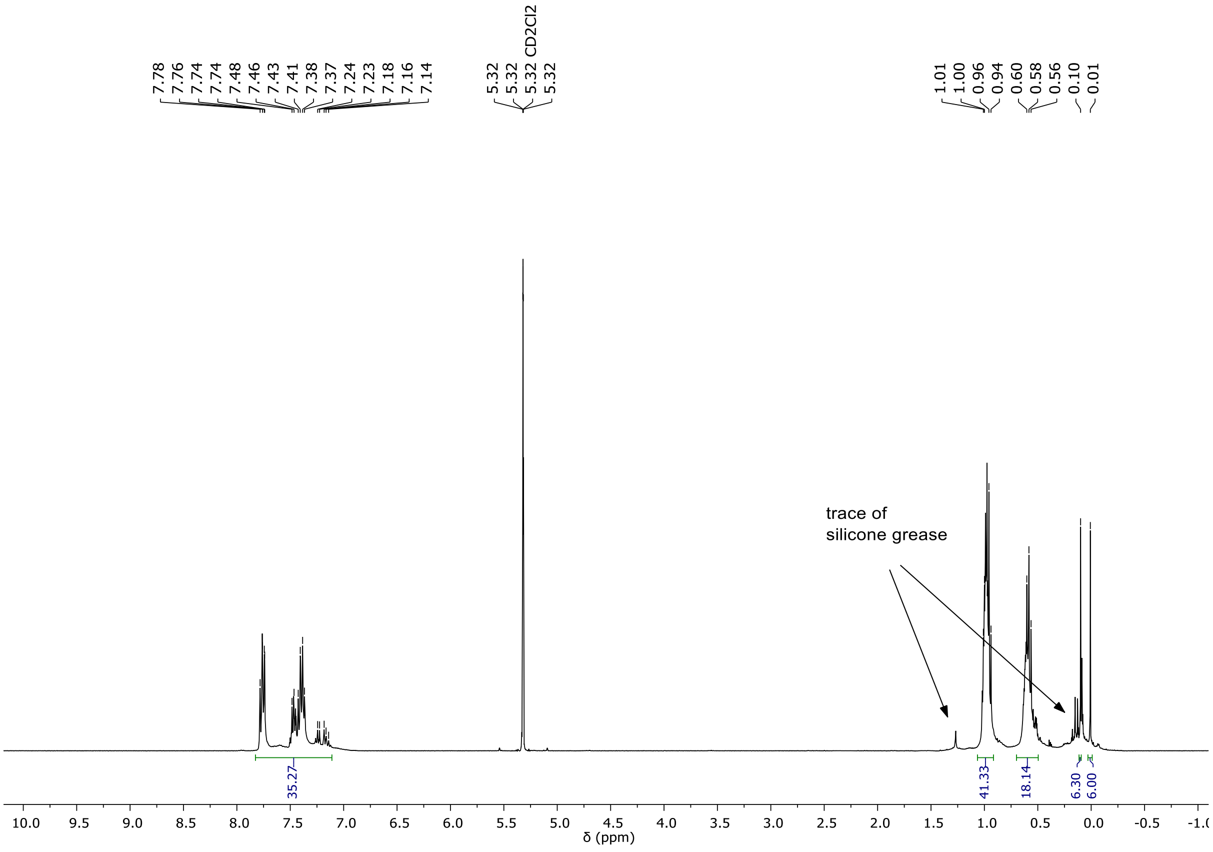

*Figure S4 ^{1}H NMR (300 MHz, CD_2Cl_2) spectrum of **Ph₇T₈-T₈Et₇**.*

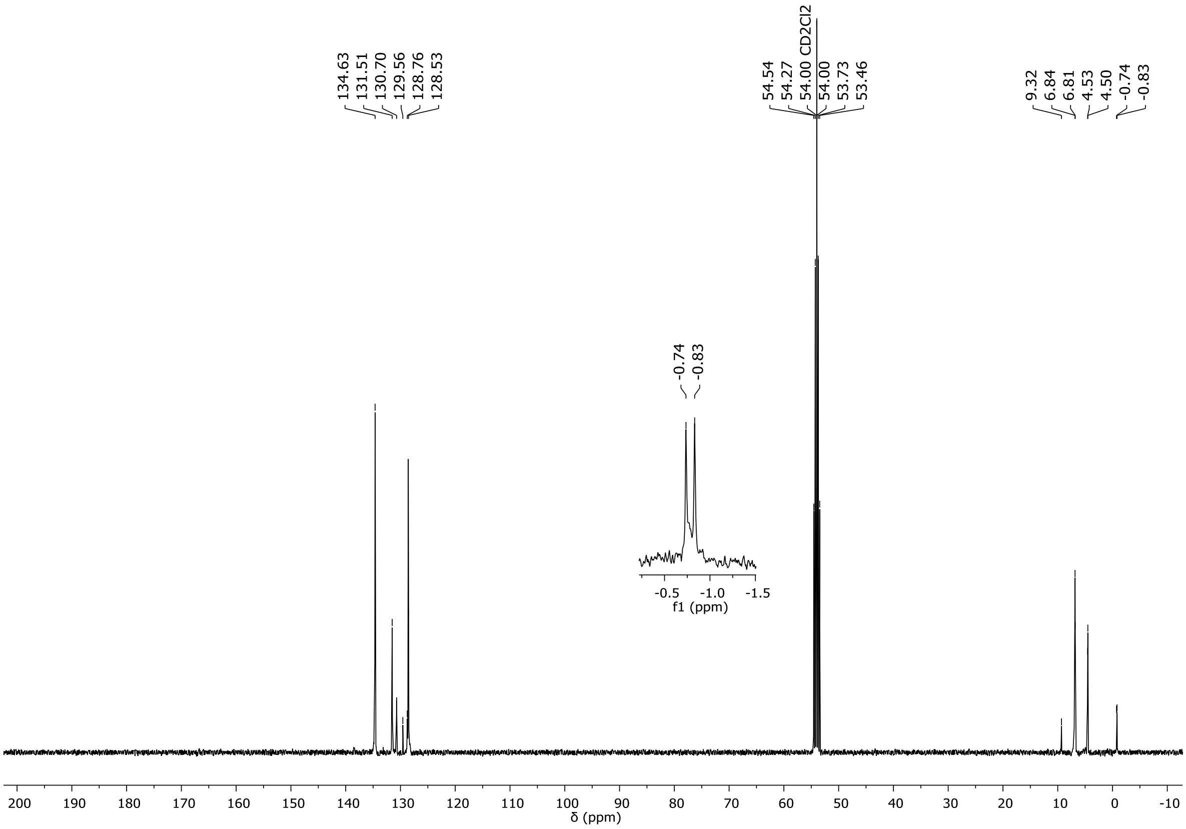

Figure S5 ^{13}C NMR (101 MHz, CD_2Cl_2) spectrum of ***Ph_7T_8-T_8Et_7****.*

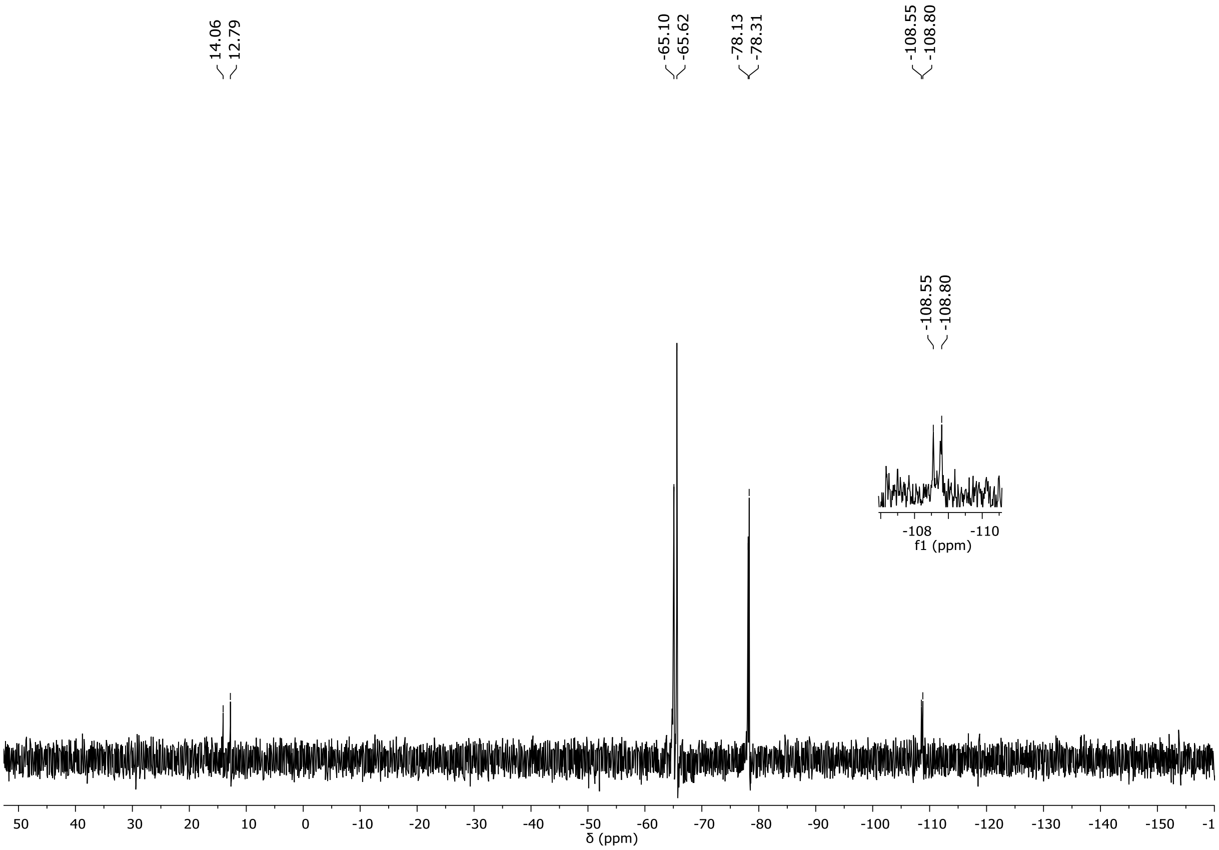

Figure S6 ^{29}Si NMR (79 MHz, CD_2Cl_2) spectrum of ***Ph_7T_8-T_8Et_7****.*

Ph$_7$T$_8$-T$_8$iOc$_7$

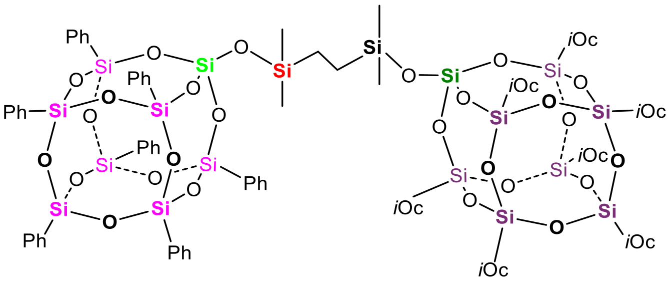

White solid. Isolated Yield 89%

^{1}H NMR (300 MHz, CD_2Cl_2, ppm): δ = 0.00 (s, 6H, $Si(CH_3)_2$), 0.10 (s, 6H, $Si(CH_3)_2$), 0.50-1.27 (m, 112H, $-CH_2-$, $-CH_3$ *i*Oc), 1.82-1.85 (m, 7H, -CH- *i*Oc) 7.35-7.79 (m, 35H, Ph);
^{13}C NMR (101 MHz, CD_2Cl_2, ppm): δ= -0.67 ($Si-(CH_3)_2$), -0.59 ($Si-(CH_3)_2$), 9.32 ($-CH_2-$), 24.04, 24.14, 25.59, 26.12, 26.15, 30.54 31.62, 31.65 (*i*Oc), 128.56, 130.69, 131.51, 134.62 (Ph);
^{29}Si NMR (79 MHz, CD_2Cl_2, ppm): δ = 14.05 (Si^M), 12.29 (Si^M), -67.23, -67.29, -68.09 ($-Si^T$-*i*Oc), -78.16, -78.32, -78.37 ($-Si^T$-Ph), -108.83 (Si^Q), -109.56 (Si^Q);
FT-IR (ATR, cm^{-1}): 3052.78 (C-H phenyl), 2954.10 (=C-H), 2907.09, 2868.67 (C-H), 1594.17 1476.05 (C-H), 1430.70 (C=C phenyl), 1363.93, 1264.09, 1226.67 (Si-C), 1133.37, 1099.11 (Si-O-Si), 998.32 (C-H phenyl).
EA: Anal. calcd for $C_{104}H_{170}O_{26}Si_{18}$ (%): C, 53.34, H, 7.32; found: C, 53.33; H, 7.32.

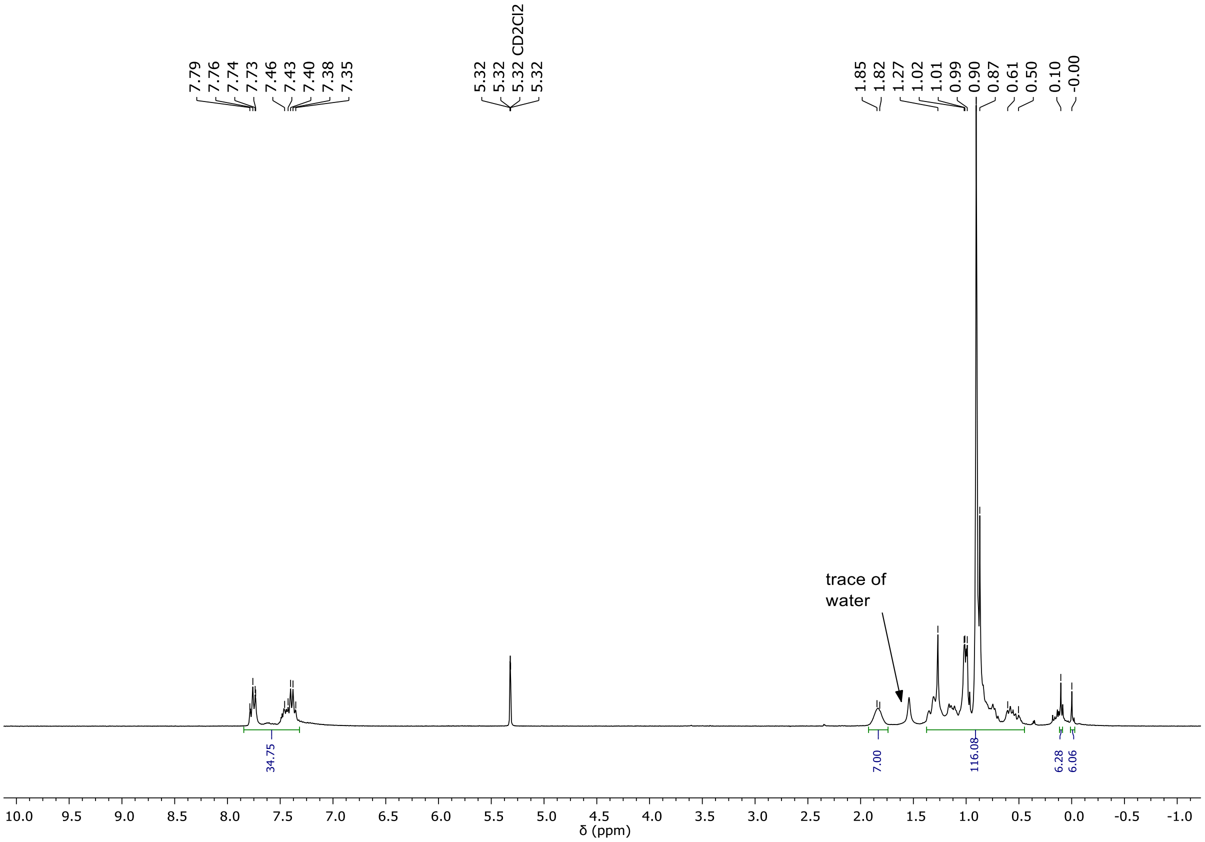

Figure S7 ^{1}H NMR (300 MHz, CD_2Cl_2) spectrum of ***Ph$_7$T$_8$-T$_8$iOc$_7$.***

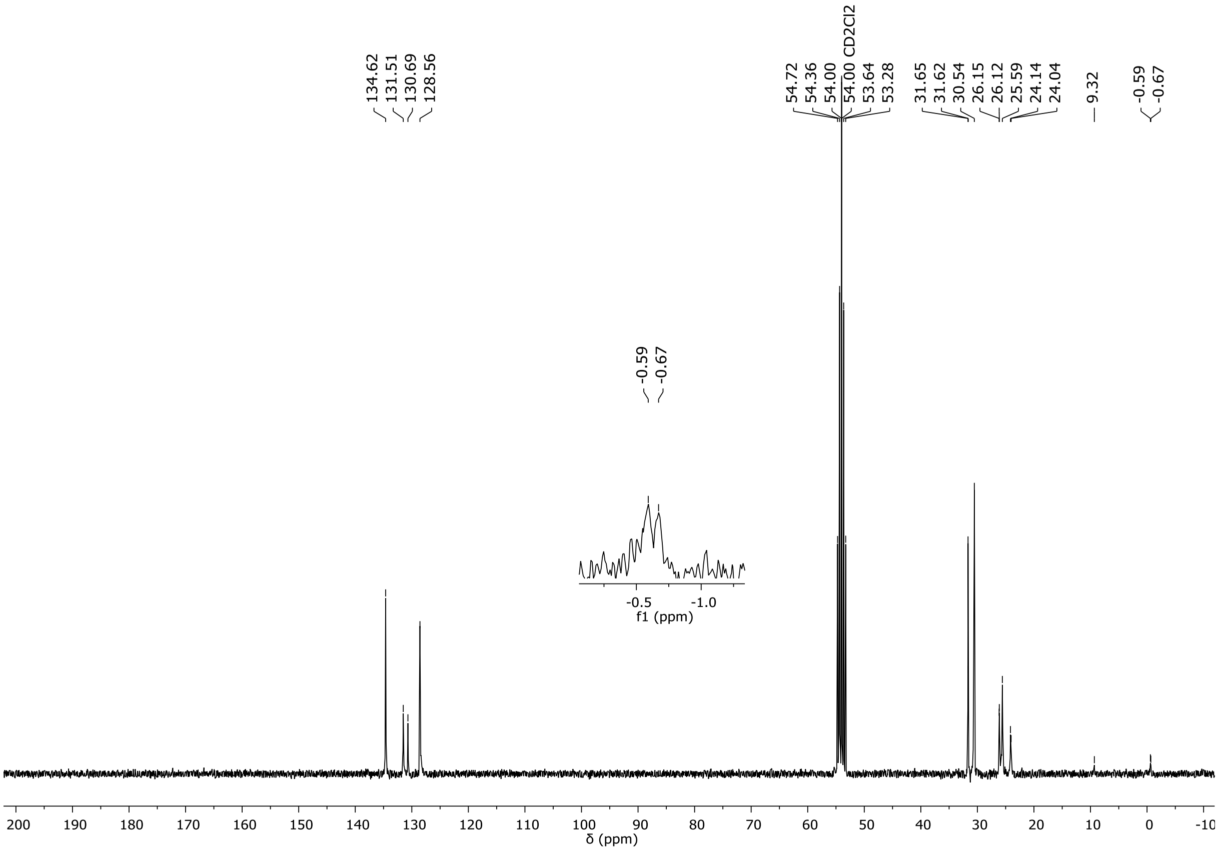

Figure S8 ^{13}*C NMR (101 MHz,* CD_2Cl_2*) spectrum of* **Ph_7T_8-T_8iOc$_7$**.

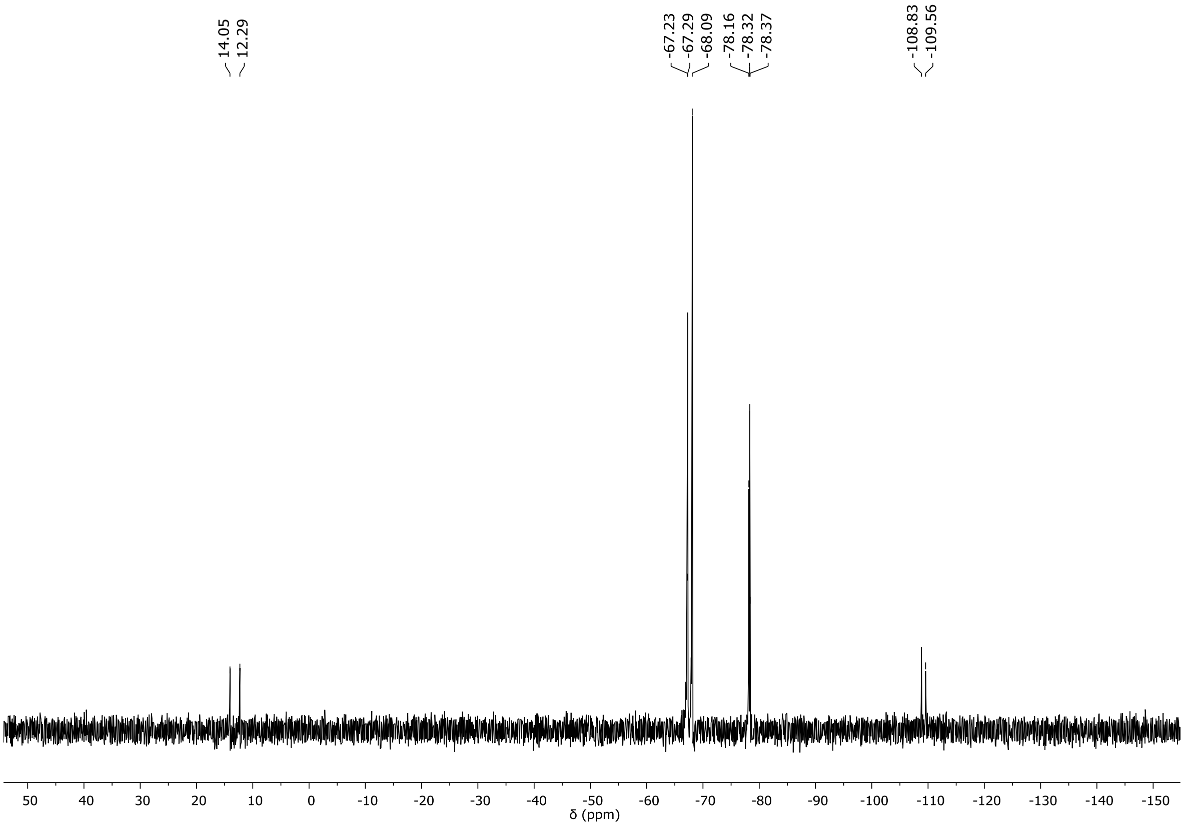

Figure S9 ^{29}*Si NMR (79 MHz,* CD_2Cl_2*) spectrum of* **Ph_7T_8-T_8iOc$_7$**.

iBu$_7$T$_8$-T$_8$Et$_7$

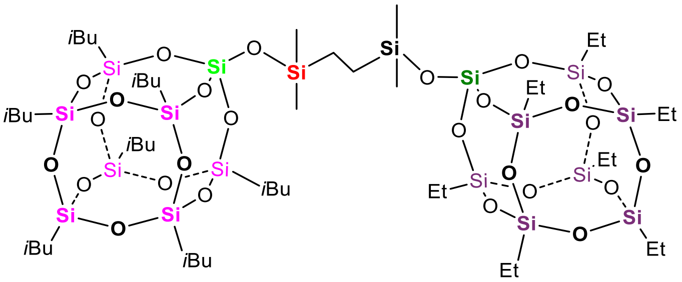

White solid. Isolated Yield 95%

^{1}H NMR (300 MHz, $CDCl_3$, ppm): δ = -0.10 (s, 6H, $Si(CH_3)_2$), 0.12 (s, 6H, $Si(CH_3)_2$), 0.51 (s, 4H, -CH_2-), 0.56-0.64 (m, 28H, -CH_2- *i*Bu, -CH_2- Et), 0.94-1.01 (m, 63H, -CH_3 *i*Bu, -CH_3 Et), 1.81-1.87 (m, 7H, -CH- *i*Bu);

^{13}C NMR (101 MHz, $CDCl_3$, ppm): δ = -0.94 (Si-$(CH_3)_2$), -0.91 (Si-$(CH_3)_2$), 4.14 (-CH_3 Et), 6.65 (-CH_2- Et), 8.97 (-CH_2-), 22.57, 22.62, 24.01, 25.86 (*i*Bu);

^{29}Si NMR (79 MHz, $CDCl_3$, ppm): δ = 12.07 (Si^M), 12.54 (Si^M), -65.20, -65.75 (Si^T-Et), -67.09, -67.88 (Si^T-*i*Bu), -108.83 (Si^Q), -109.66 (Si^Q);

FT-IR (ATR, cm^{-1}): 2955.90, 2930.18 (=C-H), 2820.30, 2880.03 (C-H), 1462.44 (C-H), 1252.65, 1229.01 (Si-C), 1076.55, 1014.20 (Si-O-Si).

EA: Anal. calcd for $C_{48}H_{114}O_{26}Si_{18}$ (%): C, 35.74, H, 7.12; found: C, 35.77; H, 7.16.

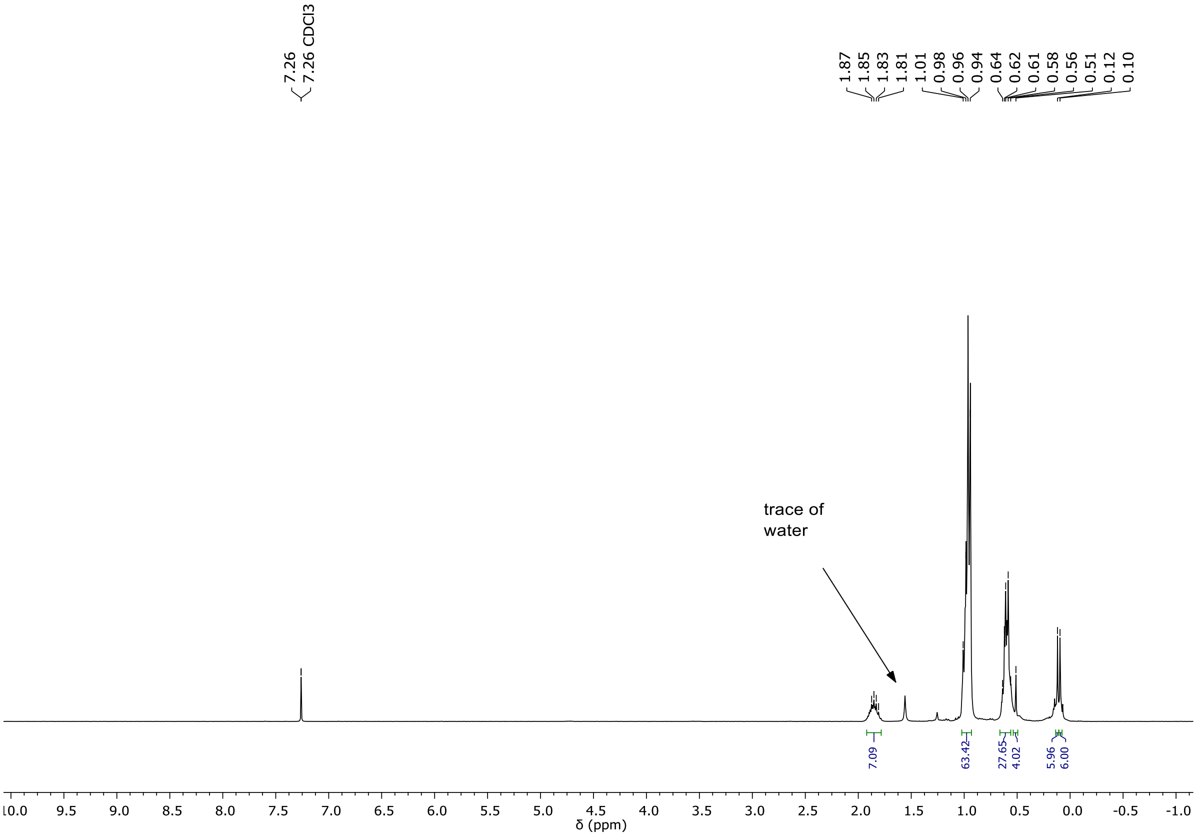

Figure S10 ^{1}H NMR (300 MHz, CD_2Cl_2) spectrum of ***iBu$_7$T$_8$-T$_8$Et$_7$****.*

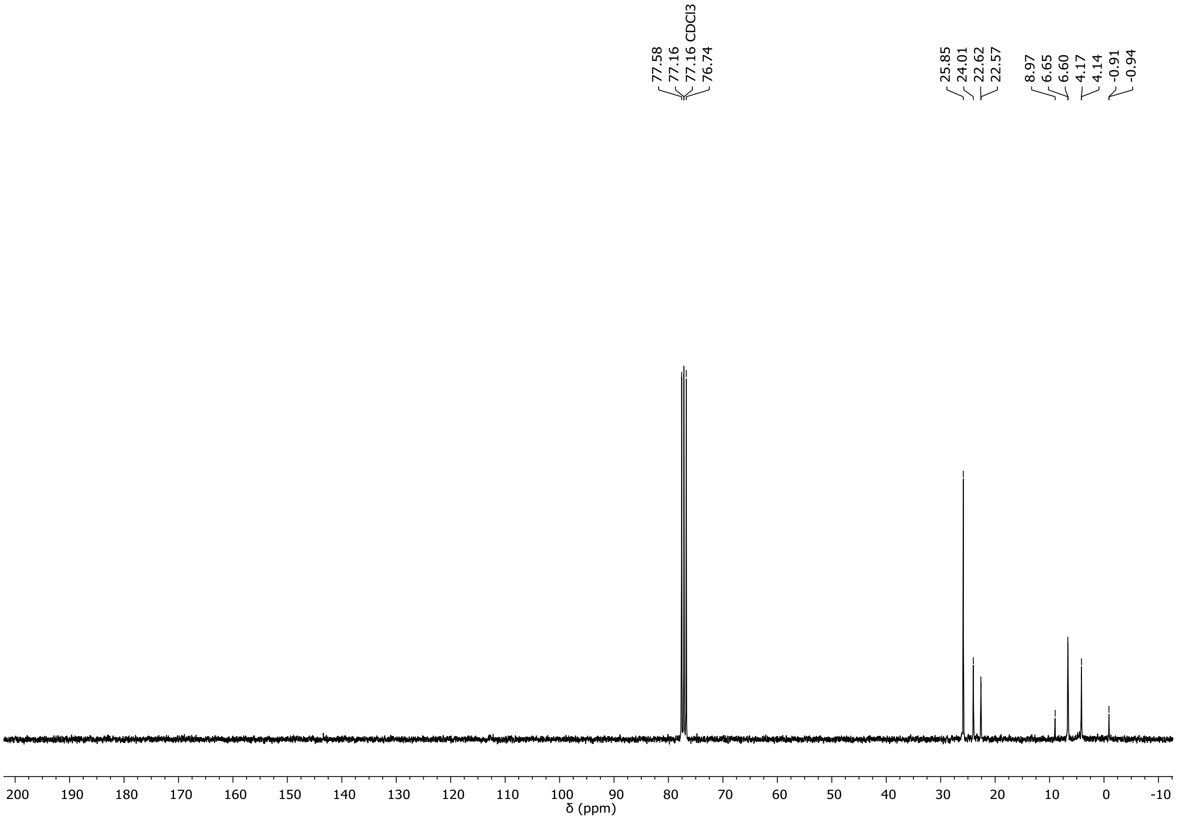

Figure S11 ^{13}C NMR (101 MHz, CD_2Cl_2) spectrum of ***iBu$_7$T$_8$-T$_8$Et$_7$.***

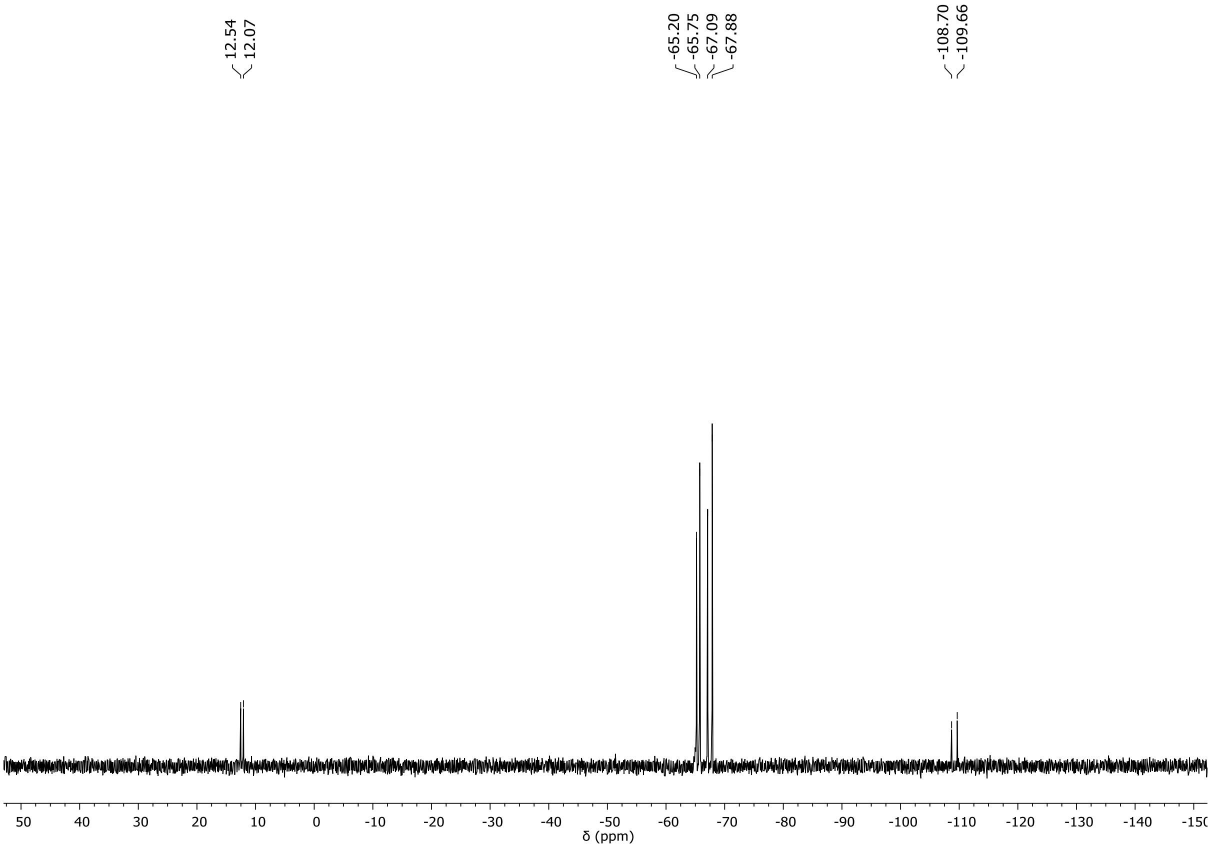

Figure S12 ^{29}Si NMR (79 MHz, CD_2Cl_2) spectrum of ***iBu$_7$T$_8$-T$_8$Et$_7$.***

***iBu*$_7$*T*$_8$*-T*$_8$*iOc*$_7$**

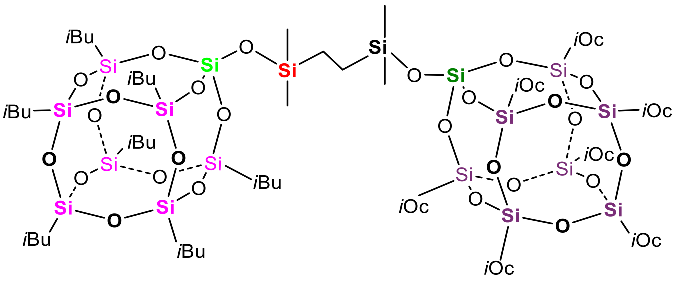

White solid. Isolated Yield 89%

^{1}H NMR (300 MHz, $CDCl_3$, ppm): δ = -0.10 (s, 6H, $Si(CH_3)_2$), 0.10 (s, 6H, $Si(CH_3)_2$), 0.59-0.62 (m, 14H, $-CH_2$, *i*Oc, 14H $-CH_2-$ *i*Bu, 4H $-CH_2-$), 0.89-1.34 (m, 42H, $-CH_3$ *i*Bu, 98H, $-CH_3$ *i*Oc), 1.81-1.90 (m, 7H, -CH *i*Oc);
^{13}C NMR (101 MHz, $CDCl_3$, ppm): δ = -0.79 (Si-$(CH_3)_2$), -0.79 (Si-$(CH_3)_2$), 8.99 ($-CH_2-$), 22.60, 22.68 (*i*Bu), 23.68, 23.78, 23.99 (*i*Oc), 24.03 (*i*Bu), 25.11, 25.17 (*i*Oc), 25.89 (*i*Bu), 30.32, 31.33, 54.12 (*i*Oc);
^{29}Si NMR (79 MHz, $CDCl_3$, ppm): δ = 12.12 (Si^M), 12.10 (Si^M), -67.10, -67.41 (Si^T-*i*Bu), -67.89, -68.21 (Si^T-iOc), -109.62 (Si^Q), -109.72 (Si^Q);
FT-IR (ATR, cm^{-1}): 2952.34, 2906.17, 2868.88 (C-H), 1465.89 (C-H), 1250.93, 1227.68 (Si-C), 1088.64 (Si-O-Si),
EA: Anal. calcd for $C_{90}H_{198}O_{26}Si_{18}$ (%): C, 49.09, H, 9.06; found: C, 49.05; H, 9.07.

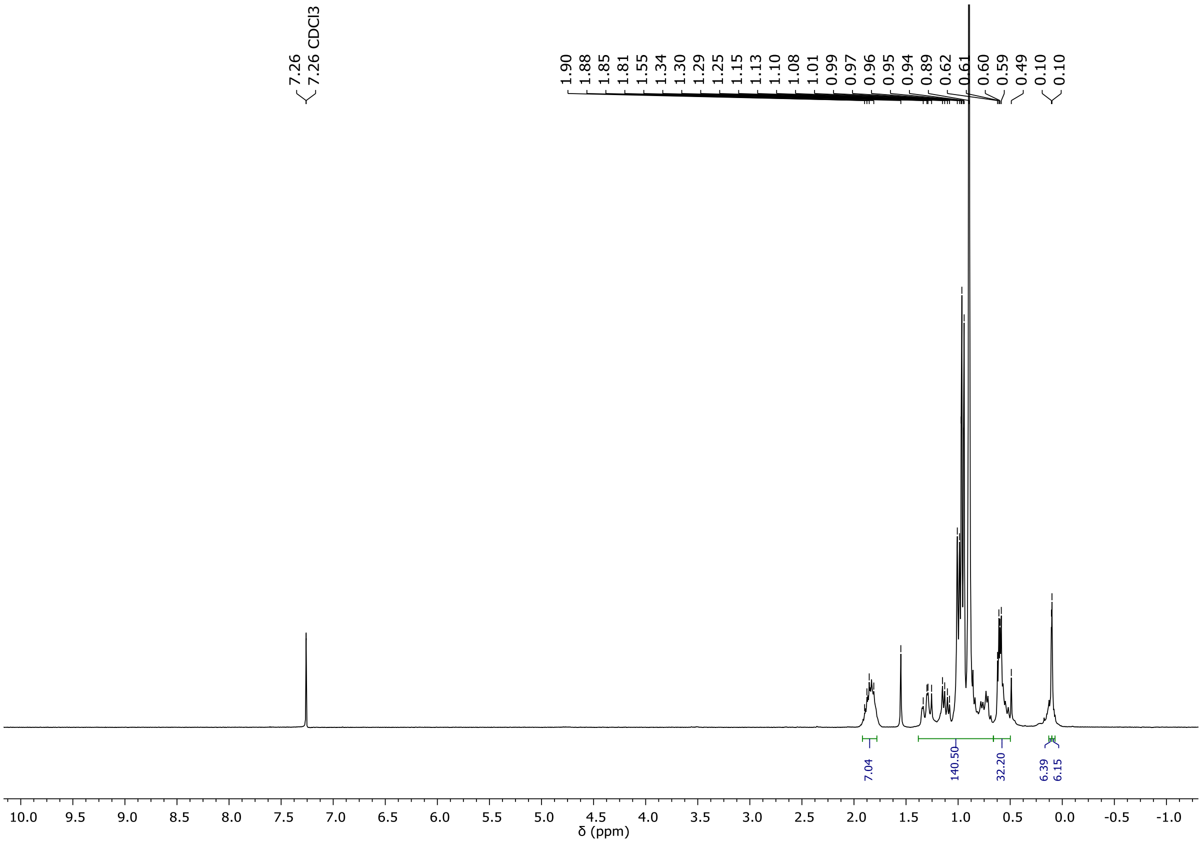

Figure S13 ^{1}H NMR (300 MHz, CD_2Cl_2) spectrum of ***iBu*$_7$*T*$_8$*-T*$_8$*iOc*$_7$***.

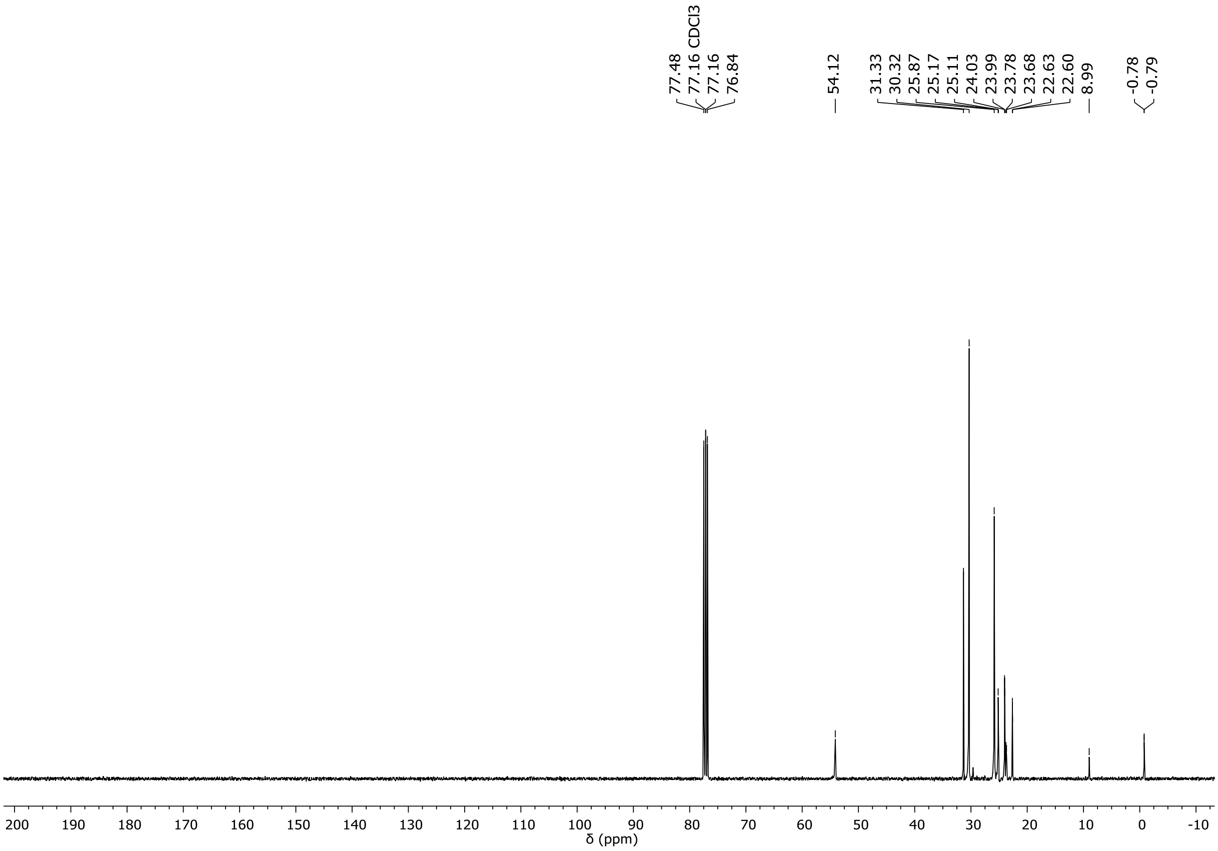

Figure S14 ^{13}C NMR (101 MHz, CD_2Cl_2) spectrum of ***iBu$_7$T$_8$-T$_8$iOc$_7$.***

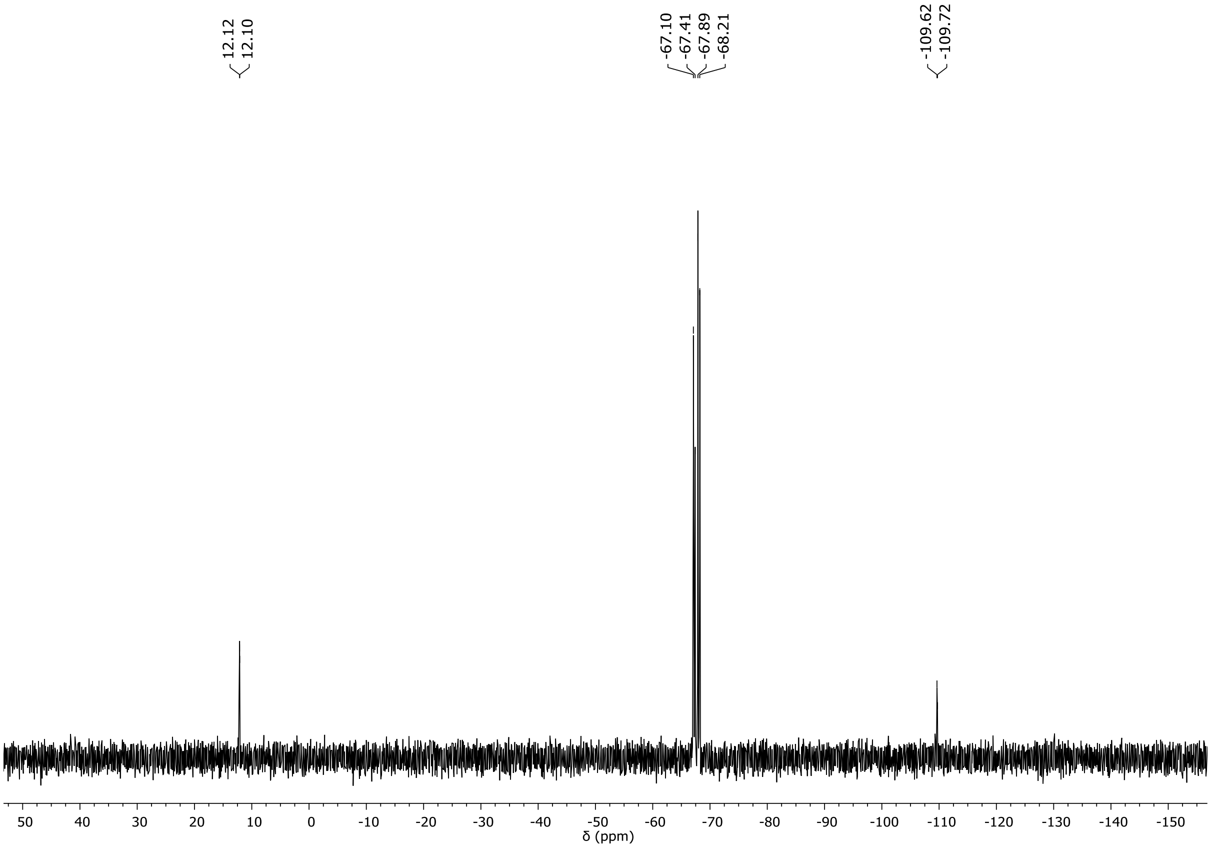

Figure S15 ^{29}Si NMR (79 MHz, CD_2Cl_2) spectrum of ***iBu$_7$T$_8$-T$_8$iOc$_7$.***

DDSQ-2Si-(T_8Ph_7)$_2$

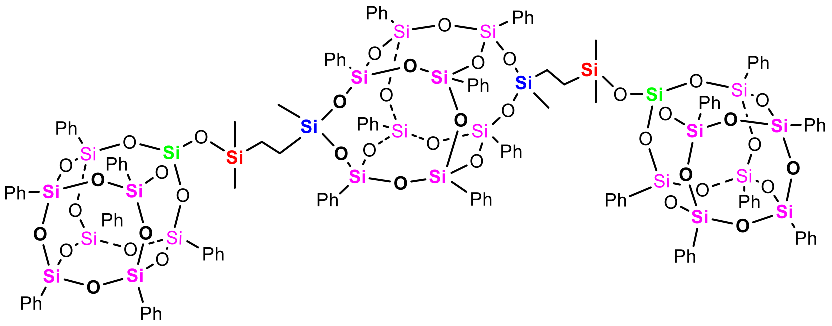

White solid. Isolated Yield 95%

^{1}H NMR (300 MHz, CD_2Cl_2, ppm): δ = -0.04 (s, 12H, $Si(CH_3)_2$), 0.21 (s, 6H, $Si(CH_3)$, 0.61-0.68 (m, 8H, $-CH_2-$), 7.17-7.77 (m, 110H, Ph);
^{13}C NMR (101 MHz, CD_2Cl_2, ppm): δ = -1.39 ($Si-CH_3$), 0.80 ($Si-CH_3$), 8.40 ($-CH_2-$), 9.02 ($-CH_2-$), 128.28-128.40, 130.59-131.50, 132.50, 134.44-134.65 (Ph);
^{29}Si NMR (79 MHz, CD_2Cl_2, ppm): δ = 13.95 (Si^M), -16.86 (Si^D), -78.12, -78.30, -78.35, -79.41, -79.60 (Si^T-Ph), -108.80 (Si^Q);
FT-IR (ATR, cm^{-1}): 3072.87, 3050.71 (C-H phenyl), 2956.91, 2922.36 (C-H), 1594.02, 1430.38 (C=C phenyl), 1260.27 (Si-C), 1087.88, 1028.94 (Si-O-Si), 999.02 (C-H phenyl).
EA: Anal. calcd for $C_{142}H_{136}O_{40}Si_{28}$ (%): C, 52.17, H, 4.19; found: C, 52.18; H, 4.21.

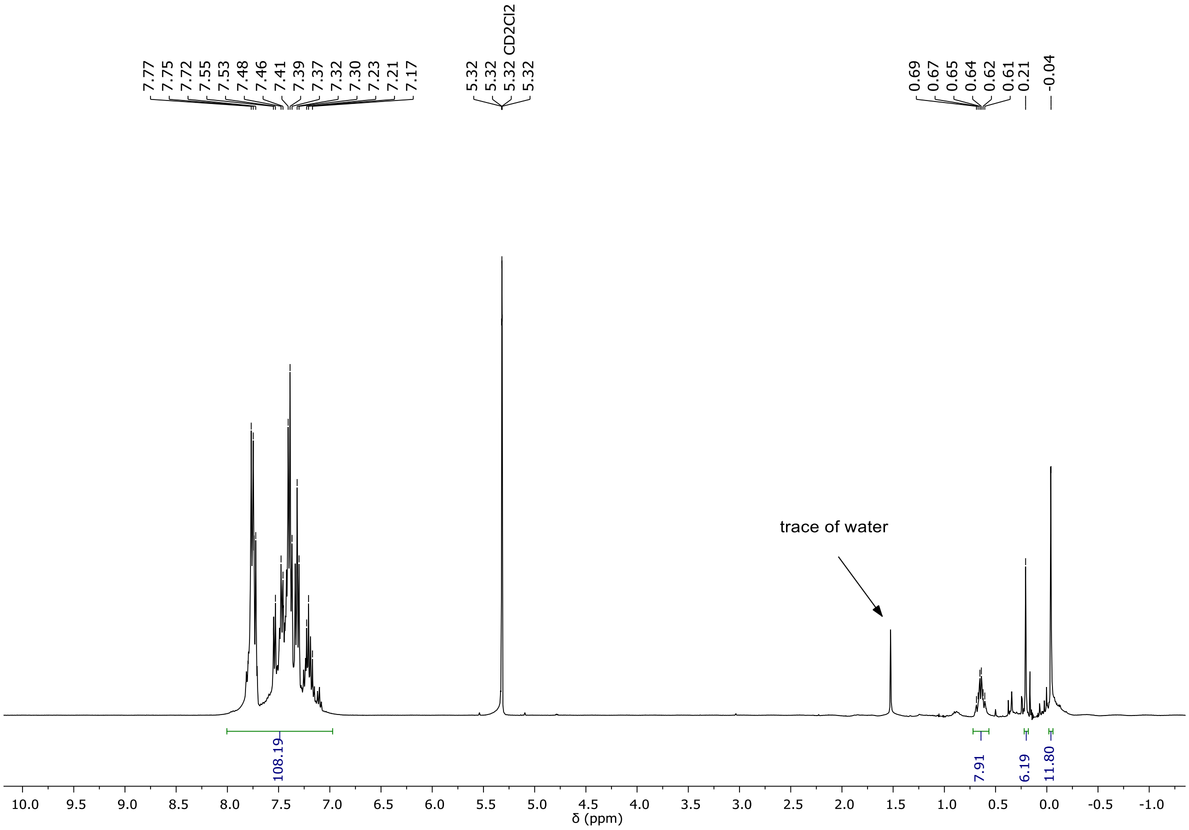

Figure S16 ^{1}H NMR (300 MHz, CD_2Cl_2) spectrum of ***DDSQ-2Si-(T_8Ph_7)$_2$.***

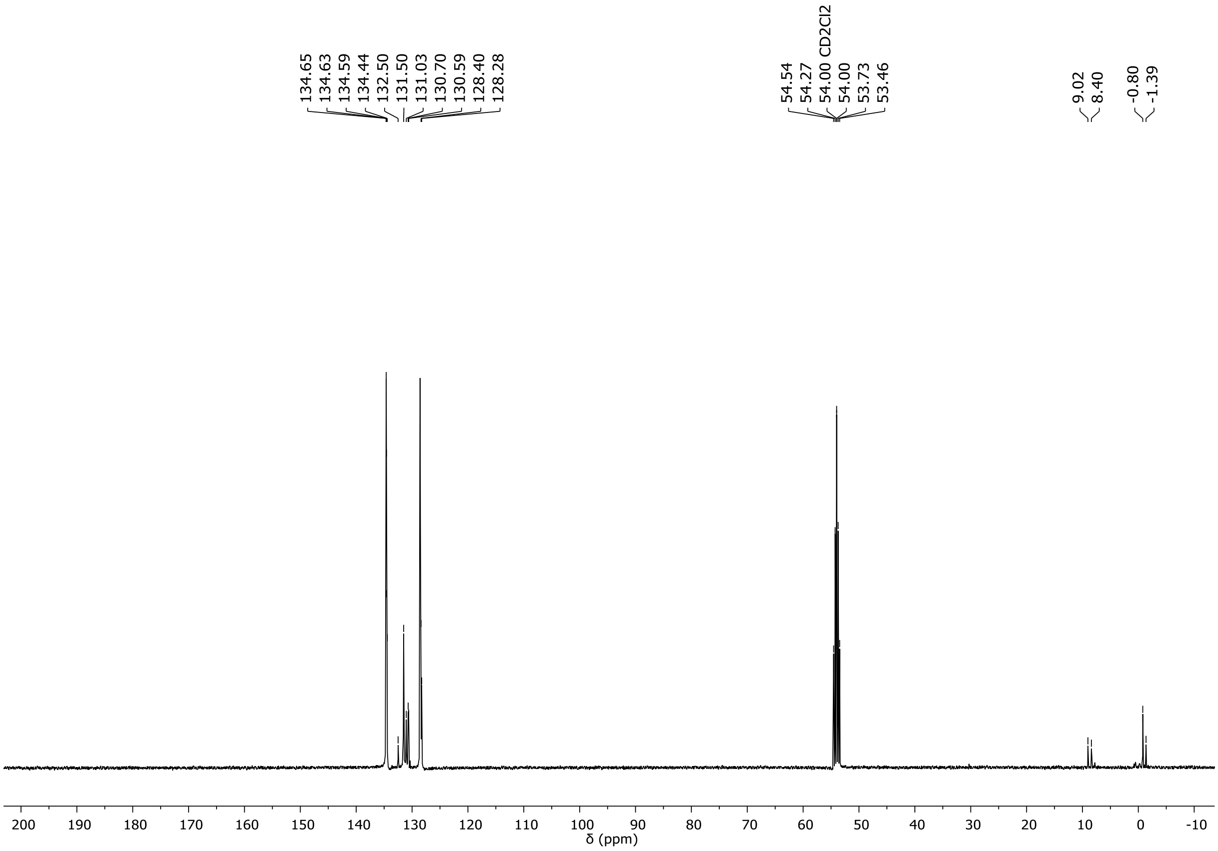

Figure S17 ^{13}C NMR (101 MHz, CD_2Cl_2) spectrum of **DDSQ-2Si-(T_8Ph_7)$_2$.**

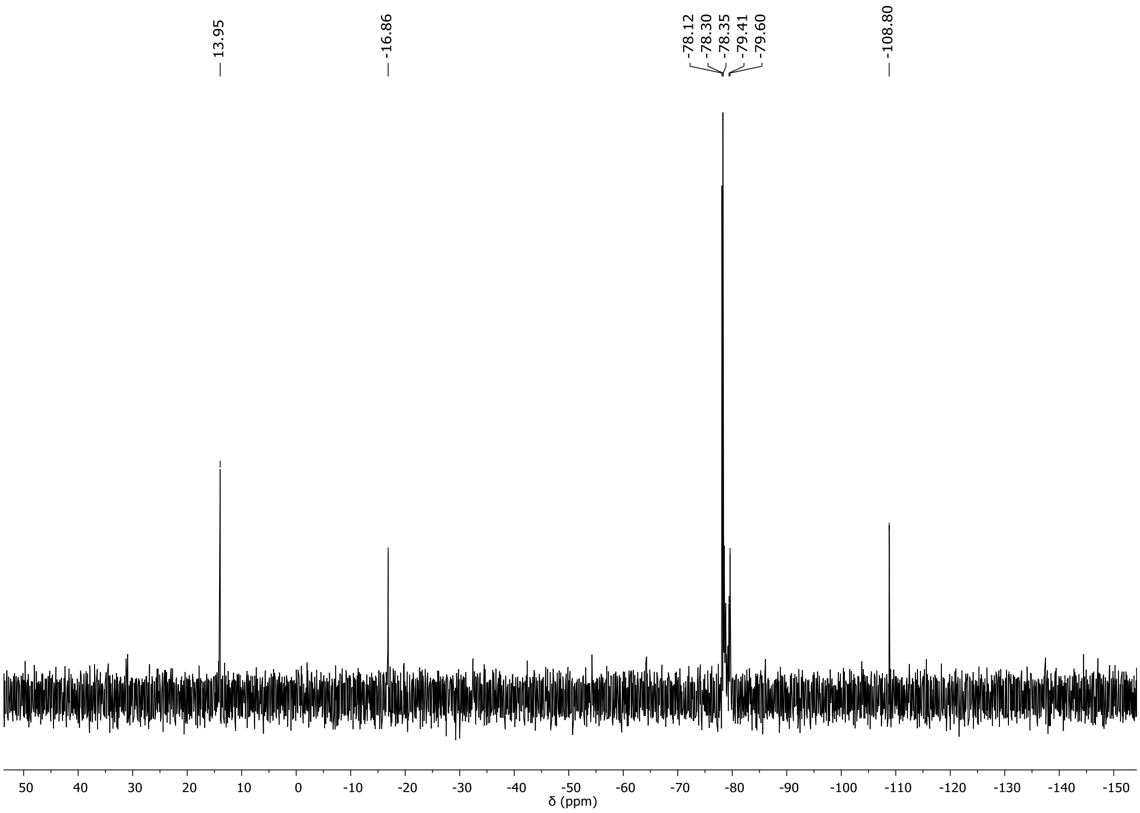

Figure S18 ^{29}Si NMR (79 MHz, CD_2Cl_2) spectrum of **DDSQ-2Si-(T_8Ph_7)$_2$.**

DDSQ-2Si-(T_8iBu$_7$)$_2$

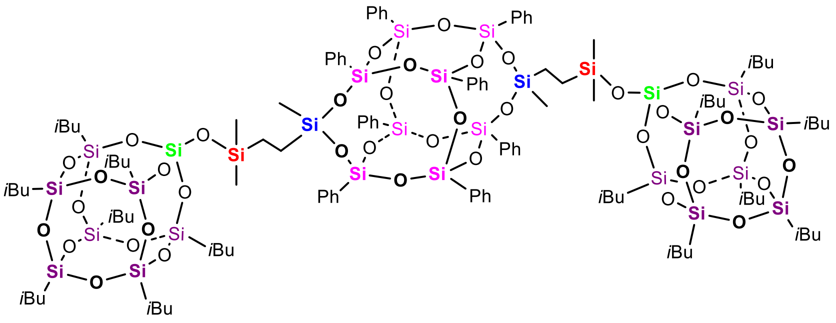

White solid. Isolated Yield 93%

^{1}H NMR (300 MHz, CD_2Cl_2, ppm): δ = -0.04 (s, 12H, $Si(CH_3)_2$), 0.33 (s, 6H, Si-CH_3), 0.54-0.60 (m, 36H, -CH_2-, -CH_2- *i*Bu), 0.87-0.95 (m, 126H, -CH_3 *i*Bu), 1.76-1.89 (m, 14H, -CH- *i*Bu), 7.17-7.57 (m, 40H, Ph);
^{13}C NMR (101 MHz, CD_2Cl_2, ppm): δ = -1.35 (Si-CH_3), -0.87 (Si-CH_3), 8.42 (-CH_2-), 9.02 (-CH_2-), 22.81, 22.90, 24.43, 26.02 (*i*Bu), 128.23, 128.38, 130.98-132.52, 134.43-134.49 (Ph);
^{29}Si NMR (79 MHz, CD_2Cl_2, ppm): δ = 12.20 (Si^M), -16.88 (Si^D), -66.99, -67.03 -67.81 (Si^T-iBu), -78.60, -79.66 (Si^T-Ph), -109.50 (Si^Q);
FT-IR (ATR, cm^{-1}): 3073.24, 3051.82 (C-H phenyl), 2952.66, 2906.01 (C-H), 1464.42 (C-H), 1430.32 (C=C phenyl), 1228.50 (Si-C), 1072.50 (Si-O-Si), 998.43 (C-H phenyl).
EA: Anal. calcd for $C_{114}H_{192}O_{40}Si_{28}$ (%): C, 45.81, H, 6.47; found: C, 45.85; H, 6.52.

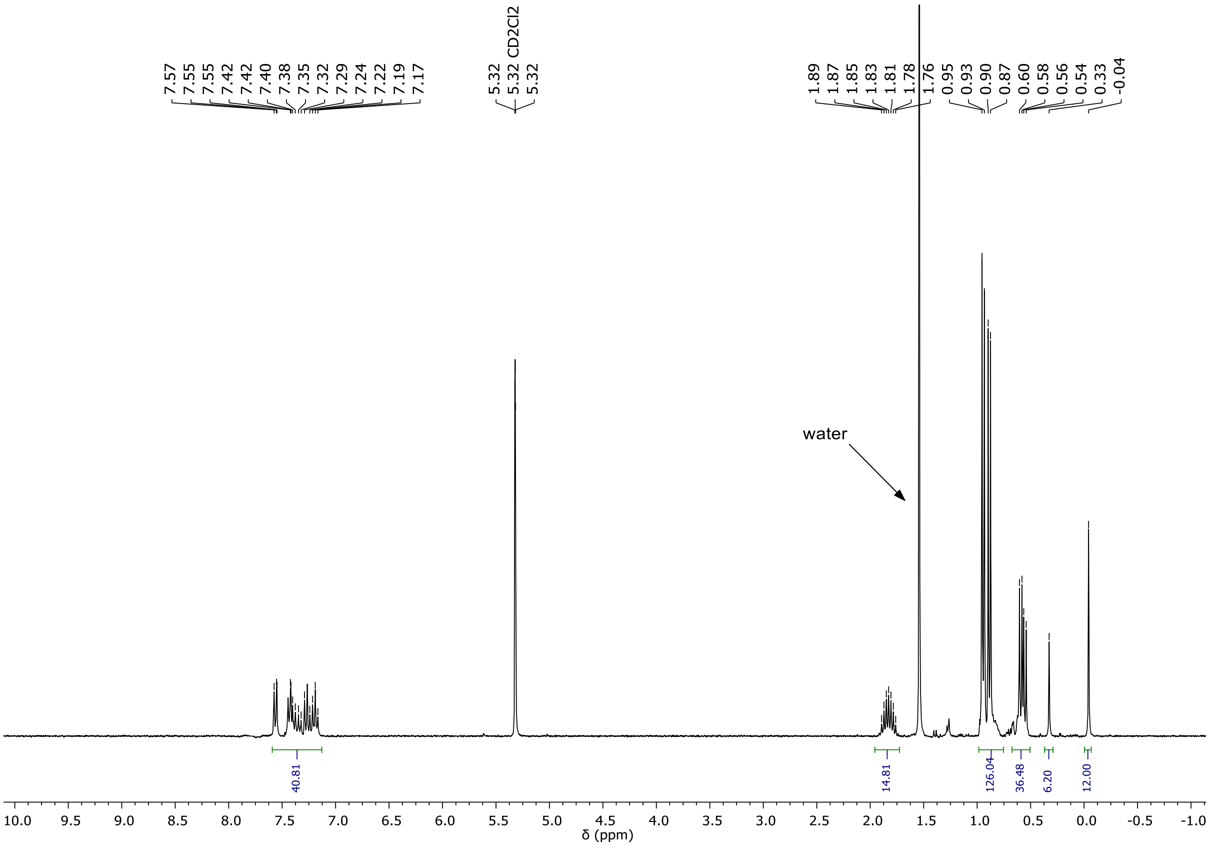

*Figure S19 ^{1}H NMR (300 MHz, CD_2Cl_2) spectrum of **DDSQ-2Si-(T_8iBu$_7$)$_2$**.*

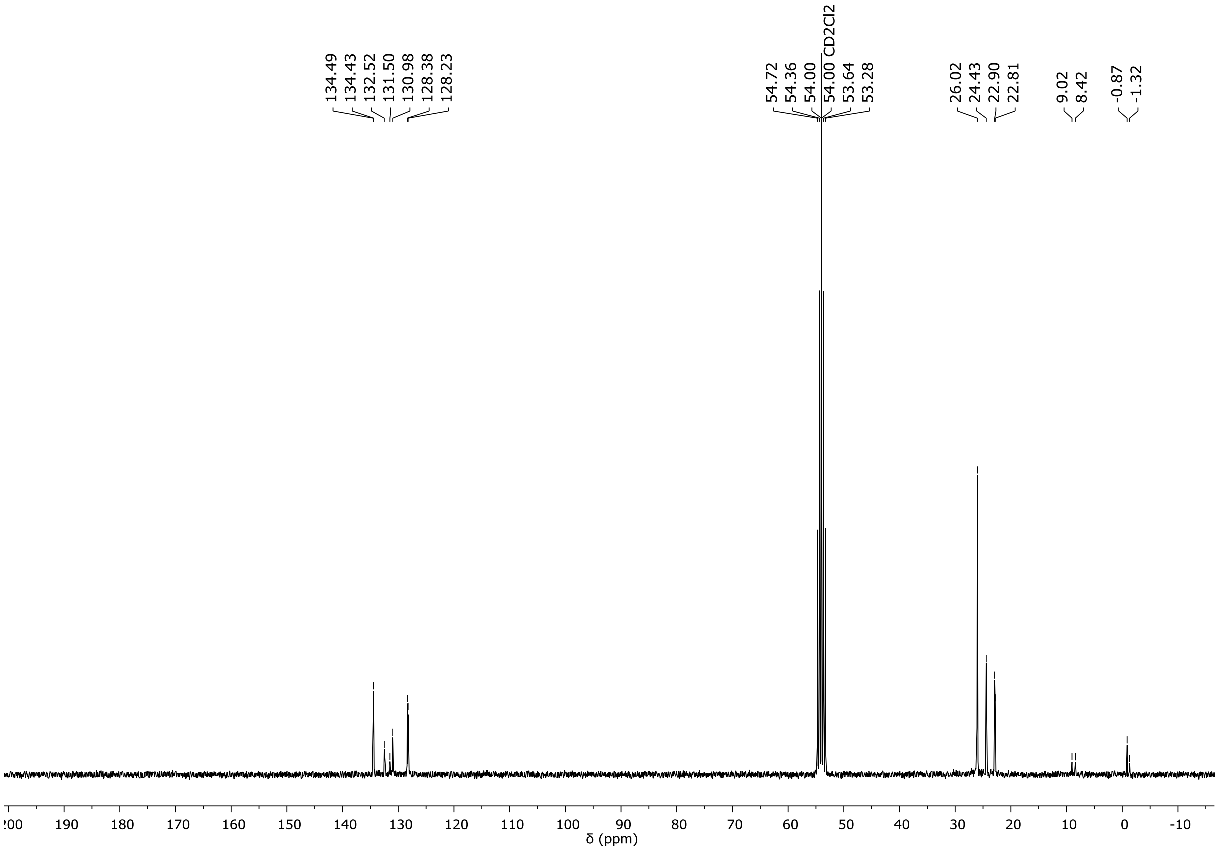

Figure S20 ^{13}C NMR (101 MHz, CD_2Cl_2) spectrum of ***DDSQ-2Si-(T_8iBu$_7$)$_2$.***

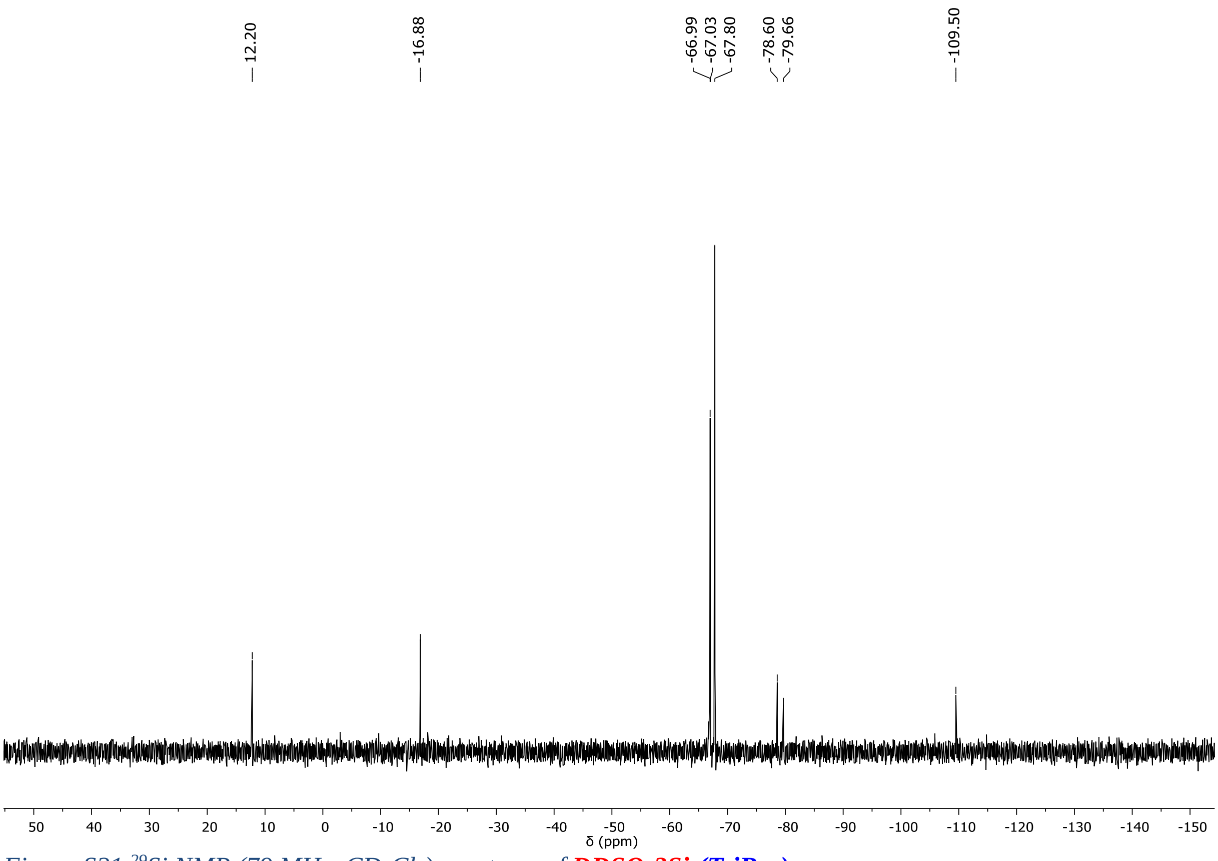

Figure S21 ^{29}Si NMR (79 MHz, CD_2Cl_2) spectrum of ***DDSQ-2Si-(T_8iBu$_7$)$_2$.***

DDSQ-2Si-(T₈Et₇)₂

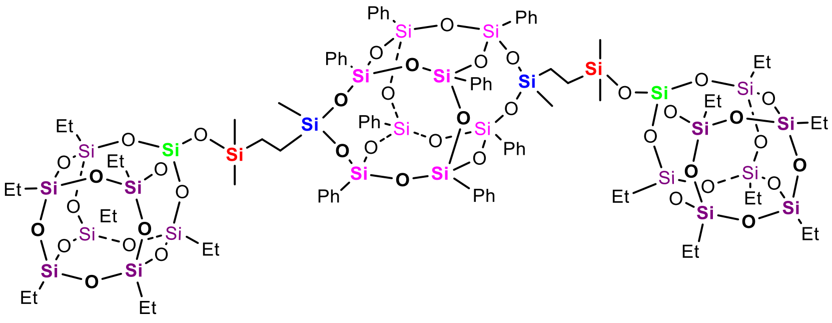

White solid. Isolated Yield 90%

^{1}H NMR (300 MHz, CD_2Cl_2, ppm): δ = -0.02 (s, 12H, $Si(CH_3)_2$), 0.34 (s, 6H, Si-CH_3), 0.53-0.61 (m, 36H, -CH_2-, -CH_2- Et), 0.90-0.99 (m, 42H, -CH_3 Et), 7.20-7.59 (m, 40H, Ph);
^{13}C NMR (101 MHz, CD_2Cl_2, ppm): δ = -1.36 (Si-CH_3), -0.87 (Si-$(CH_3)_2$), 1.38 (-CH_3, Et), 4.49 (-CH_2-Et), 6.78 (-CH_2-), 6.84 (-CH_2-), 128.27, 128.40, 131.01, 132.57, 134.47 (Ph);
^{29}Si NMR (79 MHz, CD_2Cl_2, ppm): δ = 12.67 (Si^{M}), -16.87 (Si^{D}), -65.10, -65.65 (Si^{T}-Et), -78.58, -79.62 (Si^{T}-Ph), -108.59 (Si^{Q});
FT-IR (ATR, cm^{-1}): 3073.63, 3052.81 (C-H phenyl), 2962.89, 2918.46, 2879.99 (C-H), 1460.87 (C-H), 1430.24 (C=C phenyl), 1264.00 (Si-C), 1107.23, 1077.89 (Si-O-Si), 1012.27 (C-H phenyl).
EA: Anal. calcd for $C_{86}H_{136}O_{40}Si_{28}$ (%): C, 39.78, H, 5.29; found: C, 39.79; H, 5.31.

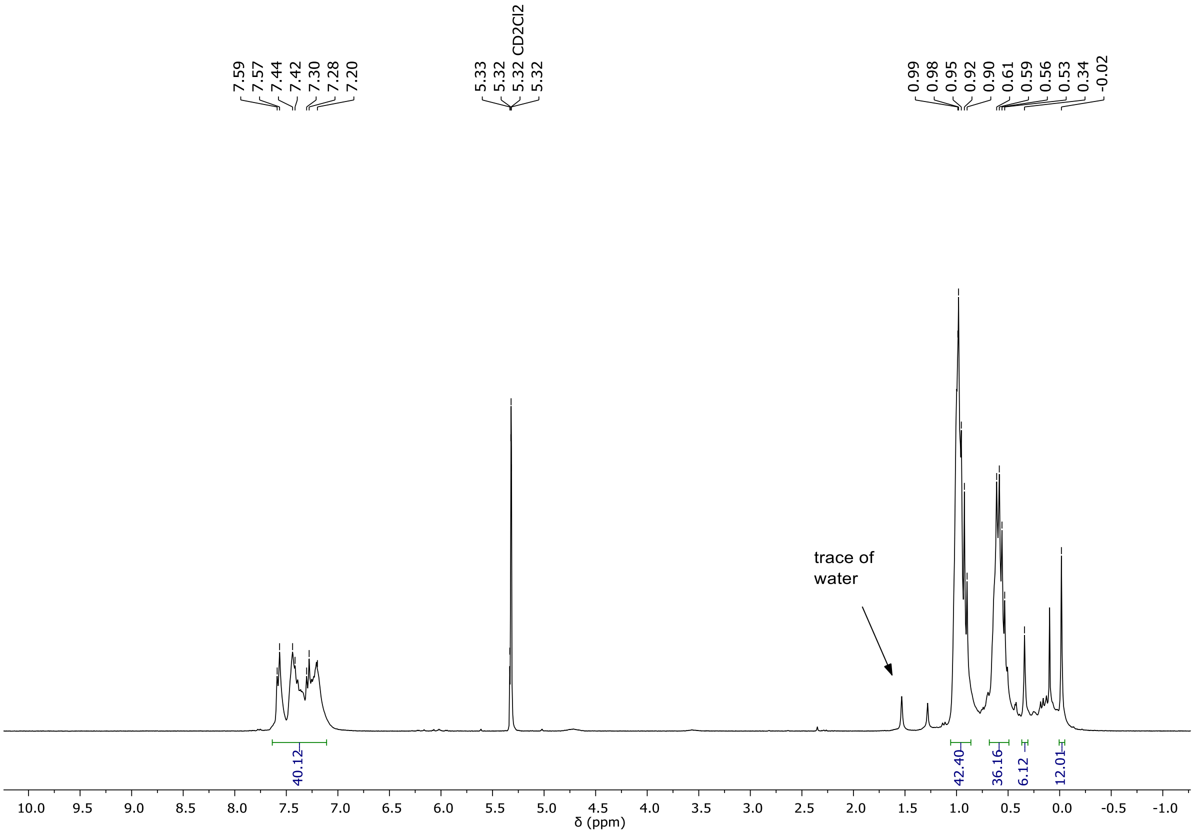

Figure S22 ^{1}H NMR (300 MHz, CD_2Cl_2) spectrum of ***DDSQ-2Si-(T₈Et₇)₂.***

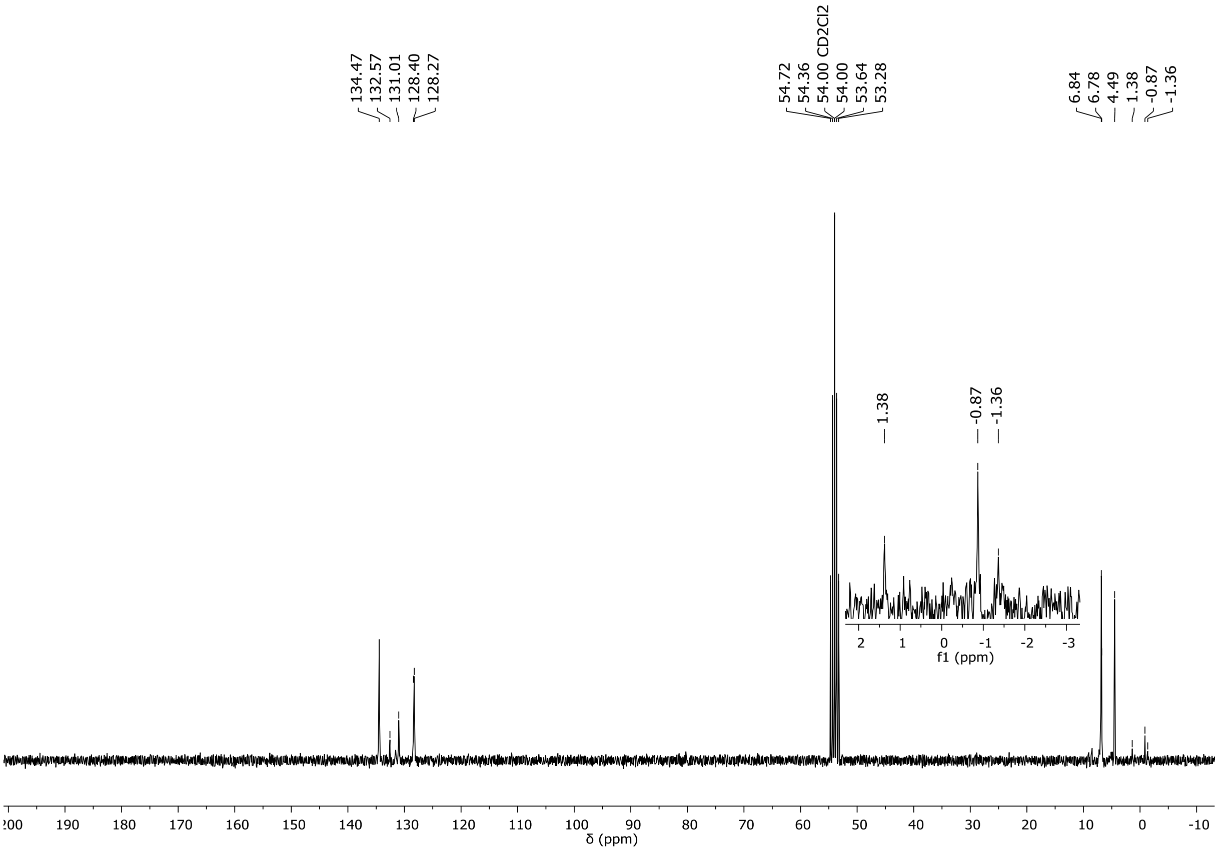

Figure S23 ^{13}C NMR (101 MHz, CD_2Cl_2) spectrum of ***DDSQ-2Si-(T_8Et_7)$_2$.***

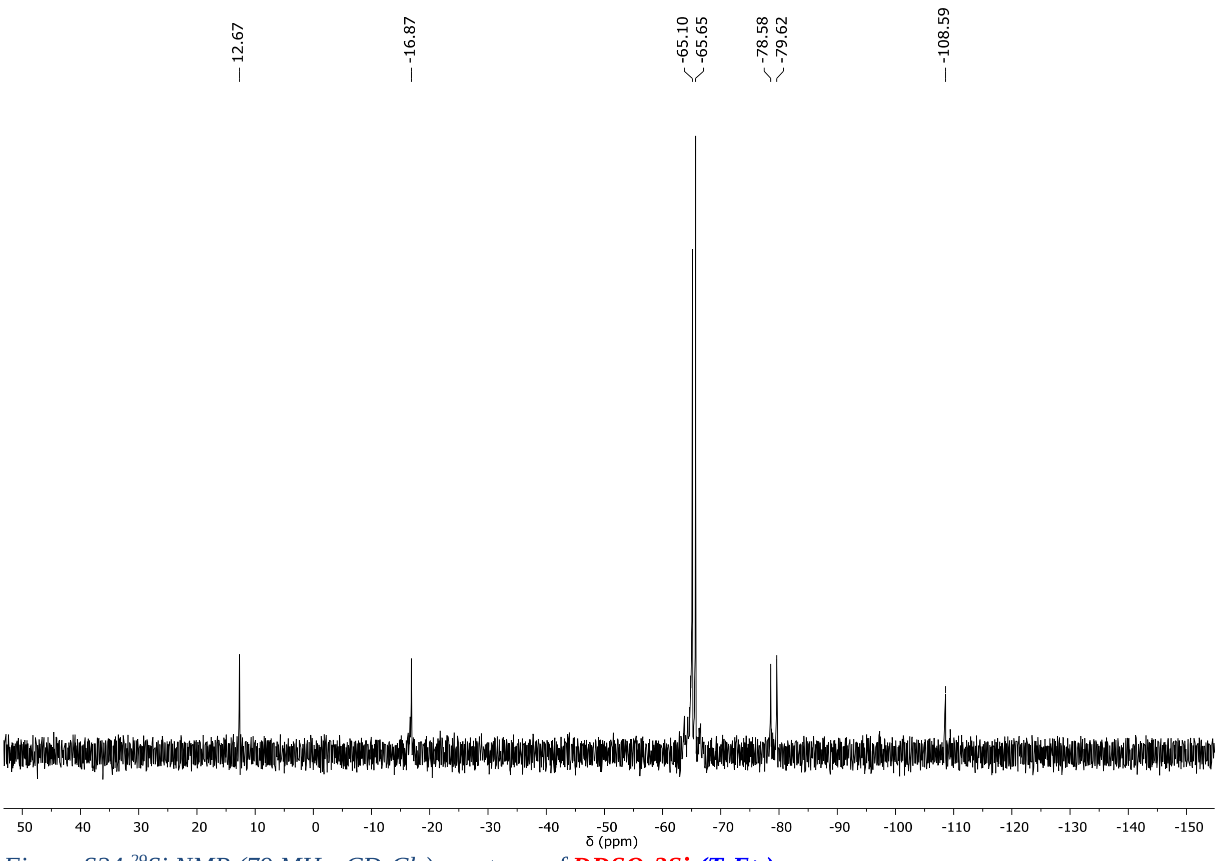

Figure S24 ^{29}Si NMR (79 MHz, CD_2Cl_2) spectrum of ***DDSQ-2Si-(T_8Et_7)$_2$.***

DDSQ-2Si-(T8iOc7)2

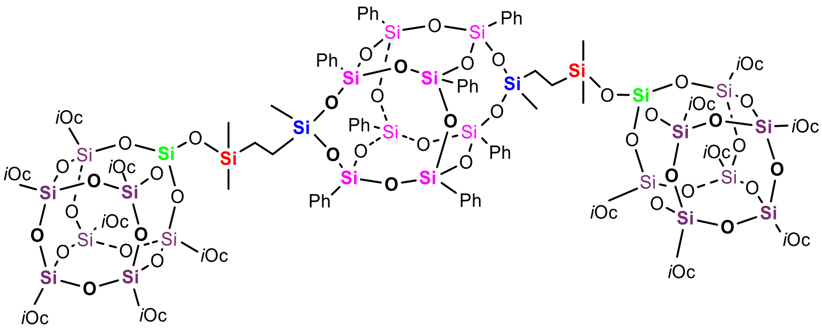

Oil. Isolated Yield 89%

^{1}H NMR (300 MHz, CD_2Cl_2, ppm): δ = -0.33 (s, 12H, $Si(CH_3)_2$), 0.34 (s, 6H, Si-CH_3), 0.53-1.31 (m, 8H, -CH_2-, 224 -CH_2, -CH_3 *i*Oc) ,1.83-1.84 (m, 14H, -CH *i*Oc) 7.20-7.80 (m, 40H, Ph);
^{13}C NMR (101 MHz, CD_2Cl_2, ppm): δ = -1.07 (Si-CH_3), 0.56 (Si-CH_3), 8.65 (-CH_2-), 9.23 (-CH_2-), 24.11, 24.27, 25.61, 25.71, 26.22, 30.66, 31.70, 31.76 (*i*Oc), 128.37, 128.52, 131.11-132.72, 134.59, 134.64 (Ph);
^{29}Si NMR (79 MHz, CD_2Cl_2, ppm): δ = 12.19, 11.31 (Si^M), -16.83, -17.37 (Si^D), -67.27, -68.12 (Si^T-*i*Oc), -78.55, -79.59 (Si^T-Ph), -109.58, -109.89 (Si^Q);
FT-IR (ATR, cm^{-1}): 3073.32 (C-H phenyl), 2951.33, 2905.92, 2868.56 (C-H), 1594.41, 1466.92 (C-H), 1430.23 (C=C phenyl), 1259.21, 1226.16 (Si-C), 1077.15 (Si-O-Si), 998.92 (C-H phenyl).
EA: Anal. calcd for $C_{170}H_{304}O_{40}Si_{28}$ (%): C, 54.09, H, 8.12; found: C, 54.10; H, 8.14.

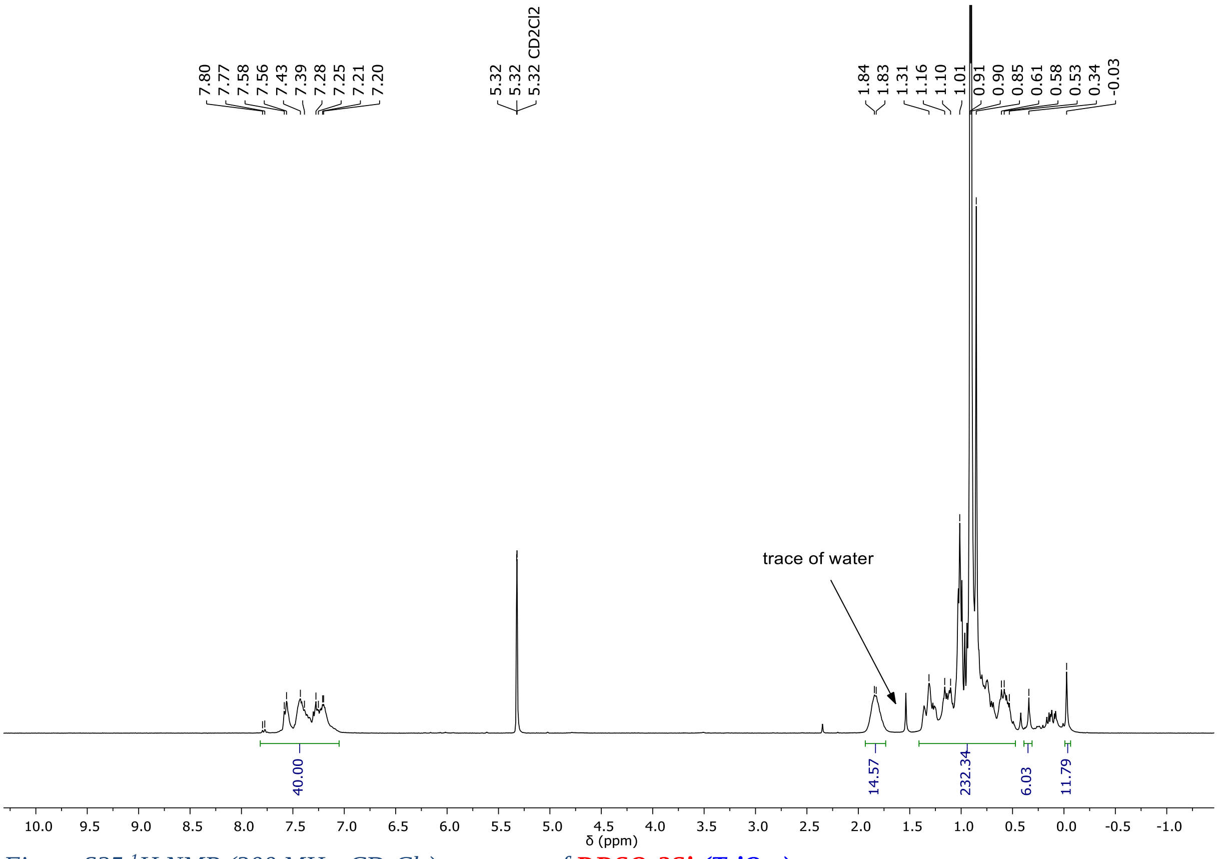

*Figure S25 ^{1}H NMR (300 MHz, CD_2Cl_2) spectrum of **DDSQ-2Si-(T8iOc7)2**.*

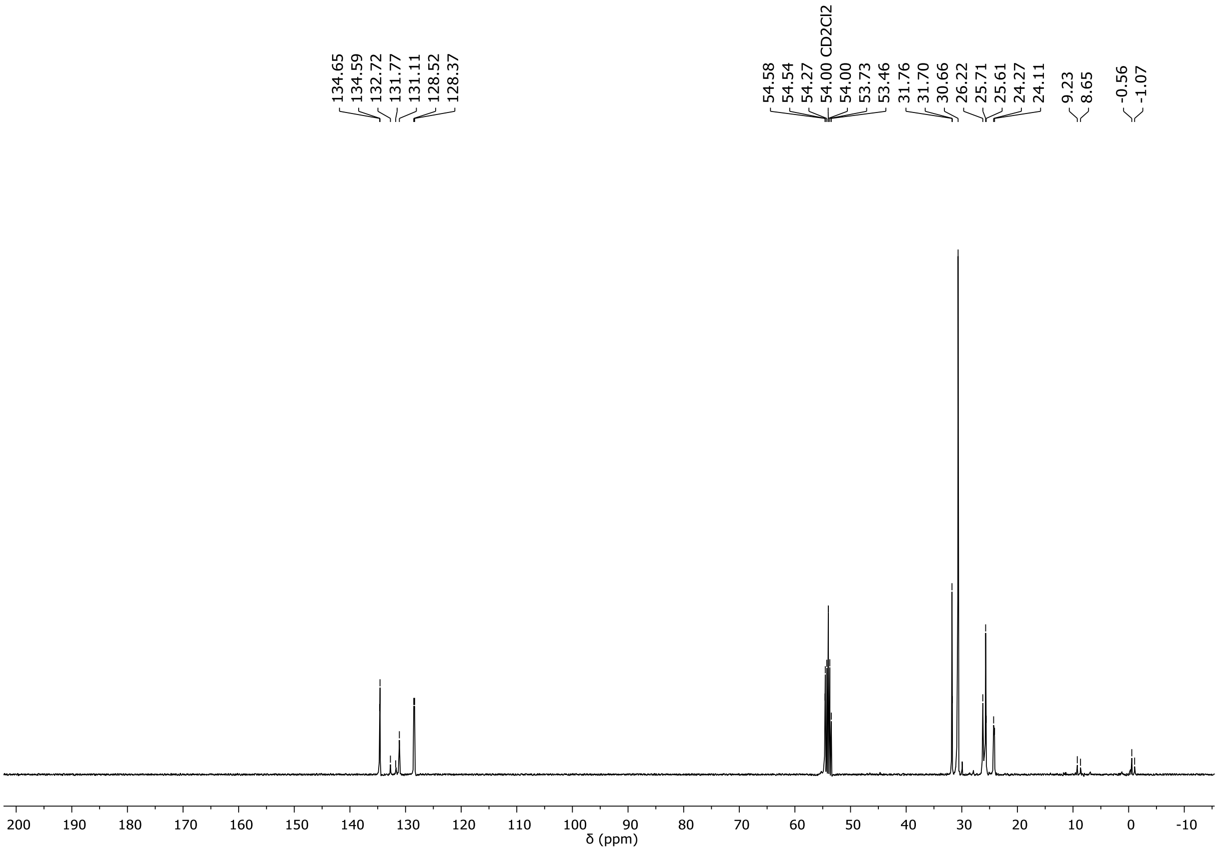

Figure S26 ^{13}C NMR (101 MHz, CD_2Cl_2) spectrum of **DDSQ-2Si-(T$_8$iOc$_7$)$_2$.**

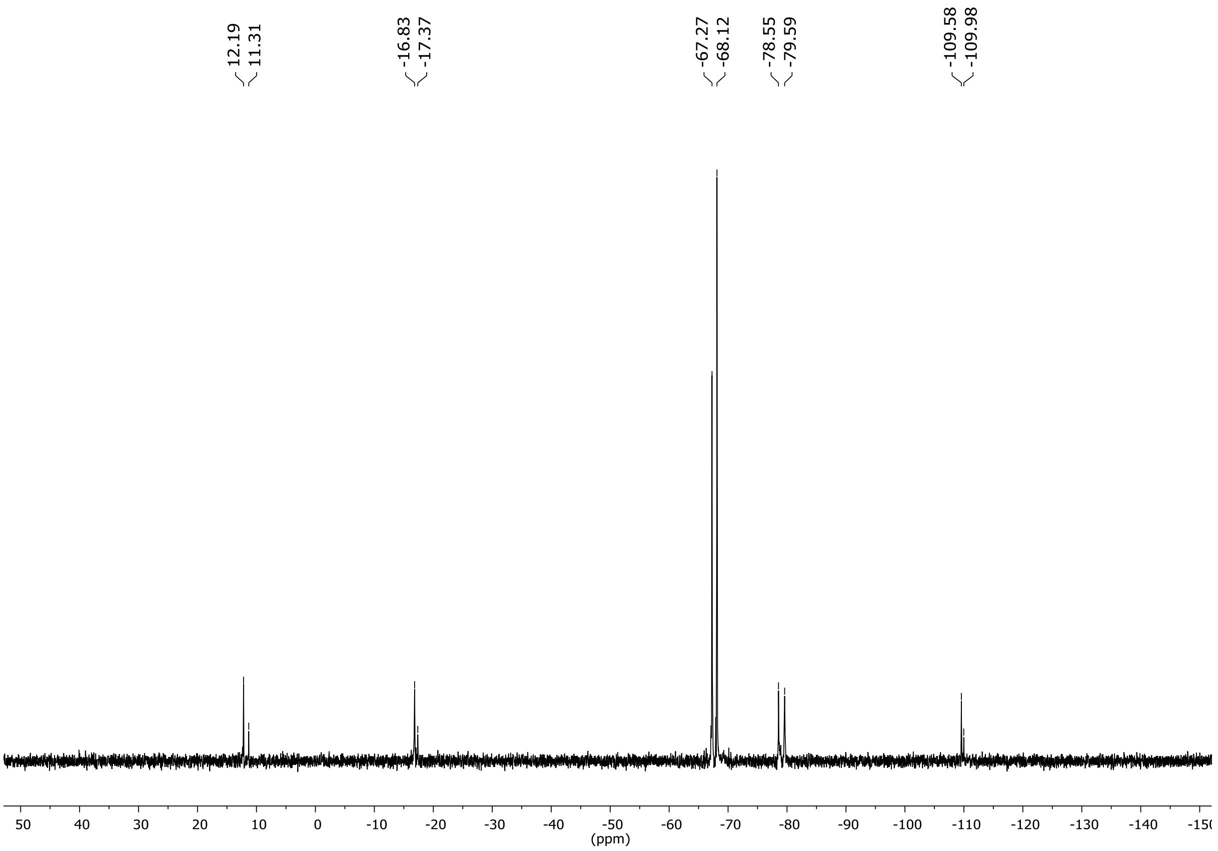

Figure S27 ^{29}Si NMR (79 MHz, CD_2Cl_2) spectrum of **DDSQ-2Si-(T$_8$iOc$_7$)$_2$.**

DDSQ-2OSi-(T_8Ph_7)$_2$

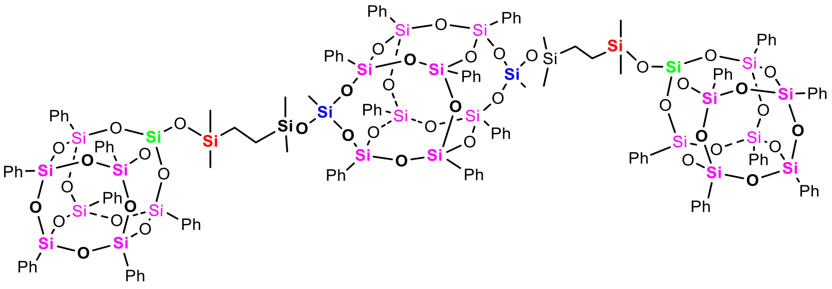

White solid. Isolated Yield 94%

^{1}H NMR (300 MHz CD_2Cl_2, ppm): δ= -0.06 (s, 12H, $Si(CH_3)_2$), -0.04 (s, 12H, $Si(CH_3)_2$), 0.43 (s, 6H, Si-CH_3), 0.85-0.93 (m, 8H, -CH_2-), 7.17-7.76 (m, 110H, Ph);
^{13}C NMR (101 MHz, CD_2Cl_2, ppm): δ = -2.45 (Si-CH_3), -0.84 ($Si(CH_3)_2$), -0.45 ($Si(CH_3)_2$), -9.29 (-CH_2-), 9.42 (-CH_2-), 128.23, 128.56, 129.55 130.68-131.49, 132.31, 134.47-134.60 (Ph);
^{29}Si NMR (79 MHz, CD_2Cl_2, ppm): δ = 14.08 (Si^M), 11.12 (Si^M), -64.10 (Si^T), -78.16, -78.32, -79.20, -79.53 (Si^T-Ph), -108.82 (Si^Q);
FT-IR (ATR, cm^{-1}): 3073.55, 3029.36 (C-H phenyl), 2923.63, 2852.93 (C-H), 1593.92 (C-H), 1430.28 (C=C phenyl), 1264.31 (Si-C), 1089.97 (Si-O-Si), 997.32 (C-H phenyl).
EA: Anal. calcd for $C_{146}H_{148}O_{42}Si_{30}$ (%): C, 51.32, H, 4.37; found: C, 51.34; H, 4.33.

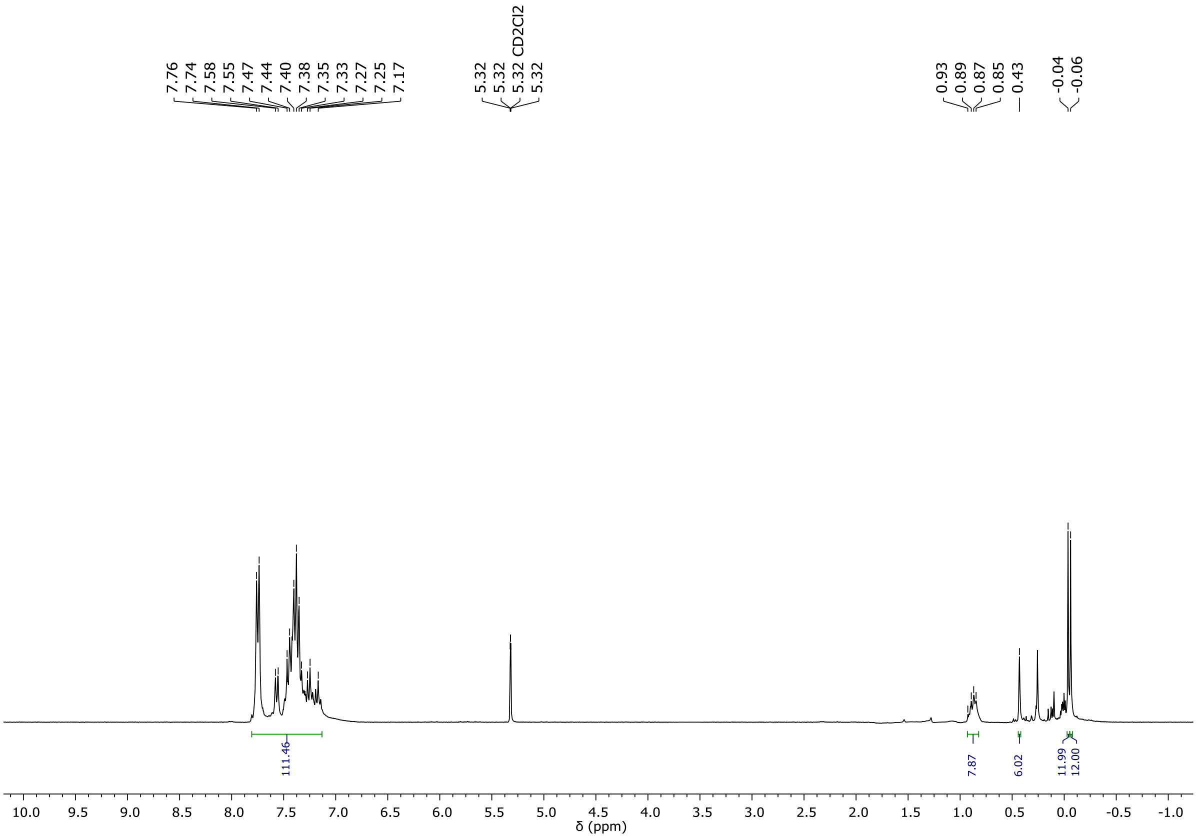

Figure S28 ^{1}H NMR (300 MHz, CD_2Cl_2) spectrum of ***DDSQ-2OSi-(T_8Ph_7)$_2$***.

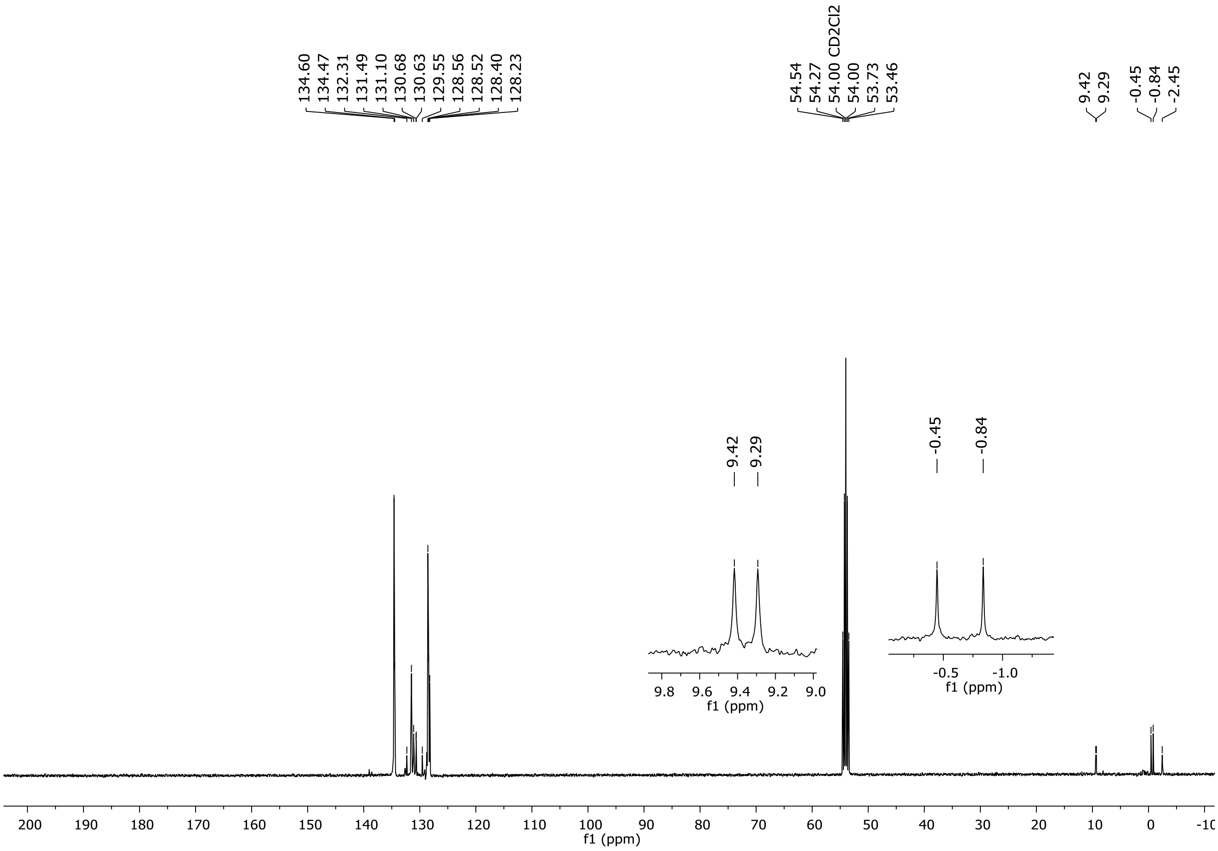

Figure S29 ^{13}C NMR (101 MHz, CD_2Cl_2) spectrum of **DDSQ-2OSi-(T_8Ph_7)$_2$**.

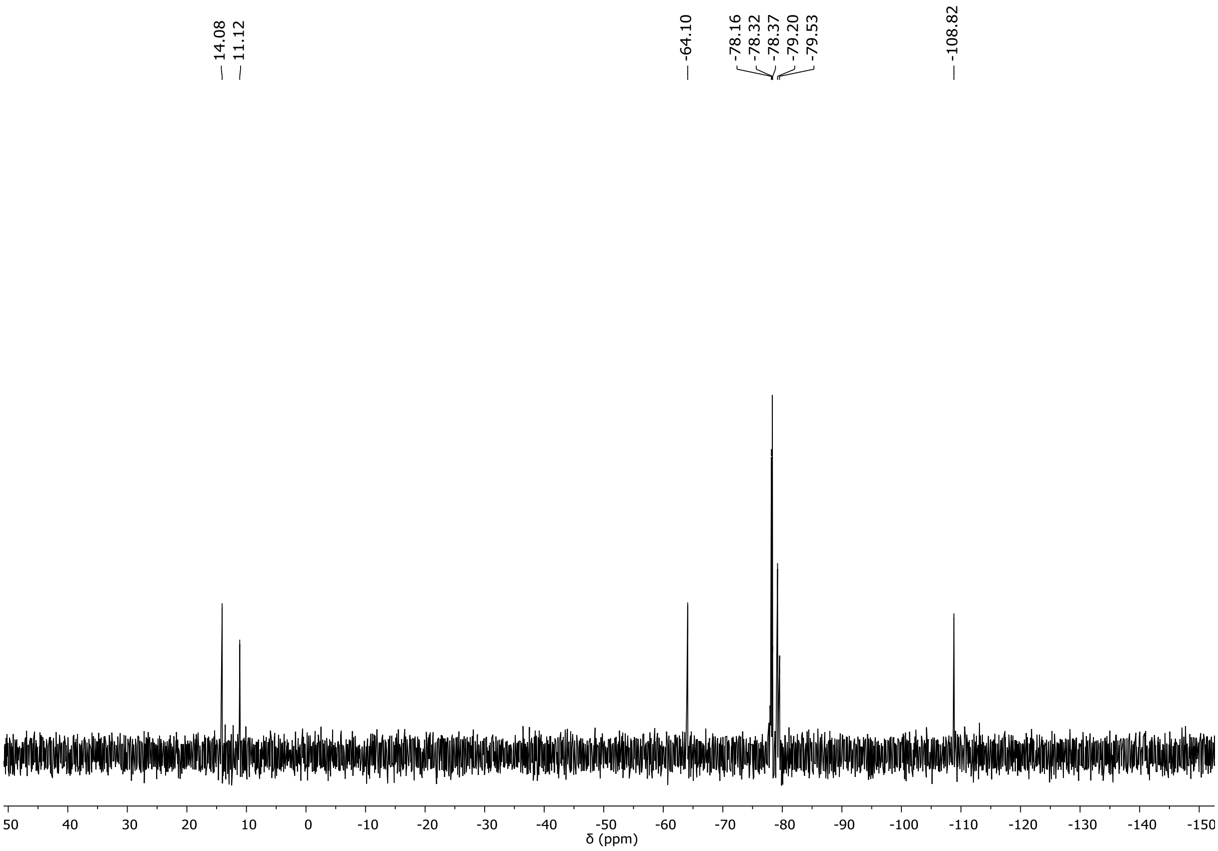

Figure S30 ^{29}Si NMR (79 MHz, CD_2Cl_2) spectrum of **DDSQ-2OSi-(T_8Ph_7)$_2$**.

DDSQ-2OSi-(T_8iBu$_7$)$_2$

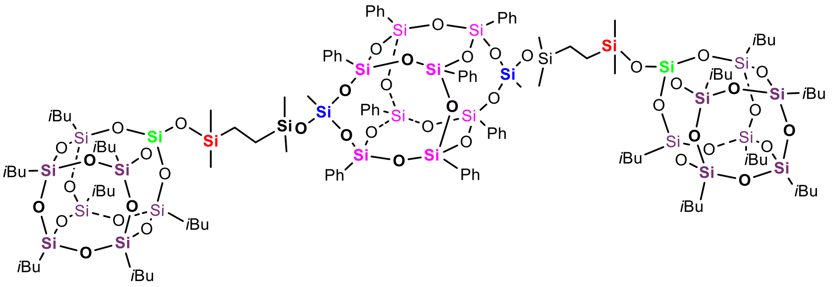

White solid. Isolated Yield 92%.

^{1}H NMR (300 MHz, CD_2Cl_2, ppm): δ = -0.02 (s, 12H, Si(CH_3)$_2$), 0.05 (s, 12H, Si(CH_3)$_2$), 0.45 (s, 6H, Si-CH_3), 0.57-0.61 (m, 36H, -CH_2-, -CH_2- *i*Bu), 0.92-0.95 (m, 126H, -CH_3 *i*Bu), 1.80-1.90 (m, 14H, -CH- *i*Bu), 7.17-7.60 (m, 40H, Ph);
^{13}C NMR (101 MHz, CD_2Cl_2, ppm): δ = -2.43 (Si-CH_3), -0.84 (Si(CH_3)$_2$), -0.35 (Si(CH_3)$_2$), 9.37 (-CH_2-), 9.51 (-CH_2-), 22.91, 22.95, 24.46, 26.06 (*i*Bu), 128.23, 128.42, 131.08, 131.45, 132.35, 134.35 (Ph);
^{29}Si NMR (79 MHz, CD_2Cl_2, ppm): δ = 12.35 (Si^M), 11.15 (Si^M), -64.13 (Si^T), -67.00, -67.77, -67.80 (Si^T-iBu), -79.20, -79.54 (Si^T-Ph), -109.48 (Si^Q);
FT-IR (ATR, cm^{-1}): 3073.31, 3051.93 (C-H phenyl), 2952.96, 2924.75, 2906.59, 2869.44 (C-H), 1594.71, 1464.58 (C-H), 1430.37, (C=C phenyl), 1228.30 (Si-C), 1087.09 (Si-O-Si), 998.75 (C-H phenyl).
EA: Anal. calcd for $C_{118}H_{204}O_{42}Si_{30}$ (%): C, 45.17, H, 6.55; found: C, 45.15; H, 6.56.

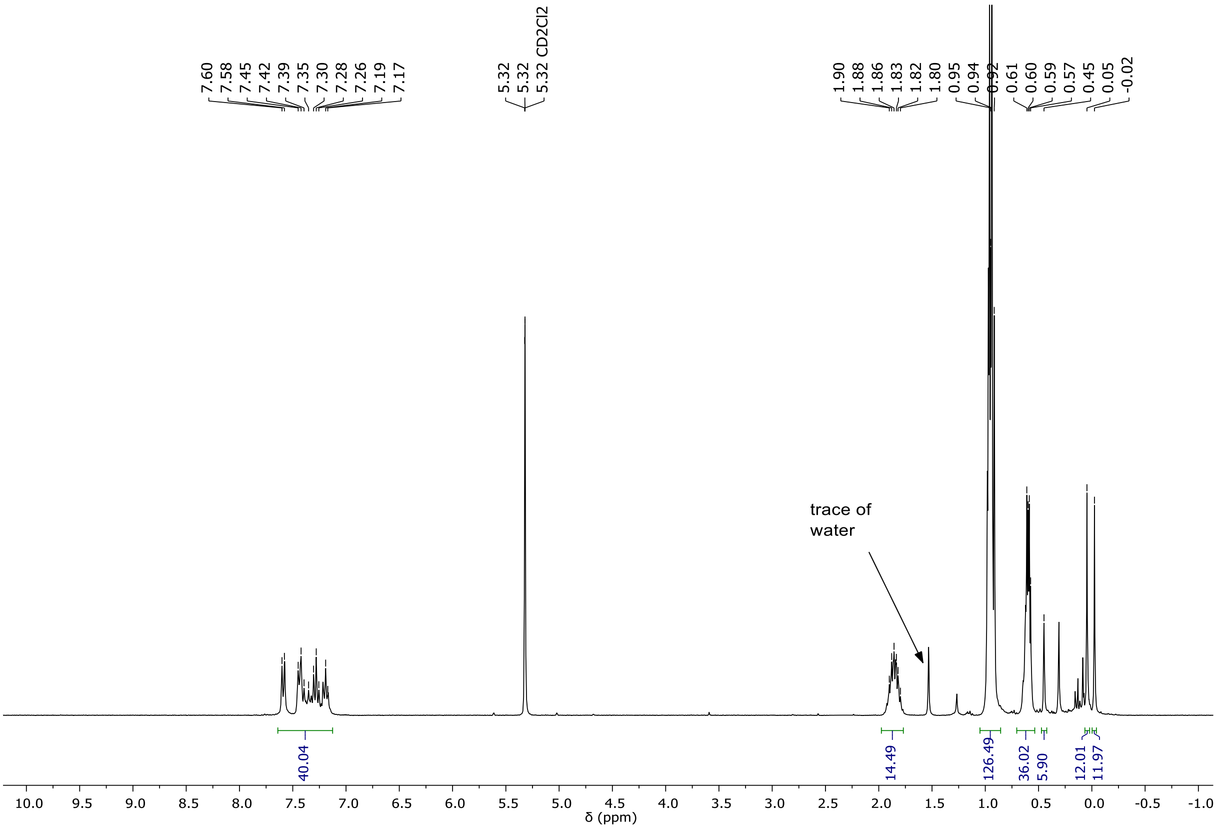

Figure S31 ^{1}H NMR (300 MHz, CD_2Cl_2) spectrum of ***DDSQ-2OSi-(T_8iBu$_7$)$_2$****.*

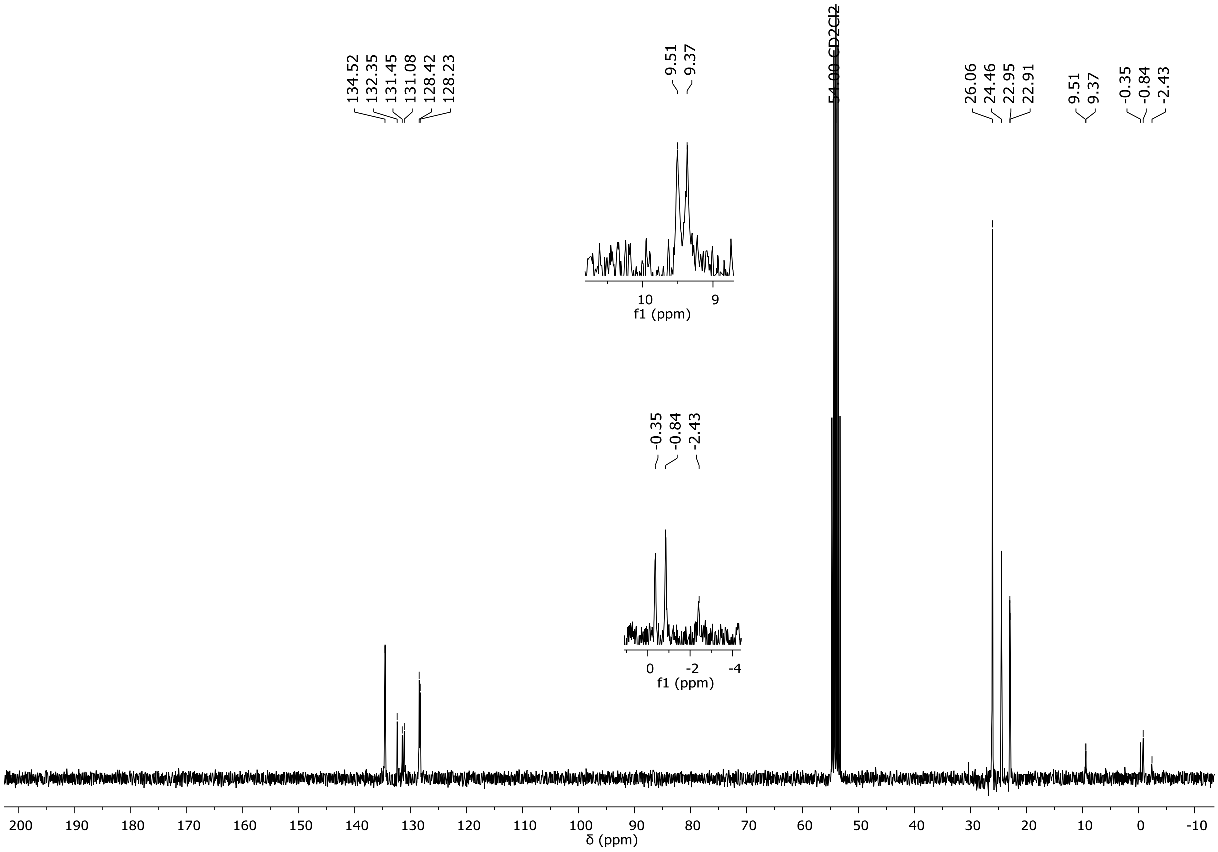

Figure S32 ^{13}C NMR (101 MHz, CD_2Cl_2) spectrum of **DDSQ-2OSi-(T_8iBu$_7$)$_2$**.

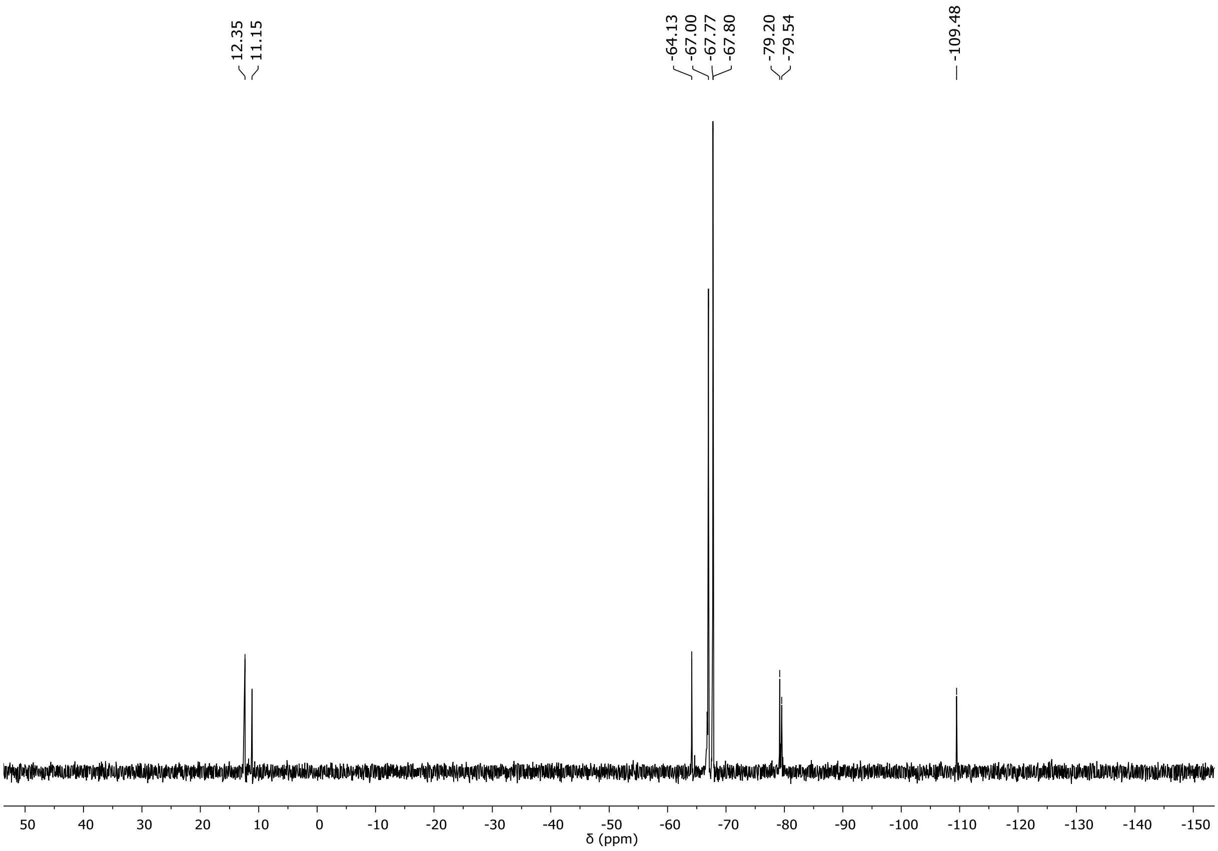

Figure S33 ^{29}Si NMR (79 MHz, CD_2Cl_2) spectrum of **DDSQ-2OSi-(T_8iBu$_7$)$_2$**.

DDSQ-2OSi-(T_8Et_7)$_2$

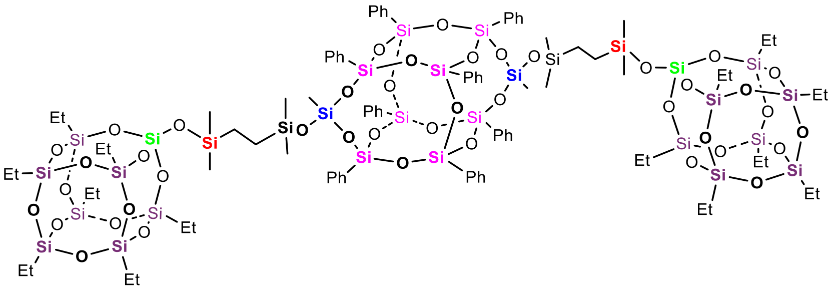

White solid. Isolated Yield 93%

^{1}H NMR (300 MHz, CD_2Cl_2, ppm): δ = 0.00 (s, 12H, $Si(CH_3)_2$), 0.05 (s, 12H, $Si(CH_3)_2$), 0.47 (s, 6H, Si-CH_3), 0.56-0.61 (m, 36H, -CH_2-, -CH_2- Et), 0.95-0.98 (m, 42H, -CH_3 Et), 7.17-7.60 (m, 40H, Ph);
^{13}C NMR (101 MHz, CD_2Cl_2, ppm): δ = -2.45 (Si-CH_3), -0.86 ($Si(CH_3)_2$), -0.41 ($Si(CH_3)_2$), 4.50 (-CH_3 Et), 6.84 (-CH_2- Et), 9.40 (-CH_2-), 9.53 (-CH_2-), 128.24-128.43, 131.10, 131.48, 132.38, 134.52, 134.56 (Ph);
^{29}Si NMR (79 MHz, CD_2Cl_2, ppm): δ = 12.82 (Si^M), 11.15 (Si^M), -64.13 (Si^T), -65.18, -65.63 (Si^T-Et), -79.21, -79.54 (Si^T-Ph), -108.58 (Si^Q);
FT-IR (ATR, cm^{-1}): 3073.29, 3051.87 (C-H phenyl), 2961.01, 2920.68, 2879.93 (C-H), 1594.67, 1460.72 (C-H), 1430.38, (C=C phenyl), 1252.32 (Si-C), 1064.12 (Si-O-Si), 1012.44 (C-H phenyl).
EA: Anal. calcd for $C_{90}H_{148}O_{42}Si_{30}$ (%): C, 39.38, H, 5.44; found: C, 39.40; H, 5.40.

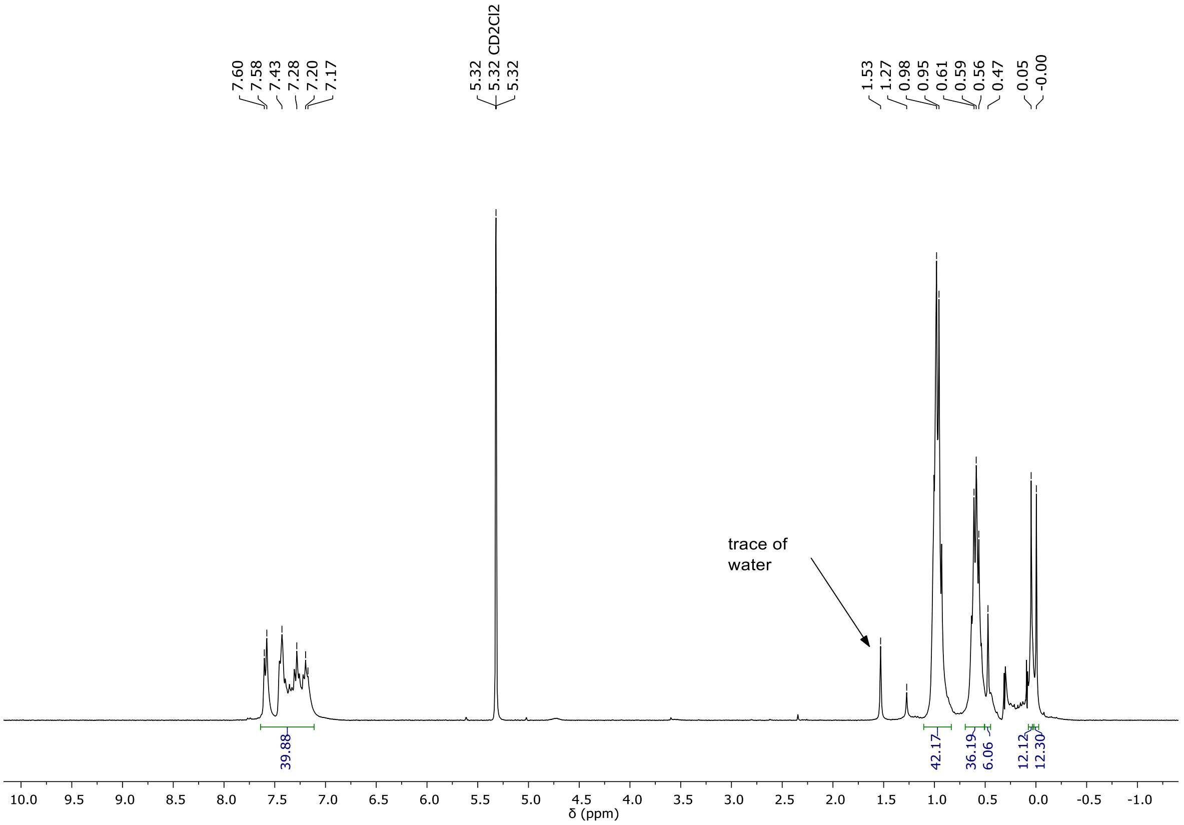

Figure S34 ^{1}H NMR (300 MHz, CD_2Cl_2) spectrum of ***DDSQ-2OSi-(T_8Et_7)$_2$****.*

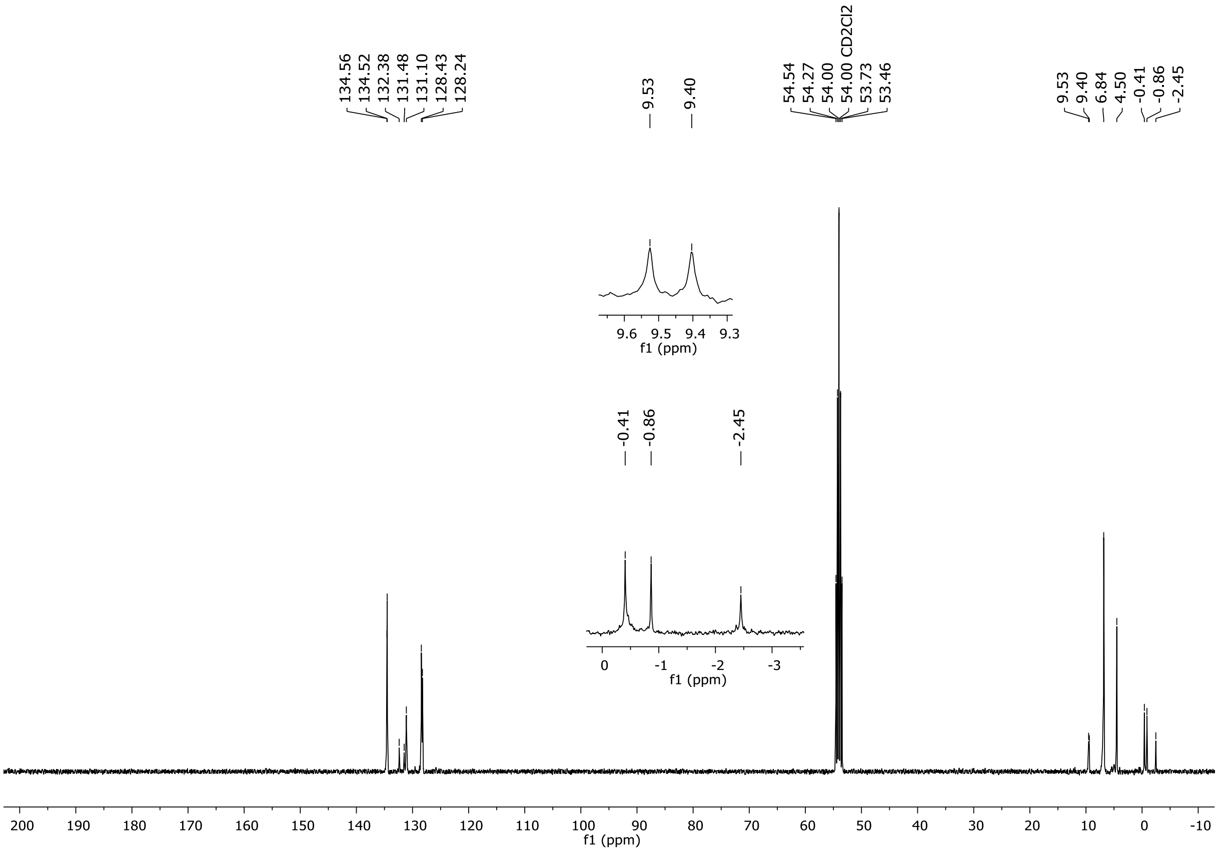

Figure S35 ^{13}*C NMR (101 MHz,* CD_2Cl_2*) spectrum of* ***DDSQ-2OSi-(T_8Et_7)$_2$***.

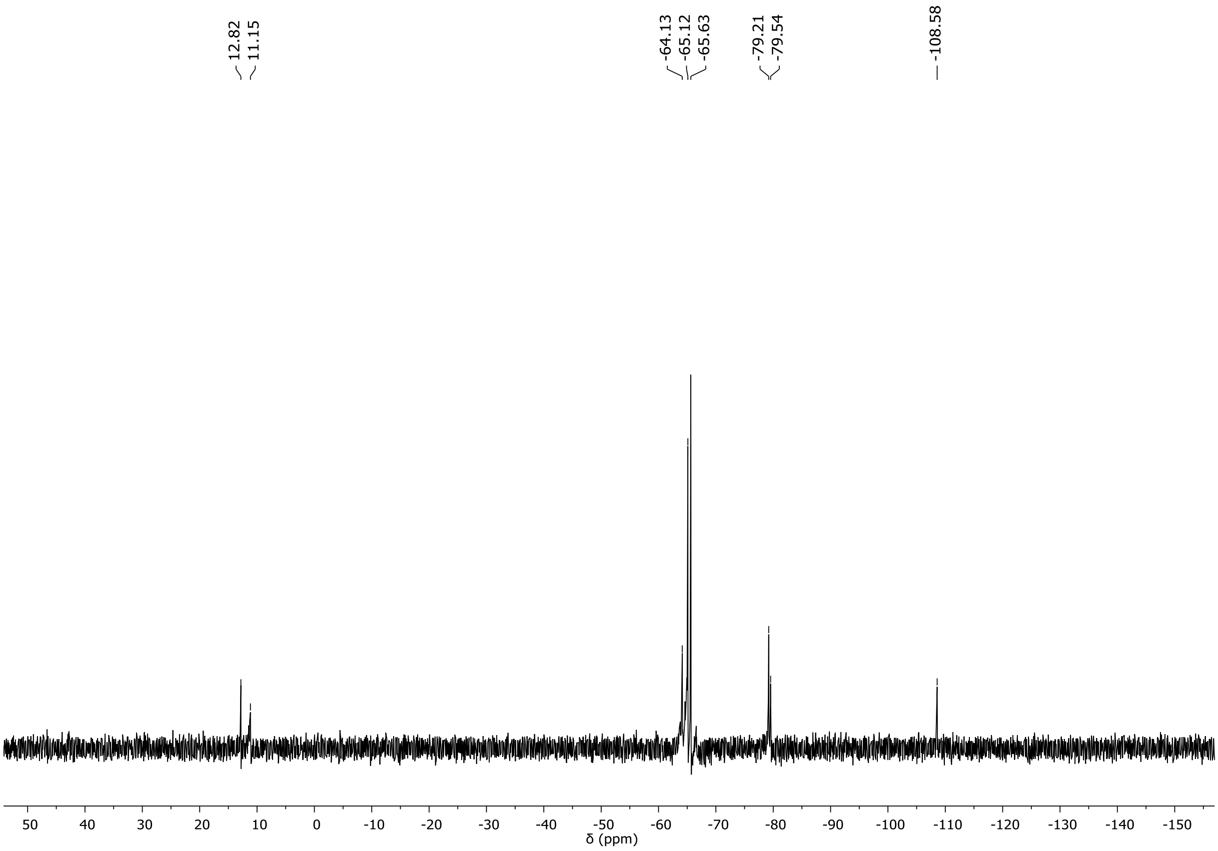

Figure S36 ^{29}*Si NMR (79 MHz,* CD_2Cl_2*) spectrum of* ***DDSQ-2OSi-(T_8Et_7)$_2$***.

DDSQ-2OSi-(T_8iOc$_7$)$_2$

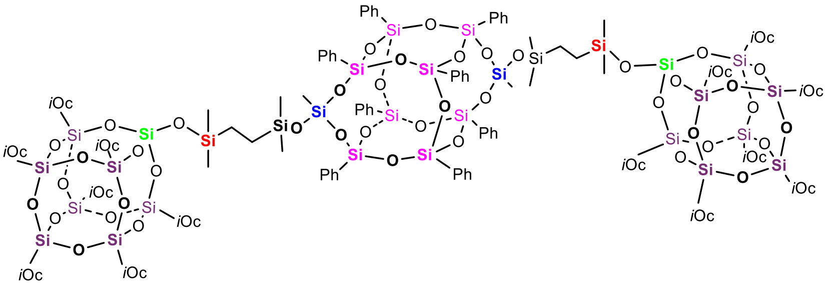

White oil. Isolated Yield 86%

^{1}H NMR (300 MHz, CD_2Cl_2, ppm): δ = -0.02 (s, 12H, $Si(CH_3)_2$), 0.05 (s, 12H, $Si(CH_3)_2$), 0.45 (s, 6H, $Si-CH_3$), 0.54-1.35 (m, 8H, $-CH_2-$, 224H, $-CH_2$, $-CH_3$ *i*Oc), 1.83-1.84 (3, 14H, -CH- *i*Oc), 7.19-7.60 (m, 40H, Ph);
^{13}C NMR (101 MHz, CD_2Cl_2, ppm): δ = -2.39 ($Si-CH_3$), -0.74 ($Si-CH_3$), -0.28 $(Si-CH_3)_2$, 9.38 ($-CH_2-$), 9.50 ($-CH_2-$), 24.03, 24.13, 25.27, 25.57, 26.10, 30.53, 31.64, 31.76 (*i*Oc), 128.23, 128.41, 131.02-132.35, 134.49 (Ph);
^{29}Si NMR (79 MHz, CD_2Cl_2, ppm): δ = 12.26 (Si^M), 11.16 (Si^M), -64.15 (Si^T), -67.32, -68.11, -68.14 (Si^T-iOc), -79.20, -79.53 (Si-Ph), -109.64 (Si^Q);
FT-IR (ATR, cm^{-1}): 3073.35, 3052.21 (C-H phenyl), 2951.47, 2906.70, 2858.43 (C-H), 1594.73 1466.79 (C-H), 1430.33 (C=C phenyl), 1376.60, 1363.81, 1251.66, 1226.19 (Si-C), 1072.50 (Si-O-Si), 998.43 (C-H phenyl).
EA: Anal. calcd for $C_{174}H_{316}O_{42}Si_{30}$ (%): C, 53.27, H, 8.12; found: C, 53.30; H, 8.13.

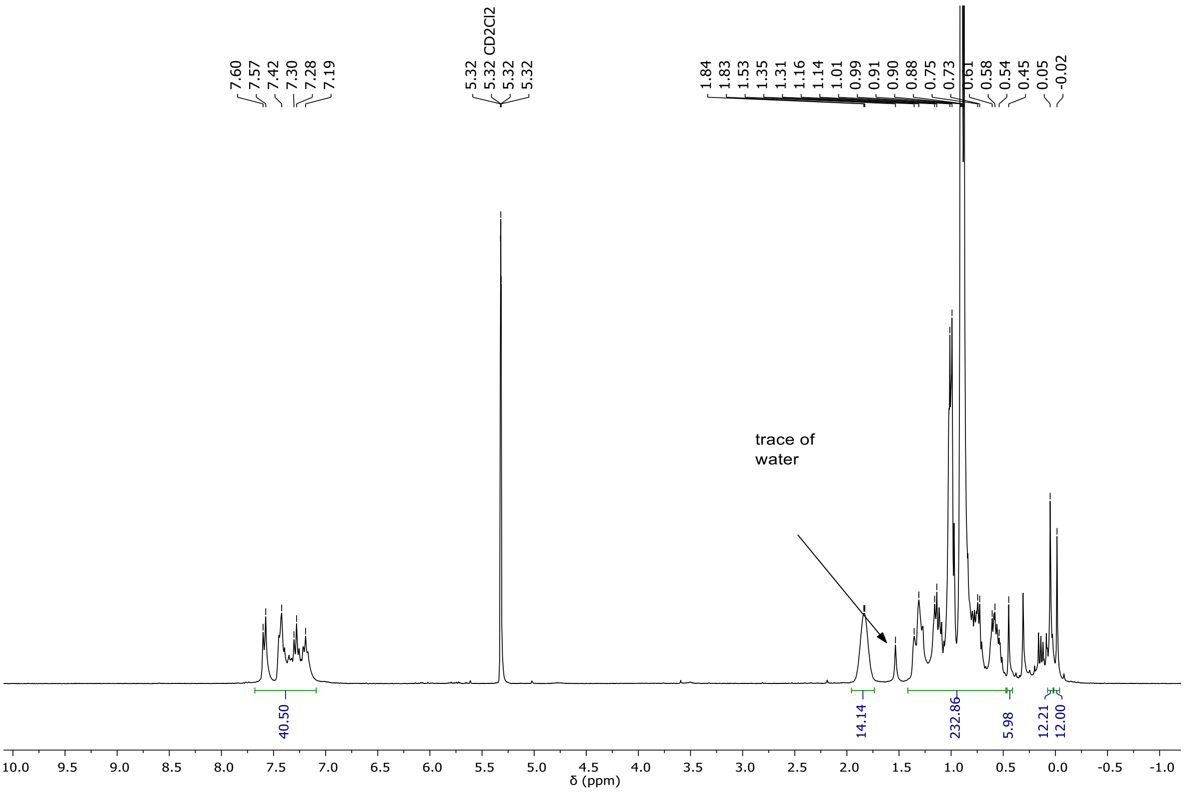

Figure S37 ^{1}H NMR (300 MHz, CD_2Cl_2) spectrum of ***DDSQ-2OSi-(T_8iOc$_7$)$_2$**.*

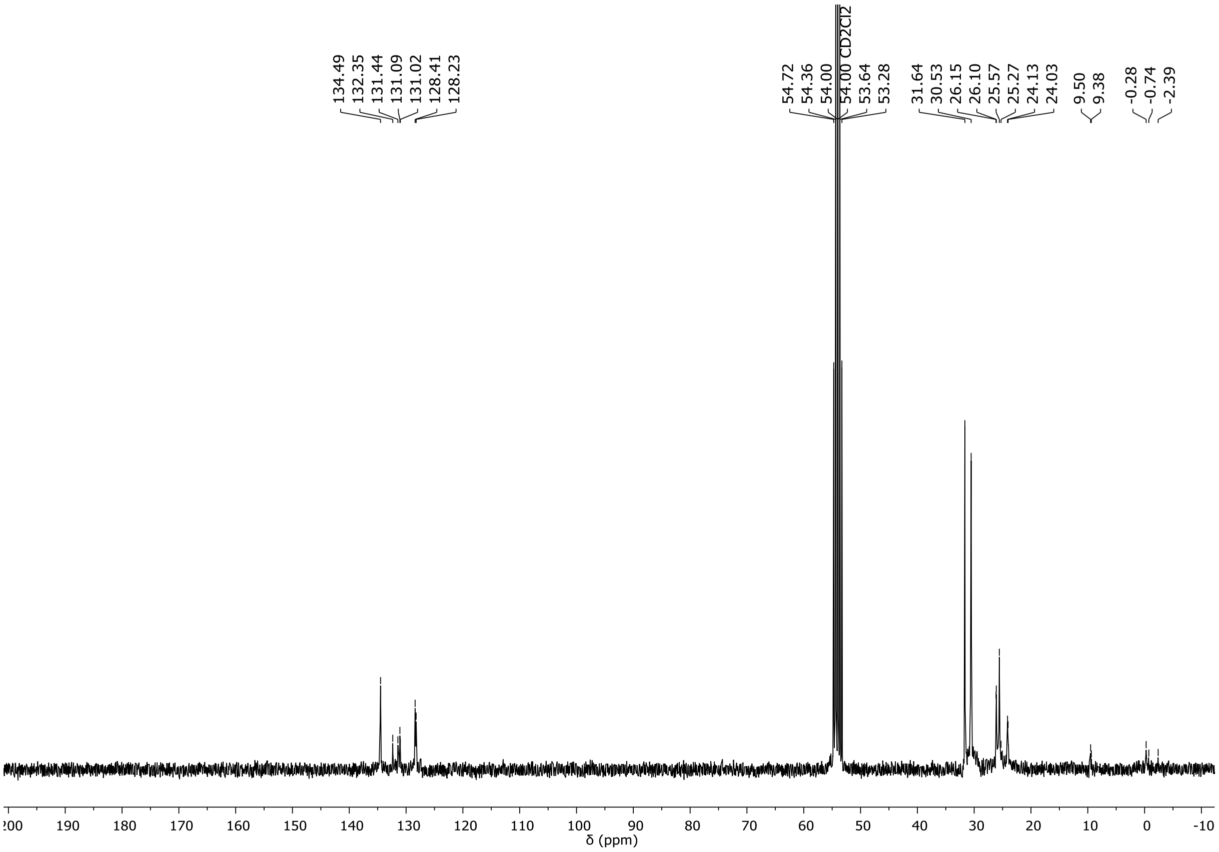

Figure S38 ^{13}C *NMR (101 MHz,* CD_2Cl_2*) spectrum of* ***DDSQ-2OSi-(T₈iOc₇)₂.***

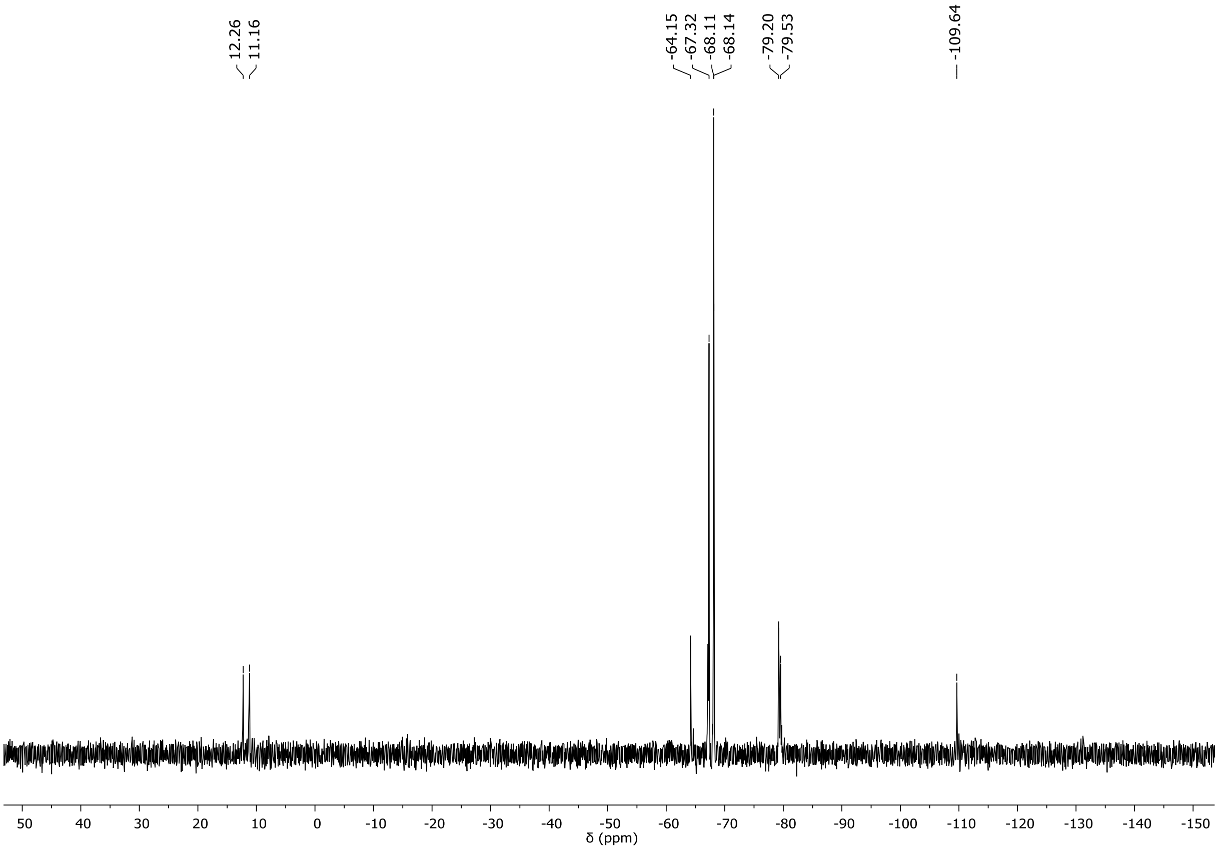

Figure S39 ^{29}Si *NMR (79 MHz,* CD_2Cl_2*) spectrum of* ***DDSQ-2OSi-(T_8iOc_7)$_2$.***

3 DFT Calculations

Table S1. Atomic coordinates of systems consisted of four molecules of Ph_7T_8-T_8Et_7 compound for clusters 1-3.

Atomic coordinates of systems consisted of four molecules of Ph_7T_8-T_8Et_7 compound									
	Cluster **1**			Cluster **2**			Cluster **3**		
Atom	x	y	z	x	y	z	x	y	z
Si	43.90400	-67.26300	-3.73400	42.00400	-67.29500	5.59000	31.08500	-64.56900	-4.17000
Si	44.51600	-66.21800	-1.00800	38.96400	-64.32400	4.09000	29.85900	-60.71200	-6.06200
Si	41.50500	-66.03900	-0.44100	39.53600	-61.88500	2.51100	31.78800	-58.76500	-7.20800
Si	43.52300	-70.27100	-2.87800	40.83800	-60.76100	0.01400	33.24200	-57.88600	-9.68600
Si	44.19900	-69.09000	-0.20500	41.06200	-58.03700	1.48000	35.09900	-56.00500	-8.05300
Si	41.23900	-68.85500	0.47400	38.43100	-57.49900	0.27800	33.19100	-53.68900	-8.60500
Si	40.53400	-69.66800	-2.33500	37.97400	-60.12700	-1.00700	31.43000	-55.64600	-10.30100
Si	40.99300	-66.79400	-3.17300	39.58900	-59.18900	3.88000	33.65000	-56.93200	-5.57900
Si	-1.34000	-60.08400	-13.86000	36.93700	-58.43300	2.86200	31.67300	-54.60100	-6.23100
Si	1.18900	-58.55000	-14.51600	36.69800	-61.05600	1.62100	30.02400	-56.55800	-7.77100
Si	-0.01000	-55.86400	-13.85500	46.88500	-59.50100	-1.08200	39.99000	-58.37400	-7.75400
Si	-0.36300	-60.32500	-11.00500	49.67000	-57.45500	1.83200	42.99900	-59.19300	-4.45600
Si	2.13200	-58.91400	-11.79400	52.49500	-58.04700	1.19800	45.74700	-59.94000	-4.72800
Si	0.77300	-56.31400	-10.95200	54.88900	-59.92000	1.39100	46.73500	-62.75100	-4.33600
Si	-1.77900	-57.78300	-10.27700	56.02000	-58.17800	3.43300	47.66200	-62.23900	-1.45800
Si	-2.47300	-57.37900	-13.07900	57.33400	-56.49400	1.25500	50.26400	-61.17500	-2.65900
Si	-1.77100	-60.56000	-0.21100	55.91900	-58.30100	-0.85100	49.49000	-61.95700	-5.43000
Si	-0.30900	-63.29200	-0.13300	53.75000	-56.18500	3.27300	46.84500	-59.32200	-2.00200
Si	-0.56300	-63.92900	-3.02600	55.08700	-54.43900	1.20500	49.34500	-58.41000	-3.26100
Si	1.02800	-59.42700	-0.52900	53.75600	-56.24100	-0.89200	48.36600	-59.24000	-5.95800
Si	2.52000	-62.06700	-0.49300	47.02800	-53.66600	-9.01400	44.06300	-50.90200	-13.54800
Si	2.13700	-62.52900	-3.41900	45.74000	-57.11500	-6.63500	41.51500	-54.56800	-13.13000
Si	0.82200	-59.84600	-3.45000	45.88200	-59.95600	-7.79900	40.05200	-55.96300	-14.99200
Si	-1.88800	-61.31500	-3.08300	47.43300	-61.71400	-9.69200	41.91500	-57.74800	-16.74700
Si	-3.45700	-66.45200	-0.43200	44.77300	-62.95000	-10.71400	39.79500	-58.02000	-18.89700
Si	-1.14900	-68.30300	-0.22600	45.08700	-65.23200	-8.75200	38.27200	-59.92900	-17.41000
Si	-1.77800	-69.12500	2.70100	47.54000	-63.89300	-7.48400	40.38700	-59.87400	-15.32500
Si	-5.41800	-68.54100	-1.37200	43.25500	-61.07100	-8.76800	38.17600	-55.85400	-17.29700
Si	-3.07600	-70.49600	-1.12400	43.47800	-63.38200	-6.83900	36.62800	-57.92500	-15.79400
Si	-3.76600	-71.18100	1.72600	45.91200	-62.18700	-5.71000	38.72600	-57.97600	-13.69100
Si	-6.08400	-69.18300	1.57700	28.64900	-56.81900	1.99000	27.32600	-47.47700	-5.87300
Si	-4.04200	-67.28200	2.47400	29.89400	-54.79600	-1.80100	26.04800	-48.04500	-10.17500
Si	42.65600	-72.01300	-10.73700	28.59900	-55.79700	-4.28600	23.50600	-48.73100	-11.24400
Si	42.44200	-74.81200	-9.59500	26.12300	-55.72600	-5.96400	20.97400	-47.34100	-12.05400
Si	40.00600	-73.71300	-8.05900	26.57600	-58.66700	-6.68500	19.63600	-49.94800	-12.47500
Si	40.89300	-72.41400	-13.12700	28.49400	-57.87300	-8.96200	20.62200	-50.01500	-15.30000
Si	40.72600	-75.24000	-12.00300	27.93100	-54.88400	-8.29100	21.97900	-47.38400	-14.85200
Si	38.21300	-74.44400	-10.45700	28.99700	-58.68100	-4.88600	22.12900	-51.48000	-11.70400

Si	38.58700	-71.57900	-11.46500	30.91100	-57.83300	-6.99500	23.19200	-51.29500	-14.44700
Si	40.37400	-71.02700	-9.02900	30.43000	-54.93200	-6.60500	24.58500	-48.54300	-14.14600
Si	41.11600	-68.35900	-8.10000	42.94700	-68.24600	3.00000	31.73100	-65.23400	-7.16000
Si	40.34200	-64.86800	-5.43300	40.20800	-68.43500	1.80300	29.34400	-64.79200	-8.81200
Si	-3.50200	-64.18400	-8.38600	41.56500	-70.87700	0.51400	30.30800	-66.73500	-10.82700
Si	-5.26700	-66.52100	-9.49600	42.35100	-69.08900	-1.89700	32.06200	-64.58700	-12.19100
Si	-5.44800	-65.20600	-12.23900	41.34200	-66.66000	-0.42800	31.00500	-62.66000	-10.26400
Si	-1.17900	-65.40700	-9.39200	44.17800	-70.78600	1.81500	32.59400	-67.38400	-8.93600
Si	-2.64900	-67.96300	-10.28600	45.02200	-68.81700	-0.37400	34.34000	-65.31200	-10.42600
Si	-2.88900	-66.59100	-12.93100	43.95600	-66.32900	0.87500	33.45200	-63.27500	-8.64100
Si	-1.20400	-64.30700	-12.20800	48.33000	-62.04100	0.01400	39.96000	-61.38400	-8.03700
Si	-3.67100	-62.99100	-11.24500	51.39700	-61.93400	-0.22200	42.21900	-63.30900	-8.14500
Si	-5.58500	-60.76500	-11.10000	51.38000	-64.97200	-0.20600	40.69100	-65.16300	-9.77600
Si	-5.39400	-57.41000	-14.05100	51.32500	-65.11100	2.82500	39.89400	-66.33000	-7.15800
Si	1.57700	-54.43400	-5.33800	51.34600	-62.15500	2.93100	41.49400	-64.41400	-5.48100
Si	2.11500	-51.92500	-3.77400	48.34500	-64.93700	-0.21100	38.31500	-63.30800	-9.67600
Si	1.10000	-53.18400	-1.33600	48.44900	-65.25200	2.86100	37.48000	-64.48100	-7.00800
Si	4.43600	-55.01100	-4.91200	48.43500	-62.24200	2.95900	39.08600	-62.51200	-5.40400
Si	4.94800	-52.61700	-3.09200	49.65700	-55.25500	-8.59100	46.24100	-52.40400	-12.11400
Si	4.06900	-54.27500	-0.62100	50.27800	-56.67200	-5.88100	45.53700	-54.34200	-9.91100
Si	3.71100	-56.58400	-2.46500	53.12600	-56.50800	-6.56700	48.52400	-54.35100	-9.04700
Si	0.76400	-55.78300	-2.75400	52.90800	-59.20300	-7.99300	49.02200	-56.53900	-11.09100
Si	-2.12500	-56.47500	-1.89800	50.03800	-59.39300	-7.22700	46.11100	-56.77100	-11.90200
Si	-3.56900	-59.48600	-4.68900	52.60600	-55.15000	-9.24100	49.17000	-52.25300	-11.31000
Si	-11.43400	-66.61700	-2.85800	52.16300	-57.80500	-10.55700	49.58900	-54.63500	-13.37600
Si	-12.17200	-64.04900	-4.33000	49.29800	-58.07300	-9.83900	46.56500	-54.73100	-14.10900
Si	-9.57500	-62.62100	-3.64900	29.10900	-59.75400	1.71100	25.72100	-49.91500	-5.55100
Si	-10.25200	-67.51700	-5.56900	28.67500	-62.08000	0.07000	23.48000	-50.45300	-7.45100
Si	-11.21600	-64.98000	-7.03900	28.83200	-63.76300	2.64700	22.95100	-52.37200	-5.35300
Si	-8.45000	-63.90400	-6.18500	31.95400	-63.82200	2.46200	24.59500	-54.64600	-6.32300
Si	-7.51300	-66.42200	-4.71200	31.81300	-62.43400	-0.24700	25.15300	-52.63300	-8.61000
Si	-8.73300	-65.25300	-2.24400	29.20200	-61.21400	4.32900	25.04500	-51.69000	-3.33600
Si	-8.83200	-65.33000	0.50400	32.08900	-61.19000	3.97000	26.92800	-53.76400	-4.64200
Si	-5.88000	-65.36200	3.96700	32.11500	-59.91700	1.36800	27.52300	-51.82400	-6.87200
O	42.85500	-73.58500	-10.52000	43.21700	-67.74000	4.54700	31.82600	-64.97300	-5.53400
O	41.54300	-74.23500	-8.34200	44.09100	-69.34800	2.54800	32.73900	-66.37900	-7.68100
O	41.31000	-73.93300	-12.77700	41.42100	-68.69400	2.84900	30.29500	-65.57400	-7.78600
O	39.12400	-74.97400	-11.72200	43.03800	-66.83700	2.13300	32.36500	-63.77700	-7.58500
O	39.36300	-72.20700	-12.71600	42.57900	-71.08600	1.77100	31.11700	-67.36900	-9.60200
O	38.10000	-72.82300	-10.50500	42.95900	-66.16500	-0.38700	32.60900	-62.59300	-9.84700
O	41.53100	-75.61100	-10.64700	43.89400	-68.57700	-1.51100	33.47800	-64.37400	-11.51900
O	41.80900	-71.64600	-12.11700	40.47900	-69.69000	0.79600	29.49600	-65.50800	-10.17600
O	39.33200	-74.45300	-9.28100	40.58400	-67.04400	0.96700	30.18400	-63.42700	-9.08000
O	41.84900	-71.42100	-9.47900	42.28100	-70.43000	-0.93500	31.52700	-66.03700	-11.61000
O	39.63900	-70.83900	-10.50300	41.32200	-68.00200	-1.30900	30.90100	-63.57400	-11.63500

O	39.58300	-72.13200	-8.14200	44.58900	-70.19300	0.36600	33.49500	-66.64300	-10.03700
O	40.08300	-69.56600	-8.40500	45.06100	-67.49600	0.54200	34.45100	-64.50000	-9.06800
O	44.80200	-66.76800	-2.51100	39.80600	-63.25100	3.32500	31.28500	-60.25900	-6.66700
O	42.96600	-65.74700	-0.98800	40.11700	-60.68200	3.37600	33.11600	-58.26100	-6.42200
O	44.43900	-69.60100	-1.69600	39.90800	-61.78700	0.91100	32.10000	-58.73200	-8.85100
O	42.70900	-69.51700	0.29000	37.91800	-61.63700	2.61800	30.57100	-57.78800	-6.82300
O	42.03400	-70.30600	-2.32300	40.17400	-58.13500	2.80700	34.74300	-56.25100	-6.52700
O	40.26800	-69.08400	-0.87700	37.30500	-61.08500	0.07300	30.53100	-56.55500	-9.29600
O	44.38600	-67.53400	0.03500	37.78800	-57.45600	1.86000	32.80200	-53.82000	-7.07700
O	43.69100	-68.87100	-3.70000	41.32200	-59.57000	0.99500	34.33300	-57.34400	-8.56400
O	41.57800	-67.28800	0.61900	39.63100	-60.12500	-0.93100	32.71900	-56.59400	-10.42500
O	42.52300	-66.49600	-3.57900	40.03200	-57.70600	0.28500	34.38200	-54.77900	-8.91100
O	40.68700	-68.37400	-3.32600	37.56200	-58.66300	-0.46300	31.85900	-54.33800	-9.37500
O	40.53900	-66.28900	-1.68600	37.95900	-59.12900	3.99200	32.39400	-55.92100	-5.63600
O	40.14100	-65.85800	-4.16600	36.16900	-59.54400	1.95700	30.43500	-55.14800	-7.09100
O	-4.84700	-65.12900	-8.74600	47.20000	-61.05700	-0.44800	40.19700	-59.84900	-8.40700
O	-5.42100	-66.35800	-11.09500	48.33400	-63.46300	-0.83600	39.32500	-62.07300	-9.37200
O	-1.44100	-66.93100	-9.98800	49.85100	-61.52500	0.06000	41.34100	-62.11100	-7.52200
O	-3.11500	-67.77700	-11.85400	47.75300	-62.41100	1.45600	38.99600	-61.57500	-6.74100
O	-1.03900	-64.33800	-10.60500	49.88900	-65.55700	-0.09500	39.09500	-64.75100	-9.71100
O	-1.52400	-65.84400	-12.56900	49.83300	-61.54700	2.83800	40.55800	-63.08700	-5.07200
O	-3.92400	-67.38800	-9.45300	49.93600	-65.90300	2.79700	38.42800	-65.66400	-7.40800
O	-2.18200	-65.10400	-8.12800	51.25700	-63.47900	-0.78400	41.52300	-63.81500	-9.53500
O	-4.09500	-65.47300	-13.11800	51.81100	-62.04000	1.33500	42.27900	-64.40900	-6.92500
O	-3.26200	-63.29300	-9.70500	51.97700	-64.95000	1.31200	40.83200	-66.01300	-8.42700
O	-2.57400	-63.48700	-12.37900	51.34600	-63.66300	3.50600	40.44600	-65.63700	-5.77600
O	-5.00000	-63.89900	-11.39700	47.79300	-65.11700	1.34500	37.42900	-63.50500	-8.29100
O	-4.12900	-61.45300	-11.47000	48.65900	-63.74800	3.42500	38.08700	-63.77400	-5.70000
O	-0.31500	-59.14500	-14.73900	50.89000	-58.18400	1.00100	44.24600	-59.70700	-5.29600
O	1.11900	-56.91600	-14.39500	52.82100	-57.47600	2.68300	45.76000	-59.85600	-3.10300
O	1.10100	-59.56900	-10.77100	53.54900	-59.23800	0.91700	46.14200	-61.45700	-5.12900
O	1.85600	-57.29000	-11.72800	52.73000	-56.72400	0.27100	46.94400	-58.91900	-5.23600
O	-1.57400	-59.37400	-10.63400	55.23100	-56.76300	3.51700	47.49100	-60.62900	-1.24400
O	-0.22800	-57.24900	-10.11600	54.55300	-57.63400	-1.21600	48.73700	-60.78800	-6.25100
O	1.89100	-59.38800	-13.31300	56.43800	-55.19100	1.62100	50.13100	-59.58700	-2.44400
O	-0.54700	-60.72000	-12.59800	54.98100	-59.29500	2.87700	46.73100	-62.59300	-2.69800
O	0.17300	-55.56200	-12.30800	56.02300	-59.49000	0.29800	48.37000	-62.78100	-4.58300
O	-2.42700	-59.02000	-13.21600	57.00600	-57.72900	2.23300	49.19700	-62.17600	-2.04000
O	-2.33800	-56.84500	-11.51200	56.53800	-56.94900	-0.09500	50.39400	-61.43000	-4.22700
O	-1.35700	-56.73600	-14.12300	53.90400	-54.97200	2.18200	47.89600	-58.24200	-2.59800
O	-3.95000	-56.84100	-13.48800	54.63900	-55.01300	-0.27500	49.43900	-58.68000	-4.82700
O	2.22000	-52.99900	-4.99800	48.55300	-54.01700	-8.61900	45.21300	-51.24800	-12.35900
O	1.04900	-52.34500	-2.71000	50.98400	-55.10000	-9.52000	47.81100	-51.95800	-12.25400
O	4.87800	-53.44800	-4.51700	50.03900	-55.52200	-7.05400	46.01200	-52.93600	-10.62900
O	5.07600	-53.60400	-1.76400	48.91100	-56.69800	-9.01200	45.85600	-53.66000	-13.10300

O	4.67700	-55.90900	-3.61600	52.96800	-55.27700	-7.65700	48.67100	-53.05000	-9.92200
O	3.64300	-55.76100	-1.06700	49.64900	-59.25600	-8.82500	46.43800	-56.21200	-13.36800
O	3.51500	-51.93100	-2.97200	52.38000	-58.95800	-9.46200	49.35800	-55.97300	-12.55800
O	2.94000	-55.24100	-5.52100	51.85800	-56.72600	-5.54700	46.93800	-54.61300	-9.12500
O	2.68400	-53.40300	-0.86500	49.59600	-58.01900	-6.47800	45.41600	-55.47400	-11.14600
O	0.75400	-54.77500	-3.98100	53.52900	-57.81900	-7.38800	49.36400	-55.51800	-9.89500
O	2.16800	-56.50500	-2.98600	51.69400	-59.38000	-7.01900	47.46900	-57.12100	-11.10000
O	0.81300	-54.74200	-1.57100	52.96100	-56.51100	-10.04400	49.79300	-53.58900	-12.09900
O	-0.64200	-56.65900	-2.56100	50.55300	-57.48000	-10.72000	48.16500	-54.45800	-14.14900
O	-0.88400	-61.83900	0.26600	46.32900	-58.40400	-7.44800	40.81200	-54.59000	-14.53800
O	-0.97100	-63.80200	-1.48100	44.26000	-60.20000	-7.82400	38.66400	-55.63300	-15.77500
O	1.89600	-60.70000	0.07400	46.49800	-60.47700	-9.19700	40.95600	-56.47300	-16.28700
O	2.69300	-61.86400	-2.06900	46.38800	-60.92300	-6.64700	40.03700	-57.04000	-13.82200
O	1.47100	-59.32900	-2.06800	44.06500	-61.58100	-10.07800	39.29800	-56.56700	-18.22100
O	1.29400	-61.26900	-3.99400	46.72100	-63.55300	-6.10700	39.19000	-59.25000	-14.49200
O	1.30700	-63.17200	-0.51700	44.37700	-64.65000	-7.30900	37.24300	-59.31400	-16.34500
O	-0.54400	-59.50900	-0.55400	46.35300	-62.61200	-10.49600	41.21900	-58.34900	-18.07500
O	1.08900	-63.66800	-3.07400	47.70800	-62.47300	-8.28400	41.45800	-58.67300	-15.49000
O	-2.29700	-61.41800	-1.48700	44.32000	-64.21300	-9.75700	38.48900	-58.98100	-18.68500
O	-0.69400	-60.18000	-3.16600	46.60900	-64.78100	-8.48700	39.62400	-60.29800	-16.65000
O	-1.31600	-62.67500	-3.72800	42.86500	-62.47900	-8.07400	37.03800	-56.97300	-17.05700
O	-3.27600	-60.75900	-3.83500	44.33500	-62.30400	-5.98000	37.44000	-57.41000	-14.50600
O	-12.42600	-65.55600	-3.70800	28.21400	-58.41300	1.83100	25.99400	-48.37800	-5.38300
O	-11.10100	-63.16800	-3.51700	28.55700	-60.57400	2.99300	24.86300	-50.33500	-4.20200
O	-11.24500	-66.40600	-6.20200	29.00200	-60.50800	0.22300	24.90000	-49.99300	-6.89400
O	-9.60800	-64.76900	-6.95400	30.69800	-59.47100	1.98600	27.08300	-50.69100	-5.84600
O	-8.64700	-67.25800	-5.57500	29.15100	-62.80100	3.91600	23.71900	-52.44200	-3.95800
O	-7.55500	-64.95000	-5.33600	32.11400	-60.87200	0.05900	26.40900	-51.76700	-8.08700
O	-11.93200	-64.02600	-5.96400	31.88200	-62.79200	3.71500	25.62300	-54.65400	-5.03100
O	-10.62100	-67.49500	-3.95300	28.43800	-62.77300	1.48900	22.70400	-50.97200	-6.12300
O	-9.27300	-62.95900	-5.15500	30.15600	-62.66500	-0.30300	23.84800	-51.71000	-8.34000
O	-10.37600	-65.59500	-2.19600	30.35100	-64.18900	2.23900	23.39200	-53.59400	-6.30900
O	-8.15600	-66.37500	-3.22700	32.46800	-63.21100	1.03100	25.31200	-53.89400	-7.61000
O	-8.71100	-63.75800	-2.88100	30.68500	-60.53500	4.37900	26.41200	-52.39900	-3.91600
O	-7.99600	-65.12600	-0.82900	32.79100	-60.69800	2.63800	27.52400	-53.25200	-6.10500
O	-1.94900	-66.91900	0.00400	28.74000	-55.42600	-2.69300	24.45600	-48.28800	-10.06000
O	-1.17300	-69.05900	1.20200	28.24100	-57.36000	-4.33800	22.57600	-50.06800	-11.00500
O	-4.60800	-69.94700	-1.42000	27.33500	-55.13300	-5.03900	22.53100	-47.47700	-11.65300
O	-2.99800	-71.31800	0.29200	29.92700	-55.41300	-5.17800	24.38700	-49.00700	-12.58100
O	-6.03700	-68.38400	0.13700	27.89400	-59.35700	-5.93000	20.57100	-51.19600	-12.14300
O	-5.18600	-70.49100	1.24400	28.98000	-54.43600	-7.16000	23.54500	-47.39600	-14.45100
O	-2.05100	-69.26400	-1.12900	29.61500	-58.38900	-7.87600	21.61200	-51.21800	-14.89200
O	-4.26500	-67.45900	-1.48600	26.10100	-57.35800	-5.85200	20.16000	-48.68700	-11.65900
O	-2.86800	-70.29600	2.71300	26.51200	-55.27900	-7.52200	20.96800	-46.98200	-13.63500
O	-4.15000	-66.62800	1.00300	27.07900	-58.14500	-8.12800	19.64500	-49.62000	-14.05800

O	-5.10800	-68.47100	2.63600	28.58600	-56.28000	-9.01100	21.45500	-48.68100	-15.71800
O	-2.41600	-67.63400	2.63000	30.32700	-58.10100	-5.52200	23.25300	-51.66800	-12.91300
O	-4.52000	-65.92500	3.22500	30.99800	-56.20700	-7.33200	24.15200	-49.97100	-14.77500
C	42.21000	-69.05700	-6.76000	46.76800	-70.46900	-1.91100	36.34000	-66.29600	-12.13200
C	41.60400	-68.03800	-9.90400	48.08200	-70.75400	-2.40200	36.98600	-67.46400	-12.54400
C	41.09200	-65.89200	-6.88300	49.21300	-70.04200	-1.86500	37.36900	-68.48300	-11.66500
C	41.61400	-63.61600	-4.83900	49.08900	-69.18400	-0.75500	37.06600	-68.35500	-10.31800
C	38.72800	-64.15200	-6.17200	47.76500	-68.73700	-0.41500	36.39100	-67.19300	-9.80800
C	40.32500	-67.02000	-7.09200	39.62300	-64.40600	-0.37700	28.34100	-61.96200	-10.86800
C	44.65600	-76.43100	-9.76900	38.53300	-63.75100	-0.96700	27.32400	-61.04700	-10.92700
C	43.74200	-75.96800	-8.85300	38.18100	-64.01800	-2.33600	27.61800	-59.66600	-10.94800
C	37.56400	-74.46900	-7.12400	39.05600	-64.74300	-3.12000	28.97400	-59.24000	-10.96100
C	38.89100	-74.64700	-6.77700	40.27200	-65.32100	-2.64900	29.99700	-60.16600	-11.03800
C	42.28400	-72.00600	-15.49800	41.04200	-61.18800	-1.82200	34.03800	-59.07600	-11.00600
C	41.09500	-71.60800	-14.90800	39.73900	-72.48000	-0.82800	28.74200	-68.60100	-11.97800
C	45.18800	-71.01700	-10.42000	39.32200	-73.78700	-1.23700	28.13700	-69.25500	-13.14600
C	36.43500	-72.00000	-12.95100	39.44600	-74.90100	-0.42900	28.24900	-68.65700	-14.39700
C	40.01600	-77.02900	-13.85400	40.29100	-74.67400	0.70900	28.93600	-67.48800	-14.57100
C	36.15200	-76.17000	-10.03000	40.76000	-73.41700	1.14600	29.42300	-66.77600	-13.45700
C	45.74200	-64.94500	-0.28700	42.84700	-57.44600	1.51400	36.78300	-55.39100	-8.63600
C	40.76600	-64.81500	0.75700	37.41600	-69.07100	1.63500	27.54100	-65.65000	-6.86300
C	43.70800	-71.27000	-4.45800	36.44800	-69.97700	2.24300	26.27000	-66.22900	-6.46600
C	41.13100	-70.02600	1.90100	36.25400	-69.96100	3.64100	25.30300	-66.45400	-7.50000
C	45.48300	-70.13100	0.84400	37.10200	-69.30500	4.49500	25.55300	-66.05700	-8.84400
C	39.62100	-70.61000	-3.62300	38.28600	-68.86100	3.91400	26.64700	-65.23800	-9.08800
C	44.98100	-67.08700	-5.24800	44.48100	-63.50600	0.56100	35.14900	-61.08700	-8.13100
C	46.64000	-69.43700	1.17500	44.29700	-62.28800	1.12700	35.88300	-60.15500	-7.33100
C	47.22800	-69.58700	2.43000	44.00000	-62.06800	2.46100	35.64100	-60.14200	-5.90900
C	46.65700	-70.45700	3.41800	43.88700	-63.18500	3.34000	34.72800	-61.09100	-5.33600
C	45.49000	-71.19800	3.09400	44.03000	-64.44800	2.79400	34.14000	-62.11900	-6.16600
C	44.82800	-70.93300	1.83500	45.15500	-71.49700	4.31600	33.37100	-69.89500	-8.01100
C	43.97400	-71.17600	-9.71500	46.10100	-72.08700	5.23600	34.16600	-71.09300	-8.33000
C	40.95500	-76.03500	-13.68100	47.43400	-72.18400	4.89700	34.40500	-71.45100	-9.64500
C	39.44500	-64.29400	0.39200	47.83200	-72.00300	3.51600	33.79800	-70.71400	-10.70400
C	39.08600	-62.95000	0.84200	46.89500	-71.51300	2.61100	33.16900	-69.49900	-10.43600
C	39.96900	-62.16400	1.62000	38.36300	-56.08000	-0.86700	34.09300	-52.24500	-9.33600
C	41.28000	-62.64800	1.86300	35.27900	-62.10400	1.12800	28.31900	-55.73300	-7.90400
C	41.66600	-64.02200	1.44000	43.78700	-68.77500	-4.48500	33.33100	-63.50600	-14.73600
C	44.62800	-66.14400	-6.28700	43.94600	-68.24900	-6.11700	33.14400	-62.42000	-16.02000
C	44.80400	-66.53200	-7.68700	42.47100	-67.98800	-7.03100	31.58900	-61.94600	-16.53700
C	45.48000	-67.80100	-7.95400	40.94500	-68.35500	-6.24300	30.13700	-62.54600	-15.71000
C	45.84700	-68.63500	-6.92300	40.90400	-68.89400	-4.67000	30.38300	-63.69700	-14.41800
C	45.69300	-68.27600	-5.55700	44.49300	-64.61800	1.45700	34.49500	-62.17100	-7.56400
C	39.19400	-69.90900	-4.75400	38.42800	-68.55900	2.51900	27.60100	-64.97200	-8.11000
C	38.09000	-70.37800	-5.56500	42.30500	-69.11000	-3.79500	31.95200	-64.16900	-13.98900

C	37.32800	-71.42500	-4.97700	40.36700	-72.30400	0.40600	29.17700	-67.30600	-12.14000
C	37.67400	-72.07200	-3.76300	40.46400	-65.17100	-1.24900	29.71400	-61.54900	-11.13300
C	38.82900	-71.66800	-3.09700	45.51300	-71.26400	2.98900	32.82900	-69.20600	-9.08800
C	36.35700	-74.89500	-10.42500	46.69600	-69.53900	-0.85800	35.99700	-66.12800	-10.75300
C	41.63200	-69.33800	3.11800	43.62600	-58.37100	2.16000	37.40900	-54.45500	-7.85300
C	40.74900	-69.35000	4.26800	39.45800	-55.21800	-0.56600	35.42400	-51.96300	-9.07200
C	39.49400	-70.04500	4.21200	42.25100	-61.89400	-2.05800	33.15900	-59.45700	-12.01300
C	39.11500	-70.81000	3.04500	35.59300	-63.41900	0.89400	27.20900	-56.55200	-7.97600
C	39.93600	-70.72700	1.86000	36.98200	-60.76300	-2.45300	30.76100	-55.15100	-11.94500
C	37.23800	-70.92500	-12.62000	37.57200	-60.53400	-3.68300	30.09700	-56.22400	-12.54800
C	44.99500	-71.57300	-4.91400	40.57300	-59.37500	5.49300	34.34600	-56.81800	-3.80500
C	45.14900	-72.60700	-5.84500	41.91100	-59.11200	5.26700	35.63400	-57.37300	-3.66700
C	44.05100	-73.39500	-6.22200	35.29100	-57.70300	3.38800	31.35400	-53.75900	-4.60100
C	42.78400	-73.22100	-5.60500	35.56800	-56.50100	3.91900	30.61700	-52.66000	-4.68500
C	42.58800	-72.17100	-4.63500	40.02200	-65.50900	5.03000	30.14400	-62.58500	-5.63600
C	46.20600	-65.30100	1.02600	40.76000	-68.57000	6.15900	29.59000	-65.60500	-3.66000
C	46.34300	-64.35200	2.02300	43.05100	-66.70500	7.13100	31.99400	-64.56900	-2.49700
C	46.07500	-62.98700	1.76200	41.10000	-66.05700	4.43200	30.93700	-62.76500	-4.55200
C	45.39000	-62.66800	0.60800	37.92300	-65.01900	2.73000	28.44300	-60.49500	-7.33300
C	45.09700	-63.65600	-0.38500	37.83900	-63.57400	5.38600	29.18300	-59.85700	-4.56300
C	-7.00000	-62.03000	-10.98400	47.25500	-67.65000	3.73600	35.84700	-66.63700	-6.63300
C	-5.04800	-60.62800	-9.24800	46.90400	-68.43200	4.86200	35.12600	-67.51300	-5.74100
C	-5.80500	-59.06700	-13.24400	46.33900	-67.79500	5.98300	34.52100	-66.99100	-4.58200
C	-5.11600	-57.99800	-15.79700	46.15300	-66.39400	5.98400	34.75700	-65.58900	-4.27800
C	-6.76600	-56.18300	-14.37100	46.52900	-65.57800	4.87900	35.49100	-64.79100	-5.14800
C	-5.70600	-59.06700	-11.84200	53.42600	-61.96200	5.02200	42.46600	-65.65200	-3.19000
C	-7.94400	-65.78800	-9.21700	53.99400	-61.35600	6.13600	42.91100	-65.56600	-1.87100
C	-6.82400	-66.23000	-8.53500	53.56500	-60.05500	6.52200	43.55100	-64.44300	-1.29900
C	-6.94200	-63.92700	-13.92300	52.61200	-59.36100	5.71900	43.71400	-63.30700	-2.12600
C	-7.08900	-64.80900	-12.86000	52.19700	-59.92500	4.50000	43.30000	-63.35200	-3.50800
C	1.32300	-65.92600	-8.48500	55.78000	-61.54600	0.84000	46.94300	-64.30700	-5.44800
C	0.46200	-66.23100	-9.46000	52.51100	-67.58400	-0.21500	42.97800	-65.74000	-11.21200
C	-4.84400	-63.24300	-6.07300	53.20600	-68.52300	-0.97500	43.47800	-66.70400	-12.11700
C	1.01400	-63.67000	-13.72900	53.17300	-68.52200	-2.38500	42.74800	-67.11200	-13.25700
C	-2.96600	-69.92700	-8.51900	52.35700	-67.54900	-3.03800	41.47500	-66.50600	-13.47600
C	-3.91200	-67.34000	-15.30500	51.56900	-66.67100	-2.26900	40.88700	-65.64800	-12.58500
C	2.46400	-59.56000	-15.35300	56.72000	-57.57600	5.04000	46.78100	-63.19800	-0.09200
C	-0.00300	-54.44100	-15.00200	53.33800	-61.20600	-2.16300	43.92400	-62.50100	-10.24000
C	0.02400	-61.47800	-9.58800	53.74800	-60.65100	-3.38400	45.08800	-61.95000	-10.79200
C	1.30000	-55.17200	-9.58700	53.05200	-59.54100	-3.90600	46.02400	-61.33700	-9.96500
C	3.52700	-59.57700	-10.74500	51.95700	-58.95700	-3.22100	45.89100	-61.39700	-8.52600
C	-2.91300	-56.73300	-9.26200	51.43200	-59.69800	-2.09800	44.75500	-62.02600	-8.01500
C	-2.23900	-61.06300	-15.28000	47.31300	-62.17900	5.55300	38.58500	-62.64500	-2.65400
C	4.15900	-60.71600	-11.26400	46.10400	-61.88300	6.29800	39.18700	-62.01300	-1.52800
C	5.54600	-60.99600	-11.14700	45.40300	-60.64300	6.04400	39.21300	-60.57800	-1.37200

C	6.30300	-60.13000	-10.31900	45.76100	-59.86800	4.87400	38.92700	-59.83000	-2.48700
C	5.79700	-58.98700	-9.66200	46.86900	-60.26300	4.06100	38.54300	-60.44600	-3.70100
C	4.39700	-58.71500	-9.93200	46.16100	-65.50500	-1.82400	36.46100	-62.25700	-11.44500
C	-3.60400	-63.56200	-6.55600	45.63000	-66.10900	-2.99500	35.87100	-62.32400	-12.69100
C	-2.53900	-69.73400	-9.82500	46.50900	-66.56100	-4.04700	36.64100	-62.78300	-13.78000
C	0.98500	-54.44500	-16.02200	47.85300	-66.20200	-4.00600	37.99500	-63.17300	-13.64000
C	1.28900	-53.25800	-16.75700	48.41200	-65.39000	-3.00400	38.63600	-62.95300	-12.40300
C	0.54500	-52.10300	-16.40600	59.22600	-56.46400	1.40200	51.91100	-61.08100	-1.74100
C	-0.33700	-52.05500	-15.30700	53.40900	-55.88400	-2.70400	48.28300	-58.39100	-7.62900
C	-0.62300	-53.25100	-14.54600	52.40900	-67.41700	3.92900	40.61800	-68.88800	-6.44800
C	-1.48900	-62.24900	-15.58300	53.17200	-68.16400	4.92900	40.40000	-70.15000	-5.99700
C	-2.20300	-63.43200	-15.91700	54.36600	-67.62400	5.47700	39.11600	-70.71700	-6.04500
C	-3.65800	-63.50700	-15.74400	54.62700	-66.20300	5.27300	38.08200	-69.98000	-6.64500
C	-4.38600	-62.35500	-15.35800	53.87500	-65.52000	4.26600	38.29000	-68.69700	-7.18700
C	-3.67600	-61.08600	-15.23100	47.68900	-61.34900	4.43800	38.29300	-61.83700	-3.82100
C	-4.23800	-57.24900	-9.22900	52.25200	-60.69100	-1.42900	43.67600	-62.31800	-8.83600
C	-5.02400	-57.16300	-8.06500	52.82800	-66.17400	3.48300	39.63700	-68.24100	-7.21500
C	-4.39600	-56.63800	-6.87200	51.60600	-66.71700	-0.87800	41.62000	-65.34600	-11.37100
C	-3.05500	-56.22600	-6.91400	52.58400	-61.23800	4.08600	42.73000	-64.52100	-4.05800
C	-2.26400	-56.26300	-8.10900	47.49400	-65.05200	-1.85800	37.88500	-62.47500	-11.28400
C	-2.70900	-66.93200	-14.71600	47.12600	-66.21600	3.75700	35.96800	-65.26900	-6.37800
C	0.97100	-53.81800	-9.90800	57.91100	-58.15100	5.47900	47.09600	-62.82900	1.21900
C	1.75100	-52.73300	-9.37000	59.69200	-55.26700	0.88900	52.83400	-62.09600	-2.08800
C	2.88800	-53.00900	-8.55800	55.30900	-62.68900	1.53200	45.76000	-64.60300	-6.07000
C	3.42900	-54.33500	-8.61900	53.02300	-54.55000	-2.76800	47.00600	-58.11100	-8.07000
C	2.59700	-55.39700	-9.08200	56.96700	-58.74300	-2.32600	49.89200	-62.67500	-7.12000
C	0.22300	-64.73100	-13.47400	58.04800	-59.50800	-1.97300	51.23000	-62.49800	-7.62200
C	1.31500	-62.09100	-9.53200	53.73800	-55.76700	5.07600	45.27000	-58.89200	-1.01100
C	1.84900	-62.50600	-8.29000	52.39700	-55.61500	5.44700	45.42200	-57.86900	-0.10400
C	1.10500	-62.47600	-7.04600	55.63300	-52.68600	1.53900	50.71800	-57.20100	-3.32300
C	-0.22100	-61.94600	-7.16600	56.19400	-52.60500	2.80000	51.33400	-57.05100	-2.09600
C	-0.70600	-61.40300	-8.39100	48.18100	-58.37900	1.11500	41.52500	-59.30200	-5.68400
C	2.96700	-58.89700	-16.50900	47.72900	-59.27500	-2.70400	40.03800	-56.90700	-8.89500
C	3.57100	-59.59800	-17.56000	45.06100	-58.88600	-1.17000	38.45100	-57.99800	-6.86700
C	3.79400	-60.97200	-17.47400	48.11400	-58.38800	-0.26500	41.48900	-58.23600	-6.62400
C	3.15400	-61.69400	-16.41000	49.54300	-55.62400	1.79500	43.24800	-57.35800	-3.80900
C	2.35900	-60.97100	-15.44600	49.68200	-58.06100	3.61500	42.51600	-60.25900	-2.99700
C	-2.36500	-56.61200	-0.02200	51.96000	-59.49600	-12.80200	50.31100	-54.15400	-16.04100
C	-2.64500	-54.67600	-2.38400	52.12900	-59.68200	-14.20600	50.63100	-53.16600	-16.97100
C	-2.83800	-57.98800	-3.99500	53.21800	-59.08000	-14.87500	51.63300	-52.22400	-16.54100
C	-5.44100	-59.29900	-4.86100	54.08400	-58.25900	-14.13000	52.14300	-52.17200	-15.18400
C	-2.87400	-59.73900	-6.39300	53.80100	-58.00000	-12.74700	51.74900	-53.18200	-14.26400
C	-3.00000	-57.91300	-2.65500	49.21900	-60.92900	-5.08800	44.10000	-58.02700	-10.22000
C	2.43200	-49.36500	-4.42000	49.65700	-61.82900	-4.07300	42.87400	-58.74700	-10.10000
C	1.90100	-50.49800	-5.00700	50.57700	-62.85300	-4.40500	42.40200	-59.46200	-11.22100

C	-0.57800	-52.40700	0.69700	51.15300	-62.82700	-5.73700	43.17700	-59.47600	-12.43700
C	0.05700	-53.54200	0.15100	50.68800	-61.98900	-6.71100	44.52400	-58.97300	-12.36700
C	5.23500	-56.86400	-6.75900	48.79300	-62.02100	-10.95700	43.73200	-57.71200	-16.21300
C	5.61900	-55.66800	-6.21400	55.42200	-57.15500	-5.32500	49.84800	-55.66400	-6.94500
C	-0.68400	-53.60900	-6.54300	56.83400	-57.14300	-5.34700	51.11900	-55.97600	-6.39300
C	5.23200	-58.87900	-2.81100	57.53400	-56.11900	-5.96200	52.13200	-54.96300	-6.25600
C	7.07000	-50.93900	-3.10300	56.84000	-55.05100	-6.53100	51.80400	-53.67200	-6.73800
C	6.13500	-54.51100	1.22400	55.48600	-54.91900	-6.32100	50.66700	-53.42600	-7.56500
C	-0.66000	-64.25600	1.41000	44.19300	-64.09100	-12.06200	39.26200	-58.27700	-20.68500
C	-1.37200	-65.03100	-4.29000	48.37600	-55.24300	-4.46400	43.07000	-53.88500	-8.94900
C	1.67500	-58.00500	0.53500	48.05400	-54.43900	-3.36300	42.00600	-54.00100	-8.00200
C	3.43600	-63.41000	-4.47800	48.63300	-54.76200	-2.07800	42.10000	-54.82900	-6.83300
C	3.88100	-63.12700	0.38100	49.50100	-55.87200	-1.98800	43.32200	-55.56100	-6.65200
C	1.47900	-58.85600	-4.92600	49.87800	-56.62200	-3.14100	44.32200	-55.54700	-7.65400
C	-2.43600	-60.37800	1.54700	46.51200	-57.90100	-10.54000	46.86800	-54.10600	-16.74500
C	5.06000	-63.29600	-0.39200	45.57000	-57.12000	-11.23400	46.56300	-53.23900	-17.81700
C	5.99200	-64.30500	-0.00300	45.98600	-56.53300	-12.44400	45.22900	-52.79400	-17.98500
C	5.67800	-65.29900	0.97300	47.20500	-56.77400	-13.06700	44.25700	-53.17800	-17.02100
C	4.40800	-65.18400	1.62300	48.14200	-57.49200	-12.33900	44.59200	-53.99100	-15.94500
C	3.50200	-64.18300	1.24600	52.80000	-52.42900	-9.67500	52.02800	-52.09500	-11.23000
C	0.18900	-54.66400	-6.56200	53.35600	-51.40800	-10.51400	53.17700	-51.47000	-11.81800
C	6.31400	-51.68700	-4.01200	54.79000	-51.52100	-10.61700	53.08400	-50.08900	-12.19500
C	-0.61400	-65.23900	-5.40600	55.58300	-52.57700	-10.15400	51.92600	-49.38000	-12.03600
C	-0.47200	-66.54800	-5.97900	54.93000	-53.61400	-9.44400	50.82100	-49.92100	-11.41200
C	-1.08800	-67.63000	-5.34800	45.46300	-66.94600	-9.51200	37.39200	-61.56300	-17.62600
C	-1.93300	-67.41300	-4.20500	45.99000	-62.75400	-3.87200	38.37900	-58.82400	-12.03900
C	-2.08700	-66.07300	-3.69600	55.69000	-59.11000	-8.41000	49.83700	-59.18800	-10.78800
C	-3.82000	-60.44800	1.63100	57.00400	-59.42400	-7.98500	50.59900	-60.00500	-9.89000
C	-4.39000	-59.80500	2.76300	57.25300	-60.42700	-6.99300	51.89200	-59.55100	-9.41500
C	-3.67700	-58.93000	3.59200	56.16600	-61.14800	-6.43700	52.20000	-58.17200	-9.44600
C	-2.32100	-58.72600	3.29100	54.84700	-60.83200	-6.83900	51.41900	-57.33100	-10.29800
C	-1.69400	-59.39900	2.24200	47.82300	-58.11500	-11.09000	45.91800	-54.54500	-15.84800
C	0.44400	-58.31900	-5.77300	49.25800	-56.37000	-4.39500	44.13900	-54.72200	-8.82200
C	0.59100	-58.32100	-7.16900	54.61800	-59.96800	-7.96600	50.32500	-57.87300	-11.02900
C	1.72000	-58.95500	-7.80400	54.72500	-56.01400	-5.75200	49.71000	-54.39200	-7.59000
C	2.75600	-59.39300	-6.95100	49.69400	-61.00300	-6.43700	45.00800	-58.21000	-11.28300
C	2.68500	-59.28100	-5.56700	53.59400	-53.53200	-9.24300	50.82500	-51.29900	-11.07000
C	4.79000	-54.30200	1.10200	52.79900	-58.64700	-12.04100	50.81400	-54.17700	-14.72200
C	4.76500	-63.16500	-4.06500	43.81300	-63.41200	-13.21100	40.02900	-57.61500	-21.58400
C	5.77000	-64.12300	-4.37100	44.25600	-67.51400	-9.99600	36.42900	-61.51300	-18.63600
C	5.29200	-65.43400	-4.79600	48.87800	-61.18400	-12.09100	44.37600	-58.90100	-16.42100
C	4.01600	-65.63800	-5.26800	45.27400	-61.95400	-3.02200	38.04700	-57.88500	-11.08300
C	3.01200	-64.68100	-5.00000	48.76800	-65.02800	-8.23000	41.87500	-60.91600	-15.00000
C	4.61200	-58.07200	-1.84500	49.67400	-65.49400	-7.31100	41.64000	-62.28200	-15.10500
C	2.58800	-58.33200	1.56300	41.68300	-60.03000	-9.20200	38.33900	-54.21700	-18.23600

C	2.38500	-57.82400	2.86200	40.73200	-60.73700	-9.91100	37.72500	-53.15500	-17.52900
C	1.43100	-56.83000	3.13800	42.26900	-63.74400	-5.55500	35.16100	-59.00600	-15.37000
C	0.56400	-56.42000	2.09000	41.23500	-62.82700	-5.28600	34.03400	-58.37500	-14.89900
C	0.65700	-57.02800	0.80100	46.54400	-55.43900	-7.15700	42.36800	-52.97200	-12.70400
C	-1.17500	-63.50900	2.54900	46.26800	-52.05100	-8.50600	43.00100	-49.43200	-13.32900
C	-1.05700	-64.00900	3.81600	46.90300	-53.82200	-10.85300	44.93100	-50.44500	-15.10700
C	-0.38100	-65.27400	3.97100	46.00600	-54.93900	-8.32600	42.88700	-52.42000	-13.81800
C	0.30700	-65.93100	2.87700	45.79000	-57.50600	-4.85100	42.85200	-55.83600	-13.27700
C	0.28100	-65.29100	1.62600	43.84400	-56.97300	-6.94600	40.79200	-55.14200	-11.47700
C	-10.49000	-64.41800	0.70400	33.22100	-60.42000	6.43600	28.30500	-54.36600	-2.29700
C	-9.30600	-67.16300	0.84700	34.32800	-59.78700	7.05900	29.37800	-55.02900	-1.62100
C	-7.46900	-65.48000	3.02500	35.62400	-60.08700	6.68200	30.04600	-56.12200	-2.12900
C	-5.27600	-63.53900	3.97400	35.83900	-60.99600	5.59000	29.63500	-56.60200	-3.36700
C	-6.57200	-65.93000	5.63500	34.76700	-61.38600	4.76800	28.65900	-55.92800	-4.19500
C	-7.29900	-65.29800	1.63100	33.75400	-62.30800	-2.26300	26.24200	-52.88100	-11.17100
C	-14.91900	-63.51900	-4.16300	34.38200	-62.70800	-3.44100	26.74800	-53.65600	-12.29200
C	-13.63900	-62.92500	-4.01000	33.79900	-63.66000	-4.23900	26.63200	-55.05400	-12.28400
C	-8.54400	-60.06600	-3.46500	32.88100	-64.59000	-3.58700	25.82900	-55.72200	-11.37400
C	-8.19200	-61.37800	-3.23600	32.44200	-64.25500	-2.28900	25.23600	-54.94600	-10.29700
C	-11.17400	-69.82300	-6.66500	24.38500	-54.99000	-5.69100	19.64100	-46.08200	-11.90400
C	-9.98500	-69.18900	-6.41500	26.35700	-64.35300	3.86900	20.81200	-52.73400	-3.73500
C	-12.29900	-69.27500	-2.49000	25.50400	-65.36800	4.25700	19.70000	-53.32200	-3.18200
C	-5.60200	-68.05000	-5.85800	25.49200	-66.59000	3.55700	18.91900	-54.02400	-4.06800
C	-10.70100	-63.67500	-9.40400	26.04500	-66.60000	2.22200	19.12500	-54.04300	-5.46800
C	-8.64600	-61.38900	-7.25000	26.87000	-65.52300	1.83000	20.32300	-53.33600	-5.94700
C	0.33600	-68.35400	-1.23100	25.04400	-59.76400	-7.00400	17.83000	-49.86300	-11.80700
C	-0.85800	-69.68100	4.22800	28.09700	-63.08900	-2.42000	20.81600	-49.88600	-7.82700
C	-6.99300	-69.04500	-2.24500	27.83800	-62.74900	-3.75200	19.59900	-49.14000	-7.92400
C	-4.03300	-72.05200	3.34200	26.84500	-61.72000	-4.02700	19.74300	-47.75600	-7.82800
C	-2.77100	-71.51800	-2.72800	26.11200	-61.13700	-2.92500	21.02200	-47.08800	-7.71100
C	-7.69500	-69.61100	2.47800	26.49400	-61.49600	-1.62000	22.22300	-47.92500	-7.67700
C	-4.26800	-64.80900	-0.83200	33.97700	-58.24800	0.03400	28.75000	-52.20800	-9.40200
C	-3.92800	-72.40700	-3.54200	34.66100	-56.97100	-0.05600	29.70500	-51.87700	-10.32400
C	-3.71900	-72.74900	-5.22900	34.11800	-55.81600	0.41300	30.80500	-51.02600	-10.01400
C	-2.34800	-72.15300	-5.98900	33.01200	-55.82900	1.30700	30.86500	-50.51200	-8.69200
C	-1.21800	-71.26400	-5.15400	32.40100	-57.12500	1.57000	30.06600	-50.94300	-7.61000
C	-1.35600	-70.95800	-3.50900	27.33200	-61.64900	6.42700	24.07100	-51.40700	-0.74400
C	-11.94600	-68.11200	-1.79200	26.87700	-61.87300	7.74500	23.89400	-51.95000	0.56500
C	-11.50800	-64.62900	-8.85700	27.84400	-62.50800	8.61500	24.03500	-53.34300	0.85300
C	-1.40400	-70.76000	4.96800	29.14200	-62.93100	8.22900	24.45500	-54.23100	-0.14000
C	-0.85600	-71.09200	6.21500	29.53400	-62.62700	6.87600	24.65300	-53.75500	-1.50400
C	-0.00200	-70.15000	6.86200	29.17800	-58.64400	-10.58500	20.67100	-49.91100	-17.11200
C	0.33900	-68.90400	6.23300	31.31700	-53.28900	-6.85600	26.18200	-47.66200	-14.31100
C	-0.01500	-68.75100	4.83300	33.69100	-66.12800	2.60200	22.35000	-56.17100	-5.65700
C	-4.67400	-63.97000	0.19200	34.95000	-66.57300	3.19800	21.07500	-56.72800	-5.96000

C	-5.43600	-62.77600	-0.12300	35.20900	-66.31300	4.55600	20.70400	-56.87500	-7.32600
C	-5.79000	-62.45400	-1.45500	34.29900	-65.58200	5.29100	21.70300	-56.85100	-8.33500
C	-5.42900	-63.40500	-2.48200	33.11000	-65.04600	4.66000	22.94600	-56.27200	-7.98300
C	-4.66000	-64.56800	-2.17800	33.01700	-58.28200	1.05500	29.03200	-51.86100	-8.03900
C	-8.63500	-70.40200	1.83800	27.53800	-62.40600	-1.36000	22.08400	-49.33200	-7.94100
C	-9.73600	-70.75000	2.62100	32.78200	-65.35500	3.36300	23.31000	-56.05200	-6.62800
C	-9.88200	-70.39200	4.00700	27.04500	-64.37600	2.64600	21.09900	-52.61800	-5.06200
C	-8.95500	-69.53100	4.57500	32.95200	-63.16700	-1.54200	25.40800	-53.55100	-10.23000
C	-7.88400	-69.06500	3.78900	28.60000	-62.12500	5.87800	24.39900	-52.38100	-1.73100
C	-7.71800	-62.42200	-7.14800	33.43100	-61.21400	5.25600	27.90100	-54.92600	-3.54500
C	-3.31900	-73.22400	3.72800	25.37100	-61.08600	-7.26500	17.76000	-49.38100	-10.51300
C	-3.58500	-73.78200	4.96800	28.54700	-58.20600	-11.73000	19.65100	-50.75900	-17.56200
C	-4.78100	-73.44700	5.63500	24.30700	-53.77300	-6.38900	19.16400	-45.84400	-10.62400
C	-5.59500	-72.38100	5.18700	32.64900	-53.55700	-6.59200	26.20800	-46.84400	-15.45800
C	-5.21800	-71.67500	3.99800	28.16800	-54.04200	-9.97700	22.46700	-45.81800	-15.96600
C	-5.74300	-66.80700	-5.22000	27.08700	-54.22700	-10.75500	21.51200	-45.29500	-16.76200
C	-6.82700	-69.87600	-3.36000	29.60400	-59.33800	-3.29200	22.16300	-53.10700	-10.64200
C	-7.97500	-70.73200	-3.66400	30.52100	-60.43400	-3.33300	21.07800	-53.12300	-9.79500
C	-9.15500	-70.71800	-2.86900	32.14700	-59.05500	-7.69600	23.79900	-52.43000	-15.83900
C	-9.19300	-70.05500	-1.60000	32.60400	-58.52400	-8.85400	25.11600	-52.84600	-15.61800
C	-8.13700	-69.11000	-1.39700	29.44900	-55.10500	0.00300	26.26800	-47.58000	-8.36200
C	1.37500	-68.96600	-0.47100	26.95500	-56.07800	2.16200	27.29000	-45.76000	-5.16800
C	2.60000	-68.29300	-0.21900	29.50300	-56.74300	3.68000	28.93700	-48.26000	-5.34100
C	2.76000	-66.98200	-0.69600	29.35400	-56.44300	0.28000	27.51000	-47.54000	-7.80500
C	1.73900	-66.35200	-1.46800	30.41000	-53.08200	-2.39900	26.48300	-46.60200	-11.20300
C	0.58700	-67.07600	-1.73300	31.45500	-55.86500	-2.33100	26.76800	-49.73900	-10.56200
H	42.51300	-70.00500	-7.01700	45.97200	-71.08100	-2.12300	36.19400	-65.41500	-12.77600
H	43.12000	-68.56500	-6.40700	48.13900	-71.25700	-3.28300	37.23000	-67.68400	-13.54600
H	41.59000	-69.32000	-5.89500	50.11100	-70.08800	-2.40800	37.99100	-69.23700	-12.03200
H	40.78800	-67.53000	-10.42100	49.93100	-68.66100	-0.35800	37.40500	-69.15800	-9.69100
H	42.33200	-67.30100	-9.97300	47.53400	-67.75800	-0.14700	35.93100	-67.25600	-8.88000
H	42.11500	-68.86600	-10.37900	40.13000	-63.91200	0.46600	28.09100	-63.02400	-10.84300
H	41.26600	-65.30800	-7.76700	38.16900	-62.85800	-0.47700	26.38400	-61.28700	-11.35000
H	42.04300	-66.27800	-6.57700	37.34700	-63.50600	-2.81200	26.89500	-58.91000	-10.90700
H	41.95900	-63.04400	-5.70600	38.64800	-65.02300	-4.06900	29.21000	-58.25300	-11.32100
H	42.43100	-64.22500	-4.61200	40.75200	-66.15800	-3.09700	30.96300	-59.76500	-11.16000
H	41.06000	-62.95000	-4.23700	40.90100	-60.28500	-2.50200	34.55100	-59.77300	-10.41600
H	38.17000	-63.68500	-5.33700	40.12400	-61.68600	-2.08100	34.92400	-58.58100	-11.29600
H	37.95400	-64.82800	-6.36200	39.40800	-71.61200	-1.31400	28.53000	-68.91300	-10.99800
H	38.93200	-63.35500	-6.85300	38.80300	-73.89000	-2.19000	27.50800	-70.11600	-13.13000
H	40.06300	-67.57600	-6.23800	39.19900	-75.91900	-0.67000	27.67200	-68.98300	-15.22300
H	39.29100	-66.99600	-7.33700	40.48700	-75.57300	1.28700	29.03200	-67.05300	-15.52900
H	44.27500	-76.74100	-10.70000	40.98600	-73.37000	2.20800	30.03300	-65.91100	-13.47700
H	45.31000	-75.63600	-10.01100	43.31200	-57.35500	0.54300	37.34900	-56.24800	-8.89500
H	45.28400	-77.15500	-9.42400	42.71000	-56.46700	1.91700	36.78200	-55.14400	-9.68100

H	43.19800	-76.76200	-8.38900	37.60100	-69.09700	0.58800	28.13400	-65.35600	-6.02000
H	44.15300	-75.47000	-8.07100	35.81500	-70.60700	1.62200	25.93500	-66.36400	-5.46100
H	36.70600	-74.83400	-6.61300	35.44000	-70.53800	4.05500	24.39400	-67.01300	-7.32400
H	37.41700	-74.41400	-8.17200	37.19200	-69.49800	5.56700	24.76000	-66.09900	-9.55200
H	37.27300	-73.47100	-6.93800	38.93700	-68.28400	4.53800	26.79700	-64.80100	-10.06800
H	39.25300	-74.39800	-5.86100	45.05200	-63.52400	-0.33300	35.40000	-61.24300	-9.18900
H	39.21000	-75.67200	-6.93600	44.12200	-61.41500	0.52000	36.30200	-59.35500	-7.80700
H	42.54700	-73.03900	-15.71000	43.75000	-61.15700	2.89200	35.88500	-59.24000	-5.39800
H	43.19700	-71.73200	-15.00400	43.68600	-63.06800	4.39800	34.39200	-61.16200	-4.34400
H	42.59800	-71.76400	-16.47200	43.63100	-65.32300	3.29200	33.28000	-62.66200	-5.85700
H	40.83600	-70.53800	-14.80100	44.20500	-71.09500	4.58700	33.13000	-69.81400	-6.94600
H	40.21200	-71.95600	-15.40700	45.88300	-72.53200	6.16200	34.63700	-71.62700	-7.57200
H	45.24300	-70.51000	-11.33000	48.21800	-72.07100	5.65200	34.96300	-72.30900	-9.82900
H	45.98300	-70.61000	-9.91900	48.86500	-71.87600	3.35100	33.98500	-70.97400	-11.70900
H	45.66500	-71.92200	-10.76500	47.29800	-71.12000	1.68700	32.75900	-68.91500	-11.28200
H	36.80300	-72.73800	-13.65400	37.40200	-55.59300	-0.83400	33.48300	-51.32200	-9.37300
H	35.44700	-71.75400	-13.36600	38.27200	-56.52500	-1.86600	33.86300	-52.34600	-10.32800
H	36.09500	-72.57300	-12.05400	34.74800	-61.60000	0.30000	28.36200	-54.85700	-8.50000
H	39.73600	-77.78200	-13.14400	34.55400	-61.83700	1.87000	28.34200	-55.00400	-7.03300
H	40.13300	-77.62600	-14.74100	44.69300	-68.96900	-3.98500	34.25100	-63.51500	-14.17800
H	38.94800	-76.72700	-13.89100	44.91600	-68.09800	-6.58800	34.00800	-62.19900	-16.64200
H	35.33700	-76.82900	-10.30300	42.66400	-67.81500	-8.08400	31.59300	-61.38700	-17.43400
H	36.81200	-76.86500	-10.50000	40.02500	-68.02900	-6.62500	29.29400	-62.17000	-16.05200
H	36.34700	-76.49400	-9.03400	39.93500	-68.78700	-4.15100	29.58100	-63.93200	-13.78300
H	47.07400	-68.82000	0.40700	43.38100	-58.56600	3.17100	37.05900	-53.47900	-7.56400
H	48.12200	-69.11700	2.73000	44.59900	-57.99500	2.34900	38.33300	-54.11500	-8.28300
H	47.47100	-71.06200	3.83300	43.70000	-59.32400	1.68100	37.43800	-54.80700	-6.87200
H	44.90300	-71.65500	3.79800	39.37500	-54.62900	0.36100	35.76700	-51.54700	-8.13100
H	43.90400	-71.27500	1.47000	40.49700	-55.53200	-0.59300	36.01100	-52.86400	-9.13800
H	43.39900	-70.32200	-9.49100	39.71300	-54.58000	-1.33500	36.02700	-51.24100	-9.52900
H	44.08500	-71.65000	-8.74200	42.54000	-62.44300	-2.86600	33.32900	-60.41800	-12.44200
H	42.02400	-76.21300	-13.82300	42.55200	-62.62900	-1.35300	32.69900	-58.72300	-12.58800
H	40.76000	-75.47400	-14.52900	43.16000	-61.50900	-1.61900	32.26200	-59.92400	-11.73100
H	38.69300	-64.89100	-0.03200	34.85600	-64.20500	0.63600	27.30500	-57.20100	-7.11800
H	38.36600	-62.45900	0.19400	36.32200	-63.75800	0.15100	26.27300	-56.19700	-7.79800
H	39.69300	-61.19100	1.91900	36.02100	-64.08600	1.67500	27.20300	-57.10300	-8.90400
H	41.93400	-62.07800	2.47700	36.99500	-61.85400	-2.46200	30.21500	-54.25300	-11.83200
H	42.63700	-64.42200	1.60600	35.99800	-60.39000	-2.30000	31.50500	-54.61400	-12.54900
H	44.38800	-65.12600	-6.04800	37.90700	-59.54900	-3.85800	30.65400	-57.07200	-12.81200
H	45.00500	-65.82500	-8.46000	36.94400	-60.81000	-4.52900	29.73400	-55.90800	-13.50100
H	45.91200	-67.91600	-8.94000	38.50000	-61.13100	-3.94600	29.29100	-56.87400	-12.26200
H	46.60100	-69.36500	-7.18900	39.99600	-58.67300	6.14800	34.23800	-55.75200	-3.65000
H	45.96100	-68.86300	-4.68500	40.34900	-60.39000	5.86600	33.53500	-57.16100	-3.15300
H	39.57100	-68.89300	-4.94000	42.47400	-59.14400	6.17600	36.08300	-57.49700	-2.70200
H	37.79500	-69.92500	-6.50600	42.14300	-58.25400	4.67700	36.46300	-56.90000	-4.19900

H	36.34300	-71.64000	-5.33200	42.45200	-59.83100	4.68500	35.63000	-58.37900	-3.84900
H	36.99800	-72.80800	-3.27600	34.81600	-58.48200	4.00600	32.35300	-53.67700	-4.23000
H	39.13200	-72.38400	-2.39100	34.70000	-57.90200	2.49900	31.06100	-54.60000	-4.05600
H	35.74000	-74.11100	-10.00700	34.82200	-55.90600	4.39900	30.75100	-52.10000	-3.79700
H	35.98100	-74.79100	-11.36800	35.68800	-55.65200	3.20900	29.57900	-52.75500	-4.92900
H	42.38300	-68.57500	2.94100	36.14400	-56.46200	4.85900	30.97100	-51.88400	-5.35300
H	41.09400	-68.99200	5.18500	40.32000	-65.02300	5.96200	29.12900	-62.97400	-5.45500
H	38.82800	-70.12700	5.12000	39.30800	-66.21100	5.48200	30.46000	-63.12700	-6.47800
H	38.19500	-71.40500	2.98600	39.94200	-68.27300	6.78000	28.76700	-65.58000	-4.39900
H	39.52400	-71.10700	0.93500	40.21000	-69.06900	5.36800	29.75400	-66.58800	-3.32700
H	36.62200	-70.28600	-12.05800	41.25500	-69.21200	6.81200	29.18100	-65.07200	-2.77600
H	37.80700	-70.31100	-13.28700	42.49900	-66.24800	7.89100	31.12900	-64.39700	-1.84300
H	45.72800	-70.76400	-4.73900	43.84300	-66.02700	6.75400	32.82700	-63.90300	-2.70000
H	46.05100	-72.64500	-6.41400	43.72500	-67.53700	7.47600	32.16500	-65.59200	-2.18700
H	44.21000	-74.28600	-6.72600	41.84500	-65.36500	4.06200	30.66000	-62.23800	-3.65400
H	41.99800	-73.85500	-5.90400	41.00800	-66.72200	3.58500	31.95200	-62.45100	-4.50100
H	41.59000	-71.91700	-4.26500	37.18300	-65.71500	3.07200	27.49100	-60.67700	-6.98400
H	46.70400	-66.18000	1.25300	38.46700	-65.35200	1.85400	28.58600	-60.85600	-8.27400
H	46.46900	-64.73300	3.06700	37.22500	-64.35600	2.25700	28.24600	-59.45600	-7.69900
H	46.24800	-62.30300	2.50200	38.26200	-62.89900	6.07000	29.63900	-60.06500	-3.63000
H	44.73200	-61.83400	0.67000	37.12400	-62.77400	5.05000	29.17700	-58.80400	-4.77300
H	44.88600	-63.22500	-1.35600	37.25500	-64.26300	5.92500	28.16100	-60.06900	-4.46600
H	-6.71600	-62.63800	-10.12700	47.69600	-68.23200	2.97400	35.73300	-66.94700	-7.63000
H	-7.86900	-61.58800	-10.48600	47.20100	-69.45700	4.93700	34.61000	-68.37300	-6.17800
H	-7.42700	-62.29200	-11.88500	46.32900	-68.37300	6.87400	33.87800	-67.53900	-3.88500
H	-5.81000	-60.10300	-8.63900	45.46500	-65.98900	6.78300	34.34100	-65.13800	-3.37700
H	-4.70400	-61.60900	-8.94400	46.68300	-64.50500	5.05100	35.66500	-63.78900	-4.82800
H	-4.27100	-59.89400	-9.13800	53.79900	-62.89900	4.70600	41.64500	-66.24400	-3.58700
H	-5.26200	-59.77000	-13.79200	54.51200	-62.02700	6.80400	42.74100	-66.38400	-1.17500
H	-6.72900	-59.50500	-13.68000	54.13100	-59.62300	7.31100	44.08400	-64.49200	-0.35900
H	-4.91200	-57.19000	-16.44200	52.25200	-58.38500	6.10500	43.81200	-62.29500	-1.78900
H	-4.52400	-58.84700	-16.00100	51.56900	-59.46400	3.79400	43.24800	-62.51100	-4.10600
H	-6.01000	-58.40400	-16.17200	55.52500	-61.61200	-0.20100	47.79400	-64.26400	-6.14900
H	-7.03000	-55.88900	-13.38200	56.80600	-61.40200	1.01500	47.39400	-65.16500	-4.92600
H	-6.51600	-55.59700	-15.22900	52.69800	-67.53400	0.83100	43.17700	-65.88300	-10.21200
H	-7.61800	-56.72400	-14.73300	53.95400	-69.10200	-0.52000	44.53700	-66.95800	-12.08800
H	-4.88100	-58.43700	-11.53300	53.91300	-69.11900	-2.96800	43.07200	-68.03200	-13.71000
H	-6.40900	-58.44800	-11.29500	52.19600	-67.67500	-4.12800	41.15600	-66.50700	-14.47700
H	-8.71100	-65.46100	-8.59300	50.97600	-65.91600	-2.67100	39.93700	-65.18700	-12.91400
H	-7.88700	-64.88700	-9.84500	55.97100	-57.47000	5.78200	46.66900	-64.26300	-0.44700
H	-8.68000	-66.35700	-9.80200	56.84200	-56.53400	4.91700	45.76900	-62.98500	-0.16200
H	-6.48900	-65.68900	-7.66900	53.83700	-62.16900	-2.04100	43.16100	-62.85600	-10.91100
H	-7.04300	-67.13500	-7.96800	54.61700	-61.04800	-3.86800	45.07400	-61.76300	-11.87000
H	-6.41500	-64.39100	-14.73800	53.45700	-58.97100	-4.70500	46.99300	-60.99400	-10.28500
H	-7.85700	-63.56300	-14.31100	51.58900	-57.98800	-3.38600	46.56700	-60.79200	-7.93500

H	-6.62000	-62.95700	-13.59100	50.58300	-59.37000	-1.53900	44.50600	-61.90800	-6.99200
H	-7.51600	-65.78900	-13.15600	47.96300	-62.99200	5.77900	38.69500	-63.73200	-2.81000
H	-7.76800	-64.50200	-12.06200	45.79100	-62.52700	7.12400	38.96100	-62.52500	-0.60500
H	2.27800	-66.42800	-8.49500	44.40700	-60.47300	6.47800	39.48300	-60.19000	-0.43000
H	1.89100	-65.06200	-8.54900	45.05900	-59.05300	4.66300	39.21200	-58.82700	-2.51700
H	1.06500	-66.14800	-7.48000	46.80500	-60.16400	2.97700	38.40200	-59.86100	-4.54000
H	0.89700	-66.39200	-10.43900	45.44300	-64.98500	-1.16600	35.78600	-61.81200	-10.69300
H	0.15000	-67.22600	-9.19600	44.77700	-66.66400	-2.78600	34.80000	-62.39200	-12.70900
H	-5.11100	-62.91900	-5.08000	46.11800	-67.08600	-4.97000	36.14200	-62.89500	-14.75400
H	-5.49900	-64.08300	-6.06300	48.30800	-66.30100	-4.92600	38.34100	-63.78300	-14.43700
H	-5.34900	-62.32700	-6.39900	49.35000	-64.99200	-3.08300	39.52200	-63.49000	-12.21700
H	1.63800	-63.39400	-12.95100	59.55400	-57.45300	1.01400	51.60800	-60.82000	-0.76400
H	0.70600	-62.71900	-14.01200	59.44000	-56.61600	2.41500	52.26500	-60.14100	-2.00300
H	1.67200	-63.80500	-14.54800	52.71000	-56.58300	-3.19300	49.10600	-58.78400	-8.13600
H	-2.91900	-70.93700	-8.03900	54.43900	-56.04700	-3.11300	48.91100	-57.54500	-7.57500
H	-3.96000	-69.59700	-8.28400	51.48000	-67.84500	3.63900	41.56500	-68.52200	-6.37400
H	-2.42200	-69.63600	-7.63400	52.87800	-69.11900	5.24900	41.17000	-70.63900	-5.43000
H	-4.53800	-68.15900	-14.82800	55.17900	-68.20800	5.85100	38.94000	-71.79700	-5.84000
H	-3.85900	-67.57700	-16.36200	55.68500	-65.98200	5.13500	37.33400	-70.56700	-7.00700
H	-4.81200	-66.75600	-15.23100	54.26200	-64.61900	3.93300	37.62300	-68.25500	-7.85900
H	3.58000	-61.33500	-11.94600	58.79200	-58.35300	4.84500	46.96800	-61.78200	1.46000
H	5.90600	-61.71600	-11.85800	57.63600	-59.18100	5.58300	48.10000	-63.02000	1.64600
H	7.32600	-60.20000	-10.24100	58.37800	-57.84900	6.36800	46.69500	-63.42200	2.08500
H	6.44100	-58.39000	-9.02300	59.40300	-54.31700	1.27200	52.47300	-63.05200	-1.73500
H	3.93700	-57.89200	-9.44200	59.47800	-55.08200	-0.19100	53.85000	-62.13900	-1.83400
H	-3.25600	-64.38200	-5.91200	60.74100	-54.95200	0.99900	53.16500	-62.02700	-3.10100
H	-2.97900	-62.74100	-6.45400	54.18500	-62.79600	1.61400	45.28300	-63.77700	-6.59700
H	-1.59300	-70.20600	-10.14000	55.75900	-63.62300	1.26200	45.57200	-65.49000	-6.65300
H	-3.05400	-70.31500	-10.57000	55.50700	-62.81600	2.58800	44.87400	-64.84300	-5.50300
H	1.25900	-55.36500	-16.41800	53.49000	-53.80900	-2.25300	46.47100	-57.31500	-7.57400
H	2.17500	-53.22400	-17.42100	52.96700	-53.95300	-3.65600	46.92300	-57.53800	-9.00400
H	0.78900	-51.18400	-16.79700	52.09100	-54.39300	-2.26900	46.27900	-58.88300	-8.27700
H	-0.92900	-51.23500	-14.98200	57.06300	-57.93300	-3.01400	49.03800	-62.59400	-7.79600
H	-1.26100	-53.18900	-13.62100	56.31300	-59.37500	-2.88700	49.80300	-63.72600	-6.96800
H	-0.41100	-62.32100	-15.31800	58.80300	-59.13600	-1.33800	51.42000	-61.56000	-7.90200
H	-1.66200	-64.33100	-16.15600	58.58900	-59.71000	-2.87000	51.49900	-63.16600	-8.46000
H	-4.25500	-64.18900	-16.42000	57.86800	-60.49400	-1.54300	52.02800	-62.91400	-7.08000
H	-5.44800	-62.43700	-15.07000	54.21400	-56.67400	5.38500	44.50500	-58.63800	-1.75100
H	-4.14700	-60.12800	-15.31400	54.44600	-54.93100	5.34000	44.83100	-59.80700	-0.62100
H	-4.57200	-57.76500	-10.15200	52.28700	-55.35100	6.50100	45.98300	-57.08100	-0.53700
H	-6.08900	-56.94700	-8.03500	51.61700	-55.06100	4.84400	44.52800	-57.34100	0.32900
H	-4.86400	-56.15900	-6.04900	51.67500	-56.36800	5.22400	46.08900	-57.95400	0.78900
H	-2.48500	-56.18300	-5.99200	56.33500	-52.29500	0.79900	51.34700	-57.70600	-4.01300
H	-1.22900	-56.12700	-8.05800	54.83500	-52.03100	1.32900	50.25600	-56.37400	-3.82100
H	-2.20000	-66.01100	-15.14300	56.35700	-51.62300	3.25900	52.04400	-56.23600	-2.06500

H	-1.80300	-67.48200	-14.88600	55.54000	-52.92500	3.62000	50.74800	-56.33900	-1.56700
H	0.15400	-53.66100	-10.58500	57.08100	-53.12300	3.10900	51.89800	-57.88000	-1.59600
H	1.11800	-51.94700	-8.97500	48.25700	-59.39700	1.56200	41.41900	-60.27700	-6.14700
H	3.43900	-52.13900	-8.07300	47.34100	-57.90900	1.59800	40.62500	-59.44000	-5.12600
H	4.35600	-54.61400	-8.19300	48.72600	-59.56300	-2.51400	40.76400	-57.09000	-9.61600
H	3.04500	-56.38300	-9.00600	47.26800	-59.78600	-3.54900	39.05400	-56.88300	-9.44000
H	-0.32100	-65.15300	-14.29900	47.94400	-58.27400	-2.92100	40.12000	-56.01700	-8.33000
H	0.67800	-65.69500	-13.21200	44.51600	-59.62400	-1.72700	37.49100	-58.08100	-7.34600
H	1.88800	-62.14300	-10.44300	44.61000	-58.97900	-0.21600	38.45400	-58.67600	-6.04800
H	2.80000	-62.99000	-8.39100	44.97500	-57.90100	-1.66600	38.37900	-57.04500	-6.40700
H	1.42400	-63.08600	-6.20400	48.11100	-57.46400	-0.85900	41.27300	-57.29600	-6.21000
H	-0.91600	-62.17400	-6.39600	49.09400	-58.55800	-0.72200	42.28600	-58.01900	-7.30500
H	-1.68100	-60.98500	-8.46100	49.49000	-55.18700	0.84100	43.81100	-56.75700	-4.50400
H	3.01700	-57.84700	-16.66100	50.31200	-55.22900	2.37700	44.00200	-57.20000	-3.01000
H	4.19700	-59.00500	-18.25200	48.66700	-55.32200	2.17500	42.30000	-57.07000	-3.46400
H	3.92400	-61.44800	-18.43800	49.65000	-59.15400	3.57300	42.06300	-61.12300	-3.39900
H	3.45600	-62.72600	-16.26200	50.65800	-57.95400	4.01500	43.17100	-60.21400	-2.11000
H	1.99400	-61.44400	-14.57400	48.80400	-57.69600	4.13100	41.58100	-59.98800	-2.53300
H	-3.34600	-56.30300	0.40300	51.07000	-59.81200	-12.28900	49.92100	-55.09600	-16.39600
H	-1.64700	-56.06900	0.58900	51.27900	-60.15700	-14.80400	49.99400	-53.05700	-17.81900
H	-2.08600	-57.62200	0.28000	53.31700	-59.04700	-15.96500	52.08900	-51.58200	-17.23300
H	-3.66300	-54.56400	-2.33000	54.69500	-57.52400	-14.68200	53.01100	-51.64800	-14.91200
H	-2.03300	-54.07800	-1.72400	54.00200	-57.01200	-12.52300	52.33800	-53.22800	-13.33900
H	-2.54200	-54.40900	-3.40200	48.86500	-59.96000	-4.86300	44.32800	-57.42500	-9.40500
H	-1.79300	-57.99900	-4.30900	49.46800	-61.72700	-3.08900	42.35200	-58.74200	-9.10000
H	-3.13000	-57.07900	-4.58000	50.54500	-63.73300	-3.82300	41.45400	-59.94300	-11.05500
H	-5.88500	-60.16400	-5.18400	52.12700	-63.34700	-5.95500	42.65500	-59.78700	-13.32800
H	-5.91800	-58.65300	-5.58300	51.17100	-62.07600	-7.68100	45.15100	-59.28400	-13.12000
H	-5.77300	-59.38100	-3.88300	49.64200	-62.02100	-10.27500	44.14900	-56.81200	-16.61400
H	-3.02000	-58.88200	-6.93600	48.83200	-63.04100	-11.01700	43.58500	-57.29600	-15.23000
H	-3.35700	-60.57900	-6.79700	54.81400	-58.00500	-4.97100	49.07700	-56.44100	-6.92300
H	-1.82100	-59.80600	-6.44000	57.40200	-58.01500	-5.18400	51.26200	-56.98800	-6.04800
H	-4.00200	-57.92700	-2.15600	58.59300	-56.02100	-5.90500	52.68900	-55.01600	-5.32100
H	-2.67800	-58.82400	-2.11500	57.29100	-54.48400	-7.31200	52.43300	-52.82500	-6.52400
H	3.48200	-49.25800	-4.31200	54.91500	-54.27000	-6.91500	50.39400	-52.48400	-7.83100
H	2.04700	-49.21500	-3.45500	43.40500	-64.56100	-11.58900	38.19400	-58.08800	-20.75900
H	2.47000	-48.42400	-4.78800	44.80200	-64.99300	-12.08100	39.25800	-59.33300	-20.91200
H	0.92700	-50.43000	-5.40200	47.81700	-55.04500	-5.39100	42.97000	-53.32000	-9.85300
H	2.53400	-50.81600	-5.83100	47.13400	-53.93500	-3.32800	41.03600	-53.67200	-8.37500
H	-1.49000	-52.60100	1.30100	48.52300	-54.11800	-1.23900	41.38100	-54.72000	-6.08500
H	-1.34200	-52.01700	0.05400	49.91200	-55.87200	-1.01500	43.12000	-56.48200	-6.09700
H	0.03000	-51.72100	1.17000	50.57900	-57.49400	-3.14700	45.24100	-56.11700	-7.46200
H	0.63500	-54.08300	0.85900	46.47000	-57.87100	-9.44400	47.86500	-54.32700	-16.55800
H	-0.69500	-54.12800	-0.36500	44.53600	-57.01500	-10.96700	47.17400	-53.13000	-18.64800
H	5.62200	-57.32900	-7.64500	45.20300	-56.13700	-13.11200	45.18800	-51.79600	-18.39500

H	5.18700	-57.77300	-6.15100	47.22500	-57.08400	-14.08200	43.19100	-52.94500	-17.10300
H	4.24600	-56.78500	-7.07200	49.02600	-57.76800	-12.83300	44.11200	-53.86100	-15.00500
H	5.66400	-54.87400	-6.81500	51.78200	-52.18200	-9.52400	52.20100	-53.05200	-10.74500
H	6.51900	-55.67600	-5.62800	52.95300	-50.44800	-10.67700	54.09500	-51.96700	-11.66100
H	-0.19300	-52.68200	-6.65500	55.34900	-50.78000	-11.12800	53.78300	-49.66100	-12.84800
H	-1.33600	-53.51700	-5.67000	56.44200	-52.81500	-10.69000	51.83100	-48.41000	-12.43300
H	-1.34200	-53.54100	-7.30900	55.43500	-54.51300	-9.21500	49.91400	-49.33900	-11.18000
H	5.98700	-58.45300	-3.42400	46.26000	-66.59500	-10.19800	38.06000	-62.34900	-17.91100
H	4.42900	-59.03500	-3.53900	46.25900	-67.34500	-8.97700	37.10500	-61.81100	-16.60200
H	5.59100	-59.82800	-2.55100	47.01300	-62.88800	-3.52600	39.12200	-59.54800	-11.79300
H	8.03900	-50.57600	-3.30000	45.85000	-63.81200	-3.77800	37.76700	-59.71100	-12.17900
H	6.61800	-50.03400	-2.70400	55.44000	-58.54800	-9.25700	49.05000	-59.60300	-11.38900
H	7.57500	-51.48200	-2.37100	57.84100	-58.81000	-8.31700	50.31700	-61.01000	-9.73900
H	6.52400	-55.50100	1.53200	58.07400	-60.32600	-6.32300	52.65400	-60.23000	-9.08900
H	6.74700	-54.00900	0.45600	56.35000	-61.80000	-5.59500	53.14200	-57.87100	-9.04300
H	6.49400	-54.10500	2.10900	54.04900	-61.30400	-6.36600	51.77400	-56.43400	-10.72800
H	5.37500	-62.49300	-0.99600	42.92800	-63.75500	-13.74800	40.00400	-57.85100	-22.63800
H	7.05900	-64.25600	-0.19400	43.22100	-62.53600	-13.07600	39.95700	-56.56300	-21.72000
H	6.38300	-66.13900	1.02800	44.53000	-62.94400	-13.90900	41.07000	-57.69500	-21.48400
H	4.13300	-65.82800	2.41900	43.72100	-66.96400	-10.73400	36.70800	-61.29500	-19.58000
H	2.60900	-63.98600	1.86800	44.20300	-68.39500	-10.56100	35.82800	-62.38900	-18.83900
H	0.76200	-54.59900	-7.49400	43.55100	-67.89300	-9.32900	35.56500	-60.87700	-18.41800
H	-0.19200	-55.64400	-6.64600	49.23400	-60.15400	-12.09100	44.51200	-59.24100	-17.38600
H	6.92500	-52.53500	-4.49100	49.66000	-61.30000	-12.78600	45.47200	-58.87700	-16.23100
H	5.83000	-51.09800	-4.85700	47.99600	-61.16700	-12.77600	44.00700	-59.78700	-15.82900
H	-0.23900	-64.32000	-5.81700	44.20000	-61.99400	-3.02800	37.16800	-57.24500	-11.35200
H	0.07000	-66.58900	-6.90800	45.41200	-62.13600	-1.96800	37.74200	-58.07800	-10.10300
H	-0.93100	-68.63400	-5.71800	45.52300	-60.89700	-2.98900	38.72700	-57.17400	-10.76000
H	-2.37100	-68.26000	-3.69100	48.19500	-65.79900	-8.76200	42.57300	-60.68300	-15.77200
H	-2.56400	-65.77600	-2.76800	49.11100	-64.46500	-9.05300	42.23700	-60.63100	-14.02100
H	-4.27500	-61.32600	1.15900	50.42300	-66.08600	-7.71100	42.56500	-62.80500	-14.85900
H	-5.43800	-59.73500	3.08600	49.57100	-66.00200	-6.38100	41.35600	-62.81700	-16.00200
H	-4.22300	-58.21900	4.23400	50.19400	-64.67700	-6.73300	40.80600	-62.63900	-14.56000
H	-1.67400	-58.26100	3.97700	42.04600	-59.17900	-9.60800	38.10600	-54.25800	-19.28800
H	-0.80200	-59.01200	1.87400	41.30500	-59.77700	-8.28200	39.33800	-53.96200	-18.53200
H	-0.30600	-57.78600	-5.28900	41.08600	-61.15000	-10.82200	37.89400	-52.15600	-17.82300
H	-0.26100	-58.04500	-7.79900	39.74900	-60.32300	-9.77400	37.98800	-52.98800	-16.47800
H	1.45500	-59.46600	-8.68400	40.49400	-61.72300	-9.55200	36.66200	-53.29500	-17.23500
H	3.54000	-60.00100	-7.39900	41.82600	-64.67200	-5.69400	35.09300	-59.68400	-16.09800
H	3.61800	-59.38500	-5.06000	42.83500	-64.23800	-4.83200	35.57800	-59.80100	-14.78500
H	4.66300	-53.27700	1.34000	40.44900	-62.77500	-6.01400	33.55100	-57.56300	-15.49500
H	4.13600	-54.56400	1.79300	41.49900	-61.76800	-5.22500	34.06500	-57.97800	-13.95400
H	5.00900	-62.12600	-3.91300	40.50500	-63.05800	-4.58200	33.15100	-58.92600	-15.02400
H	6.89000	-63.96100	-4.20400	46.34200	-54.75700	-6.29000	41.57900	-52.42900	-12.15800
H	5.96100	-66.22000	-4.97700	47.63000	-55.55000	-7.10700	42.91300	-53.25600	-11.83000

H	3.73600	-66.46300	-5.86100	45.29500	-51.81600	-8.96200	42.25200	-49.26500	-14.07700
H	2.01800	-64.73000	-5.43100	46.73900	-51.14200	-8.76100	43.49900	-48.55200	-13.08000
H	3.92600	-58.49400	-1.13800	46.02500	-51.99500	-7.48100	42.44500	-49.51900	-12.39800
H	5.31700	-57.73600	-1.09300	45.97300	-53.60600	-11.37700	44.24100	-50.04500	-15.86200
H	3.15900	-59.19900	1.53300	47.29800	-54.75700	-11.18200	45.43800	-51.30000	-15.54600
H	2.98800	-58.12200	3.67200	47.39900	-53.01500	-11.32800	45.54700	-49.52800	-14.95200
H	1.39500	-56.58800	4.18400	44.97100	-54.76600	-8.49200	42.14600	-52.23200	-14.54700
H	-0.14600	-55.63000	2.29300	46.06700	-55.62900	-9.15400	43.38700	-53.17100	-14.39000
H	0.02300	-56.63200	0.01100	46.79100	-57.77500	-4.52000	43.28200	-55.56000	-14.25500
H	-1.37900	-62.47100	2.38100	45.18200	-58.36200	-4.75600	42.39700	-56.73000	-13.58100
H	-1.18200	-63.36100	4.66300	45.35200	-56.76200	-4.17600	43.54200	-56.06800	-12.38900
H	-0.25300	-65.61900	4.98000	43.55500	-56.71700	-7.97100	40.11500	-54.38900	-11.09900
H	0.98400	-66.65300	3.13700	43.43300	-56.01900	-6.60100	41.43300	-55.05600	-10.61300
H	0.52800	-65.96500	0.84600	43.32300	-57.84900	-6.74000	40.64200	-56.23300	-11.50900
H	-10.81100	-64.50100	1.72000	32.23900	-60.21500	6.93600	27.67800	-53.62400	-1.78700
H	-11.25000	-64.77200	0.06500	34.19200	-59.12500	7.89000	29.72000	-54.67600	-0.63500
H	-10.28100	-63.39400	0.43000	36.44200	-59.38100	6.93200	31.05700	-56.39400	-1.81900
H	-8.47900	-67.87100	0.80600	36.69500	-60.80900	4.98400	30.19900	-57.40000	-3.82700
H	-9.54800	-67.30800	1.86200	34.80900	-61.76700	3.76700	28.44300	-56.37700	-5.17200
H	-10.06000	-67.51400	0.11100	34.39300	-61.60400	-1.71400	26.43400	-51.87400	-10.94800
H	-8.12800	-64.88500	3.59600	35.34800	-62.37200	-3.73100	27.60900	-53.25600	-12.84400
H	-7.87000	-66.39300	3.33500	33.71500	-63.49300	-5.25800	26.93500	-55.62600	-13.20400
H	-4.37900	-63.29300	4.52400	32.46700	-65.37300	-4.20900	25.45200	-56.68300	-11.72800
H	-4.92800	-63.16300	3.03800	31.66000	-64.79800	-1.84500	24.61200	-55.42600	-9.55900
H	-6.01000	-62.81600	4.23000	24.17900	-54.93900	-4.65600	18.87000	-46.30000	-12.60800
H	-5.82000	-65.58400	6.38200	23.73400	-55.80300	-5.95500	19.89300	-45.20700	-12.48100
H	-6.73600	-66.92700	5.53300	26.35400	-63.34000	4.28100	21.45600	-52.23500	-3.02700
H	-7.39800	-65.39900	5.90100	24.70700	-65.32000	4.96700	19.41000	-53.03100	-2.27100
H	-6.79800	-64.38800	1.35000	24.69900	-67.34300	3.72600	18.03300	-54.45600	-3.70800
H	-6.63800	-65.91400	1.08800	25.66600	-67.29900	1.46300	18.52500	-54.63000	-6.20000
H	-15.17900	-63.77300	-5.14500	27.23700	-65.45000	0.83900	20.40400	-53.25600	-7.00900
H	-15.16600	-64.38300	-3.61300	24.45500	-59.15500	-7.67100	17.32500	-50.72700	-12.23500
H	-15.76900	-62.94200	-4.23300	24.46600	-59.66100	-6.15000	17.34600	-49.09700	-12.44700
H	-13.42800	-62.52100	-3.03100	28.43200	-64.08200	-2.20400	20.82500	-50.96200	-8.10100
H	-13.43000	-62.11200	-4.67400	28.29800	-63.29600	-4.54100	18.70000	-49.63500	-7.77700
H	-8.85200	-59.70000	-4.43800	26.88700	-61.17600	-4.95500	18.81600	-47.21600	-7.83200
H	-9.38400	-59.71500	-2.99400	25.33800	-60.37200	-3.10700	21.01400	-46.11700	-8.15100
H	-7.73300	-59.38100	-3.37200	25.86300	-61.18300	-0.80600	23.20500	-47.53900	-7.38900
H	-7.39000	-61.79600	-3.74200	34.38200	-59.09500	-0.42600	27.82400	-52.72700	-9.51200
H	-7.86000	-61.52500	-2.24900	35.51600	-56.99300	-0.73600	29.38300	-51.89300	-11.40200
H	-11.53500	-70.23000	-5.72000	34.78800	-54.98300	0.36700	31.40800	-50.72200	-10.86500
H	-11.90800	-69.24000	-7.16100	32.83100	-54.92800	1.84500	31.69900	-49.93600	-8.41900
H	-11.24500	-70.75200	-7.27100	31.56200	-57.20000	2.23200	30.46300	-51.08900	-6.57200
H	-9.23000	-69.77300	-5.88800	26.88200	-60.89000	5.80700	23.88000	-50.36000	-0.99100
H	-9.47100	-68.91300	-7.34500	25.97900	-61.39300	8.11600	23.93700	-51.23200	1.39800

H	-11.58800	-69.82000	-3.11500	27.54400	-62.52100	9.61200	23.98100	-53.79900	1.82900
H	-13.05600	-69.25300	-3.16200	29.83800	-62.95100	9.05500	25.16300	-54.94200	0.22500
H	-12.43700	-70.06900	-1.82100	30.46900	-62.90400	6.47900	25.12900	-54.36800	-2.27300
H	-4.62900	-68.47000	-5.72600	30.27800	-58.58500	-10.69400	20.78700	-48.93900	-17.46500
H	-6.10700	-68.88300	-5.49100	29.04000	-59.64700	-10.31500	21.67900	-50.09000	-17.39900
H	-6.08200	-68.12600	-6.81100	30.70700	-52.50600	-6.47000	27.09800	-48.29200	-14.23400
H	-10.67100	-63.63700	-10.55000	31.04800	-53.10000	-7.88800	26.31900	-47.06900	-13.37600
H	-9.63500	-63.75200	-9.20600	33.50800	-66.20600	1.57300	22.67200	-56.19900	-4.61400
H	-10.82200	-62.62400	-9.13900	35.57700	-67.17300	2.57400	20.43600	-57.02400	-5.15500
H	-9.03100	-60.96500	-6.37600	36.20000	-66.43700	5.08100	19.79800	-57.40000	-7.62800
H	-8.32700	-60.62500	-7.81900	34.51400	-65.53900	6.34700	21.43800	-56.74200	-9.32400
H	-9.64300	-61.62300	-7.61000	32.31700	-64.52600	5.19100	23.82800	-56.32100	-8.61600
H	-4.63000	-72.94300	-2.93900	25.84900	-61.56400	-6.44500	18.16500	-48.49200	-10.28200
H	-4.35700	-73.43800	-5.68200	25.77700	-61.41700	-8.17900	18.16100	-49.90200	-9.66700
H	-1.88500	-72.71800	-6.75100	24.59100	-61.72100	-7.20900	16.79500	-48.98300	-10.21900
H	-0.45700	-70.75700	-5.64300	28.43800	-57.26700	-12.13500	18.63400	-50.36900	-17.57800
H	-0.53600	-70.64900	-2.87800	28.88700	-58.68100	-12.61400	19.40600	-50.83900	-18.59500
H	-11.17700	-68.12400	-1.04200	27.45900	-58.29200	-11.70200	19.66500	-51.79300	-17.14600
H	-12.55500	-67.77000	-1.04400	24.62100	-53.84300	-7.41600	18.95100	-46.65600	-9.96100
H	-11.25300	-65.55000	-9.33600	23.41500	-53.22600	-6.26500	19.67000	-45.21200	-9.95200
H	-12.52600	-64.70800	-9.22200	25.04600	-53.08800	-6.06600	18.25800	-45.18100	-10.66600
H	-1.99800	-71.47600	4.39400	32.77900	-53.94300	-5.59700	26.10800	-47.27400	-16.43500
H	-0.73600	-72.08500	6.48400	33.43700	-52.86100	-6.96000	27.10600	-46.32600	-15.67800
H	-0.13500	-70.12900	7.90200	32.96800	-54.39400	-7.13600	25.39800	-46.15800	-15.63100
H	0.91700	-68.17200	6.70500	28.32300	-53.01100	-9.71200	22.75600	-45.19500	-15.13300
H	0.04700	-67.71000	4.51900	29.09500	-54.32100	-10.40000	23.39700	-46.13000	-16.38300
H	-4.24900	-64.12900	1.19900	27.05600	-53.71600	-11.66600	21.91800	-44.40000	-17.29600
H	-6.18100	-62.46300	0.61000	27.01700	-55.24600	-11.15800	21.11500	-45.91500	-17.49600
H	-6.57500	-61.75500	-1.73800	26.11500	-53.99100	-10.31000	20.63900	-44.93600	-16.31300
H	-6.00700	-63.39100	-3.39600	30.05600	-58.48200	-2.85000	22.26700	-53.83100	-11.43600
H	-4.25000	-65.14700	-2.97600	28.70300	-59.52700	-2.73400	23.16900	-53.43500	-10.30900
H	-8.46000	-70.99800	0.94100	30.06300	-61.33500	-3.76000	21.15400	-52.49600	-8.98200
H	-10.50300	-71.41600	2.30100	31.34100	-60.44700	-4.01500	20.08500	-53.03800	-10.22400
H	-10.76300	-70.48600	4.61300	30.75200	-61.10500	-2.47800	21.01400	-54.05900	-9.27500
H	-8.89600	-69.34500	5.62600	32.86900	-59.17200	-6.93100	23.03700	-53.23000	-15.74500
H	-7.26000	-68.40200	4.32100	31.77100	-60.08100	-7.72900	23.49200	-52.01000	-16.77800
H	-7.21600	-62.84800	-7.98500	32.97400	-57.55500	-8.85400	25.33500	-53.21400	-14.64700
H	-6.79500	-62.00100	-6.76900	31.95400	-58.13400	-9.60200	25.94700	-52.16700	-15.59200
H	-2.48400	-73.64500	3.17300	33.35900	-58.99400	-9.44700	25.48300	-53.51500	-16.38400
H	-3.03400	-74.65100	5.30700	30.24000	-54.65600	0.58500	25.66700	-46.69900	-8.16600
H	-4.93100	-73.65800	6.65300	28.54900	-54.60700	0.10000	25.68500	-48.27400	-7.83100
H	-6.53100	-72.18900	5.58800	27.17500	-55.07200	2.40900	28.12100	-45.21700	-5.55400
H	-5.75500	-70.75500	3.63900	26.38800	-56.08600	1.22900	26.57800	-45.24300	-5.81600
H	-5.46200	-66.00900	-5.91400	26.24900	-56.55300	2.90400	27.17700	-45.60500	-4.09300
H	-5.28900	-66.45300	-4.27600	28.87000	-57.16900	4.44200	29.02200	-48.40500	-4.27200

H	-5.98700	-69.90500	-4.06100	30.44900	-57.25100	3.82300	28.87500	-49.21800	-5.88500
H	-7.88300	-71.55900	-4.45200	29.47200	-55.78500	4.06900	29.70400	-47.61400	-5.55400
H	-10.01700	-71.12600	-3.26400	28.71000	-56.89800	-0.42600	27.98700	-48.47300	-8.00500
H	-9.97900	-70.09900	-0.91100	30.29400	-56.98200	0.15000	28.22500	-46.77400	-8.04300
H	-8.10400	-68.49000	-0.50900	30.62900	-52.98500	-3.50400	26.62600	-46.84800	-12.21300
H	1.17900	-70.01600	-0.24500	31.30000	-52.67800	-1.91000	27.51500	-46.31700	-10.98700
H	3.02200	-68.61100	0.70800	29.61500	-52.41000	-2.17000	25.99100	-45.70400	-10.99600
H	3.66300	-66.44200	-0.34100	31.41600	-56.91300	-2.05700	26.44700	-50.34000	-9.79300
H	1.79200	-65.38600	-1.99000	32.30200	-55.45600	-1.87100	27.83800	-49.77500	-10.58700
H	-0.24800	-66.46200	-2.07400	31.93700	-55.54200	-3.15800	26.47200	-50.18100	-11.50600

Table S2. Atomic coordinates of systems consisted of four molecules of Ph_7T_8-T_8Et_7 compound for clusters 4-6.

Atomic coordinates of systems consisted of four molecules of Ph_7T_8-T_8Et_7 compound									
	Cluster **4**			Cluster **5**			Cluster **6**		
Atom	x	y	z	x	y	z	x	y	z
Si	30.63600	-66.58800	-4.93800	24.94300	-51.83700	-3.49200	38.88000	-37.68600	-12.80900
Si	30.60000	-62.08400	-5.67500	24.06600	-53.85200	-1.68800	39.74800	-39.88700	-8.83400
Si	31.86600	-59.96300	-7.10600	22.31600	-51.84100	-0.18100	38.58200	-41.33900	-6.38200
Si	33.18000	-59.11900	-9.46100	22.57000	-52.45900	-5.41300	36.73000	-43.47300	-5.54000
Si	34.77400	-56.92200	-8.18800	21.67100	-54.52300	-3.35300	36.34100	-42.15300	-2.81300
Si	32.58800	-54.96700	-8.64700	19.87800	-52.61500	-1.90800	38.49900	-43.70100	-1.49100
Si	31.03400	-56.92000	-10.04100	20.82600	-50.60400	-3.92200	38.94600	-44.89200	-4.18000
Si	33.58700	-57.93300	-5.70900	23.21400	-49.80600	-2.13900	38.14800	-39.89300	-3.67000
Si	31.36900	-56.02800	-6.04400	43.49800	-55.81400	-10.03700	40.38200	-41.41700	-2.30600
Si	29.58500	-57.98100	-7.55000	46.43500	-55.88400	-9.64300	40.87800	-42.68300	-4.91000
Si	40.85400	-60.23500	-8.38400	46.09200	-53.86700	-7.34500	30.88400	-45.42200	-8.64800
Si	43.71300	-62.29700	-5.65100	42.94100	-58.10900	-8.32600	26.65000	-47.04500	-8.95600
Si	46.38000	-63.09100	-6.68300	46.01400	-58.20000	-7.76500	26.24100	-49.54100	-10.44400
Si	47.48900	-65.81400	-7.06400	45.77100	-56.21100	-5.42900	25.70000	-50.03700	-13.32500
Si	49.18400	-65.57600	-4.58800	42.85100	-56.20500	-5.87400	22.74200	-50.27000	-12.73900
Si	51.21700	-63.91100	-6.38300	43.17600	-53.90700	-7.72300	22.98000	-53.16000	-12.25500
Si	49.60600	-64.47900	-8.62500	35.92100	-64.30000	-2.72000	25.90800	-53.08700	-12.74800
Si	47.92900	-62.79000	-4.18700	33.98600	-65.76300	-4.57500	23.12800	-49.57500	-9.88500
Si	50.04700	-61.27200	-5.69500	34.59900	-64.07900	-6.82600	23.39200	-52.60600	-9.30700
Si	48.57800	-61.55800	-8.22100	38.21700	-66.11100	-3.49000	26.38400	-52.49600	-9.88500
Si	43.12000	-52.11200	-12.14100	36.26100	-67.61600	-5.40500	33.46700	-53.95600	0.41200
Si	39.90500	-55.20500	-12.39900	36.86800	-65.92500	-7.71900	34.59600	-51.32000	-3.04000
Si	40.01400	-57.44700	-14.41800	38.95900	-64.48100	-6.00800	35.14000	-52.39400	-5.80000
Si	41.29800	-58.85300	-16.65600	36.77100	-62.64500	-5.11800	33.37400	-53.36800	-8.20000
Si	38.93500	-58.50200	-18.40800	30.07800	-63.33900	-5.53400	35.83700	-54.66800	-9.48700
Si	37.40000	-60.71200	-17.30600	30.44800	-65.58600	-7.48800	36.66800	-52.10600	-10.76500
Si	39.81200	-61.18400	-15.52100	28.00300	-67.07100	-6.47300	34.17300	-50.85100	-9.47000
Si	37.74700	-56.80200	-16.10700	28.26200	-61.86800	-7.38700	37.53100	-53.74800	-7.15900

Si	36.20500	-59.03600	-15.04400	28.58800	-64.01100	-9.32100	38.25700	-51.18800	-8.44700
Si	38.47000	-59.82200	-13.13100	26.12900	-65.52200	-8.34300	35.87400	-49.87700	-7.27500
Si	26.03800	-49.17900	-5.58000	25.79000	-63.28700	-6.30200	48.26900	-44.90300	-0.16000
Si	24.14900	-50.90000	-9.21600	27.70400	-64.80800	-4.44400	46.61800	-48.71300	-1.76800
Si	21.38400	-51.88500	-9.97000	21.53700	-46.83000	-10.39600	48.04900	-49.62900	-4.20800
Si	18.80800	-50.12500	-10.73900	19.77400	-49.10000	-11.15500	50.75700	-50.90800	-4.32300
Si	17.34400	-52.71900	-10.31100	19.29800	-49.84000	-8.20300	50.56500	-50.73400	-7.22900
Si	17.86400	-53.14600	-13.25800	19.15800	-44.92400	-10.21600	49.46700	-53.60700	-7.32500
Si	19.65700	-50.69100	-13.44000	17.30400	-47.39400	-10.88700	49.61900	-53.55800	-4.27000
Si	19.76100	-54.39700	-9.60100	16.88100	-48.04900	-7.99900	47.88800	-49.55900	-7.15500
Si	20.17500	-54.95300	-12.52500	18.79900	-45.60300	-7.48100	46.68000	-52.45100	-7.21500
Si	21.98800	-52.52000	-12.84600	21.15100	-47.48100	-7.55600	46.81700	-52.40900	-4.14600
Si	31.54400	-66.63900	-7.81900	23.66800	-47.46600	-6.25300	37.89400	-40.27500	-13.84800
Si	28.95700	-65.54400	-8.99900	24.94400	-47.26000	-2.10000	40.49600	-41.60700	-13.18700
Si	29.57700	-67.22600	-11.50000	35.20800	-58.72800	-10.06300	40.50600	-43.08700	-15.93800
Si	31.05300	-64.91000	-12.68000	32.75600	-58.01600	-11.31100	38.67600	-45.25200	-14.73400
Si	30.47800	-63.14400	-10.31600	34.18100	-55.76300	-12.86300	38.96800	-43.95700	-12.09900
Si	32.09500	-68.23400	-10.09900	36.14800	-60.59000	-12.26400	38.00200	-41.45100	-16.56500
Si	33.68400	-65.82100	-11.37200	33.39900	-59.93700	-13.37900	36.21800	-43.75800	-15.42400
Si	33.11300	-64.11000	-8.96300	34.93100	-57.92300	-14.96700	36.24200	-42.54700	-12.66700
Si	39.88800	-63.06800	-9.00900	37.59300	-58.35600	-13.49100	31.18000	-46.46400	-11.26000
Si	42.05800	-65.22200	-9.24500	36.71500	-56.48100	-11.25300	29.98800	-48.56300	-13.09500
Si	39.92500	-66.93100	-10.81900	38.71700	-55.76300	-9.36000	32.20600	-48.06200	-15.07400
Si	39.22200	-68.29100	-8.29100	41.22500	-52.13900	-8.94800	30.80800	-45.84800	-16.40400
Si	41.05400	-66.41100	-6.78500	44.55600	-59.73600	-2.74300	28.52900	-46.22100	-14.51100
Si	37.88500	-64.74400	-10.52700	45.60100	-58.53200	-0.20100	33.59000	-45.98100	-13.20200
Si	37.08500	-66.06900	-7.98200	43.57000	-60.48700	1.20600	31.86000	-43.82600	-14.34000
Si	38.92700	-64.27900	-6.42700	46.58500	-61.86100	-2.59500	29.56000	-44.13200	-12.50800
Si	44.86000	-54.52300	-12.12000	47.70000	-60.67800	-0.10400	31.41200	-54.63800	-1.68500
Si	44.96300	-57.05600	-10.47600	45.64500	-62.49200	1.29000	29.98300	-51.96000	-1.77000
Si	47.82000	-57.29600	-11.25500	44.70900	-63.82900	-1.21800	27.41000	-53.68800	-1.90700
Si	47.16600	-58.83000	-13.75400	42.68800	-61.62200	-1.38100	27.71600	-53.63300	-4.95800
Si	44.19900	-58.44700	-13.04200	39.78400	-61.55300	-0.91800	30.41900	-52.28200	-4.75200
Si	47.72900	-54.68300	-13.03500	37.18200	-59.89600	-4.18500	28.83400	-56.24300	-1.70600
Si	47.16600	-56.25900	-15.43000	26.94800	-55.77300	-4.00700	29.02700	-56.29300	-4.75900
Si	44.17900	-55.90500	-14.74500	28.87800	-54.20900	-2.25700	31.78200	-54.85500	-4.62800
Si	25.39800	-52.08100	-4.60100	30.55000	-56.64100	-1.65400	48.66200	-44.09000	-2.97700
Si	22.69100	-53.15000	-5.61600	28.81500	-55.06900	-6.26100	49.11500	-45.52800	-5.66400
Si	22.80000	-55.03100	-3.31400	30.73100	-53.41700	-4.57000	49.20300	-42.63400	-6.94000
Si	24.24800	-57.13400	-4.87100	32.30200	-56.01600	-4.05700	46.23200	-42.65500	-7.39200
Si	24.23600	-55.20600	-7.22200	30.51300	-57.45600	-6.02900	46.09500	-45.43900	-6.10300
Si	25.37000	-53.92500	-2.16400	28.65500	-58.17000	-3.60400	48.87300	-41.32900	-4.29500
Si	26.84700	-56.03700	-3.85600	26.95600	-60.17900	-2.61400	45.92200	-41.39800	-4.70500
Si	26.93100	-54.18600	-6.07300	25.97700	-64.51900	-2.05400	45.50300	-44.03800	-3.43800
O	31.66500	-66.75300	-6.18000	20.80800	-47.85300	-11.46200	37.80200	-38.73200	-13.42300

O	32.39000	-67.74900	-8.58600	19.98700	-49.58300	-9.64800	37.42200	-40.58900	-15.38200
O	30.00500	-66.62200	-8.32000	18.14900	-45.98400	-10.91800	39.47500	-40.55500	-13.81400
O	32.15900	-65.15200	-8.11500	16.52900	-47.46300	-9.45500	37.14100	-41.22400	-12.69200
O	30.51200	-68.17600	-10.55800	18.42600	-44.74300	-8.78500	39.54800	-41.89400	-16.49600
O	32.05400	-63.26400	-9.94300	17.91000	-46.99100	-7.38500	37.37600	-43.72200	-12.37400
O	32.52000	-64.87200	-12.06600	18.18900	-48.72700	-11.06500	37.06800	-44.91900	-14.77700
O	28.84700	-66.16700	-10.51000	20.70100	-45.46300	-10.22900	40.91000	-42.71000	-14.38300
O	29.72100	-64.10300	-9.21300	17.65500	-49.48300	-8.16900	39.73600	-42.49600	-12.10500
O	30.52600	-66.48400	-12.60300	21.65300	-47.65700	-9.03700	39.55500	-44.43400	-15.86300
O	30.18800	-63.98800	-11.64300	20.40500	-46.04000	-7.47200	39.49000	-44.96600	-13.29700
O	32.71800	-67.19000	-11.19400	20.12000	-48.67200	-7.23600	37.10900	-42.86500	-16.48600
O	33.85900	-65.21200	-9.93100	22.08100	-47.27600	-6.24500	35.75200	-42.88300	-14.20700
O	31.87500	-61.45600	-6.42300	24.88400	-53.33600	-2.96100	38.64300	-40.62400	-7.90300
O	33.21000	-59.18800	-6.59300	23.61300	-52.82200	-0.49900	37.85500	-40.39900	-5.21600
O	32.02500	-60.04400	-8.72200	22.55000	-53.82300	-4.56200	37.96100	-42.78000	-6.36500
O	30.63700	-58.95500	-6.68800	20.24100	-53.80400	-2.98400	40.02400	-41.47200	-5.69000
O	34.42200	-56.98000	-6.64200	21.16000	-51.67000	-5.04500	37.13900	-40.71900	-2.71000
O	30.16800	-57.84200	-9.08100	20.28400	-51.19700	-2.54800	39.93600	-43.98700	-5.11400
O	32.36700	-55.22500	-7.07400	22.70300	-54.65400	-2.10700	39.20900	-42.24600	-1.54100
O	34.51800	-58.43300	-8.71600	23.77500	-51.65700	-4.62000	36.08200	-42.54600	-4.37600
O	32.28800	-57.90200	-10.17800	21.05200	-52.70500	-0.77600	37.43000	-44.51100	-4.50200
O	33.83300	-55.90300	-8.95700	24.49500	-50.86400	-2.24700	36.95400	-43.39800	-1.97600
O	31.22600	-55.52600	-9.24900	22.24600	-49.92700	-3.44500	39.22900	-44.48300	-2.65000
O	32.26400	-57.15300	-5.24100	22.23800	-50.42700	-0.98300	39.68200	-40.24300	-3.15300
O	30.08100	-56.61000	-6.84400	23.66000	-48.23700	-1.89300	41.17500	-42.44600	-3.32300
O	40.58200	-61.62600	-9.24100	33.82100	-57.80700	-10.11400	31.33500	-46.51700	-9.66600
O	39.13600	-63.75700	-10.28400	33.15100	-56.94500	-12.43600	32.52400	-46.54200	-12.14400
O	41.26800	-63.90700	-8.73300	34.84600	-60.63800	-13.23300	30.12900	-47.50300	-11.91300
O	38.88000	-63.38900	-7.76300	33.67600	-58.80100	-14.50800	30.68100	-44.95800	-11.57200
O	38.51200	-66.18000	-10.82500	37.36200	-59.52000	-12.44400	33.25400	-46.87100	-14.51100
O	40.39700	-64.93400	-6.49200	36.44900	-58.57300	-14.65600	28.50100	-45.13800	-13.22200
O	37.88800	-67.40400	-8.42600	32.66900	-59.44000	-12.07800	31.82200	-44.77700	-15.69200
O	41.12300	-65.83200	-10.46800	35.28200	-60.04700	-11.02400	31.48900	-48.65500	-13.74500
O	42.01900	-66.30700	-8.03900	34.98900	-56.54700	-14.07400	29.13600	-47.69000	-14.17600
O	39.98900	-68.00600	-9.61700	36.56300	-57.81500	-10.36100	31.24300	-47.46300	-16.22600
O	39.81200	-67.41800	-7.07500	37.23400	-57.01700	-12.71200	29.41000	-45.52800	-15.62800
O	36.91700	-65.00200	-9.21200	35.26700	-55.94000	-11.66800	33.02800	-44.54200	-13.42400
O	37.80500	-65.45500	-6.60800	37.83400	-55.44500	-10.70900	30.41100	-43.49400	-13.70700
O	44.83100	-62.54600	-6.76100	45.02500	-55.36000	-10.13600	27.08700	-48.41900	-9.76700
O	46.69600	-63.34900	-5.12800	46.81700	-55.01200	-8.33000	24.63900	-49.21000	-10.37000
O	46.72500	-64.42900	-7.53100	44.40100	-58.38800	-7.61900	26.59800	-49.84800	-11.97900
O	47.38700	-61.96500	-7.19900	46.43400	-57.13400	-6.61800	26.32700	-50.90700	-9.63900
O	48.97500	-64.01500	-4.19700	42.40500	-57.02400	-7.25700	22.24500	-50.03100	-11.22700
O	48.73900	-63.07100	-8.84900	44.19800	-56.71400	-5.15500	26.73500	-52.55400	-11.45700
O	50.93000	-62.58000	-5.51100	46.18700	-57.36300	-9.11500	22.60200	-52.76700	-10.70900

O	47.94400	-65.98900	-5.53100	43.21300	-57.41300	-9.83300	24.12500	-49.54600	-13.12200
O	48.84700	-65.71000	-7.94000	45.82300	-54.60300	-5.90200	25.57500	-51.63800	-13.39300
O	50.24600	-65.09000	-5.73700	42.77300	-55.04700	-8.84700	23.05600	-51.78100	-13.07900
O	50.83000	-63.81900	-7.92000	43.08000	-54.69600	-6.28500	24.49500	-53.79200	-12.32100
O	48.61900	-61.49700	-4.88200	44.68000	-53.54300	-8.07300	23.35100	-50.97200	-9.08700
O	49.80500	-61.08300	-7.32500	42.12700	-52.63900	-7.68500	24.92000	-53.15900	-9.42900
O	44.21600	-53.13200	-11.58300	45.48300	-58.80100	-1.80400	32.68600	-54.87600	-0.71600
O	46.19000	-54.27000	-12.98800	44.18400	-59.11300	0.47300	30.45800	-55.98000	-1.80500
O	45.19200	-55.46700	-10.82800	47.35900	-60.76800	-1.67200	30.55300	-53.38400	-1.07400
O	43.98700	-55.36800	-13.22200	46.90300	-62.00400	0.44500	31.98200	-54.23400	-3.12800
O	47.76100	-55.76000	-11.83500	46.10600	-63.17300	-1.81300	28.22000	-54.85900	-1.14300
O	44.06700	-57.52600	-14.38600	44.76600	-63.48600	0.37700	31.03700	-53.61700	-5.33300
O	46.80100	-57.77400	-14.95100	46.75400	-59.47800	0.44300	28.50900	-54.92600	-5.43800
O	46.37800	-57.80500	-10.61900	45.37400	-61.07000	-3.30800	28.45600	-52.47100	-2.12900
O	44.00900	-57.61600	-11.67300	44.89300	-61.19200	1.78700	30.80300	-51.74700	-3.20100
O	48.02300	-58.04700	-12.64500	43.30100	-60.18800	-1.79200	27.25700	-54.15600	-3.47300
O	45.75200	-59.01500	-13.01400	43.51500	-62.93700	-1.87500	28.84100	-52.47800	-4.74700
O	48.12200	-55.56400	-14.35700	42.89200	-61.57400	0.22300	28.38600	-56.63200	-3.23000
O	45.69500	-55.52200	-15.20400	41.12600	-61.65200	-1.77100	30.63800	-56.06100	-4.57600
O	40.57700	-56.34200	-13.38800	34.94800	-65.44800	-3.28100	34.84000	-52.38300	-4.19500
O	38.50200	-56.83100	-14.67400	33.66300	-64.54200	-5.55500	36.63500	-52.76400	-6.19800
O	40.77900	-57.59200	-15.84500	37.01300	-67.09400	-4.05800	34.18500	-53.32000	-6.74500
O	39.69200	-58.81500	-13.58600	36.97500	-67.27000	-6.85600	35.04500	-50.88400	-6.36300
O	38.62300	-57.30300	-17.37500	38.74200	-65.75300	-5.03200	36.27400	-54.41800	-7.94100
O	38.95700	-60.98600	-14.17000	37.95000	-64.78700	-7.24400	35.07800	-49.77200	-8.69400
O	36.49500	-60.41300	-15.95300	34.74500	-67.02600	-5.27700	37.56200	-51.01300	-9.90600
O	40.33900	-59.19600	-17.89300	37.43300	-64.97000	-2.67100	34.26400	-54.27400	-9.28900
O	40.94200	-60.07700	-15.68500	35.39200	-65.38500	-7.33400	33.26400	-51.74400	-8.38800
O	37.58400	-59.38100	-18.21400	35.89900	-63.12200	-3.85800	36.80400	-53.70200	-10.40300
O	38.84200	-61.26700	-16.80800	38.30300	-63.30300	-5.11000	35.17600	-51.89900	-10.27600
O	36.51200	-57.82100	-16.03000	35.96900	-63.25700	-6.37200	38.29000	-52.76700	-8.19600
O	37.05700	-59.13900	-13.69200	37.06200	-61.06600	-5.29500	37.41700	-50.32500	-7.34300
O	25.36900	-50.47700	-4.78200	27.63500	-54.53700	-3.24100	49.17700	-44.40400	-1.45400
O	24.98300	-52.71800	-3.16600	29.49100	-55.43900	-1.36100	49.38900	-42.70700	-3.61800
O	24.20400	-52.49100	-5.57500	29.40400	-53.76900	-5.50000	48.69900	-45.28300	-4.11400
O	26.68800	-52.89900	-5.06500	31.71400	-54.68300	-4.72400	47.02900	-43.83700	-2.84300
O	24.11000	-54.95800	-2.35000	29.98200	-56.04900	-6.65300	49.07400	-41.39800	-5.89400
O	25.52300	-54.29900	-6.87600	31.75500	-57.15500	-5.02700	45.91000	-45.15500	-4.52600
O	25.47100	-56.83800	-3.83700	30.13600	-53.52500	-3.02700	46.41300	-41.50200	-6.25300
O	22.57700	-53.61900	-4.05400	27.49700	-55.63500	-5.53300	49.53600	-44.00400	-6.08500
O	22.90100	-54.36000	-6.68400	31.63100	-55.93700	-2.60300	47.68300	-45.66900	-6.41300
O	23.02300	-56.19900	-4.31300	27.59900	-57.05800	-3.25800	47.76800	-43.02300	-7.59600
O	24.63200	-56.52800	-6.34400	29.29500	-57.89900	-5.01400	45.51000	-44.00900	-6.73200
O	26.56400	-54.65200	-3.03100	29.94000	-57.75400	-2.65700	47.31100	-41.21900	-3.82600
O	27.23700	-55.60200	-5.38600	28.28800	-59.74500	-3.42400	45.12500	-42.75200	-4.31700

O	22.52300	-51.10500	-9.06900	30.57300	-64.76100	-6.09500	47.76900	-48.68200	-2.94700
O	20.35400	-52.86200	-9.28200	29.21100	-66.63900	-7.48200	48.22500	-49.06200	-5.69400
O	20.25500	-50.78200	-10.52500	28.00000	-62.65000	-8.75400	49.38400	-50.42000	-3.61700
O	22.00100	-52.64900	-11.26200	27.42800	-65.14900	-9.24500	46.85700	-50.77700	-4.19500
O	18.21700	-54.09500	-10.08900	27.04700	-62.28500	-6.38600	49.16700	-50.16900	-7.93400
O	21.06500	-51.16300	-12.91100	25.67300	-64.08200	-7.71700	48.28000	-52.92400	-3.60500
O	18.71300	-54.37100	-12.72700	29.84600	-64.43600	-8.47300	48.10800	-52.84500	-7.92300
O	18.02300	-51.32500	-9.93900	29.70200	-62.39300	-6.78700	50.82300	-50.11700	-5.75400
O	18.64600	-50.29200	-12.33700	26.62100	-66.42400	-7.08100	50.81100	-52.48200	-4.62200
O	17.19700	-52.49800	-11.93300	28.79600	-63.62900	-4.60400	50.60200	-52.38300	-7.30200
O	19.05400	-52.16800	-13.82700	26.31200	-64.42600	-5.24400	49.28200	-54.14600	-5.76900
O	20.55200	-55.00600	-10.87400	28.27300	-66.22800	-5.06600	46.90800	-50.82900	-6.97800
O	21.13100	-53.84600	-13.23200	27.33000	-64.94500	-2.83000	46.82500	-53.07500	-5.66400
C	35.61200	-66.58600	-13.33100	24.06000	-49.27800	-6.48000	34.98500	-43.72600	-18.00700
C	36.63600	-67.39400	-13.97300	24.54000	-46.50300	-7.64500	34.64000	-44.31800	-19.23600
C	37.07400	-68.44500	-13.18700	25.18900	-47.39500	-3.90900	34.69500	-45.73500	-19.26000
C	36.68600	-68.68900	-11.78500	26.32600	-48.28000	-1.29600	35.09700	-46.54300	-18.18400
C	35.68200	-67.85900	-11.20800	24.51400	-45.60700	-1.41000	35.32600	-45.95900	-16.96100
C	28.57200	-61.12400	-9.95300	24.05100	-46.86700	-4.51900	40.35700	-45.64600	-10.42400
C	27.62800	-60.15800	-10.42700	19.84700	-50.72700	-13.35300	40.14500	-46.52300	-9.33900
C	27.81600	-59.72300	-11.74400	19.61200	-50.81200	-11.94800	39.34300	-46.27900	-8.22400
C	28.95300	-60.10200	-12.50200	19.65700	-52.38100	-8.87400	38.68200	-45.00900	-8.18800
C	29.90000	-61.00800	-12.03200	19.90500	-51.52100	-7.81600	38.77000	-44.14800	-9.26600
C	33.52300	-59.03500	-11.26000	19.57500	-42.34300	-11.32800	35.21500	-43.21600	-6.47200
C	29.49300	-69.75700	-12.59000	18.59500	-43.30400	-11.16600	42.31400	-41.19600	-16.84400
C	28.80000	-70.94500	-12.65100	24.20200	-46.58000	-11.31800	43.61800	-40.99800	-17.42500
C	27.41900	-70.90900	-12.78100	16.65700	-44.51200	-6.31200	44.20100	-41.92900	-18.31700
C	26.72000	-69.68200	-12.67800	15.59400	-48.43300	-12.56400	43.46300	-43.01700	-18.76300
C	27.42600	-68.44800	-12.71600	14.56700	-48.19700	-6.64400	42.20300	-43.26800	-18.17800
C	36.42400	-56.05600	-7.85100	25.19300	-55.04100	-0.76600	34.64900	-41.33500	-2.37800
C	27.07200	-64.86700	-7.11000	22.13000	-51.33000	1.54700	42.45600	-39.67200	-12.71500
C	25.69900	-64.54700	-6.82600	22.85800	-53.12700	-7.12500	43.73800	-39.16500	-13.13300
C	24.64100	-65.04000	-7.60000	18.09700	-52.53800	-1.42700	44.75400	-40.11400	-13.47000
C	24.98700	-65.83300	-8.72500	20.94100	-56.24800	-3.85200	44.63200	-41.49100	-13.26900
C	26.35000	-66.20100	-8.93900	19.48200	-49.42600	-4.31900	43.30200	-41.96300	-13.09400
C	35.22300	-62.21900	-8.83000	26.49300	-51.40400	-4.46200	34.11600	-42.22600	-10.92000
C	36.08800	-61.34100	-8.15400	21.01200	-56.50700	-5.25900	33.53600	-41.31800	-9.97600
C	36.01200	-61.45500	-6.71800	19.85000	-57.00000	-5.87600	34.39700	-40.60900	-9.09300
C	35.37500	-62.60000	-6.11000	18.66300	-57.31500	-5.13300	35.83300	-40.79500	-9.17300
C	34.71000	-63.57000	-6.87300	18.66000	-57.16000	-3.68500	36.41700	-41.76600	-10.06900
C	33.39000	-70.51200	-9.11900	19.85100	-56.69000	-3.04900	37.57400	-40.23600	-19.08300
C	34.40800	-71.50500	-9.18600	23.14200	-45.90900	-10.73000	38.15700	-40.06200	-20.36900
C	34.73900	-71.86800	-10.51600	16.60600	-47.49100	-12.62100	39.51700	-39.69900	-20.46800
C	34.11200	-71.50400	-11.75000	20.83800	-51.22700	2.07800	40.25700	-39.43900	-19.27800
C	33.02200	-70.57600	-11.51200	20.65800	-51.30200	3.52100	39.71100	-39.65200	-17.98500

C	33.10100	-53.21900	-8.14200	21.62800	-51.82500	4.34000	39.07600	-44.86400	-0.05600
C	27.72000	-57.73800	-7.41300	22.88200	-52.11100	3.73700	42.31100	-43.44600	-5.89500
C	30.53800	-65.08900	-15.70800	23.18400	-51.77500	2.39900	38.79400	-47.43200	-17.03400
C	31.31400	-64.94500	-17.26500	27.08100	-50.15600	-4.13800	39.49500	-48.89500	-17.68200
C	32.62700	-63.81200	-17.48000	27.91800	-49.60000	-5.10600	40.28700	-50.01400	-16.64900
C	33.14600	-62.85300	-16.16700	28.06400	-50.27900	-6.39800	40.41900	-49.64100	-14.99300
C	32.42500	-62.99700	-14.62800	27.37200	-51.48300	-6.73700	39.82400	-48.22700	-14.33200
C	34.68700	-63.37900	-8.25600	26.50800	-51.98100	-5.75500	35.53200	-42.43400	-10.90500
C	27.36400	-65.88600	-8.07000	18.16800	-49.94100	-4.13700	42.24800	-41.09300	-12.74400
C	31.07300	-64.10200	-14.39500	17.06500	-49.07800	-3.91900	38.95800	-47.06400	-15.36700
C	28.79700	-68.51100	-12.65600	17.36800	-47.70100	-3.61600	41.63100	-42.36800	-17.25200
C	29.69000	-61.46200	-10.73300	18.76600	-47.27900	-3.51400	39.57400	-44.41600	-10.40200
C	32.68800	-70.08400	-10.26300	19.73400	-48.04900	-4.11400	38.39100	-40.15300	-17.92400
C	35.21000	-66.77700	-11.98800	15.02700	-47.68900	-7.84300	35.23100	-44.54600	-16.88900
C	37.55300	-56.80600	-7.75100	17.20300	-53.15300	-2.30600	34.24000	-40.45700	-3.36400
C	34.48800	-52.99700	-7.96900	15.88500	-53.46100	-1.81000	38.40800	-44.69800	1.17600
C	33.77400	-60.24200	-11.94900	15.56900	-53.31100	-0.43600	34.88900	-44.17200	-7.44300
C	27.25400	-59.06800	-7.39700	16.53500	-53.09300	0.49700	41.98400	-43.47300	-7.20500
C	30.40200	-56.85900	-11.80000	17.83900	-52.61900	-0.01700	39.70000	-46.50300	-4.83100
C	29.61500	-55.78200	-11.99300	18.04100	-44.45700	-6.21800	38.98000	-47.64700	-4.65800
C	33.96600	-58.79300	-4.08900	23.87100	-52.38700	-7.87700	38.59300	-38.09400	-3.27200
C	35.25300	-59.29900	-4.02900	24.68200	-53.23100	-8.70400	39.10900	-37.43000	-4.36100
C	30.64200	-54.64200	-4.95500	24.47700	-54.59200	-8.84700	41.61200	-41.00600	-0.88600
C	31.61500	-53.66100	-4.83700	23.39600	-55.29300	-8.10800	42.35100	-42.08800	-0.50700
C	31.14700	-63.88100	-5.37800	22.68000	-54.51900	-7.16900	38.98300	-39.07100	-10.38500
C	28.96600	-67.43700	-4.63200	25.84600	-54.41000	0.27200	40.34500	-37.04700	-13.88300
C	31.86300	-66.75400	-3.58400	26.01500	-55.09300	1.56600	37.68900	-36.41900	-12.35400
C	30.08400	-64.75400	-4.99200	25.57000	-56.42700	1.78500	39.76700	-38.25500	-11.26100
C	29.26000	-62.11100	-7.02800	24.89400	-57.05100	0.66800	41.19300	-40.96200	-9.38500
C	29.52500	-61.62400	-4.27300	24.72600	-56.35800	-0.56600	40.13200	-38.73400	-7.44300
C	35.14000	-68.10700	-7.43500	37.56500	-56.31600	-7.99500	32.03800	-42.15000	-16.57400
C	34.87600	-69.01000	-6.39500	39.72800	-57.28900	-9.72600	32.82300	-41.30500	-17.32900
C	34.81100	-68.53000	-5.05000	40.36200	-53.66000	-9.66500	33.73400	-40.38800	-16.71000
C	34.90100	-67.15400	-4.80100	42.69600	-51.92300	-10.12500	33.86700	-40.35400	-15.28100
C	35.27500	-66.28300	-5.78000	39.92900	-50.85500	-8.41400	33.15300	-41.33600	-14.55100
C	41.83600	-68.38800	-5.16600	39.49200	-54.20000	-8.79600	26.23600	-46.38500	-16.23600
C	42.13900	-69.01000	-3.92300	30.23600	-57.01100	-10.85300	24.82800	-46.39300	-16.51700
C	41.93500	-68.35200	-2.71400	31.53900	-56.97800	-10.32900	23.97000	-46.09200	-15.40200
C	41.35000	-67.05900	-2.72500	33.28100	-53.15400	-12.18000	24.48400	-46.01700	-14.00300
C	40.98300	-66.40700	-3.96300	33.49900	-54.00900	-13.25800	25.86300	-46.32400	-13.84100
C	46.24200	-67.11700	-6.93300	38.44200	-62.33800	-12.38800	26.19300	-50.25300	-15.07600
C	39.84700	-66.72900	-13.69500	37.29900	-61.96100	-11.64400	33.86800	-50.32100	-14.88000
C	40.08100	-67.09700	-15.03800	35.59600	-60.27800	-7.86100	34.40000	-51.49500	-15.43600
C	41.07100	-68.07100	-15.22600	40.23500	-59.03200	-12.80600	34.45600	-51.63600	-16.84600
C	41.97500	-68.50800	-14.21700	32.10700	-61.85800	-14.82900	34.13600	-50.55200	-17.67700

C	41.66700	-68.07400	-12.93100	35.75900	-56.23000	-16.86800	33.39400	-49.49600	-17.11200
C	49.62100	-66.66800	-3.13600	47.60100	-55.64900	-11.06000	20.95800	-49.53100	-12.93500
C	44.47700	-65.13100	-10.69500	46.88200	-52.20700	-7.13600	30.43700	-51.11300	-14.12600
C	45.31500	-64.31800	-11.55200	41.97700	-59.76600	-8.40400	30.96100	-52.49000	-13.97000
C	45.49200	-62.99800	-11.29000	46.94700	-56.30600	-4.01800	30.59600	-53.17200	-12.78400
C	44.68300	-62.35100	-10.31800	47.14200	-59.75500	-8.08200	30.04600	-52.40100	-11.70900
C	43.73800	-63.05300	-9.54300	41.33900	-56.14300	-4.87800	29.67300	-51.05100	-11.78100
C	38.06600	-63.78800	-3.93600	42.71900	-55.37000	-11.70300	27.20600	-42.68000	-12.75600
C	37.51000	-62.94600	-2.97200	46.64300	-60.91500	-7.47000	25.93900	-42.40000	-12.15500
C	37.65000	-61.52500	-3.15600	46.68700	-62.13700	-8.20000	25.94200	-42.12300	-10.73600
C	38.57200	-61.01400	-4.07000	47.51300	-62.20600	-9.35900	27.06600	-42.34800	-9.87900
C	39.20600	-61.93000	-4.95300	48.04100	-61.08300	-9.97400	28.30100	-42.72000	-10.50400
C	36.10200	-63.28800	-12.22400	47.85800	-59.81300	-9.34500	34.99000	-46.05700	-10.82500
C	35.58000	-63.00200	-13.50000	36.22000	-59.35000	-8.65000	35.77200	-46.88100	-9.97200
C	36.38400	-63.36700	-14.59800	31.88700	-60.82100	-13.92500	36.68000	-47.81400	-10.56900
C	37.63200	-63.98600	-14.49500	48.08500	-52.27300	-7.88600	36.95200	-47.80200	-11.99500
C	38.15200	-64.06900	-13.20700	49.26300	-51.89500	-7.22000	36.14400	-46.97300	-12.79800
C	52.82800	-63.66500	-5.49400	49.23700	-51.36000	-5.88700	21.27200	-53.76100	-12.74900
C	47.59700	-60.63100	-9.53700	48.00800	-51.18300	-5.24000	27.76400	-53.40800	-9.09300
C	40.09400	-70.80900	-7.19900	46.81300	-51.73500	-5.80200	31.17600	-44.79400	-18.94500
C	39.92700	-72.02200	-6.48400	42.31600	-56.55000	-12.41000	30.36400	-43.85200	-19.62800
C	38.67900	-72.62100	-6.68700	42.59200	-56.74000	-13.78200	29.12500	-44.32500	-20.08500
C	37.63500	-72.00700	-7.32100	43.55600	-55.94200	-14.37900	28.44200	-45.52700	-19.56700
C	37.81300	-70.73200	-7.95100	43.82400	-54.65900	-13.77700	29.17500	-46.22000	-18.57900
C	38.94200	-63.30200	-4.89900	43.44100	-54.48500	-12.44900	28.38600	-42.81400	-11.90900
C	43.76300	-64.47200	-9.59800	41.37900	-57.06700	-3.81800	29.80400	-50.45800	-13.08900
C	39.10300	-70.13800	-8.00800	40.56600	-56.88100	-2.62400	30.56700	-45.92100	-18.32100
C	40.69500	-67.03700	-12.60100	39.83100	-55.63500	-2.61400	33.49500	-49.26500	-15.70200
C	41.16400	-67.16400	-5.16700	39.80400	-54.75000	-3.73400	26.73300	-46.61800	-14.92100
C	37.45800	-63.66500	-11.98000	40.70900	-54.90400	-4.80300	35.18800	-46.09900	-12.21600
C	35.56800	-66.74300	-7.09000	34.68400	-56.98100	-16.52000	32.13200	-42.18100	-15.19900
C	50.34200	-67.82500	-3.39000	47.53300	-57.57000	-3.80400	21.00200	-48.18100	-13.18900
C	53.96700	-64.33400	-5.95900	48.78400	-57.63000	-3.14000	20.67700	-54.62500	-11.83900
C	44.99700	-66.69200	-6.50200	49.40000	-56.46600	-2.60200	25.09300	-50.06900	-15.95800
C	48.09000	-60.80000	-10.82100	48.77000	-55.18500	-2.81100	27.88300	-54.70400	-9.51000
C	49.89600	-65.49700	-10.16700	47.62300	-55.09900	-3.67200	27.51400	-53.91000	-13.20000
C	48.77700	-65.40700	-10.98800	39.40600	-58.64000	-13.86600	27.75700	-54.31100	-14.51600
C	46.56300	-62.76500	-2.91900	41.71600	-60.27700	-9.69400	22.31300	-48.74300	-8.44900
C	46.70900	-61.87100	-1.84600	40.47000	-60.82500	-10.01600	21.56900	-47.63800	-8.94100
C	50.95700	-59.75700	-4.97600	39.45300	-60.77900	-9.03200	21.89700	-53.21400	-8.39600
C	51.41100	-60.19100	-3.73800	39.68400	-60.36700	-7.70000	22.03900	-54.49900	-7.99600
C	42.16100	-61.53500	-6.53500	40.96700	-59.87700	-7.37100	28.49500	-46.69700	-9.02500
C	40.91600	-59.02000	-9.90100	48.59200	-56.63500	-11.02300	31.57800	-45.62000	-6.89900
C	39.41500	-59.61700	-7.31400	48.94600	-57.24500	-12.19100	31.53800	-43.76700	-8.96400
C	42.40300	-60.50600	-7.39700	48.28000	-56.95400	-13.46500	28.99000	-45.36400	-9.00400

C	44.19400	-61.13100	-4.28500	47.18400	-56.08200	-13.40500	25.48300	-46.86000	-7.49100
C	43.07700	-63.80900	-4.68600	46.91200	-55.30500	-12.27800	25.72700	-45.97900	-10.16600
C	47.13100	-55.93600	-18.13900	39.27400	-62.61100	0.55200	29.61900	-56.98200	-7.39000
C	47.00200	-56.82500	-19.27500	40.07400	-59.78400	-0.27400	29.76000	-58.12100	-8.27700
C	47.77900	-57.96400	-19.35000	38.65400	-60.31500	-3.11800	28.80100	-59.18600	-8.22100
C	48.70800	-58.31900	-18.39600	35.76600	-59.57000	-3.02600	27.85300	-59.24500	-7.10900
C	48.84800	-57.44200	-17.25900	37.54600	-58.29700	-4.98500	27.84200	-58.24500	-6.13400
C	42.22800	-60.38600	-12.51400	38.43100	-61.34100	-2.19500	29.68900	-51.26500	-7.25100
C	41.85100	-61.70300	-12.19200	46.92800	-56.58600	1.07400	29.61400	-50.25900	-8.21900
C	42.31500	-62.60900	-13.12200	46.38400	-56.83400	-0.18100	30.47700	-49.16600	-8.15500
C	43.29100	-62.35700	-14.15000	42.13100	-60.54700	3.52500	31.37900	-49.08600	-7.06400
C	43.75900	-61.05000	-14.22600	42.26600	-61.21000	2.25300	31.44900	-50.10800	-6.08100
C	43.14100	-59.00100	-16.88100	47.13500	-63.13400	-4.96500	31.71500	-54.09300	-8.70800
C	50.14500	-57.30800	-9.85600	47.74100	-62.65800	-3.83000	25.20800	-51.96500	-2.54800
C	50.59900	-57.78400	-8.64000	43.02600	-60.19600	-4.95600	23.90500	-51.63600	-2.22500
C	49.86100	-57.54400	-7.47300	45.13900	-66.12100	-2.60300	23.50400	-51.66500	-0.82700
C	48.60900	-56.84700	-7.54700	50.16300	-59.52700	-0.81900	24.41600	-51.79500	0.21000
C	48.16300	-56.38100	-8.78900	47.16200	-62.76200	3.50400	25.70900	-52.19000	-0.15500
C	38.76900	-58.16400	-20.26000	32.67200	-66.64500	-3.49500	36.58500	-55.96800	-10.56500
C	44.34400	-56.62700	-7.87300	33.91800	-63.09100	-8.32600	31.51700	-50.70300	0.13600
C	45.08700	-56.80800	-6.73100	39.89500	-66.50600	-2.60600	31.70600	-49.75100	1.16500
C	45.71600	-58.05900	-6.48300	37.09400	-66.30200	-9.48900	30.68800	-49.11100	1.82700
C	45.58100	-59.14800	-7.36200	36.59600	-69.43900	-5.45100	29.34300	-49.58300	1.51500
C	44.82000	-58.99200	-8.55100	40.53300	-63.56500	-5.97400	29.03000	-50.43700	0.45000
C	43.23800	-55.49400	-17.22300	35.10300	-63.05200	-1.55300	32.83700	-56.64700	-6.26900
C	42.02900	-55.16700	-17.87700	36.85500	-69.94600	-6.76800	34.02100	-57.26200	-6.79700
C	41.19800	-54.18200	-17.32100	37.57000	-71.16600	-6.85700	35.22700	-57.29000	-6.01900
C	41.61200	-53.33400	-16.23400	38.20200	-71.69900	-5.76000	35.08400	-57.02700	-4.62100
C	42.81200	-53.72300	-15.64100	37.98900	-71.16600	-4.45400	33.91000	-56.44400	-4.07600
C	48.30700	-53.22100	-10.68500	37.29900	-69.96500	-4.34000	28.67300	-58.81900	-0.89500
C	49.27100	-53.28300	-9.62400	44.33000	-59.78400	-4.62300	27.89800	-59.58500	-0.01400
C	50.62300	-53.16600	-9.94200	49.63900	-60.64100	-0.27200	27.92100	-59.13500	1.38000
C	51.02800	-53.20800	-11.30400	34.50200	-63.23300	-9.56400	28.54800	-57.94300	1.78800
C	50.08600	-53.21800	-12.30100	33.85200	-63.02800	-10.72500	29.16500	-57.15500	0.79300
C	36.31500	-61.89000	-18.23700	32.55500	-62.44600	-10.66200	37.28600	-51.14200	-12.27300
C	38.25200	-60.67300	-11.46500	31.93000	-62.33400	-9.37500	35.86600	-48.07600	-6.81500
C	47.44200	-60.98300	-15.67100	32.54400	-62.71500	-8.20600	26.60600	-51.38700	-6.05100
C	48.30500	-61.62700	-16.56200	33.77300	-63.39800	-1.33900	25.53000	-50.44200	-6.02500
C	49.64800	-61.76900	-16.23600	33.20300	-63.38400	0.00500	24.15200	-50.92100	-5.77500
C	50.14800	-61.30400	-15.04200	33.97100	-62.87800	1.10800	23.94200	-52.31500	-5.65900
C	49.29700	-60.68100	-14.08900	35.27900	-62.42500	0.88500	25.04200	-53.21700	-5.77900
C	43.70200	-54.75200	-16.09600	35.85800	-62.60300	-0.40000	32.81500	-56.28500	-4.90300
C	44.26600	-57.67400	-8.84900	41.25400	-63.89500	-4.74200	30.16800	-51.11900	-0.09700
C	47.92200	-60.49000	-14.38100	42.72000	-63.77700	-4.77700	26.36600	-52.77200	-5.97400
C	48.93800	-56.63500	-9.94000	43.39900	-63.63800	-6.01400	26.11400	-52.28500	-1.49600

C	43.19000	-60.00800	-13.48100	42.71200	-63.38200	-7.23500	30.67900	-51.23400	-6.22700
C	48.71800	-53.40800	-12.00500	41.27100	-63.42800	-7.16300	29.19000	-57.53600	-0.52100
C	47.97100	-56.33800	-17.09400	45.84600	-62.53700	3.17100	28.65100	-57.14700	-6.34800
C	37.86000	-57.14700	-20.53100	37.63400	-65.31900	-10.34200	36.38900	-57.24600	-10.04400
C	36.98000	-62.86700	-18.90700	38.26800	-65.69400	-11.60000	38.55800	-51.58300	-12.70600
C	43.55400	-59.89700	-17.81900	38.43600	-67.05900	-11.83700	31.69700	-54.44800	-10.05800
C	37.70600	-59.62900	-10.63800	38.09100	-68.05900	-10.90300	34.67200	-47.68700	-6.27600
C	40.48800	-62.75500	-16.14100	37.38200	-67.70400	-9.69600	33.17300	-49.83100	-10.68000
C	41.42500	-62.59500	-17.12200	44.92200	-65.76000	-1.28000	33.92400	-49.46200	-11.77800
C	38.11300	-55.00100	-16.47000	40.54700	-65.53200	-1.87900	38.02300	-55.43800	-6.51600
C	37.35000	-54.67500	-17.56100	41.58700	-65.88200	-1.06100	39.12500	-55.49100	-5.76100
C	34.42600	-59.50400	-14.98600	41.99300	-67.21500	-0.97500	40.12200	-50.63200	-8.15300
C	33.59300	-58.44500	-15.17900	41.27500	-68.29600	-1.62600	40.26900	-49.27900	-7.85600
C	41.14700	-54.01100	-11.60900	40.18900	-67.88200	-2.44300	34.54900	-52.73000	-1.85900
C	42.79600	-50.45500	-11.17700	31.61600	-65.94100	-2.86800	32.21500	-53.78500	1.80700
C	44.18100	-51.46300	-13.55600	30.54000	-66.71400	-2.44700	35.04500	-54.53900	1.21100
C	41.69800	-53.19200	-12.58700	30.45400	-68.11100	-2.72800	33.99500	-52.41300	-0.61100
C	39.19100	-56.16200	-10.95200	31.50300	-68.72800	-3.34900	33.15800	-50.21900	-3.38800
C	38.76800	-54.06300	-13.33700	32.68500	-68.04100	-3.60200	35.97400	-50.03400	-2.64500
C	28.42200	-56.60800	-1.59700	27.26300	-59.01300	-1.16200	45.49900	-38.69500	-5.55000
C	29.77400	-56.96500	-1.23600	25.46300	-59.63300	-3.51000	44.53900	-37.83500	-6.11500
C	30.50000	-57.84300	-2.03600	26.10100	-62.70900	-2.64000	43.18400	-37.82400	-5.60900
C	29.93500	-58.47900	-3.17400	26.36800	-64.79400	-0.25600	42.76000	-38.85300	-4.71000
C	28.61200	-58.22900	-3.49600	24.39300	-65.50500	-2.22700	43.80700	-39.67900	-4.11600
C	23.19100	-56.40200	-9.56800	27.12200	-61.97800	-2.18400	43.71800	-46.59800	-6.88900
C	23.17200	-56.50500	-11.01500	28.30500	-51.65800	-1.41400	42.62100	-47.51200	-6.63200
C	24.36400	-56.28000	-11.74900	28.76100	-52.86200	-0.90300	42.80400	-48.58600	-5.75100
C	25.51500	-55.81200	-11.04900	31.48600	-57.32100	0.82300	44.09800	-48.88200	-5.29300
C	25.60300	-55.80400	-9.61100	31.08100	-57.86700	-0.39900	45.18600	-48.04000	-5.51800
C	18.96000	-48.40900	-9.96200	28.14000	-54.92700	-8.76800	52.29400	-51.15800	-3.25500
C	20.94200	-55.38700	-1.39900	27.51700	-55.00900	-7.53500	49.88800	-41.39300	-9.18000
C	19.84200	-56.09500	-0.83300	24.82400	-56.91700	-5.21900	51.06900	-40.91300	-9.78200
C	19.02100	-56.80100	-1.74100	32.63700	-58.68900	-7.37000	52.17200	-41.84800	-9.80400
C	19.15000	-56.64600	-3.14700	32.99700	-52.39800	-3.69500	52.11500	-43.17700	-9.32500
C	20.19000	-55.86700	-3.65700	34.81000	-55.90400	-2.75100	50.86400	-43.58000	-8.72500
C	16.02600	-52.93200	-9.02800	32.10900	-66.36300	-7.90700	52.11700	-49.80800	-7.82500
C	20.42600	-51.77000	-6.37500	28.36800	-68.85900	-7.18100	51.38700	-46.23300	-7.11900
C	19.76100	-50.53000	-6.56200	28.06200	-60.05500	-7.78500	52.79500	-46.45600	-7.17900
C	20.27700	-49.38000	-5.91000	25.80700	-66.37500	-9.96700	53.38200	-47.14500	-6.07600
C	21.24000	-49.51600	-4.86300	28.67600	-64.66500	-11.11800	52.56100	-47.51300	-4.94200
C	21.86500	-50.77600	-4.71200	24.10800	-62.41200	-6.14900	51.15300	-47.17300	-4.95300
C	28.26100	-54.19700	-8.51300	31.33000	-62.57700	-4.38500	43.11200	-45.45100	-3.20500
C	28.88900	-53.58000	-9.59700	27.51200	-64.02300	-12.11500	42.36000	-46.34100	-2.42500
C	29.35800	-52.26500	-9.48000	27.85100	-63.71900	-13.82600	42.39200	-46.35900	-1.01300
C	29.34500	-51.72400	-8.17300	29.46600	-64.02100	-14.41400	43.40100	-45.60100	-0.37500

C	28.89900	-52.43300	-7.04600	30.65500	-64.69200	-13.33200	44.17000	-44.65300	-1.12800
C	24.68600	-52.91200	0.40500	30.28600	-64.96700	-11.71200	50.45200	-39.50300	-3.09000
C	25.27800	-52.63300	1.68400	25.27400	-55.71600	-4.73700	50.58200	-38.31400	-2.37300
C	25.90300	-53.74800	2.31700	31.69700	-51.98800	-3.91200	49.68000	-37.25100	-2.62300
C	26.24800	-54.98600	1.68500	29.42400	-69.47400	-6.39900	48.74800	-37.32100	-3.69500
C	25.77800	-55.10700	0.35500	29.35900	-70.86400	-6.09900	48.76800	-38.47400	-4.51000
C	16.46600	-52.55200	-14.35200	28.20900	-71.58700	-6.40000	49.56000	-55.44700	-7.66700
C	23.08800	-51.73600	-14.14500	27.09000	-70.99100	-6.99400	45.06300	-52.08500	-3.54700
C	22.19500	-59.02500	-4.04000	27.21100	-69.64900	-7.44300	46.05100	-41.25600	-9.82300
C	21.15500	-59.97800	-4.30500	30.79400	-62.44300	-3.06400	45.97300	-39.89000	-10.10800
C	20.65300	-60.08600	-5.65600	31.32300	-61.40800	-2.22900	44.80700	-39.27200	-9.72300
C	21.23800	-59.24800	-6.65100	32.15700	-60.37900	-2.79400	43.87600	-39.74500	-8.82700
C	22.16300	-58.19900	-6.37700	32.59300	-60.50100	-4.11700	44.13500	-41.04600	-8.28000
C	28.38800	-53.71000	-7.21100	32.16700	-61.54400	-4.98000	43.97800	-44.54600	-2.51100
C	21.34800	-51.89800	-5.29800	24.12300	-61.04200	-6.33800	50.51500	-46.64800	-6.08300
C	22.55900	-58.09500	-5.05100	23.08900	-60.27400	-5.68700	45.20200	-41.82300	-8.81100
C	21.03800	-55.12900	-2.80500	21.95500	-60.92800	-5.06800	49.73900	-42.69300	-8.64900
C	24.37200	-56.17100	-8.88600	22.09900	-62.32400	-4.91100	45.00700	-46.93500	-6.39000
C	24.97400	-54.13300	-0.30500	23.18800	-63.11000	-5.29800	49.63800	-39.58500	-4.24400
C	27.83400	-57.22900	-2.74900	34.14700	-56.41500	-3.86600	45.12700	-39.66800	-4.60200
C	16.35300	-52.54900	-7.73900	26.08500	-67.75900	-9.90800	52.18700	-49.94700	-9.23600
C	16.20300	-53.35000	-15.43100	25.32200	-68.66400	-10.63000	50.27100	-55.73800	-8.84800
C	19.97300	-48.33600	-9.03900	24.30800	-68.22600	-11.50100	53.45100	-50.86400	-3.99600
C	23.44800	-50.43700	-13.79800	23.91500	-66.83900	-11.53200	44.46300	-53.09900	-2.86700
C	19.15500	-50.03600	-15.20300	24.70700	-65.94900	-10.76700	49.93600	-54.84400	-2.93000
C	17.82100	-49.55800	-15.26500	31.34700	-58.89200	-6.89400	51.23200	-55.35100	-2.98200
C	19.71100	-54.96500	-7.83400	28.46100	-59.74600	-9.09700	47.27100	-48.52000	-8.63200
C	18.63500	-54.47400	-7.14200	27.74000	-58.84100	-9.94400	46.51000	-49.16300	-9.65200
C	20.94500	-55.71500	-14.01600	26.46900	-58.36600	-9.46900	44.97000	-52.84800	-8.00500
C	20.14700	-56.76000	-14.46200	26.02100	-58.58800	-8.11000	43.93900	-52.11000	-7.43300
C	24.36300	-49.98900	-7.56300	26.82400	-59.43400	-7.29100	46.92200	-46.86600	-1.29700
C	25.56600	-47.44000	-5.03400	32.34100	-67.57000	-7.27800	49.43100	-45.58300	1.16600
C	27.89100	-49.16400	-5.26800	33.25800	-68.47200	-7.88200	46.92600	-43.64200	0.17100
C	25.72800	-49.84500	-7.34600	33.87200	-68.15900	-9.11900	47.96900	-46.64100	-0.46100
C	24.28000	-49.77200	-10.74100	33.50900	-66.98100	-9.83200	46.83500	-50.13400	-0.60800
C	25.22800	-52.30900	-9.62600	32.64000	-66.06600	-9.19600	44.86700	-48.96300	-2.11300
H	35.00300	-65.93900	-14.00800	23.78800	-49.68300	-7.39000	34.73500	-42.73500	-17.86200
H	36.92800	-67.24600	-15.03500	25.15100	-49.49200	-6.43300	34.51100	-43.68000	-20.12400
H	37.72500	-69.05900	-13.72100	23.57300	-49.98200	-5.87400	34.43300	-46.32600	-20.14100
H	37.35200	-69.33400	-11.30500	24.50400	-45.50700	-7.30100	35.30400	-47.51700	-18.43900
H	34.96200	-68.32100	-10.59400	25.57900	-46.67900	-7.53000	36.04000	-46.59300	-16.42200
H	28.17400	-61.64900	-9.08700	24.18900	-46.42800	-8.66600	40.67400	-45.94700	-11.40600
H	26.82700	-59.79600	-9.89600	25.96300	-46.76500	-4.10500	40.54800	-47.48200	-9.35700
H	26.96000	-59.47300	-12.30700	25.44400	-48.40800	-4.11400	39.41800	-46.91500	-7.37500
H	29.18200	-59.69800	-13.47200	27.26100	-48.02200	-1.67100	38.39500	-44.66800	-7.24500

H	30.93400	-60.92300	-12.31100	26.15600	-49.18400	-1.71500	38.07900	-43.37400	-9.39700
H	34.27300	-58.28800	-11.24100	26.19900	-48.13400	-0.25800	35.09900	-42.17300	-6.76900
H	32.80500	-58.35700	-11.64000	24.35800	-45.50600	-0.35400	34.51400	-43.09500	-5.69900
H	30.48500	-69.67300	-12.29200	23.58900	-45.28500	-1.87400	42.15800	-40.69200	-15.90500
H	29.16700	-71.84600	-12.22600	25.36800	-44.95000	-1.53700	44.27200	-40.24600	-17.08000
H	26.85600	-71.71900	-13.10400	23.19300	-47.29100	-3.96200	45.08800	-41.63200	-18.83800
H	25.64300	-69.84400	-12.61700	23.86100	-45.76400	-4.32900	43.77300	-43.66900	-19.53700
H	26.92300	-67.57400	-12.88800	19.92600	-51.62400	-13.96100	41.87100	-44.23500	-18.50500
H	36.50500	-55.68900	-8.84900	19.47400	-49.93800	-13.97100	34.00900	-42.12800	-2.03500
H	36.21400	-55.39000	-7.05500	20.75100	-50.46400	-13.75800	34.66700	-40.97900	-1.35400
H	27.86600	-64.22900	-6.71800	18.75200	-51.30200	-11.51500	41.79300	-38.93400	-12.43900
H	25.54800	-63.82700	-6.05400	20.09900	-51.58100	-11.42000	43.86300	-38.11900	-13.32800
H	23.71100	-64.44700	-7.73400	20.03200	-53.37800	-8.67100	45.75000	-39.80600	-13.83400
H	24.35600	-66.52500	-9.22700	19.98800	-52.01400	-9.86000	45.44400	-42.12300	-13.40400
H	26.62900	-66.79200	-9.78700	18.60200	-52.66000	-9.13400	43.16300	-42.95900	-12.85300
H	35.13700	-62.03000	-9.92900	19.45100	-51.72000	-6.85700	33.50500	-42.79600	-11.54100
H	36.53600	-60.51000	-8.63000	20.98500	-51.44500	-7.61500	32.64900	-40.77400	-10.25300
H	36.81600	-60.85100	-6.25900	20.50500	-42.66900	-11.76400	34.00000	-40.03400	-8.24800
H	35.21200	-62.48600	-5.05900	19.95600	-41.88400	-10.39300	36.48500	-40.43400	-8.38200
H	33.95300	-64.27400	-6.54600	19.40700	-41.41600	-11.90600	37.45200	-41.93600	-10.20700
H	33.08000	-70.31600	-8.14500	17.52300	-43.13500	-10.85900	36.65600	-40.73800	-19.15000
H	35.11100	-71.44000	-8.32000	18.20000	-43.56200	-12.11900	37.62100	-39.76900	-21.21500
H	35.41400	-72.74300	-10.54100	24.09800	-47.05200	-12.31700	39.96700	-39.68000	-21.48900
H	34.53400	-71.85200	-12.65700	25.05300	-45.94600	-11.57600	41.26900	-39.15700	-19.36600
H	32.43100	-70.27900	-12.39700	24.67400	-47.39200	-10.84900	40.26900	-39.53700	-17.08600
H	32.38100	-52.98700	-7.46300	16.17200	-44.06400	-5.49800	40.12700	-44.76600	-0.12800
H	32.71000	-52.71000	-8.99700	16.09000	-45.43700	-6.17700	39.01900	-45.83700	-0.48500
H	27.26100	-56.97700	-8.02200	16.05500	-44.13600	-7.11000	42.48000	-44.41300	-5.39600
H	27.38800	-57.27900	-6.56500	15.77200	-49.36900	-12.11200	43.14400	-42.80800	-5.79700
H	29.49900	-65.40400	-15.62800	15.22700	-48.59500	-13.56400	38.10900	-46.98400	-17.76700
H	31.14800	-65.69600	-18.07200	14.79500	-48.28600	-11.93000	39.73400	-48.92600	-18.76500
H	33.00700	-63.44700	-18.38100	13.53800	-47.99300	-6.38000	40.67300	-50.90000	-17.06000
H	34.15400	-62.46400	-16.20600	14.65200	-49.25500	-6.47700	41.05700	-50.27400	-14.43300
H	32.92400	-62.57900	-13.78300	15.06800	-47.85800	-5.73500	40.38200	-47.77900	-13.47700
H	37.70200	-57.40700	-6.92100	21.92400	-56.45500	-5.79700	34.28800	-40.76500	-4.42200
H	38.45100	-56.37400	-7.45300	19.97200	-57.07000	-6.95000	34.86900	-39.58700	-3.51000
H	37.75700	-57.54400	-8.52700	18.14300	-58.16900	-5.54300	33.17600	-40.10800	-3.29300
H	35.06100	-53.44000	-7.21200	17.78800	-57.02900	-3.04100	37.33400	-44.61600	1.22000
H	35.18300	-53.41800	-8.65900	20.05600	-56.54500	-1.96100	38.43100	-45.47600	1.87600
H	34.88800	-52.04600	-7.90300	22.88300	-44.97800	-11.20900	38.73400	-43.86600	1.72100
H	33.89200	-60.16500	-13.02300	23.26100	-45.35000	-9.78700	34.00100	-43.99300	-7.99900
H	32.98700	-60.92300	-11.83000	17.46900	-47.75800	-13.12400	34.56100	-45.07100	-7.02400
H	34.46500	-60.93000	-11.51500	16.46800	-46.43100	-12.90100	35.32200	-44.26100	-8.40100
H	27.59900	-59.72500	-6.63800	19.98100	-50.89600	1.49500	41.58200	-42.56400	-7.53500
H	26.18600	-59.37700	-7.33000	19.66200	-51.06100	3.83200	42.75600	-43.68300	-7.95500

H	27.80800	-59.72900	-8.10000	21.34900	-52.31900	5.27000	41.19500	-44.21100	-7.42400
H	31.32100	-56.63700	-12.35400	23.71800	-52.33400	4.40500	40.19300	-46.43300	-5.78300
H	30.09200	-57.80700	-12.19600	24.22200	-51.91600	2.10000	40.57800	-46.38200	-4.24000
H	28.74400	-55.93900	-11.40200	27.06600	-49.86300	-3.12200	38.48600	-47.87800	-3.72400
H	29.18900	-55.46000	-12.92900	28.60900	-48.89500	-4.83700	39.58400	-48.60000	-4.84800
H	30.04200	-54.88900	-11.60200	28.92600	-49.87500	-6.99400	38.09300	-47.85400	-5.16700
H	33.86800	-58.14800	-3.26700	27.54000	-52.02900	-7.63000	37.68200	-37.66000	-2.86100
H	33.08500	-59.33500	-3.75400	26.10700	-52.95900	-5.80900	39.23900	-38.09000	-2.35000
H	35.61800	-59.62300	-3.06700	18.13000	-50.98500	-3.92400	39.24500	-36.38900	-4.50500
H	36.07600	-58.68900	-4.18200	16.18500	-49.39700	-3.50300	38.39100	-37.29700	-5.14900
H	35.42300	-60.11000	-4.73000	16.57000	-46.97900	-3.63800	40.02100	-37.81900	-4.79300
H	30.18300	-54.99900	-4.03900	18.96200	-46.24000	-3.38200	41.16200	-40.44100	-0.07600
H	29.76400	-54.16400	-5.32300	20.73900	-47.69300	-3.92800	42.19300	-40.22700	-1.27100
H	31.29800	-52.91800	-4.16800	15.00600	-46.59200	-7.99600	43.30400	-41.89000	0.00200
H	31.75700	-52.95200	-5.64900	14.47100	-48.13500	-8.64800	42.49700	-42.70500	-1.33300
H	32.62200	-53.83100	-4.43500	17.31800	-53.02900	-3.35600	41.98600	-42.75300	0.25600
H	31.68400	-64.20200	-6.24000	15.09000	-53.95400	-2.37600	38.71000	-39.99900	-10.82900
H	32.06200	-63.89100	-4.74000	14.68100	-53.67100	-0.07000	38.12600	-38.61400	-9.93400
H	28.31000	-67.22500	-5.42100	16.11700	-52.78300	1.47800	41.15800	-37.73200	-14.07100
H	29.02600	-68.53500	-4.43900	18.72600	-52.60600	0.57400	39.98300	-36.70900	-14.79000
H	28.44300	-66.97700	-3.80700	18.60200	-44.88000	-5.31100	40.85100	-36.16000	-13.64800
H	31.42300	-66.57000	-2.58200	18.46800	-43.51800	-6.25800	38.16300	-35.57700	-11.93200
H	32.56300	-65.92100	-3.68600	23.63100	-51.38800	-8.24800	36.98200	-36.68600	-11.64700
H	32.36100	-67.68800	-3.69100	25.36900	-52.79200	-9.42100	37.16800	-36.04800	-13.17000
H	29.25500	-64.76800	-5.62500	25.38900	-55.13000	-9.07500	40.75200	-38.53600	-11.60700
H	29.55800	-64.47700	-4.09800	23.24700	-56.32400	-8.07200	40.32200	-37.44400	-10.80900
H	28.32800	-62.57100	-6.94600	21.72000	-54.87700	-6.76300	42.10600	-40.37500	-9.51000
H	29.33700	-62.83000	-7.78900	25.96200	-53.34400	0.11600	40.90100	-41.84300	-9.94200
H	29.16900	-61.17200	-7.50400	26.84200	-54.77000	2.22100	41.65500	-41.26600	-8.43000
H	30.12900	-61.60700	-3.33100	25.63700	-57.00000	2.70500	39.35100	-38.06900	-7.19000
H	28.93800	-60.71900	-4.39100	24.46200	-57.99700	0.87100	40.49000	-39.20000	-6.50100
H	28.91000	-62.50900	-4.16200	24.66200	-56.92100	-1.52700	40.99800	-38.07600	-7.62800
H	35.00600	-68.41500	-8.44100	37.04400	-57.20500	-8.35800	31.67700	-43.01900	-17.01500
H	34.75600	-70.04100	-6.56300	38.17900	-56.48600	-7.12800	32.45400	-40.98600	-18.27500
H	34.86700	-69.30200	-4.33900	36.62200	-55.72900	-7.84600	34.39000	-39.87200	-17.41500
H	34.57200	-66.64700	-3.89400	40.16800	-57.70100	-8.83200	34.42500	-39.54000	-14.93000
H	35.41700	-65.23200	-5.56700	39.17200	-58.12000	-10.05700	33.00000	-41.27400	-13.44400
H	41.78600	-69.03200	-5.96000	40.40200	-57.18400	-10.53600	26.84500	-46.45500	-17.11300
H	42.65000	-69.92900	-3.84300	41.18900	-54.23800	-9.91300	24.44200	-46.32400	-17.56200
H	41.78100	-68.82700	-1.74700	39.94800	-53.56100	-10.64600	22.94900	-45.86600	-15.70600
H	41.15400	-66.57200	-1.82600	43.24700	-51.00700	-9.87100	23.71500	-45.65300	-13.31000
H	40.69600	-65.37000	-3.91000	43.32800	-52.79800	-10.25500	26.34800	-45.71000	-13.13300
H	46.24200	-67.73600	-7.77800	42.47400	-51.87000	-11.15900	26.98500	-49.54700	-15.14200
H	46.75300	-67.86200	-6.37300	39.35900	-51.26400	-7.66500	26.81100	-51.08800	-15.30600
H	38.78700	-66.59000	-13.42000	40.36800	-49.93600	-8.10300	34.22300	-50.07800	-13.93300

H	39.26800	-67.11700	-15.69500	39.23100	-50.73800	-9.25500	34.48500	-52.27400	-14.73000
H	41.35700	-68.10300	-16.22300	39.87000	-54.37500	-7.82300	34.51500	-52.64800	-17.23500
H	42.65800	-69.33100	-14.37100	38.69400	-53.69400	-8.29600	34.41500	-50.68600	-18.75400
H	42.20500	-68.63300	-12.23900	29.44000	-56.64700	-10.17000	32.90300	-48.69800	-17.68300
H	48.74800	-66.80900	-2.50100	29.91000	-56.55200	-11.78600	20.21800	-49.99700	-12.26900
H	49.86400	-66.01400	-2.33600	30.04000	-57.96800	-11.30300	20.48000	-50.06500	-13.74500
H	44.18200	-66.06000	-11.06600	31.88100	-56.02800	-9.91100	30.50100	-50.52100	-15.04600
H	45.99800	-64.98600	-12.13000	31.55800	-57.42300	-9.34100	31.37100	-53.04200	-14.82800
H	46.03900	-62.48300	-12.10300	34.01600	-52.85500	-11.53500	31.31300	-53.92200	-12.45300
H	44.76500	-61.28700	-10.18500	33.09500	-52.10400	-12.44400	29.95200	-53.00400	-10.83900
H	43.20000	-62.44500	-8.90600	32.58900	-53.27600	-11.37100	29.49400	-50.35400	-10.96700
H	37.76800	-64.84400	-4.00100	34.16800	-53.61400	-13.93400	27.31900	-43.03200	-13.75100
H	36.66400	-63.07600	-2.39900	32.60900	-54.25200	-13.84200	25.01400	-42.26500	-12.70500
H	37.02100	-60.86900	-2.53200	38.25700	-62.55300	-13.39900	25.17400	-41.61600	-10.28200
H	38.89600	-60.00500	-3.93200	39.16300	-61.62100	-12.65800	26.98900	-42.36900	-8.81100
H	39.59800	-61.50300	-5.88000	39.15300	-63.05100	-12.15600	29.19700	-42.73100	-9.96500
H	35.55800	-63.15100	-11.30800	36.71100	-62.87000	-11.43200	34.02900	-45.74500	-10.62300
H	34.63800	-62.47700	-13.55400	37.49800	-61.64600	-10.66200	35.67300	-46.66900	-8.91600
H	35.97400	-63.32000	-15.55300	34.71500	-59.94300	-7.52500	37.38700	-48.18800	-9.92200
H	38.34200	-63.96600	-15.36400	36.06000	-60.82700	-7.09200	37.46800	-48.54700	-12.62000
H	39.07300	-64.68300	-13.02200	35.09300	-61.08500	-8.33700	36.12300	-46.94500	-13.85500
H	52.60800	-63.88500	-4.48100	40.21800	-58.44400	-11.88800	21.33500	-53.90000	-13.78100
H	53.03100	-62.62600	-5.46400	41.30700	-59.09000	-12.94500	20.80900	-52.78300	-12.84100
H	47.29900	-59.65200	-9.22600	40.06100	-59.98300	-12.26300	27.54200	-53.24100	-8.06900
H	46.63800	-60.99500	-9.56500	32.32900	-61.67200	-15.87700	28.71600	-52.85100	-9.26400
H	41.08200	-70.42600	-7.37300	31.23000	-62.38600	-15.12100	32.16700	-44.55700	-18.68100
H	40.52100	-72.48000	-5.72400	32.90200	-62.56600	-14.76500	30.71500	-42.90500	-20.04600
H	38.62900	-73.67000	-6.43700	36.79800	-56.61600	-16.79800	28.63500	-43.80900	-20.89400
H	36.64300	-72.30600	-7.21700	36.04400	-55.32700	-16.39400	27.33600	-45.47300	-19.44600
H	36.96900	-70.24100	-8.41500	35.66900	-55.99000	-17.93300	28.82100	-47.19200	-18.39500
H	51.25700	-67.71800	-3.94200	45.99800	-60.79400	-6.61100	20.07100	-47.69300	-13.47200
H	49.91600	-68.53600	-4.06700	45.95500	-62.94100	-8.06300	21.74900	-47.83500	-13.88600
H	50.72500	-68.36000	-2.54600	47.51300	-63.17700	-9.86200	21.36200	-47.60000	-12.32900
H	53.81300	-65.37800	-5.87000	48.56000	-61.10100	-10.90100	21.06200	-55.61300	-11.74800
H	54.87700	-64.18700	-5.34000	48.10300	-58.90000	-9.82000	19.73300	-55.04300	-12.15400
H	54.25700	-64.18700	-6.96700	37.10800	-59.73800	-9.06400	20.30300	-54.32600	-10.88100
H	44.54200	-65.90300	-7.09700	36.44900	-58.44100	-8.13700	24.45700	-49.23100	-15.81800
H	44.33900	-67.51700	-6.19600	31.07000	-60.15400	-14.13000	25.26500	-50.08700	-17.04700
H	45.01800	-66.12700	-5.57900	31.54000	-61.11500	-12.98900	24.36100	-50.81600	-16.10700
H	47.86600	-61.72900	-11.28300	48.02700	-52.47500	-8.93600	28.14500	-54.90500	-10.51200
H	47.82800	-60.19500	-11.65400	50.23900	-52.05800	-7.70800	28.70500	-55.15000	-8.96600
H	49.15700	-60.66900	-10.84900	50.09700	-50.85000	-5.41800	27.09800	-55.41700	-9.32700
H	50.42000	-66.41800	-10.05200	47.91500	-50.57400	-4.36600	27.71200	-54.72900	-12.48100
H	50.71000	-65.04700	-10.54500	45.86700	-51.47200	-5.43100	28.24100	-53.28800	-12.74300
H	47.86100	-65.63300	-10.52800	42.08100	-57.43000	-11.88300	27.05500	-55.00700	-14.96100

H	48.77500	-65.93600	-11.94200	42.19100	-57.61800	-14.27700	28.60000	-54.90700	-14.78300
H	48.43000	-64.39000	-11.22100	43.72900	-56.07400	-15.44200	27.90300	-53.46400	-15.24100
H	45.67700	-62.77700	-3.50600	43.82600	-53.77200	-14.35000	21.59200	-49.43000	-8.06300
H	46.45000	-63.80500	-2.73000	43.60300	-53.56200	-11.94700	23.09300	-48.55600	-7.76700
H	47.02200	-60.82000	-2.05200	41.62200	-58.03800	-4.24000	20.85400	-47.85000	-9.74900
H	45.94500	-62.08500	-1.04800	40.55700	-57.43100	-1.74000	20.86200	-47.11800	-8.24300
H	47.62100	-61.88700	-1.31300	39.13500	-55.36900	-1.83300	22.06500	-46.76900	-9.38300
H	51.63900	-59.48100	-5.79800	39.09600	-53.97800	-3.85800	21.90900	-52.47900	-7.60600
H	50.34100	-58.83100	-4.98700	40.64200	-54.44900	-5.78900	20.98500	-52.79500	-8.61500
H	52.00400	-59.41500	-3.31100	34.54000	-57.73400	-17.25000	21.42700	-54.90600	-7.21800
H	50.73000	-60.35400	-2.96700	33.72400	-56.54000	-16.58900	22.12500	-55.25700	-8.77700
H	52.14500	-60.96800	-3.62800	47.13100	-58.43200	-4.23700	22.94000	-54.76600	-7.48000
H	41.72400	-62.37400	-6.97200	49.18300	-58.45500	-2.59000	28.84200	-47.38400	-8.26900
H	41.42400	-61.48300	-5.79900	50.30300	-56.35600	-1.99800	28.86200	-47.13400	-9.90600
H	41.54300	-59.32700	-10.70600	48.96400	-54.35000	-2.15900	31.12900	-46.44100	-6.46200
H	39.99500	-58.96800	-10.38900	47.24400	-54.14000	-4.00900	32.58800	-45.84400	-6.86700
H	41.16200	-58.00300	-9.63400	39.66900	-57.85000	-14.51700	31.19300	-44.80200	-6.30500
H	38.53800	-59.72200	-8.00700	39.40800	-59.41700	-14.65100	31.30800	-43.53000	-10.00000
H	39.20000	-60.29100	-6.48000	42.24600	-59.80100	-10.48500	31.20400	-43.14300	-8.12300
H	39.62000	-58.58600	-7.10300	40.09900	-60.92500	-11.00200	32.55000	-43.78700	-8.70300
H	42.78200	-59.59600	-7.00400	38.38900	-60.70800	-9.36100	28.81600	-44.77300	-9.83600
H	43.19500	-60.86200	-8.05400	38.84600	-60.13000	-7.06500	28.35800	-44.78700	-8.38000
H	44.33800	-60.21300	-4.78700	41.07100	-59.21500	-6.56700	26.11200	-47.28200	-6.69400
H	45.05100	-61.60900	-3.80100	48.50000	-57.27300	-10.20700	24.55400	-47.44400	-7.50100
H	43.38100	-61.14100	-3.63800	49.90100	-57.74900	-11.94900	25.46700	-45.87200	-7.08900
H	42.43700	-64.49500	-5.21900	48.19200	-57.78600	-14.10600	26.28100	-45.73800	-11.04500
H	43.86500	-64.35000	-4.18400	46.59600	-55.92300	-14.28600	24.64400	-46.16800	-10.42500
H	42.48600	-63.39000	-3.87400	46.33200	-54.41600	-12.39000	25.64300	-44.96100	-9.75400
H	46.38300	-55.19900	-17.88100	38.47800	-62.21000	1.09300	29.96400	-56.07300	-7.71700
H	46.05600	-56.68400	-19.84300	40.09400	-62.73400	1.19400	30.61500	-58.15200	-8.92800
H	47.67500	-58.70100	-20.13500	39.10300	-63.62400	0.26400	28.52800	-59.77000	-9.07000
H	49.66300	-58.77900	-18.72600	39.07200	-59.38800	0.03800	27.11300	-60.02000	-7.04700
H	49.70100	-57.58300	-16.58000	40.99500	-59.65700	0.26700	26.98100	-58.13900	-5.52400
H	42.25300	-59.74500	-11.61500	40.23500	-59.22500	-1.18600	29.25800	-52.23000	-7.48700
H	41.07700	-61.84300	-11.40100	39.63200	-60.39200	-3.47500	28.80100	-50.13500	-8.91200
H	42.06800	-63.65200	-12.97500	38.74100	-59.31700	-2.74500	30.49400	-48.44400	-8.94000
H	43.74400	-63.06700	-14.85200	34.93200	-59.34000	-3.61400	31.94900	-48.20500	-6.83700
H	44.48200	-60.81600	-14.93000	36.13600	-58.94600	-2.27800	32.29300	-50.06000	-5.39600
H	43.41900	-58.05700	-17.17900	35.46000	-60.50700	-2.60900	31.49000	-54.94400	-8.10300
H	43.63500	-59.07200	-15.92500	37.77300	-57.44200	-4.30400	31.01100	-53.41600	-8.34300
H	50.81700	-57.35300	-10.63200	36.74500	-58.09800	-5.62600	25.64700	-51.81400	-3.51800
H	51.56900	-58.23800	-8.67600	38.37000	-58.39900	-5.65600	23.16900	-51.37600	-2.90900
H	50.27300	-57.86100	-6.49100	37.53700	-61.06200	-1.73100	22.43900	-51.39400	-0.64300
H	48.16200	-56.49200	-6.66500	38.33300	-62.30100	-2.58900	24.24200	-51.68000	1.23200
H	47.45100	-55.58200	-8.89000	47.64500	-57.25100	1.52900	26.24900	-52.91600	0.39300

H	38.68900	-59.11200	-20.72900	46.24400	-56.70600	1.84200	37.62500	-55.69300	-10.69000
H	39.78300	-57.99200	-20.64700	47.47600	-55.76100	1.41300	36.15700	-55.90300	-11.47900
H	43.95900	-55.67500	-8.23300	45.72000	-56.08900	-0.53400	32.20900	-51.43500	-0.16400
H	45.37000	-55.96300	-6.15300	47.12500	-56.85600	-0.93700	32.64800	-49.42300	1.17100
H	45.98400	-58.20300	-5.49500	42.95000	-60.80700	4.13000	30.90100	-48.66400	2.81800
H	46.30300	-59.94800	-7.37500	41.29500	-60.89200	4.14200	28.78900	-49.73800	2.38900
H	44.60100	-59.76600	-9.23900	41.99300	-59.43700	3.68100	28.07600	-50.64200	0.07300
H	43.92200	-56.08200	-17.79700	42.32600	-62.26800	2.15200	31.91200	-56.62600	-6.80900
H	41.65800	-55.53000	-18.84000	41.35700	-61.15800	1.65700	34.02200	-57.74500	-7.75800
H	40.29100	-53.90300	-17.86700	46.66800	-62.30300	-5.48500	36.08500	-57.64200	-6.52200
H	40.94600	-52.70500	-15.72300	47.58300	-63.54400	-5.85300	35.92800	-57.15900	-3.97600
H	42.98300	-53.44700	-14.63000	46.33500	-63.87100	-4.81700	34.00000	-55.97800	-3.06000
H	47.26500	-53.39700	-10.46700	48.46200	-61.87600	-4.14900	28.72500	-59.02700	-1.91600
H	49.02600	-53.87900	-8.83400	48.21800	-63.38700	-3.27200	27.33400	-60.41900	-0.46200
H	51.41800	-53.18700	-9.24700	42.88500	-61.28200	-4.81700	27.49600	-59.78300	2.13100
H	52.02600	-52.90800	-11.54300	42.66500	-59.92900	-5.90500	28.91000	-57.99600	2.81200
H	50.51000	-53.40200	-13.27000	42.17900	-59.79100	-4.48400	29.90000	-56.48400	1.13000
H	35.83600	-62.38100	-17.45400	44.22300	-66.08000	-3.18500	36.52200	-51.26600	-12.97600
H	35.69300	-61.23900	-18.86600	45.28000	-67.22900	-2.73000	36.98100	-50.13500	-12.13100
H	39.03300	-61.26900	-11.11400	45.95700	-65.58100	-2.97300	36.41300	-47.54200	-7.60600
H	37.68200	-61.60800	-11.52000	50.03700	-58.67600	-0.16800	36.55000	-47.85800	-6.05400
H	46.39000	-60.90800	-15.87900	51.18600	-59.37800	-1.14600	27.42600	-51.07800	-6.54900
H	47.90300	-61.85700	-17.53700	49.71600	-59.37100	-1.78400	25.78200	-49.51900	-6.43500
H	50.16300	-62.60100	-16.64000	47.37200	-62.56900	4.58100	23.34300	-50.28000	-5.94300
H	51.14500	-61.37400	-14.73500	47.53200	-63.77100	3.22000	22.93200	-52.71200	-5.43100
H	49.67700	-60.13800	-13.24200	47.98200	-62.32100	2.99700	24.99600	-54.28200	-5.53900
H	37.44700	-56.98000	-21.49900	36.31500	-69.59900	-7.60300	36.60600	-57.45100	-9.04100
H	36.91500	-57.27300	-20.09300	37.73300	-71.57300	-7.78400	35.39900	-57.70700	-9.89600
H	37.96100	-56.24400	-19.88400	38.88700	-72.48100	-6.01300	36.63600	-58.01900	-10.70700
H	37.53700	-63.52400	-18.29900	38.56200	-71.58200	-3.64000	38.88700	-52.57500	-12.69400
H	36.29600	-63.55300	-19.38000	37.68300	-69.28900	-3.62500	38.84400	-51.21000	-13.67400
H	37.80100	-62.43800	-19.50400	44.58400	-58.78800	-4.99600	39.39900	-51.31700	-12.13200
H	43.22700	-59.85800	-18.83100	45.09200	-60.11400	-5.25200	31.86000	-53.70700	-10.86500
H	44.61900	-60.01400	-18.03300	49.99000	-60.85000	0.66700	32.28800	-55.35900	-10.34300
H	43.17200	-60.88600	-17.94500	49.95300	-61.58000	-0.75700	30.77100	-54.77400	-10.53400
H	37.01100	-58.92600	-10.96800	35.49000	-63.67000	-9.50600	34.67300	-46.66900	-5.99300
H	37.41500	-59.87200	-9.69200	34.22000	-63.59900	-11.60700	33.81900	-47.78100	-6.84200
H	38.31600	-58.74500	-10.60800	31.97900	-62.43000	-11.62300	34.21800	-48.31400	-5.52300
H	40.82400	-63.35400	-15.33700	30.88600	-62.19300	-9.39000	32.36500	-50.42800	-11.07500
H	39.75600	-63.37600	-16.44600	32.05400	-62.55200	-7.23900	32.68100	-49.02200	-10.20200
H	41.75900	-63.59400	-17.40800	33.25400	-63.88400	-2.13500	33.41100	-49.11900	-12.67600
H	42.42000	-62.12100	-17.01400	32.20900	-63.70200	0.19800	34.58400	-50.13800	-12.23700
H	41.05400	-62.34900	-18.07700	33.46600	-62.33500	1.91100	34.69100	-48.75500	-11.51300
H	39.24700	-54.99000	-16.42200	35.97500	-62.37400	1.70200	37.96300	-56.13600	-7.29200
H	37.88500	-54.58700	-15.52000	36.75000	-62.17800	-0.77600	37.15700	-55.88600	-6.13100

H	37.55700	-53.64300	-17.80700	40.80200	-63.72000	-3.71800	39.08900	-54.95600	-4.79700
H	36.29700	-54.85900	-17.40600	43.13600	-64.57000	-4.21800	40.03600	-55.02000	-6.11200
H	37.36700	-55.25000	-18.43800	44.48400	-63.85600	-5.97600	39.24800	-56.40800	-5.16700
H	34.52500	-60.30500	-15.68900	43.24500	-63.33800	-8.18900	40.36900	-51.37800	-7.37800
H	34.27600	-60.28500	-14.26200	40.69300	-63.09000	-7.95900	40.72200	-51.17100	-8.82700
H	33.89500	-57.91000	-16.07000	45.37600	-61.56000	3.39500	39.61500	-48.83900	-7.08100
H	33.48500	-57.72700	-14.41400	44.99900	-62.89900	3.78500	40.12300	-48.47900	-8.60000
H	32.52000	-58.58400	-15.15300	37.68200	-64.32500	-9.93000	41.22400	-48.93500	-7.39900
H	40.65200	-53.39100	-10.89800	38.04400	-65.06400	-12.43000	33.97200	-53.46400	-2.36400
H	41.86900	-54.65700	-11.12200	39.08400	-67.35900	-12.63800	35.47800	-53.17900	-1.89400
H	42.00600	-49.91000	-11.63000	38.56900	-68.95600	-10.88700	31.41900	-53.25600	1.45700
H	43.67000	-49.78400	-10.92300	37.05500	-68.38300	-9.01300	32.66100	-53.25800	2.60100
H	42.24000	-50.59100	-10.27000	44.15100	-66.13200	-0.63700	31.96400	-54.79100	2.05200
H	43.55400	-50.96000	-14.24500	45.71000	-65.91500	-0.56800	35.33100	-53.94100	2.04800
H	44.75600	-52.32200	-13.96600	40.34500	-64.49300	-2.20000	35.77800	-54.59500	0.50100
H	44.84100	-50.71700	-13.25600	42.27100	-65.10200	-1.07800	34.78100	-55.43100	1.76400
H	40.99600	-52.51500	-13.03900	42.81400	-67.41700	-0.32200	33.16000	-51.74800	-0.69600
H	42.21100	-53.63500	-13.38600	41.71300	-69.24800	-1.92600	34.50400	-51.68500	-0.01500
H	39.82900	-56.99100	-10.61100	39.28100	-68.45200	-2.63900	32.33500	-50.78700	-3.69400
H	38.29100	-56.64600	-11.23200	31.33800	-65.02800	-3.29400	33.37300	-49.61900	-4.18300
H	38.60400	-55.47600	-10.27200	29.88000	-66.40700	-1.70900	32.69500	-49.66000	-2.59300
H	39.20200	-53.56600	-14.17000	29.81800	-68.72100	-2.11700	36.89800	-50.33400	-2.15800
H	38.03600	-53.52500	-12.73800	31.27000	-69.61900	-3.88200	35.82000	-49.42600	-1.78600
H	38.03900	-54.71700	-13.85500	33.60700	-68.47500	-3.94400	36.18200	-49.36100	-3.47700
H	28.02600	-55.69300	-1.21000	26.50700	-59.08400	-0.39400	46.57500	-38.55400	-5.91200
H	30.19700	-56.31300	-0.50600	27.34300	-58.04600	-1.59000	44.86400	-37.27700	-6.99700
H	31.54300	-57.89800	-1.96700	28.24800	-59.14400	-0.85000	42.41100	-37.17200	-6.00200
H	30.38700	-59.27100	-3.75300	25.36900	-58.57900	-3.66200	41.91100	-38.63800	-4.16900
H	28.16300	-58.71700	-4.28800	25.22500	-60.11600	-4.42000	43.50000	-40.37900	-3.39100
H	22.38800	-56.79600	-9.00700	24.56700	-59.93100	-3.04600	43.55000	-45.73700	-7.51300
H	22.25500	-56.87000	-11.32300	25.10800	-62.32600	-2.31000	41.61800	-47.37300	-7.03300
H	24.18400	-56.14300	-12.80000	25.97100	-62.64600	-3.69200	42.00000	-48.72400	-5.02700
H	26.37300	-55.44700	-11.56900	26.16400	-65.87400	-0.14800	44.28300	-49.78100	-4.74500
H	26.48400	-55.58600	-9.06000	27.33800	-64.59900	0.03100	46.18500	-48.18000	-5.04400
H	18.02800	-47.95600	-9.64300	25.67600	-64.33100	0.43700	52.20600	-52.14700	-2.80600
H	19.08600	-47.62400	-10.73300	23.58500	-65.17600	-1.64300	52.23400	-50.48800	-2.40700
H	21.21300	-54.56900	-0.81600	24.57600	-66.56500	-2.00400	48.93400	-40.87900	-9.30200
H	19.60000	-56.07200	0.19100	23.96800	-65.22600	-3.12000	51.24200	-39.87800	-9.84800
H	18.48500	-57.66300	-1.39200	27.37300	-61.96400	-1.14300	53.18200	-41.49700	-10.05000
H	18.25400	-56.93400	-3.72500	28.13500	-62.28700	-2.44400	52.79800	-43.90200	-9.84400
H	20.32200	-55.90800	-4.68000	28.90200	-51.14400	-2.11900	50.69400	-44.52500	-8.19100
H	15.56700	-53.92100	-9.07200	27.58400	-51.83400	-2.18000	52.95600	-50.14100	-7.25800
H	15.07200	-52.52400	-9.42800	27.91500	-51.00700	-0.67200	52.14100	-48.78600	-7.61300
H	20.33400	-52.63900	-7.02800	28.01100	-53.19600	-0.22000	50.93600	-45.75500	-7.94400
H	19.16500	-50.23100	-7.46600	29.56800	-52.80000	-0.16800	53.44400	-46.07700	-7.99100

H	19.65200	-48.55300	-5.76800	31.95200	-56.40000	1.01700	54.48100	-47.12000	-6.00100
H	21.61700	-48.75100	-4.21500	30.76400	-57.10700	1.59700	53.11100	-47.50500	-4.02000
H	22.28000	-50.90300	-3.75900	32.04100	-57.92400	1.45500	50.56900	-47.19200	-4.03700
H	27.56100	-55.00300	-8.64500	31.84400	-58.34500	-0.99800	43.02900	-45.54000	-4.26700
H	28.46500	-53.69400	-10.55000	30.47300	-58.72800	-0.15200	41.52200	-46.77500	-2.95300
H	29.73000	-51.76700	-10.35600	28.80000	-55.71000	-9.11400	41.59600	-46.89800	-0.46500
H	30.08200	-50.92300	-8.12400	27.55600	-54.89400	-9.69600	43.77800	-46.02200	0.55700
H	28.97600	-51.92200	-6.19800	28.82100	-54.12400	-8.94000	44.88200	-44.08100	-0.59800
H	23.79300	-52.39200	0.08600	26.85000	-54.32800	-7.25700	50.94300	-40.32200	-2.63500
H	24.52500	-52.06200	2.26400	26.73900	-55.80300	-7.48600	51.40800	-38.30700	-1.72000
H	26.18200	-53.63800	3.31800	25.18900	-57.42600	-6.06100	49.81800	-36.29700	-2.14300
H	26.91400	-55.65700	2.13700	23.79800	-56.93900	-5.46400	48.15100	-36.49700	-4.04500
H	25.96900	-55.99900	-0.10900	25.05700	-57.78400	-4.63000	47.98800	-38.69300	-5.19600
H	15.61200	-52.53200	-13.72100	32.65700	-58.06600	-8.22300	49.95100	-55.83100	-6.83500
H	16.62000	-51.49200	-14.53600	33.41900	-58.25800	-6.75500	48.69600	-55.99200	-7.49600
H	22.63200	-51.84600	-15.11100	33.25200	-59.52300	-7.63700	44.55700	-51.70400	-4.34600
H	23.96600	-52.25900	-14.36400	33.77200	-51.67000	-3.63000	45.05800	-51.15800	-3.01700
H	22.66900	-58.94100	-3.08600	33.53700	-52.94800	-4.45500	47.01700	-41.64800	-10.02200
H	20.79700	-60.64400	-3.52200	33.16300	-53.00100	-2.82100	46.73500	-39.35400	-10.55000
H	20.19900	-61.04000	-5.85200	34.53100	-56.39200	-1.86800	44.49400	-38.49400	-10.28400
H	20.90200	-59.52400	-7.66300	35.78500	-56.20600	-2.67700	42.92700	-39.27400	-8.60800
H	22.54400	-57.67300	-7.24500	34.89900	-54.85500	-2.63100	43.68700	-41.37000	-7.35100
H	16.85400	-51.65900	-7.59900	26.54100	-63.88400	-11.63100	51.41300	-49.51300	-9.88600
H	16.94300	-53.25200	-7.17500	27.13300	-63.15200	-14.37300	52.34000	-50.94900	-9.58200
H	15.66300	-52.74600	-6.93000	29.61900	-63.92300	-15.46300	52.95700	-49.40900	-9.72700
H	15.94200	-54.33400	-15.08400	31.68400	-64.70800	-13.53400	50.46200	-56.78500	-8.95500
H	15.53800	-53.02500	-16.16600	30.93800	-65.41100	-10.98000	49.74300	-55.63300	-9.72900
H	17.05800	-53.78800	-15.90100	24.89900	-55.39500	-3.82300	51.03000	-55.08200	-9.14700
H	20.14300	-48.87000	-8.18100	25.07500	-54.81000	-5.27700	53.60700	-51.61500	-4.73400
H	20.93900	-48.72700	-9.22300	31.66600	-51.27200	-4.69800	53.70400	-50.01100	-4.55900
H	20.42800	-47.45300	-8.66200	31.13300	-51.53200	-3.10300	54.46100	-50.87800	-3.61600
H	22.69100	-49.69500	-13.56800	30.40700	-69.11500	-6.39800	45.02600	-53.30100	-1.97200
H	24.11700	-49.93600	-14.45500	30.32500	-71.26000	-5.84200	44.36000	-53.99900	-3.33000
H	24.00700	-50.15600	-12.92400	28.13600	-72.53700	-5.88600	43.51300	-52.98100	-2.38300
H	19.92200	-49.31300	-15.35100	26.28000	-71.63400	-7.27900	49.73300	-54.42500	-1.99900
H	19.56400	-50.59500	-15.98600	26.41200	-69.05100	-7.77200	49.16200	-55.57400	-2.93200
H	17.46300	-49.12200	-16.17300	30.38100	-63.30700	-2.61000	51.58600	-56.07200	-3.73900
H	17.01800	-50.21500	-15.35300	31.14800	-61.54200	-1.18000	52.07700	-54.66000	-3.05300
H	17.47300	-48.99200	-14.40300	32.52600	-59.50000	-2.30000	51.43900	-55.98800	-2.20300
H	19.59000	-56.07300	-7.83200	33.23500	-59.72700	-4.50500	46.80700	-47.71500	-8.02400
H	20.70900	-54.94400	-7.50800	32.26900	-61.39000	-6.04600	48.06600	-47.83000	-8.89000
H	18.71000	-53.46500	-6.85600	24.84400	-60.51100	-6.94100	46.91600	-49.97800	-10.21600
H	17.63900	-54.44700	-7.49800	23.34700	-59.22100	-5.54500	45.59300	-49.75900	-9.63500
H	18.38900	-54.88400	-6.15500	21.30600	-60.31500	-4.49900	46.11500	-48.51900	-10.38500
H	20.97000	-54.84100	-14.67600	21.40500	-62.88000	-4.30200	45.06600	-52.79000	-9.05200

H	22.02900	-55.86200	-13.96400	22.88200	-64.12100	-5.65100	44.82400	-53.90500	-8.04200
H	19.22300	-56.28200	-14.68800	34.58600	-56.32300	-4.83100	44.16900	-51.07500	-7.47200
H	20.39700	-57.75800	-14.08500	34.26600	-57.47400	-4.05000	43.85900	-52.10200	-6.36000
H	20.40100	-57.22300	-15.39300	26.51900	-68.07800	-8.98100	42.85700	-52.25200	-7.50500
H	23.70900	-49.14000	-7.51200	25.10100	-69.57900	-10.10500	47.03700	-46.44600	-2.23000
H	23.83000	-50.63700	-6.89400	23.74000	-68.99100	-12.00000	46.03100	-46.47100	-0.79600
H	26.15100	-46.82500	-5.68000	23.22700	-66.52200	-12.32200	48.81000	-46.09000	1.94100
H	24.52400	-47.24700	-5.19800	24.30200	-64.98700	-10.51600	50.25700	-46.13300	0.90100
H	25.75200	-47.18100	-3.99900	31.28100	-59.68000	-6.06000	49.99700	-44.82000	1.63300
H	28.21600	-49.11600	-4.24900	30.55200	-59.35800	-7.48100	47.45900	-42.83800	0.64800
H	28.32600	-50.01200	-5.66200	29.41400	-59.95100	-9.47500	46.28300	-43.54500	-0.73100
H	28.23400	-48.28800	-5.70200	27.94700	-58.72400	-10.97700	46.52100	-43.79400	1.14900
H	26.36200	-50.69100	-7.53800	25.81400	-57.69000	-10.04400	47.67800	-46.95500	0.47100
H	26.31900	-49.18800	-7.99100	25.17300	-57.95400	-7.87700	48.86900	-47.12300	-0.65700
H	24.18800	-50.35700	-11.66300	26.26500	-59.99800	-6.58000	46.37600	-51.04200	-0.86900
H	25.26500	-49.27400	-10.70800	32.00600	-67.58600	-6.24200	46.05600	-50.06000	0.12500
H	23.60700	-48.97000	-10.46200	33.36400	-69.47100	-7.40900	47.87500	-50.14700	-0.31400
H	25.03900	-53.19100	-9.09600	34.62800	-68.77800	-9.56900	44.55000	-48.15500	-2.75900
H	26.23600	-52.19100	-9.67100	34.09300	-66.64000	-10.66300	44.26000	-48.97000	-1.21100
H	24.96000	-52.73100	-10.62200	32.80400	-65.07000	-9.55900	44.67000	-49.91600	-2.53400

Table S3. Atomic coordinates of systems consisted of four molecules of Ph_7T_8-T_8Et_7 compound for clusters 7 and 8.

Atomic coordinates of systems consisted of four molecules of Ph_7T_8-T_8Et_7 compound						
	Cluster **7**			Cluster **8**		
Atom	x	y	z	x	y	z
Si	42.81700	-33.08600	-5.94900	53.79900	-70.12200	1.93800
Si	45.68400	-36.30500	-4.60100	50.00600	-68.84100	-0.02400
Si	44.90800	-38.52600	-3.08700	49.24000	-67.36700	-2.46200
Si	44.15900	-41.44100	-2.95900	49.62100	-67.28200	-5.44900
Si	44.29000	-41.44500	0.04600	48.64600	-64.51200	-5.84000
Si	47.11000	-42.61300	-0.17600	45.88900	-65.82300	-6.21800
Si	46.94000	-42.47800	-3.25600	46.93700	-68.65800	-5.98900
Si	44.89600	-38.58800	-0.15900	48.17700	-64.52300	-2.94200
Si	47.76500	-39.69600	0.03800	45.33000	-65.49300	-3.23800
Si	47.75400	-39.65000	-3.03800	46.44600	-68.24800	-2.98200
Si	39.06500	-43.06600	-0.30400	-6.88000	-61.22100	-5.27400
Si	35.47400	-41.61500	1.93900	-4.61300	-58.66100	-2.31200
Si	32.85900	-43.13000	1.91000	-1.98700	-58.01900	-3.41700
Si	30.48800	-41.98300	0.33900	0.72800	-58.90500	-3.54200
Si	29.48300	-40.45300	2.77100	1.75200	-56.84700	-1.53000
Si	27.82800	-43.13600	3.41300	2.34100	-55.19300	-3.93900
Si	28.98600	-44.56200	1.03100	1.35700	-57.20300	-5.98900

Si	31.68500	-41.65900	4.32700	-1.10800	-55.85700	-1.64900
Si	30.13900	-44.22100	5.20300	-0.53400	-54.15200	-3.90000
Si	31.29300	-45.53300	2.67000	-1.44500	-56.28200	-5.84300
Si	37.10600	-54.56600	3.70000	-8.77500	-56.82400	-14.78800
Si	36.72400	-52.48500	-0.32100	-10.05000	-60.09000	-11.94900
Si	34.88500	-51.62400	-2.49400	-9.12100	-62.79100	-12.32500
Si	33.62000	-48.96600	-2.58000	-8.10500	-64.41700	-14.64400
Si	31.73400	-50.09700	-4.72800	-10.51800	-65.87500	-14.75000
Si	33.60500	-49.09700	-6.87600	-9.61200	-67.96400	-12.78900
Si	35.43900	-48.02600	-4.79300	-7.07900	-66.32500	-12.68000
Si	33.09800	-52.78700	-4.50800	-11.65000	-64.42500	-12.47000
Si	34.99600	-51.76400	-6.85800	-10.47000	-66.44600	-10.43700
Si	36.85400	-50.76700	-4.61400	-7.97500	-64.68300	-10.30700
Si	-2.75200	-41.96700	0.26000	37.57200	-64.87600	-0.50300
Si	-4.91400	-44.45300	-2.95500	37.17000	-68.59600	-3.19500
Si	-3.96400	-44.67600	-5.78300	36.41600	-71.38900	-2.65100
Si	-1.27700	-45.54100	-7.04500	33.89200	-72.56500	-1.48000
Si	-1.97400	-43.78800	-9.48200	35.48500	-75.12000	-1.00100
Si	-3.53700	-46.19300	-10.41400	34.87100	-76.07100	-3.87500
Si	-2.89100	-47.88900	-8.16300	33.28300	-73.48900	-4.31800
Si	-4.56700	-42.96400	-8.20800	37.98100	-73.77800	-2.00600
Si	-6.16300	-45.28200	-9.50500	37.39400	-74.73100	-4.94800
Si	-5.49800	-46.98500	-6.81200	35.71600	-72.26600	-5.50900
Si	41.72600	-35.35300	-7.66800	54.55600	-70.84400	-0.83400
Si	43.99600	-36.43200	-9.32300	52.44900	-72.87300	-1.50500
Si	42.19100	-36.89100	-11.60900	54.42800	-74.38200	-3.29800
Si	41.43100	-39.65000	-10.66100	53.61500	-72.58100	-5.56700
Si	43.38900	-39.14200	-8.31900	51.66900	-71.16800	-3.88900
Si	39.90000	-35.68600	-9.93300	56.52100	-72.38100	-2.57000
Si	39.19500	-38.61900	-8.94000	55.67200	-70.54300	-4.87300
Si	41.13100	-38.15000	-6.68400	53.59400	-69.10900	-3.22600
Si	37.34100	-42.17100	-2.70300	-4.48200	-63.22600	-4.70200
Si	34.41300	-42.67900	-3.29300	-1.77700	-62.04000	-5.18500
Si	34.77500	-41.90400	-6.19000	-0.73600	-64.56100	-6.10200
Si	34.11300	-38.95900	-5.76100	0.18800	-65.46400	-3.39900
Si	33.60200	-39.78800	-2.90600	-0.84500	-62.74600	-2.40800
Si	37.66500	-41.24100	-5.53200	-3.39800	-66.02300	-5.51800
Si	36.92400	-38.37900	-4.98700	-2.50200	-66.78600	-2.63700
Si	36.51300	-39.42100	-2.19100	-3.59300	-64.15500	-1.90800
Si	35.31700	-52.24700	4.46900	-6.01500	-56.94700	-14.10100
Si	37.43700	-50.09200	4.07200	-5.30400	-58.09100	-11.36200
Si	36.51300	-49.47100	6.92200	-2.62300	-56.91300	-11.67500
Si	34.26700	-47.80500	5.97300	-1.54900	-59.27000	-13.18900
Si	35.14100	-48.28600	3.16100	-4.29200	-60.58600	-12.95600
Si	34.56700	-51.92800	7.34300	-3.30900	-55.71200	-14.46100

Si	32.40000	-50.21700	5.91200	-2.21900	-58.18100	-15.93800
Si	33.21700	-50.63500	3.08400	-5.06600	-59.36000	-15.52100
Si	-3.13500	-39.52600	-1.55100	39.47500	-66.80300	0.74900
Si	-2.98300	-39.60100	-4.68000	39.19900	-69.71200	1.31400
Si	-3.36800	-36.65100	-4.35700	41.34900	-69.52500	3.36200
Si	-6.23700	-36.96200	-4.89600	43.30400	-69.80800	1.14200
Si	-5.93800	-40.02200	-4.96400	41.14100	-70.27400	-0.94900
Si	-3.45400	-36.58400	-1.31400	41.48600	-66.43200	3.07100
Si	-6.49800	-37.03700	-1.87500	43.61500	-66.85700	0.73800
Si	-6.14500	-39.92600	-1.91800	41.56300	-67.26200	-1.43400
O	41.74800	-34.14100	-6.57200	54.50300	-69.94900	0.51500
O	40.31900	-35.35900	-8.41600	55.92000	-71.09900	-1.76900
O	42.89000	-35.34300	-8.74500	53.73800	-72.27000	-0.73200
O	41.95900	-36.75300	-6.78800	53.64000	-69.87800	-1.84200
O	41.32300	-35.81600	-10.78600	55.56800	-73.66700	-2.38500
O	42.05200	-39.30400	-7.39400	52.52800	-69.86500	-4.20200
O	40.21300	-39.79100	-9.52900	54.61500	-71.32900	-5.81700
O	43.59100	-36.93100	-10.81700	53.05300	-73.87100	-2.68700
O	43.99800	-37.64900	-8.24100	51.72800	-71.71800	-2.37600
O	41.41600	-38.27200	-11.47400	54.48600	-73.74800	-4.79100
O	42.90600	-39.45600	-9.87200	52.29400	-72.24800	-4.84400
O	39.38900	-37.25400	-9.82300	56.37000	-71.80700	-4.12500
O	39.68400	-38.07200	-7.46800	55.04900	-69.47400	-3.82700
O	44.55900	-37.29100	-3.96700	50.19700	-68.00900	-1.35900
O	44.22700	-38.63200	-1.62100	49.26800	-65.76000	-2.85100
O	44.68300	-40.05600	-3.68100	49.62200	-67.84300	-3.96600
O	46.48600	-38.58300	-2.95700	47.66000	-67.61600	-2.09400
O	44.57900	-39.99100	0.65200	48.04900	-63.88800	-4.41500
O	47.23800	-40.98700	-3.74400	47.02800	-68.78000	-4.39800
O	47.38300	-41.21600	0.60000	45.37500	-65.16700	-4.81300
O	43.58500	-41.28100	-1.44400	49.70100	-65.65000	-5.47300
O	45.36800	-42.48100	-2.95800	48.41200	-67.99400	-6.28700
O	45.53100	-42.46700	-0.10700	47.45200	-65.43900	-6.51500
O	47.62600	-42.47700	-1.73100	45.86800	-67.45300	-6.11800
O	46.53800	-38.54400	-0.08600	46.76400	-65.20800	-2.53800
O	48.28500	-39.98400	-1.48200	45.34500	-67.11000	-3.25200
O	38.27400	-43.06600	-1.75000	-5.90800	-62.46600	-4.87200
O	37.54700	-42.29900	-4.29800	-3.97000	-64.48100	-5.53900
O	35.76700	-42.34500	-2.50400	-3.31400	-62.13000	-4.62500
O	37.56700	-40.58800	-2.36700	-4.45500	-64.08500	-3.29200
O	36.29000	-41.25400	-6.39600	-1.82800	-65.81000	-5.89300
O	34.96400	-39.89600	-1.99400	-2.44200	-63.00100	-2.13000
O	35.71500	-38.66300	-5.97800	-0.96600	-66.62500	-3.22600
O	34.74700	-42.88800	-4.87600	-1.56700	-63.20700	-6.26500
O	33.55500	-41.31500	-3.24400	-0.75800	-62.29900	-3.95200

O	33.82900	-40.58000	-5.89100	0.17800	-64.73600	-4.87400
O	33.81700	-38.68700	-4.15400	-0.22900	-64.26800	-2.35900
O	37.90300	-39.72500	-5.02500	-3.37600	-66.62800	-3.97100
O	36.49300	-38.26000	-3.38600	-2.87200	-65.59400	-1.63500
O	34.37700	-42.80200	1.52500	-3.40500	-58.76300	-3.39400
O	32.45900	-41.94200	2.90800	-1.50100	-57.37700	-2.08800
O	31.89800	-42.83600	0.62400	-0.83400	-58.95600	-4.00100
O	32.58300	-44.52700	2.73500	-2.20200	-56.89500	-4.52900
O	30.34500	-40.75500	4.05200	0.51600	-55.74300	-1.27200
O	30.42600	-45.17600	1.35200	-0.23100	-57.26500	-6.37200
O	28.99200	-43.32800	4.51200	1.03400	-54.28400	-3.56200
O	30.49500	-40.78900	1.50100	0.93500	-58.10300	-2.09000
O	29.25600	-43.04100	0.38200	1.55800	-58.22500	-4.76100
O	28.42800	-41.73700	2.80100	2.52000	-56.44500	-2.89400
O	28.06600	-44.41000	2.39200	1.89100	-55.83900	-5.30300
O	31.32100	-43.07100	5.11500	-1.58100	-54.71200	-2.73600
O	30.31500	-45.25700	3.95100	-0.80400	-54.88600	-5.31600
O	35.75100	-53.72200	4.11500	-7.45900	-56.39500	-14.01900
O	34.50400	-52.60100	5.88400	-4.82500	-56.17900	-14.85600
O	36.72400	-51.47200	4.56400	-5.74300	-56.95200	-12.47800
O	34.63600	-51.41000	3.26700	-6.06900	-58.43600	-14.69500
O	35.84900	-50.94900	7.11400	-3.15200	-55.81300	-12.82300
O	33.64500	-49.00800	2.91700	-4.53600	-60.48800	-14.48600
O	32.94200	-48.70900	5.66500	-1.63800	-59.24200	-14.81500
O	37.39500	-49.43800	5.56300	-3.80300	-57.72900	-10.94800
O	36.43300	-49.37900	3.03000	-5.24300	-59.45100	-12.31000
O	35.23500	-48.44300	7.00100	-1.56600	-57.81500	-12.48800
O	35.14500	-47.66200	4.66000	-2.71800	-60.27000	-12.66000
O	33.20200	-50.99200	7.15600	-2.29000	-56.80600	-14.99700
O	32.59100	-51.07400	4.56300	-3.81600	-58.50500	-16.17900
O	35.44500	-52.47300	-1.24500	-8.95200	-61.16900	-12.40500
O	34.49000	-52.71000	-3.66200	-10.58100	-63.38000	-11.86100
O	33.73600	-50.52500	-2.17000	-9.00600	-63.30300	-13.92900
O	36.29000	-50.88000	-3.02300	-7.99800	-63.52900	-11.44700
O	32.12500	-51.57400	-4.02900	-11.30400	-64.66600	-14.01400
O	36.49000	-49.21700	-5.07400	-7.44000	-66.02200	-11.13000
O	34.80200	-50.17500	-7.18500	-9.62700	-67.63900	-11.21000
O	32.60700	-49.08900	-3.82100	-9.01700	-65.53000	-15.24800
O	35.00300	-48.33200	-3.19600	-7.02100	-65.03200	-13.57800
O	32.38700	-50.05200	-6.19900	-10.62000	-67.20300	-13.77700
O	34.13400	-48.08400	-5.73200	-8.29700	-67.19000	-13.25800
O	33.75500	-52.19300	-5.90000	-11.44400	-65.78900	-11.57900
O	36.12200	-51.79700	-5.63200	-9.46600	-65.24500	-9.91800
O	-2.35900	-40.65100	-0.64100	38.16200	-65.90800	0.53500
O	-2.58000	-37.95100	-1.34100	40.12200	-66.59000	2.24000

O	-3.23900	-40.02000	-3.12700	38.90000	-68.29300	0.59100
O	-4.71500	-39.69000	-1.17400	40.60100	-66.57700	-0.37800
O	-3.48000	-36.11300	-2.83600	41.89900	-67.99200	3.45600
O	-5.86900	-40.42700	-3.41100	40.90300	-68.76500	-1.38700
O	-6.25400	-36.45400	-3.34900	43.89300	-68.29600	1.41600
O	-2.63800	-38.10600	-4.26200	39.87500	-69.40300	2.74300
O	-4.40900	-39.82600	-5.38500	40.14800	-70.56600	0.32400
O	-4.68200	-36.94800	-5.31100	42.48600	-70.28400	2.46700
O	-6.64300	-38.51600	-4.99800	42.52600	-69.92600	-0.27200
O	-5.07200	-37.00000	-1.04600	42.42300	-66.19600	1.70900
O	-6.97800	-38.54300	-2.14100	43.09700	-67.19300	-0.78400
O	-4.00700	-44.13200	-4.30100	36.70800	-69.82200	-2.25300
O	-3.95200	-43.36200	-6.70300	37.25000	-72.42000	-1.70300
O	-2.55600	-45.46400	-6.04000	34.86900	-71.52500	-2.21300
O	-5.27300	-45.50900	-6.18500	36.51100	-71.56600	-4.28300
O	-3.45200	-43.04600	-9.40300	37.06000	-74.98600	-1.41700
O	-4.02600	-47.69300	-7.03100	34.18100	-72.31300	-4.99000
O	-4.85500	-45.27700	-10.50800	36.45400	-75.82700	-4.25200
O	-1.57300	-44.26600	-8.00800	34.98800	-73.60700	-0.73600
O	-1.67300	-46.91400	-7.75500	33.08800	-73.36800	-2.68500
O	-2.28000	-45.23400	-10.12000	34.65000	-75.64300	-2.31300
O	-3.59200	-47.59300	-9.61200	33.90100	-74.97400	-4.62700
O	-5.72300	-44.01700	-8.61100	38.00800	-73.93200	-3.64300
O	-5.81200	-46.39800	-8.31800	36.22300	-73.76800	-5.52000
C	36.44700	-38.44700	-8.99300	57.25500	-69.85700	-6.98700
C	35.41200	-38.62400	-9.91000	58.54400	-70.02000	-7.52400
C	35.27000	-39.86700	-10.59900	59.67200	-69.80900	-6.70000
C	36.27100	-40.91100	-10.44400	59.49500	-69.65600	-5.34700
C	37.34600	-40.65300	-9.51900	58.23000	-69.55100	-4.73400
C	45.33900	-40.51300	-7.07800	48.76700	-71.50100	-3.51200
C	46.38800	-41.42200	-7.34400	47.56200	-72.10700	-3.85100
C	45.97900	-42.69000	-7.97600	47.27500	-72.50300	-5.12300
C	44.71700	-42.93600	-8.50700	48.29700	-72.41600	-6.14600
C	43.75900	-41.88300	-8.42400	49.57500	-71.88800	-5.83500
C	42.98500	-41.97100	-4.32400	50.96000	-67.71400	-6.63700
C	43.61400	-36.97000	-13.93700	53.73300	-77.07000	-3.40500
C	43.93200	-36.09200	-15.02600	53.94900	-78.43200	-3.09100
C	42.98400	-35.32700	-15.68800	55.28600	-78.92700	-2.98800
C	41.65800	-35.32100	-15.18900	56.40000	-78.00300	-3.14500
C	41.30300	-36.01300	-14.04400	56.10500	-76.61800	-3.45100
C	43.03400	-42.53100	0.96900	48.69400	-63.06000	-7.03700
C	46.69700	-36.18000	-9.51400	49.88500	-73.51300	-0.68300
C	47.82700	-35.36400	-9.69200	49.23500	-73.78500	0.51300
C	47.76600	-33.99600	-9.27200	49.70700	-74.69500	1.48700
C	46.79600	-33.62800	-8.32500	51.03200	-75.18900	1.35400

C	45.70300	-34.44300	-8.19400	51.74200	-74.84200	0.20300
C	40.86500	-39.30800	-4.07300	53.07000	-66.50500	-4.10100
C	40.82300	-39.19400	-2.67800	52.35100	-65.26800	-3.91200
C	41.07000	-37.99300	-2.02500	52.30700	-64.74100	-2.63200
C	41.25000	-36.76400	-2.72400	52.91600	-65.35500	-1.53100
C	41.42100	-36.90700	-4.16800	53.52900	-66.60500	-1.71800
C	37.10800	-35.11800	-10.25100	58.56700	-74.01400	-1.86300
C	35.99200	-34.91200	-11.04200	59.65300	-74.80500	-2.35300
C	36.14700	-34.37000	-12.35900	60.59300	-74.38200	-3.34000
C	37.43800	-34.37000	-12.93600	60.24200	-73.16500	-3.99800
C	38.55800	-34.76700	-12.18200	59.22800	-72.28600	-3.49100
C	46.95300	-44.18300	0.68800	45.54100	-64.64700	-7.62000
C	49.30200	-38.96500	-3.83900	45.18200	-69.39100	-2.25900
C	40.73600	-41.85100	-12.53400	51.99600	-72.38500	-8.29100
C	41.20500	-43.22100	-13.42400	51.72800	-73.08600	-9.81100
C	42.81900	-43.50600	-13.78600	52.94700	-74.09900	-10.52700
C	44.04000	-42.39200	-13.26900	54.43800	-74.31500	-9.75600
C	43.57600	-40.97800	-12.41900	54.67300	-73.64900	-8.19300
C	40.85100	-38.07200	-4.79600	53.67400	-67.18100	-3.02500
C	45.63900	-35.77500	-8.71300	51.22100	-74.00700	-0.77300
C	41.90000	-40.72100	-12.09600	53.46800	-72.64900	-7.45000
C	42.27000	-36.84300	-13.45300	54.77500	-76.19400	-3.73400
C	44.06000	-40.74400	-7.62900	49.80300	-71.30000	-4.50100
C	38.39800	-35.32800	-10.91000	58.35600	-72.78000	-2.45000
C	37.45500	-39.40500	-8.86300	57.11600	-69.61800	-5.58300
C	43.18200	-42.20500	2.35000	48.56000	-63.44200	-8.35700
C	48.10300	-44.46600	1.45500	45.74900	-65.15200	-8.87300
C	42.05300	-42.85100	-3.89400	50.76400	-68.92700	-7.34900
C	50.08400	-38.06700	-3.12500	45.80500	-70.46100	-1.68700
C	47.52100	-43.55300	-4.70600	46.03300	-69.78600	-7.18200
C	48.86000	-43.66800	-4.64300	44.64500	-69.73000	-7.05200
C	44.73300	-36.79800	0.44600	47.50200	-63.00800	-2.11500
C	45.01600	-36.05100	-0.69600	47.77300	-62.72100	-0.79200
C	49.38400	-39.49300	0.93500	44.11400	-64.05600	-3.15400
C	50.12200	-38.31800	0.62700	43.89500	-63.46100	-1.89900
C	44.75300	-34.93500	-5.53200	51.76700	-68.88500	0.70600
C	43.77700	-31.76400	-6.98100	54.11300	-71.71500	2.81300
C	41.54700	-32.37900	-4.68300	54.35400	-68.56100	2.89900
C	43.89400	-34.17800	-4.72700	52.10500	-70.07600	1.31100
C	46.70900	-37.49000	-5.62200	49.03700	-70.38100	-0.04200
C	46.95000	-35.46300	-3.52000	48.97600	-67.83800	1.03700
C	37.02400	-35.97300	-6.50400	-1.68800	-69.21700	-1.36100
C	37.36600	-34.62300	-6.65300	-1.27900	-69.79900	-0.13800
C	38.03400	-33.91700	-5.62600	-1.94400	-69.34400	1.06200
C	38.15100	-34.51700	-4.30100	-2.93700	-68.32100	1.03200

C	37.91400	-35.87900	-4.16600	-3.45200	-67.94900	-0.25900
C	31.60300	-37.77700	-2.48400	0.73200	-61.67100	-0.32000
C	31.33900	-36.67500	-1.59400	0.90900	-60.93900	0.92800
C	31.44700	-36.85900	-0.19800	-0.15400	-60.23200	1.46600
C	32.03300	-37.98800	0.39600	-1.33400	-60.04000	0.65400
C	32.46600	-39.00200	-0.49100	-1.49800	-60.74200	-0.58700
C	29.96400	-41.42700	-1.29200	1.70400	-60.47200	-3.48400
C	33.21500	-42.45000	-8.56800	-0.16000	-66.52600	-7.92300
C	32.52700	-43.35500	-9.40400	-0.28300	-67.03700	-9.20000
C	32.68300	-44.74800	-9.30200	-0.34000	-66.24700	-10.34400
C	33.67800	-45.15000	-8.39200	-0.30400	-64.79300	-10.15300
C	34.50900	-44.23000	-7.65000	-0.31000	-64.26800	-8.87200
C	28.09000	-39.32800	2.07500	2.55600	-56.02200	-0.06100
C	33.18000	-45.07100	-4.31000	-0.68800	-61.08000	-7.58700
C	32.01200	-45.85900	-4.28800	-0.80000	-60.46200	-8.80400
C	31.14800	-45.78900	-3.15200	-1.87100	-59.58600	-9.12100
C	31.54400	-45.13600	-1.95400	-2.79500	-59.32700	-8.09200
C	32.79200	-44.44000	-1.90600	-2.69400	-59.87300	-6.80600
C	37.73700	-38.08500	-0.16100	-3.97200	-63.82000	0.85900
C	37.62400	-37.63100	1.18700	-4.86200	-63.40200	1.92300
C	36.36800	-37.11800	1.52700	-5.86400	-62.45200	1.66200
C	35.42000	-36.80000	0.58200	-6.00400	-61.87400	0.37100
C	35.57600	-37.13000	-0.80600	-5.10600	-62.22200	-0.66200
C	40.14800	-42.09300	-6.48100	-5.36100	-67.12600	-7.08400
C	40.72900	-42.48700	-7.71100	-5.73600	-67.88800	-8.20300
C	40.07400	-43.38500	-8.60900	-5.11000	-67.67000	-9.49400
C	38.86200	-43.95300	-8.16400	-4.09800	-66.66200	-9.49800
C	38.17200	-43.46300	-7.01100	-3.72000	-65.98100	-8.32100
C	26.56400	-42.86200	4.74600	3.28300	-53.72600	-3.39200
C	30.93500	-47.29200	3.12100	-3.27200	-56.10600	-6.30200
C	33.77500	-36.18400	-5.71900	2.71700	-64.55500	-3.20300
C	33.03600	-35.06600	-6.19300	4.00800	-64.94900	-2.75000
C	31.73900	-35.19400	-6.76900	4.50100	-66.23600	-3.08600
C	31.16200	-36.45600	-6.73100	3.67000	-67.15900	-3.70000
C	31.83600	-37.62400	-6.29300	2.42400	-66.79800	-4.27100
C	36.73000	-37.86500	-1.15200	-4.12600	-63.18300	-0.42000
C	33.51000	-44.31500	-3.10600	-1.66500	-60.82300	-6.58800
C	33.10600	-37.40400	-5.63900	1.95300	-65.46300	-4.00600
C	34.28600	-42.85400	-7.80600	-0.15400	-65.12500	-7.74300
C	32.19000	-38.97300	-1.89100	-0.43200	-61.47900	-1.10400
C	38.85100	-42.62300	-6.14200	-4.36800	-66.18100	-7.08100
C	37.48300	-36.56800	-5.33600	-2.70200	-68.21500	-1.44100
C	28.46400	-38.11400	1.47900	2.19200	-56.60500	1.16000
C	26.59300	-41.64700	5.37100	3.45700	-53.61200	-2.06000
C	28.92200	-40.46100	-1.30900	2.92800	-60.29000	-2.86900

C	29.69200	-47.75700	2.72700	-3.71100	-54.82300	-6.08100
C	28.01500	-45.95300	0.20400	2.90000	-58.03300	-6.85000
C	28.56700	-46.37900	-0.95900	2.58600	-58.93800	-7.88400
C	32.70700	-40.47900	5.39100	-1.89800	-55.79700	0.12100
C	32.74800	-39.26200	4.74900	-1.18200	-56.49100	1.06000
C	29.91800	-45.40300	6.68800	-0.50000	-52.63600	-4.93900
C	29.05200	-45.06400	7.71100	-0.02000	-51.55200	-4.25800
C	36.91200	-41.84700	0.85000	-5.61800	-60.00900	-3.07900
C	40.15900	-44.49500	0.26600	-7.46600	-60.97100	-7.01000
C	40.12900	-41.59400	-0.49800	-8.68600	-61.43400	-4.64500
C	37.70100	-43.03700	1.03900	-5.93000	-59.76200	-4.38200
C	35.97600	-42.07500	3.68900	-5.24300	-56.95400	-2.71300
C	34.64300	-39.94200	1.77200	-4.30900	-58.83500	-0.45300
C	29.74700	-50.96000	5.44800	-0.13700	-58.36900	-17.92500
C	28.33800	-50.92600	5.75100	0.11400	-58.52800	-19.34200
C	27.81500	-49.99600	6.69400	-0.68800	-59.47300	-20.02900
C	28.74700	-49.05800	7.20400	-1.77500	-60.09100	-19.39300
C	30.11500	-48.93800	6.87300	-1.86200	-60.03900	-17.93800
C	34.60000	-46.71300	0.96200	-5.66000	-61.50900	-10.73500
C	35.06400	-46.39000	-0.34800	-5.31800	-61.74600	-9.38000
C	36.45400	-46.22700	-0.49100	-4.20600	-62.51000	-9.08000
C	37.33000	-46.47000	0.60300	-3.36100	-62.98200	-10.09900
C	36.81100	-46.48300	1.91600	-3.66800	-62.69500	-11.43400
C	32.90800	-48.99200	-0.83100	-7.14100	-64.12500	-16.20100
C	37.05000	-49.94300	9.67500	-0.45300	-56.99500	-9.91900
C	38.05200	-50.32600	10.62100	0.63700	-56.28100	-9.32400
C	39.42800	-49.99300	10.44600	0.52100	-54.88800	-8.97800
C	39.74600	-49.19800	9.26500	-0.70800	-54.27900	-9.26700
C	38.77400	-48.70000	8.39200	-1.76500	-54.86200	-9.94600
C	30.08000	-49.44400	-4.01400	-11.32700	-66.88300	-16.16100
C	39.88200	-51.31200	3.60600	-5.85600	-58.36700	-8.72300
C	40.78000	-51.90400	2.70700	-6.28500	-57.54500	-7.70900
C	41.00600	-51.23500	1.46300	-7.46200	-56.81800	-7.87800
C	40.15800	-50.13500	1.08500	-8.26100	-56.91100	-8.99000
C	39.40900	-49.51900	2.08900	-7.76900	-57.72000	-10.06200
C	30.83200	-50.66200	1.57900	-7.40300	-60.15100	-16.71000
C	30.26400	-51.01100	0.33800	-8.31800	-60.07800	-17.76600
C	30.94400	-51.77900	-0.64900	-7.94200	-59.55200	-19.05000
C	32.06700	-52.56700	-0.23300	-6.57100	-59.20800	-19.25500
C	32.56300	-52.43200	1.07800	-5.63700	-59.45500	-18.28100
C	33.41900	-52.60800	9.84600	-3.74900	-53.30700	-15.65100
C	33.43600	-53.38800	11.00300	-3.34000	-52.33800	-16.54100
C	34.64900	-53.64400	11.64400	-2.00400	-51.97200	-16.52500
C	35.80800	-53.62900	10.85200	-1.06300	-52.61700	-15.65900
C	35.75200	-53.24800	9.49400	-1.55400	-53.55100	-14.75200

C	32.72900	-48.21600	-8.25700	-9.80700	-69.79500	-12.50200
C	38.46400	-51.81400	-4.79100	-7.12400	-64.72100	-8.62700
C	33.33300	-45.84900	7.68400	-0.10100	-61.41000	-12.32300
C	33.42300	-44.57000	8.30000	0.91300	-61.76200	-11.39800
C	34.24900	-43.55300	7.66500	2.10500	-61.08300	-11.27000
C	34.84700	-43.76600	6.39800	2.18600	-59.86600	-11.87500
C	34.61600	-45.00000	5.75800	1.18100	-59.36800	-12.76800
C	31.97300	-51.47800	1.96900	-6.05500	-60.01000	-17.02200
C	39.18500	-50.15300	3.34500	-6.53500	-58.40900	-9.91500
C	33.80700	-46.03100	6.37700	0.10000	-60.23300	-13.04100
C	37.41400	-49.12400	8.58200	-1.70100	-56.28800	-10.25700
C	35.46200	-46.74000	2.11700	-4.78200	-61.95300	-11.80600
C	34.52300	-52.87300	8.95100	-2.91800	-53.91500	-14.72300
C	30.58100	-50.11000	6.21400	-1.13600	-59.05600	-17.22000
C	29.01900	-50.29400	-4.23900	-11.11200	-66.35300	-17.44800
C	32.06700	-49.11700	-9.11100	-9.07900	-70.24800	-11.43100
C	31.90400	-48.08300	-0.76200	-7.81600	-63.34200	-17.15800
C	39.54300	-51.31000	-4.11700	-7.86700	-64.36900	-7.53500
C	36.72500	-46.67900	-5.08600	-6.28800	-67.91100	-13.15600
C	36.57400	-45.65600	-4.12700	-5.00400	-68.18100	-12.71200
C	32.03500	-53.70600	-5.69000	-13.29800	-64.16800	-11.74000
C	30.96300	-54.32600	-5.04000	-14.03600	-63.31500	-12.57000
C	34.31800	-52.72800	-8.38500	-11.99200	-67.28300	-9.59900
C	33.24700	-52.16800	-9.00400	-12.38000	-68.43400	-10.28400
C	36.66900	-53.77900	1.07600	-9.53900	-58.49800	-12.72000
C	38.51900	-54.68000	5.00100	-10.32700	-55.79800	-14.54100
C	36.30900	-56.26100	3.26500	-8.13400	-56.42900	-16.55000
C	37.59300	-53.62200	2.09000	-9.32900	-58.52300	-14.07700
C	36.44600	-50.68300	0.23600	-10.17400	-60.22500	-10.16400
C	38.50100	-52.58100	-1.04800	-11.74900	-60.32800	-12.71300
C	-7.31100	-35.69500	0.44000	44.73300	-64.38200	1.28200
C	-7.11700	-34.38600	1.04800	45.54800	-63.59200	2.14600
C	-7.12300	-33.15400	0.26500	46.65800	-64.12300	2.82700
C	-7.55900	-33.26100	-1.06200	46.80100	-65.52400	2.69100
C	-7.76800	-34.54800	-1.66100	46.06200	-66.36900	1.83600
C	-7.30300	-42.32500	-5.86500	40.52800	-71.38100	-3.43800
C	-8.35600	-42.95900	-6.55700	40.94800	-71.73600	-4.73900
C	-9.25900	-42.21200	-7.36100	42.23500	-72.19600	-4.95500
C	-9.20300	-40.79100	-7.32100	43.22300	-72.20100	-3.91600
C	-8.27900	-40.10200	-6.52500	42.78200	-71.81000	-2.60500
C	0.60400	-45.81000	-7.08000	32.40800	-72.13100	-0.46000
C	-0.83500	-35.79700	-5.19000	41.28200	-71.79900	4.74800
C	-0.02200	-35.40200	-6.27200	41.79400	-72.62600	5.77900
C	-0.64100	-34.90300	-7.46700	42.13200	-72.06600	7.03000
C	-1.98500	-34.48200	-7.48500	42.04100	-70.65600	7.16100

C	-2.74600	-34.79300	-6.35400	41.50700	-69.82700	6.17300
C	-0.52200	-43.33600	-10.60000	34.85800	-76.25000	0.42300
C	-1.44100	-41.19800	-6.53300	37.96800	-71.98100	2.48500
C	-0.18800	-41.32500	-7.23100	36.79900	-72.65000	2.98700
C	0.91100	-40.48000	-7.10000	35.50400	-72.17000	2.60500
C	0.94000	-39.76300	-5.91400	35.38300	-71.09200	1.65800
C	-0.26300	-39.59300	-5.12300	36.53500	-70.38900	1.18800
C	-8.57900	-41.25300	-2.25500	42.28700	-67.83300	-4.14500
C	-9.46800	-42.22200	-1.76900	41.96600	-67.69300	-5.53500
C	-9.32800	-42.84500	-0.48700	41.35300	-66.48300	-5.96400
C	-8.25800	-42.33600	0.34700	40.97000	-65.42900	-5.08200
C	-7.52200	-41.22400	-0.01400	41.12800	-65.73500	-3.69900
C	-1.46600	-34.62600	-0.59500	41.02500	-66.05300	5.95700
C	-1.19500	-33.49700	0.18200	41.79000	-65.98900	7.17000
C	-2.12300	-32.44100	0.33500	43.03300	-65.33200	7.03400
C	-3.47100	-32.63200	-0.06800	43.41700	-64.56400	5.81600
C	-3.75600	-33.84300	-0.66200	42.50000	-64.56400	4.69600
C	-3.20300	-46.13300	-12.19000	35.37800	-77.95600	-4.07100
C	-6.81000	-47.49700	-5.58800	36.56500	-71.50500	-6.96700
C	-8.26400	-34.87900	-4.86200	43.78100	-72.45000	1.05900
C	-9.56500	-34.40000	-5.23700	44.88100	-73.28000	0.66600
C	-10.31800	-35.16500	-6.13500	46.20100	-72.98500	1.04900
C	-9.68800	-36.23400	-6.76700	46.46100	-71.92100	2.00100
C	-8.40700	-36.63100	-6.52300	45.46100	-70.93700	2.12300
C	-7.70800	-40.71200	-1.31300	41.87100	-66.80200	-3.27600
C	-1.35600	-40.37800	-5.33500	37.78700	-70.94400	1.49900
C	-7.61400	-35.90200	-5.65200	44.05700	-71.31300	1.84200
C	-2.17900	-35.44900	-5.19200	41.09200	-70.41000	4.97800
C	-7.25300	-40.92500	-6.06700	41.46400	-71.38200	-2.37600
C	-2.78400	-34.81200	-0.97700	41.37000	-65.43000	4.69400
C	-7.54800	-35.70300	-0.93300	45.04000	-65.75700	1.09200
C	-0.99500	-42.74700	-11.74300	34.87400	-75.75700	1.74100
C	-2.27800	-47.06700	-12.58300	34.33900	-78.85600	-4.30100
C	1.11900	-45.67100	-8.36300	31.30200	-71.73600	-1.22900
C	-7.09200	-48.86400	-5.63700	37.86200	-71.89600	-7.14500
C	-2.17800	-49.48200	-8.96100	31.59200	-73.29900	-5.09200
C	-0.92500	-49.18300	-9.51000	31.61300	-73.82400	-6.39000
C	-4.71200	-41.13200	-8.50800	39.39800	-74.46000	-0.99900
C	-5.65900	-40.94900	-9.52600	40.03600	-75.42700	-1.73400
C	-7.90100	-44.62000	-9.50100	39.01400	-74.72200	-5.83500
C	-8.85600	-45.58500	-9.67400	39.95500	-75.67300	-5.47400
C	-4.44200	-43.11600	-1.65600	37.11700	-67.35200	-1.75600
C	-1.11200	-42.57400	0.90500	35.81200	-64.40600	-0.34000
C	-4.16400	-41.69500	1.43600	38.83500	-63.50700	-0.72600
C	-3.32800	-43.37300	-0.84900	37.58100	-66.06900	-2.03100

C	-4.28700	-46.21600	-2.65100	35.92200	-67.92400	-4.48200
C	-6.66500	-44.09700	-3.16300	38.76700	-68.59000	-4.19400
H	36.50400	-37.58200	-8.38000	56.38600	-70.20100	-7.63000
H	34.78000	-37.82800	-10.21200	58.63400	-70.62600	-8.41300
H	34.42700	-40.00700	-11.16200	60.67700	-69.77500	-7.08700
H	36.28100	-41.70300	-11.22400	60.37800	-69.81000	-4.74200
H	37.97600	-41.45700	-9.28600	58.08700	-69.02000	-3.77900
H	45.62200	-39.52100	-6.95200	49.13800	-71.62700	-2.57000
H	47.34600	-41.27100	-6.89800	46.84000	-72.19400	-3.05600
H	46.76300	-43.39900	-8.06300	46.28400	-72.87700	-5.40800
H	44.57100	-43.87500	-8.95900	48.06300	-72.76300	-7.12100
H	42.68800	-42.10000	-8.62800	50.16300	-71.83500	-6.72700
H	43.26000	-42.07700	-5.32100	51.83100	-67.42900	-6.07700
H	42.44100	-41.08000	-4.51400	51.01300	-66.92600	-7.37200
H	44.26400	-37.78400	-13.61200	52.84200	-76.78300	-3.93000
H	44.77800	-36.25600	-15.62800	53.16900	-79.02000	-2.66000
H	43.11700	-34.72200	-16.50400	55.49800	-79.96600	-2.88500
H	40.89800	-34.76700	-15.73200	57.35800	-78.40000	-2.99600
H	40.37100	-35.88500	-13.50800	56.92300	-75.93300	-3.41400
H	42.13600	-42.30500	0.47700	47.83100	-62.50500	-6.68100
H	43.19200	-43.40900	0.51700	49.49900	-62.36100	-6.82600
H	46.63800	-37.08200	-10.03200	49.28000	-73.05100	-1.52700
H	48.63400	-35.74900	-10.30700	48.60500	-73.01400	0.73700
H	48.72700	-33.47800	-9.30200	49.22900	-74.85500	2.48000
H	46.94500	-32.75800	-7.71800	51.49700	-75.87500	2.05100
H	44.94200	-34.19000	-7.44000	52.47000	-75.45900	-0.24400
H	40.69100	-40.24100	-4.55800	53.30100	-66.72100	-5.15000
H	40.70900	-40.05300	-2.09900	51.57800	-65.08700	-4.62900
H	41.05500	-37.82500	-0.98200	52.03400	-63.64400	-2.45800
H	41.63500	-35.83600	-2.33300	52.93000	-64.89900	-0.53700
H	41.66300	-35.99900	-4.61100	53.76100	-67.20600	-0.86700
H	37.06300	-35.34800	-9.26800	57.91500	-74.35200	-1.07400
H	34.99600	-35.07000	-10.68200	60.14600	-75.33000	-1.57100
H	35.31200	-34.35400	-12.99000	61.16800	-75.12400	-3.82400
H	37.53900	-34.10400	-13.95700	60.95300	-72.75900	-4.68200
H	39.45600	-34.98400	-12.64800	59.20900	-71.33700	-3.85600
H	46.52000	-44.88100	0.04100	46.06800	-63.79700	-7.32900
H	46.13400	-43.99300	1.38600	44.58700	-64.20100	-7.49200
H	49.12400	-38.49600	-4.81300	44.41400	-69.35500	-2.99900
H	49.88700	-39.76800	-4.21700	44.60400	-68.74100	-1.60500
H	39.73300	-41.83900	-12.08700	51.26100	-71.73100	-7.89200
H	40.60700	-44.10900	-13.47100	50.72900	-73.04900	-10.24900
H	43.07900	-43.97900	-14.69200	52.85000	-74.20800	-11.55800
H	45.06600	-42.60000	-13.25600	55.19200	-74.57900	-10.34000
H	44.26700	-40.29300	-12.02100	55.68700	-73.67000	-7.92000

H	42.81900	-41.24900	2.46200	49.41600	-63.78400	-8.89000
H	44.02300	-42.42600	2.94900	48.27600	-62.65500	-9.02600
H	42.65100	-42.66800	3.09800	47.77300	-64.21500	-8.43100
H	48.56200	-43.78700	2.15000	46.76500	-65.44700	-9.15600
H	48.26300	-45.34700	2.03500	45.26400	-66.11500	-9.03500
H	49.04500	-44.79600	0.95700	45.66300	-64.55900	-9.80000
H	41.13300	-42.86800	-4.48900	49.79200	-69.17200	-7.81800
H	41.65400	-42.58200	-2.90400	50.75700	-69.86300	-6.77100
H	42.31700	-43.82800	-3.72600	51.42700	-69.20200	-8.16900
H	50.38600	-38.43900	-2.14000	45.25600	-71.12500	-1.07700
H	50.86500	-37.66600	-3.80500	46.34300	-71.25300	-2.19500
H	49.72500	-37.17400	-2.80100	46.10900	-70.36400	-0.64900
H	47.00000	-44.50700	-4.69600	46.20200	-69.38800	-8.18700
H	47.03600	-43.39800	-5.59200	46.50300	-70.77700	-7.21100
H	49.52200	-42.78100	-4.74800	44.10700	-70.24900	-7.83800
H	49.36300	-44.47800	-5.08300	44.28700	-68.70900	-7.09200
H	49.15900	-43.89900	-3.66700	44.07800	-70.05600	-6.21000
H	43.72200	-36.65200	0.80000	47.97700	-62.23500	-2.71300
H	45.39500	-36.70400	1.25200	46.59900	-62.90100	-2.68800
H	44.45600	-36.31000	-1.61300	47.42500	-61.73300	-0.41200
H	46.00200	-35.98800	-1.06200	48.76800	-62.59600	-0.41900
H	45.06700	-35.00800	-0.57200	47.20400	-63.29100	-0.12700
H	49.99300	-40.36700	0.90700	43.23400	-64.28100	-3.76500
H	49.13100	-39.37700	1.96500	44.56000	-63.36600	-3.84300
H	50.87000	-37.94200	1.29200	43.28100	-62.57100	-1.93300
H	49.64000	-37.35800	0.48100	44.80200	-63.44000	-1.32600
H	50.72900	-38.37800	-0.27200	43.25800	-64.15900	-1.33000
H	45.56800	-34.37900	-6.01400	52.26900	-68.50600	-0.15600
H	44.23000	-35.38400	-6.39700	51.81800	-68.06800	1.28800
H	44.57600	-31.31700	-6.50600	53.36800	-71.86400	3.59800
H	44.46700	-32.24300	-7.66100	53.64600	-72.52600	2.35400
H	43.06700	-31.33100	-7.57700	55.11000	-71.78900	3.23000
H	41.83000	-31.55700	-4.10000	53.99200	-68.47200	3.84800
H	40.98100	-33.04600	-4.15200	54.38300	-67.63600	2.33800
H	40.93900	-31.66100	-5.26300	55.39000	-68.82600	2.96300
H	44.38400	-33.70900	-3.88700	51.96400	-70.93100	0.73200
H	43.26100	-34.75400	-4.07500	51.36600	-70.25900	2.08100
H	47.48300	-36.91100	-6.07600	49.01100	-70.75500	0.97000
H	46.34500	-37.70100	-6.58600	49.41900	-71.00700	-0.86300
H	47.29700	-38.18200	-5.05600	48.13900	-70.08600	-0.49400
H	46.40700	-34.90100	-2.80200	49.12900	-66.78900	1.12400
H	47.58400	-36.18400	-3.06700	47.98400	-68.03400	0.60500
H	47.69900	-35.07800	-4.13200	48.84000	-68.29800	2.02900
H	36.49700	-36.47700	-7.28300	-1.10800	-69.60700	-2.17100
H	36.92300	-34.07000	-7.46400	-0.43900	-70.41200	-0.05800

H	38.54700	-32.97500	-5.88000	-1.59000	-69.81400	1.99800
H	38.14500	-33.78600	-3.53400	-3.61200	-68.28700	1.88600
H	38.31600	-36.38600	-3.28000	-4.12000	-67.16100	-0.39100
H	31.78300	-37.56800	-3.48300	1.55900	-61.92000	-0.94600
H	30.65700	-35.97600	-2.06100	1.97100	-60.99000	1.28800
H	31.21300	-36.01900	0.44500	-0.01100	-59.70400	2.39000
H	32.01600	-38.08900	1.48900	-2.18400	-59.48000	0.95900
H	33.09400	-39.75100	-0.07500	-2.50500	-60.80000	-0.96000
H	30.88800	-40.98700	-1.64500	0.96200	-61.19200	-3.06800
H	29.91200	-42.23300	-1.99000	1.52400	-60.73600	-4.48400
H	32.94900	-41.43000	-8.56900	0.12200	-67.24100	-7.20200
H	32.37700	-42.91200	-10.35400	-0.51700	-68.07000	-9.42700
H	32.23900	-45.57500	-9.86000	-0.81600	-66.61400	-11.25200
H	33.85400	-46.19500	-8.34300	-0.05900	-64.16800	-10.99600
H	35.25600	-44.60400	-6.97100	-0.11700	-63.22800	-8.70500
H	27.24100	-39.27900	2.72200	2.62700	-54.99200	-0.31800
H	27.46800	-40.00300	1.52400	3.56600	-56.02200	-0.33000
H	33.62800	-44.92800	-5.24700	-0.01900	-61.92600	-7.39300
H	31.66500	-46.53900	-5.05500	-0.28000	-60.73900	-9.66500
H	30.17300	-46.13000	-3.34600	-1.81600	-59.17400	-10.10000
H	31.12700	-45.28700	-0.98200	-3.25600	-58.32800	-8.08600
H	33.02200	-43.87500	-1.02400	-3.15600	-59.21900	-6.12300
H	38.67900	-38.52300	-0.51800	-3.16600	-64.52400	0.84300
H	38.46100	-37.58100	1.91800	-4.46500	-63.49500	2.95600
H	36.22500	-36.57500	2.44100	-6.57600	-62.15800	2.45700
H	34.60400	-36.22300	0.97200	-6.86000	-61.21400	0.20300
H	34.66600	-37.00300	-1.44300	-4.97000	-61.50100	-1.42800
H	40.67300	-41.49800	-5.84900	-5.72400	-67.41700	-6.13200
H	41.80700	-42.30700	-7.95100	-6.40200	-68.68800	-8.03900
H	40.44300	-43.42600	-9.57300	-5.34300	-68.33900	-10.33000
H	38.37000	-44.62300	-8.78800	-3.56900	-66.50500	-10.43700
H	37.40800	-44.01500	-6.57900	-2.93100	-65.37800	-8.55100
H	26.37200	-43.75800	5.33100	2.99200	-52.88000	-3.97000
H	25.61300	-43.08800	4.25000	4.20600	-53.75900	-3.80500
H	31.08800	-47.36400	4.12700	-3.81600	-56.99200	-6.01800
H	31.83200	-47.79000	2.79700	-3.18600	-56.42200	-7.37100
H	34.59800	-35.94200	-5.03900	2.19000	-63.66700	-2.86000
H	33.45500	-34.05800	-6.31900	4.55500	-64.21700	-2.12600
H	31.39900	-34.36300	-7.37800	5.51600	-66.54000	-2.86600
H	30.20300	-36.54500	-7.23400	4.09200	-68.11300	-3.92700
H	31.27900	-38.54900	-6.18800	1.81400	-67.62900	-4.62000
H	27.75500	-37.40300	1.13600	2.70900	-56.13800	2.02500
H	29.16700	-38.12400	0.66600	2.44200	-57.67300	1.36800
H	28.85700	-37.45100	2.10700	1.24100	-56.49400	1.75200
H	27.43200	-41.60600	6.05400	3.81600	-54.50400	-1.52200

H	25.87700	-41.40700	6.13500	2.70300	-53.18700	-1.39000
H	26.74700	-40.64500	4.92900	4.22800	-53.00900	-1.71200
H	28.78800	-39.80600	-2.11300	3.66000	-60.99500	-3.07800
H	27.84700	-40.73500	-1.26200	3.62000	-59.58900	-3.21100
H	29.07100	-39.63200	-0.63300	2.99300	-60.22200	-1.84600
H	29.40200	-47.57700	1.71900	-4.79100	-54.67500	-6.29600
H	29.52000	-48.79700	3.03100	-3.92200	-54.38000	-5.05300
H	28.86500	-47.17700	3.06200	-3.06200	-54.12300	-6.71900
H	27.03300	-45.46200	0.07800	3.51200	-58.20400	-5.95600
H	27.69400	-46.72700	0.91800	3.66900	-57.31300	-7.04100
H	28.91500	-45.54400	-1.53300	1.78100	-59.69700	-7.64500
H	28.01800	-47.09400	-1.64400	3.23400	-59.73000	-8.28700
H	29.57400	-46.88200	-0.92000	2.31200	-58.47800	-8.80500
H	32.21700	-40.32200	6.30000	-2.09400	-54.71700	0.35700
H	33.62800	-40.88500	5.67800	-2.94000	-55.99000	-0.01800
H	32.66200	-38.31500	5.15300	-0.73700	-57.48800	0.92700
H	32.99000	-39.18400	3.67000	-0.40800	-56.05700	1.70700
H	31.79200	-38.98100	4.45500	-1.57600	-56.83300	2.00500
H	29.59400	-46.26900	6.18500	0.16400	-52.81900	-5.78400
H	30.89500	-45.76500	6.92100	-1.35700	-52.61400	-5.56300
H	28.82100	-45.76000	8.48300	0.42000	-50.81200	-4.96000
H	29.18700	-44.08600	8.09300	-0.55700	-50.98100	-3.51900
H	28.00400	-45.20100	7.51500	0.95900	-51.66900	-3.81000
H	36.63500	-41.72100	-0.17900	-5.11200	-60.93400	-2.77600
H	37.39400	-40.94800	1.07000	-6.48200	-60.26900	-2.52500
H	39.84000	-45.47500	0.25100	-6.63000	-61.13700	-7.65300
H	40.92200	-44.53500	-0.44500	-8.04300	-61.78300	-7.25900
H	40.66600	-44.14800	1.16900	-8.10800	-60.18200	-7.16300
H	39.67300	-40.72000	-0.81800	-9.26100	-62.17000	-5.18200
H	40.78900	-41.24400	0.33700	-8.73800	-61.44000	-3.62900
H	40.73500	-41.72900	-1.34700	-9.35100	-60.62000	-4.84600
H	38.20200	-43.04700	2.00800	-6.44400	-58.89900	-4.57400
H	37.14800	-43.97500	1.02800	-5.03500	-59.58900	-5.04700
H	36.45000	-43.02100	3.67300	-5.41300	-56.82700	-3.74400
H	35.16500	-42.21200	4.38300	-4.70000	-56.17800	-2.22600
H	36.63900	-41.22200	3.97800	-6.18700	-56.88900	-2.12500
H	34.49600	-39.91600	0.74100	-3.99300	-59.76700	-0.10400
H	33.82800	-39.95500	2.45800	-3.59400	-58.13000	-0.05300
H	35.26000	-39.10300	1.94200	-5.29500	-58.67800	0.03500
H	30.07900	-51.62900	4.68500	0.56800	-57.80600	-17.27000
H	27.73600	-51.19600	5.00000	0.87000	-57.93900	-19.71400
H	26.76900	-49.99800	6.99600	-0.64200	-59.51300	-21.08100
H	28.34700	-48.17800	7.71900	-2.37600	-60.77000	-19.95300
H	30.75300	-48.34500	7.37200	-2.42900	-60.75000	-17.39000
H	33.59400	-46.98900	1.14200	-6.69300	-61.52600	-10.94200

H	34.52200	-46.04200	-1.17800	-5.91000	-61.39500	-8.53400
H	36.85300	-46.06700	-1.47800	-3.74000	-62.29300	-8.19300
H	38.33900	-46.70400	0.34500	-2.42200	-63.42000	-9.80900
H	37.62000	-46.47700	2.64500	-2.81100	-62.69800	-12.07500
H	32.60000	-50.02600	-0.60000	-6.15900	-63.75400	-16.12400
H	33.65400	-49.02200	-0.02700	-6.72700	-65.02500	-16.57000
H	36.11000	-50.31200	9.81900	-0.43000	-58.02100	-10.09500
H	37.69700	-50.80200	11.55700	1.35300	-56.79500	-8.69700
H	40.06800	-49.82800	11.25500	1.42600	-54.34500	-8.71100
H	40.79100	-49.05500	9.00100	-0.75600	-53.21900	-8.95800
H	38.96200	-48.08800	7.54500	-2.31800	-54.20300	-10.57500
H	29.89200	-48.43600	-4.22300	-12.31600	-67.19000	-15.85900
H	30.19000	-49.30800	-2.98500	-10.91500	-67.87600	-16.10900
H	39.59000	-51.91500	4.43900	-4.91000	-58.81400	-8.69800
H	41.28600	-52.72300	3.04600	-5.60600	-57.15100	-6.98500
H	42.01400	-51.14600	1.11500	-7.86300	-56.20800	-7.14800
H	40.40800	-49.65800	0.15000	-9.35800	-56.89600	-9.02800
H	38.59500	-48.86000	1.84800	-8.21200	-57.71900	-11.04500
H	30.29900	-50.12600	2.37400	-7.69100	-60.68800	-15.76300
H	29.52900	-50.27800	-0.11600	-9.25300	-60.58300	-17.67400
H	30.46900	-51.78000	-1.56600	-8.72600	-59.30600	-19.82000
H	32.47000	-53.37100	-0.77500	-6.26800	-59.08900	-20.32400
H	33.56200	-52.71400	1.49600	-4.68800	-58.99200	-18.35000
H	32.52100	-52.22100	9.44800	-4.78700	-53.57100	-15.80000
H	32.68600	-53.24100	11.69300	-4.08800	-51.60400	-16.87500
H	34.50200	-54.28100	12.55700	-1.70100	-51.26700	-17.28800
H	36.81000	-53.94800	11.24400	0.01200	-52.59100	-15.90200
H	36.55200	-53.83900	8.95500	-0.89400	-53.91000	-13.97200
H	32.32700	-47.41700	-7.68400	-10.84400	-70.08900	-12.39300
H	33.41600	-47.46000	-8.72200	-9.79700	-70.45700	-13.34300
H	38.58100	-51.91300	-5.89200	-6.09300	-64.36200	-8.75400
H	38.25800	-52.86100	-4.53300	-6.79300	-65.72700	-8.61700
H	32.76800	-46.61800	8.08300	-0.94800	-62.02000	-12.50900
H	32.88800	-44.33200	9.22300	0.93400	-62.70100	-10.90900
H	34.52000	-42.58500	8.13300	2.96000	-61.46900	-10.69200
H	34.95500	-42.97100	5.74100	2.96000	-59.21900	-11.49900
H	35.12100	-45.25500	4.76400	1.49500	-58.55700	-13.35200
H	28.83400	-50.35500	-5.27700	-10.15300	-66.23700	-17.95800
H	29.13400	-51.29300	-3.86700	-11.68400	-66.60900	-18.30000
H	28.04500	-50.09400	-3.79600	-11.37400	-65.34500	-17.74500
H	31.39900	-49.76300	-8.54700	-9.07700	-69.64800	-10.57500
H	31.78300	-48.74600	-10.13300	-9.43900	-71.12300	-10.89500
H	32.75500	-49.76200	-9.71200	-7.98200	-70.33600	-11.51300
H	32.16300	-47.04300	-0.65200	-8.24700	-62.43000	-16.75000
H	31.16500	-48.01700	-1.53300	-7.49300	-62.94300	-18.10300

H	31.35700	-48.09900	0.21700	-8.82900	-63.58600	-17.52200
H	40.40500	-51.93100	-4.21100	-8.21400	-63.40600	-7.62100
H	39.81000	-50.36000	-4.42900	-8.76800	-64.86500	-7.22200
H	39.44200	-51.23900	-3.02900	-7.47000	-64.46600	-6.63200
H	37.68200	-47.25200	-5.01800	-6.98500	-68.66300	-12.90800
H	36.98300	-46.42200	-6.11400	-6.44900	-68.18800	-14.19400
H	36.73200	-45.88300	-3.11800	-4.69600	-68.21700	-11.64400
H	35.88200	-44.79200	-4.10600	-4.29400	-67.52900	-13.09900
H	37.27200	-44.81300	-4.30600	-4.63800	-69.02300	-13.26200
H	32.73300	-54.13700	-6.38600	-13.27800	-63.88700	-10.70100
H	31.70100	-52.99400	-6.46200	-13.83700	-65.06200	-11.51700
H	30.28100	-53.70600	-4.50100	-13.57100	-62.38500	-12.94800
H	31.22300	-54.90000	-4.20100	-15.01600	-62.90600	-12.33300
H	30.13700	-54.81500	-5.58600	-14.23700	-63.66900	-13.51800
H	35.20900	-52.79400	-8.95300	-11.77800	-67.51700	-8.56700
H	34.22900	-53.73300	-8.09500	-12.69200	-66.55400	-9.29800
H	33.19300	-51.12600	-9.10100	-11.64800	-69.18900	-10.21400
H	32.22200	-52.19800	-8.63800	-12.33400	-68.31900	-11.36900
H	32.95200	-52.45700	-10.01300	-13.27200	-68.93200	-10.07200
H	35.64700	-53.74400	1.43900	-10.28000	-57.73800	-12.44900
H	36.46900	-54.79900	0.70400	-8.70800	-58.26900	-12.17300
H	38.83200	-53.65000	5.15100	-11.07400	-56.27100	-15.13900
H	39.24600	-55.26500	4.45600	-10.33300	-54.72800	-14.78700
H	38.33100	-55.22800	5.91000	-10.65200	-55.63700	-13.52600
H	37.05700	-56.64900	2.53800	-8.88100	-56.59000	-17.26600
H	35.57000	-55.98300	2.53700	-7.33800	-57.10000	-16.63700
H	35.87800	-56.78400	4.10300	-7.66200	-55.42500	-16.54900
H	37.86800	-52.59900	2.33800	-10.19900	-58.94100	-14.57600
H	38.55400	-54.02500	1.85800	-8.59300	-59.22200	-14.43600
H	35.46300	-50.42500	0.74300	-9.38500	-59.87600	-9.63500
H	36.86200	-50.03800	-0.54200	-10.33400	-61.17900	-9.69900
H	37.20500	-50.50500	0.99700	-11.09500	-59.64400	-9.90000
H	38.64100	-53.57800	-1.35700	-11.62400	-60.16000	-13.80800
H	39.28100	-52.52400	-0.31400	-12.49900	-59.62400	-12.47600
H	38.63800	-51.91600	-1.83500	-12.35100	-61.15800	-12.43900
H	-7.21700	-36.62800	0.97300	43.96600	-63.86200	0.74500
H	-6.38700	-34.44500	1.79900	45.59400	-62.54100	2.00400
H	-7.01200	-32.19300	0.69600	47.28000	-63.62000	3.62600
H	-7.88600	-32.40500	-1.59800	47.48100	-66.05000	3.34000
H	-8.08300	-34.75300	-2.64800	45.96400	-67.40900	1.95100
H	-6.83900	-42.83800	-5.02300	39.46200	-71.47300	-3.23500
H	-8.66100	-43.91800	-6.12300	40.28100	-71.50900	-5.57300
H	-10.08600	-42.78100	-7.74900	42.48300	-72.58700	-5.96200
H	-9.97900	-40.14900	-7.63900	44.27800	-72.13800	-4.24300
H	-8.10900	-39.04400	-6.73900	43.18100	-72.42900	-1.82000

H	0.80700	-46.69000	-6.48000	32.76800	-71.41400	0.25000
H	1.02800	-45.04900	-6.46100	32.20600	-72.89400	0.21000
H	-0.40300	-36.36800	-4.41400	40.63800	-72.36700	4.12600
H	1.04600	-35.68100	-6.28700	41.67900	-73.66500	5.70400
H	-0.00700	-35.00800	-8.32700	42.40000	-72.69700	7.81900
H	-2.48100	-34.15500	-8.36300	42.54500	-70.16900	7.96100
H	-3.63200	-34.17300	-6.25900	41.74000	-68.78400	6.27900
H	0.18800	-44.04300	-10.72800	35.10200	-77.33000	0.21200
H	0.09800	-42.75300	-10.01800	33.82400	-76.49500	0.16200
H	-2.39000	-41.45600	-6.97000	39.00800	-72.35600	2.71200
H	-0.13400	-41.74800	-8.20600	36.91800	-73.66600	3.30400
H	1.87300	-40.94600	-7.37100	34.69400	-72.30200	3.25900
H	1.67900	-39.00500	-5.74300	34.35000	-70.72100	1.55400
H	-0.29500	-38.76600	-4.48200	36.36400	-69.41800	0.77500
H	-8.46800	-41.16600	-3.34700	43.00600	-68.60100	-3.87000
H	-10.35100	-42.28800	-2.36900	42.01500	-68.52500	-6.21300
H	-9.80700	-43.71000	-0.10400	41.44400	-66.27900	-7.03000
H	-7.98600	-42.66000	1.28600	40.17000	-64.71400	-5.33900
H	-6.65700	-41.05800	0.49100	40.97500	-64.98100	-2.91600
H	-0.82100	-35.40500	-0.35000	40.24200	-66.77100	5.91100
H	-0.19300	-33.33300	0.55900	41.45100	-66.37600	8.12200
H	-1.98100	-31.94900	1.28000	43.78300	-65.20500	7.77200
H	-4.31800	-31.93400	-0.05500	44.35700	-64.00700	5.69600
H	-4.83400	-34.03200	-0.82100	42.70500	-63.93600	3.86600
H	-4.18200	-46.28600	-12.55500	36.15900	-78.08600	-4.84800
H	-3.02600	-45.11800	-12.54900	36.17100	-78.28500	-3.43100
H	-7.55300	-46.71600	-5.59600	36.29800	-70.46900	-6.77000
H	-6.35400	-47.21100	-4.66800	36.06500	-71.84900	-7.86900
H	-7.58900	-34.13900	-4.47200	42.80500	-72.87000	0.85300
H	-10.12300	-33.50500	-4.83000	44.55400	-74.02200	-0.10000
H	-11.36900	-35.00700	-6.37100	46.81800	-73.79500	1.05800
H	-10.23600	-36.94200	-7.26900	47.42800	-71.82200	2.45000
H	-7.85400	-37.25100	-7.19700	45.74300	-69.84800	2.33200
H	-1.43200	-41.82700	-11.64800	34.14300	-75.05300	2.04100
H	-1.59800	-43.32300	-12.37800	35.77800	-75.64600	2.31500
H	-0.17100	-42.44000	-12.39000	34.40800	-76.38400	2.47400
H	-2.53700	-48.08400	-12.45000	33.58300	-78.53600	-4.94000
H	-2.18500	-47.11000	-13.68900	34.58100	-79.82900	-4.76800
H	-1.24100	-47.10000	-12.33400	33.44000	-78.91000	-3.64600
H	0.84700	-46.52100	-9.03200	30.96500	-72.36600	-1.97200
H	1.02300	-44.77800	-8.86000	30.37200	-71.70400	-0.63600
H	2.14800	-45.79800	-8.40400	31.31000	-70.85600	-1.75900
H	-7.63600	-49.20700	-6.45300	38.67500	-72.09500	-6.44300
H	-7.86400	-49.39100	-5.10300	38.36900	-71.60400	-8.02900
H	-6.27500	-49.53100	-5.42400	38.08300	-72.91100	-7.38100

H	-2.02000	-50.17700	-8.24300	30.92300	-73.81500	-4.41000
H	-2.97400	-49.79600	-9.55600	31.23400	-72.33200	-4.84300
H	-0.46100	-49.95100	-10.11600	30.56900	-73.79500	-6.84200
H	-0.82400	-48.35300	-10.18800	32.29900	-73.47300	-7.13900
H	-0.12100	-49.00100	-8.85400	31.81800	-74.86700	-6.58200
H	-5.01300	-40.78600	-7.55200	39.99200	-73.58200	-0.74700
H	-3.69400	-40.80700	-8.42000	39.12700	-74.80200	-0.05400
H	-5.26700	-41.15300	-10.54900	39.38200	-76.25200	-1.84700
H	-6.52700	-41.51800	-9.51200	40.18200	-75.37000	-2.79000
H	-5.91500	-39.88900	-9.63800	40.97300	-75.78000	-1.31000
H	-7.97100	-44.17000	-8.51400	38.75800	-74.63900	-6.86100
H	-8.04700	-43.74400	-10.13400	39.43200	-73.73600	-5.79300
H	-8.65900	-46.52200	-9.12400	40.19900	-76.01400	-4.46600
H	-9.16300	-45.82900	-10.63800	40.89700	-75.34200	-5.93900
H	-9.90600	-45.27900	-9.71100	39.87000	-76.69400	-5.83200
H	-4.58700	-42.20800	-2.15800	36.22800	-67.47600	-1.19500
H	-5.16800	-42.65000	-1.02600	37.57300	-67.81300	-0.95200
H	-1.05200	-43.50000	1.47100	35.36200	-63.84200	-1.16300
H	-0.39200	-42.85800	0.15900	35.11900	-65.26200	-0.37100
H	-0.60900	-41.78000	1.45100	35.76400	-63.84700	0.58500
H	-3.91700	-40.72800	1.75000	39.29000	-63.00700	0.16400
H	-5.06100	-41.60900	0.77200	39.81900	-63.70600	-1.12900
H	-4.38300	-42.56300	2.01600	38.50500	-62.82400	-1.50200
H	-3.47300	-44.17700	-0.17500	38.48100	-65.85700	-2.60200
H	-2.38700	-43.70500	-1.22000	37.01100	-65.45900	-2.67600
H	-4.44000	-47.02600	-3.29000	35.80400	-68.60600	-5.22800
H	-4.59800	-46.43300	-1.59800	36.28900	-67.07600	-5.09800
H	-3.26000	-46.22000	-2.53400	34.93400	-67.77400	-4.10100
H	-6.84000	-43.09100	-3.38500	39.61600	-68.78000	-3.69900
H	-7.24400	-44.46400	-2.30900	39.01900	-67.71500	-4.73500
H	-7.16500	-44.82400	-3.74500	38.81100	-69.39000	-4.90200

Table S4. Molecule interatomic distances of each of Ph_7T_8-T_8Et_7 structure from eight obtained clusters.

	Molecule interatomic distance along the axis [Å]											
	1st molecule			2nd molecule			3rd molecule			4th molecule		
	x	y	z	x	y	z	x	y	z	x	y	z
Cluster **1**	23.34	12.56	10.19	20.80	15.63	13.84	20.33	14.37	15.18	21.22	12.44	14.52
Cluster **2**	22.72	14.23	12.86	19.91	14.88	15.20	19.79	15.83	15.19	20.46	12.55	14.94
Cluster **3**	20.92	12.97	15.18	22.37	11.82	13.41	21.04	14.38	13.13	20.62	13.17	15.80
Cluster **4**	21.59	11.86	14.91	20.33	11.00	15.13	20.69	13.23	13.98	20.95	14.12	12.22
Cluster **5**	21.82	13.01	15.76	23.56	10.85	13.80	21.43	12.60	15.70	21.63	13.77	12.50
Cluster **6**	20.70	15.59	12.27	20.64	11.71	14.37	21.64	15.56	14.49	23.22	13.72	12.27
Cluster **7**	21.45	14.90	14.84	22.72	12.95	15.27	21.16	13.32	14.63	21.85	14.49	11.60
Cluster **8**	21.88	15.36	13.14	21.72	13.07	13.55	19.22	15.75	13.70	21.50	11.18	14.61

Table S5. Atomic coordinates of systems consisted of four molecules of Ph_7T_8-T_8iBu_7 compound for clusters 1-3.

Atomic coordinates of systems consisted of four molecules of Ph_7T_8-T_8iBu_7 compound									
	Cluster **1**			Cluster **2**			Cluster **3**		
Atom	x	y	z	x	y	z	x	y	z
Si	26.932000	5.429000	33.461000	32.588000	35.383000	33.182000	7.061000	35.469000	30.156000
Si	26.823000	1.272000	33.624000	28.608000	35.353000	33.880000	5.766000	37.349000	33.087000
Si	28.425000	-1.166000	32.884000	27.746000	36.561000	36.404000	3.575000	35.189000	32.794000
Si	28.029000	-3.958000	32.096000	26.104000	38.985000	37.368000	1.176000	34.835000	34.540000
Si	30.644000	-4.151000	30.737000	25.419000	37.476000	39.962000	-0.348000	33.977000	32.168000
Si	32.184000	-4.803000	33.196000	27.935000	38.715000	41.141000	0.210000	31.045000	32.494000
Si	29.667000	-4.682000	34.564000	28.439000	40.129000	38.636000	1.910000	31.841000	34.945000
Si	31.043000	-1.304000	31.409000	26.980000	35.060000	38.964000	1.983000	34.321000	30.325000
Si	32.673000	-1.929000	33.779000	29.470000	36.255000	40.221000	2.647000	31.494000	30.702000
Si	30.087000	-1.755000	35.215000	30.128000	37.876000	37.669000	4.241000	32.371000	33.093000
Si	55.221000	45.598000	41.150000	16.264000	22.118000	37.627000	35.332000	36.017000	44.623000
Si	56.363000	47.710000	44.608000	18.627000	25.381000	39.153000	30.828000	35.223000	43.450000
Si	56.192000	48.916000	47.241000	18.903000	28.386000	39.346000	30.325000	36.785000	40.863000
Si	54.951000	51.043000	48.979000	20.943000	30.475000	40.029000	28.419000	39.051000	40.178000
Si	55.681000	49.658000	51.475000	19.259000	32.420000	38.503000	30.149000	39.937000	37.752000
Si	58.449000	50.918000	51.667000	17.884000	33.206000	40.999000	28.711000	37.825000	35.978000
Si	57.677000	52.345000	49.192000	19.492000	31.028000	42.535000	26.916000	37.159000	38.416000
Si	56.659000	47.398000	49.947000	17.239000	30.329000	37.842000	31.958000	37.538000	38.491000
Si	59.462000	48.541000	49.926000	15.807000	31.042000	40.428000	30.470000	35.443000	36.710000
Si	58.918000	50.211000	47.488000	17.451000	29.068000	41.890000	28.671000	34.913000	39.142000
Si	31.995000	20.591000	50.378000	37.882000	21.909000	32.164000	55.139000	8.942000	37.532000
Si	28.998000	20.164000	47.865000	37.592000	18.946000	34.526000	51.748000	7.168000	37.067000
Si	30.477000	17.508000	47.608000	40.540000	19.301000	34.656000	50.910000	9.205000	39.420000
Si	30.560000	15.868000	50.178000	41.979000	21.714000	35.507000	51.145000	9.252000	42.463000
Si	29.940000	13.357000	48.678000	43.398000	20.233000	37.761000	50.899000	12.267000	42.441000
Si	32.932000	12.985000	48.534000	45.705000	19.777000	35.984000	47.920000	11.974000	42.691000
Si	33.584000	15.479000	50.132000	44.375000	21.121000	33.717000	48.074000	8.923000	42.503000
Si	30.269000	14.930000	46.013000	41.781000	17.784000	36.894000	50.800000	12.263000	39.492000
Si	33.368000	14.458000	45.902000	44.265000	17.243000	35.366000	47.676000	12.086000	39.698000
Si	33.458000	16.987000	47.558000	42.883000	18.616000	32.998000	47.921000	9.075000	39.530000
Si	46.447000	46.320000	46.194000	51.257000	50.795000	54.846000	9.917000	49.294000	40.128000
Si	43.378000	49.012000	45.113000	51.788000	49.275000	51.232000	10.181000	52.656000	37.870000
Si	45.505000	51.159000	44.810000	52.704000	48.507000	48.427000	11.458000	54.514000	35.848000
Si	43.874000	53.579000	44.179000	50.836000	46.994000	46.648000	13.807000	53.786000	34.167000
Si	45.771000	55.538000	45.630000	52.081000	48.159000	44.189000	12.305000	54.325000	31.638000
Si	47.280000	55.948000	43.191000	54.063000	45.857000	44.236000	13.363000	57.132000	31.726000
Si	45.509000	53.964000	41.579000	52.720000	44.601000	46.741000	14.901000	56.519000	34.256000
Si	47.224000	52.911000	46.382000	54.175000	49.610000	45.917000	10.002000	55.117000	33.247000
Si	48.842000	53.544000	43.868000	56.034000	47.465000	46.015000	10.982000	57.932000	33.461000
Si	47.070000	51.606000	42.365000	54.623000	46.211000	48.365000	12.666000	57.290000	35.948000

Si	28.427000	7.238000	35.373000	35.220000	36.546000	32.476000	9.221000	37.517000	30.540000
Si	30.049000	9.668000	35.511000	37.200000	35.871000	34.608000	10.964000	36.423000	32.760000
Si	28.587000	10.353000	38.035000	39.288000	35.530000	32.440000	13.297000	37.353000	31.304000
Si	30.768000	8.988000	39.694000	40.152000	38.396000	32.997000	13.165000	39.943000	32.791000
Si	32.166000	8.425000	37.063000	37.934000	38.727000	35.115000	10.718000	39.061000	34.275000
Si	26.976000	7.754000	38.001000	37.235000	36.331000	30.451000	11.535000	38.470000	29.018000
Si	29.230000	6.307000	39.422000	38.064000	39.166000	30.874000	11.662000	41.028000	30.522000
Si	30.649000	5.773000	36.814000	35.950000	39.476000	32.955000	9.172000	40.266000	31.842000
Si	55.209000	42.507000	40.955000	15.130000	22.650000	34.910000	36.517000	38.682000	45.448000
Si	57.127000	40.569000	40.162000	12.794000	24.446000	34.046000	37.172000	40.336000	43.047000
Si	56.612000	38.990000	42.636000	14.655000	26.293000	32.655000	36.473000	42.789000	44.388000
Si	54.730000	37.209000	41.251000	14.339000	24.917000	30.090000	39.396000	42.914000	45.322000
Si	55.008000	38.947000	38.744000	12.659000	22.868000	31.560000	39.964000	40.247000	44.074000
Si	54.609000	40.915000	43.542000	16.931000	24.654000	33.657000	35.896000	41.241000	46.779000
Si	52.710000	39.272000	42.037000	16.820000	23.389000	31.078000	38.687000	41.374000	47.814000
Si	52.927000	40.998000	39.543000	15.163000	21.294000	32.243000	39.186000	38.752000	46.565000
Si	33.039000	22.348000	52.608000	35.828000	23.457000	30.818000	57.849000	8.189000	36.259000
Si	35.021000	22.451000	55.003000	35.293000	26.211000	29.781000	57.626000	7.732000	33.338000
Si	35.070000	25.493000	55.012000	34.192000	25.109000	27.278000	60.317000	8.874000	32.970000
Si	32.538000	25.440000	56.704000	31.496000	25.275000	28.636000	61.624000	6.319000	33.082000
Si	32.520000	22.503000	56.839000	32.590000	26.376000	31.124000	58.983000	4.894000	33.564000
Si	33.296000	25.346000	52.532000	34.464000	22.284000	28.347000	60.615000	9.334000	35.936000
Si	30.754000	25.498000	54.280000	31.773000	22.403000	29.674000	61.949000	6.729000	36.076000
Si	30.783000	22.529000	54.502000	33.062000	23.439000	32.114000	59.260000	5.562000	36.418000
Si	46.334000	46.448000	49.222000	48.545000	49.685000	53.965000	12.487000	49.571000	41.754000
Si	47.603000	47.708000	51.755000	46.145000	50.386000	52.354000	14.267000	47.144000	41.147000
Si	45.087000	47.370000	53.071000	45.425000	47.496000	51.847000	16.714000	48.828000	41.138000
Si	45.952000	44.740000	54.247000	43.620000	47.613000	54.357000	17.194000	48.230000	44.022000
Si	48.621000	45.057000	52.768000	44.588000	50.371000	54.884000	14.647000	46.653000	44.031000
Si	43.686000	46.048000	50.671000	47.785000	46.752000	53.464000	14.993000	51.312000	41.715000
Si	44.691000	43.511000	51.827000	45.937000	46.713000	55.947000	15.492000	50.735000	44.637000
Si	47.323000	43.807000	50.364000	46.951000	49.435000	56.373000	13.057000	49.074000	44.702000
O	27.425000	6.797000	34.131000	33.667000	36.116000	32.286000	8.034000	36.678000	29.780000
O	27.304000	7.841000	36.396000	36.174000	35.741000	31.482000	10.390000	37.331000	29.424000
O	29.670000	8.218000	35.031000	35.851000	36.438000	33.957000	9.620000	36.918000	31.978000
O	29.166000	6.057000	36.204000	35.373000	38.119000	32.182000	9.003000	39.194000	30.656000
O	27.614000	9.178000	38.619000	38.692000	36.035000	31.018000	12.905000	38.277000	29.957000
O	31.586000	7.080000	36.433000	36.378000	39.011000	34.439000	9.525000	39.355000	33.203000
O	29.708000	7.756000	39.853000	39.478000	38.652000	31.547000	13.000000	40.584000	31.354000
O	28.816000	10.196000	36.424000	38.002000	35.147000	33.379000	12.105000	36.276000	31.661000
O	31.463000	9.710000	36.426000	38.156000	37.111000	35.222000	11.268000	37.562000	33.890000
O	29.942000	10.057000	38.801000	40.013000	36.807000	33.186000	13.472000	38.359000	32.545000
O	31.767000	8.288000	38.647000	39.014000	39.028000	33.977000	11.793000	40.081000	33.683000
O	27.860000	6.561000	38.596000	37.159000	37.896000	30.238000	11.195000	40.006000	29.287000
O	30.405000	5.740000	38.410000	37.262000	39.874000	32.161000	10.500000	41.215000	31.639000

O	27.389000	0.051000	32.651000	27.526000	35.921000	34.958000	4.358000	36.602000	32.974000
O	29.436000	-1.225000	31.602000	26.829000	35.685000	37.472000	2.579000	35.254000	31.509000
O	27.697000	-2.638000	32.994000	27.068000	38.057000	36.423000	2.732000	35.112000	34.169000
O	29.503000	-0.874000	34.036000	29.232000	36.770000	36.983000	4.355000	33.849000	32.486000
O	31.554000	-2.770000	30.975000	26.196000	36.044000	40.063000	0.499000	33.874000	30.770000
O	29.264000	-3.149000	35.035000	29.301000	39.307000	37.455000	3.435000	32.462000	34.552000
O	32.907000	-3.358000	33.065000	28.522000	37.240000	41.173000	1.070000	31.288000	31.178000
O	29.088000	-3.705000	30.910000	25.511000	37.827000	38.387000	0.396000	35.092000	33.147000
O	28.926000	-4.907000	33.148000	26.894000	40.078000	38.280000	1.066000	33.237000	34.836000
O	31.251000	-5.174000	31.913000	26.393000	38.632000	40.624000	-0.461000	32.515000	32.824000
O	31.283000	-4.730000	34.545000	28.718000	39.595000	40.170000	1.344000	30.935000	33.688000
O	31.907000	-0.873000	32.786000	28.587000	35.285000	39.342000	2.916000	33.043000	30.122000
O	31.646000	-2.190000	35.045000	30.145000	37.304000	39.188000	3.446000	31.309000	32.150000
O	55.167000	44.085000	40.604000	15.238000	21.927000	36.409000	35.538000	37.362000	45.527000
O	55.388000	41.946000	42.506000	16.027000	24.054000	34.903000	35.604000	40.002000	45.799000
O	56.131000	41.821000	39.890000	13.643000	23.136000	34.553000	37.109000	39.007000	43.990000
O	53.689000	42.105000	40.449000	15.742000	21.797000	33.722000	37.587000	38.417000	46.673000
O	55.432000	39.515000	43.617000	16.172000	25.786000	32.751000	36.201000	42.542000	45.920000
O	54.159000	40.337000	38.736000	13.557000	21.733000	32.258000	39.579000	39.022000	45.021000
O	53.732000	38.102000	42.211000	15.911000	24.555000	30.443000	38.742000	42.591000	46.776000
O	57.285000	40.156000	41.720000	13.929000	25.605000	33.938000	36.127000	41.491000	43.471000
O	56.516000	39.238000	39.358000	12.272000	24.070000	32.601000	38.724000	40.826000	43.234000
O	56.202000	37.781000	41.627000	13.942000	25.994000	31.229000	38.071000	43.013000	44.375000
O	54.394000	37.861000	39.813000	13.593000	23.557000	30.416000	40.150000	41.554000	45.063000
O	53.255000	40.610000	42.756000	17.391000	23.508000	32.583000	37.166000	40.843000	47.747000
O	52.529000	39.628000	40.405000	15.898000	22.044000	30.992000	39.526000	40.141000	47.229000
O	55.573000	48.345000	45.835000	19.217000	26.860000	38.848000	30.746000	36.452000	42.361000
O	55.885000	48.102000	48.653000	18.503000	29.283000	38.033000	31.384000	37.668000	40.025000
O	55.565000	50.389000	47.577000	20.093000	29.114000	40.110000	28.927000	37.661000	40.847000
O	57.841000	49.031000	47.166000	17.688000	28.461000	40.418000	29.964000	35.435000	40.007000
O	56.507000	48.263000	51.307000	17.847000	31.813000	37.985000	31.380000	38.839000	37.645000
O	58.051000	51.499000	47.854000	18.883000	29.485000	42.509000	27.452000	35.918000	39.286000
O	59.003000	49.520000	51.114000	16.593000	32.499000	40.321000	29.956000	36.846000	36.045000
O	54.790000	49.898000	50.110000	20.233000	31.132000	38.719000	29.616000	39.681000	39.279000
O	56.097000	52.101000	49.517000	20.628000	31.388000	41.359000	27.345000	38.546000	39.109000
O	56.820000	50.708000	51.793000	19.100000	33.030000	39.944000	29.020000	39.215000	36.835000
O	58.633000	51.834000	50.381000	18.302000	32.143000	42.208000	27.619000	37.118000	36.956000
O	58.259000	47.517000	49.487000	16.275000	30.244000	39.143000	31.404000	36.153000	37.809000
O	59.663000	49.724000	48.861000	16.444000	30.333000	41.706000	29.172000	34.891000	37.628000
O	32.947000	21.410000	51.323000	37.367000	23.011000	31.125000	56.724000	8.749000	37.286000
O	33.746000	23.767000	52.329000	35.618000	22.945000	29.270000	58.999000	9.274000	35.902000
O	33.969000	21.792000	53.947000	35.725000	25.040000	30.868000	57.216000	7.536000	34.946000
O	31.560000	22.609000	53.124000	34.512000	22.775000	31.559000	58.641000	6.974000	36.884000
O	34.051000	25.962000	53.803000	33.874000	23.518000	27.414000	61.134000	9.189000	34.369000
O	31.761000	21.791000	55.629000	33.210000	25.116000	32.054000	58.500000	5.132000	35.095000

O	31.769000	26.084000	55.425000	31.601000	23.696000	28.684000	62.270000	6.950000	34.438000
O	35.576000	23.978000	54.522000	35.360000	25.416000	28.395000	58.718000	8.941000	33.334000
O	34.016000	22.634000	56.286000	33.797000	26.903000	30.125000	58.289000	6.250000	32.888000
O	34.132000	25.430000	56.342000	32.828000	25.746000	27.837000	60.660000	7.417000	32.399000
O	32.000000	24.028000	56.990000	31.419000	25.772000	30.167000	60.539000	5.129000	33.605000
O	31.720000	25.214000	52.970000	33.242000	21.750000	29.291000	61.211000	8.015000	36.760000
O	30.389000	24.050000	54.915000	31.789000	22.999000	31.187000	60.908000	5.527000	36.254000
O	29.684000	18.861000	47.176000	39.118000	19.279000	34.013000	51.760000	8.270000	38.310000
O	29.842000	16.148000	46.985000	40.662000	18.754000	36.214000	51.440000	10.762000	39.623000
O	30.754000	17.148000	49.162000	41.089000	20.852000	34.468000	50.945000	8.616000	40.954000
O	31.992000	17.477000	47.037000	41.524000	18.437000	33.758000	49.388000	9.362000	38.947000
O	30.182000	13.708000	47.094000	42.934000	18.654000	37.665000	50.574000	12.851000	40.973000
O	33.407000	16.789000	49.165000	43.183000	20.209000	33.095000	48.119000	8.338000	40.977000
O	32.949000	13.273000	46.898000	45.181000	18.237000	36.293000	47.914000	12.591000	41.177000
O	29.712000	14.852000	49.274000	42.108000	20.908000	36.928000	51.555000	10.812000	42.390000
O	32.069000	15.366000	50.563000	43.546000	21.849000	34.923000	49.573000	9.188000	42.977000
O	31.499000	13.084000	49.177000	44.735000	20.611000	36.938000	49.470000	12.012000	43.209000
O	33.893000	14.107000	49.227000	45.551000	20.214000	34.431000	47.452000	10.418000	42.566000
O	31.916000	15.174000	45.564000	42.683000	17.029000	35.775000	49.189000	12.017000	39.105000
O	33.939000	15.565000	46.888000	44.041000	17.993000	33.931000	47.230000	10.549000	39.769000
O	46.201000	46.978000	47.679000	50.056000	50.246000	53.912000	11.084000	49.992000	41.041000
O	44.919000	46.776000	49.958000	48.296000	48.308000	53.169000	13.734000	50.413000	41.141000
O	47.478000	47.004000	50.278000	47.434000	50.668000	53.328000	12.997000	48.052000	41.696000
O	46.355000	44.881000	49.544000	48.257000	49.164000	55.460000	12.580000	49.879000	43.360000
O	43.905000	46.535000	52.239000	46.391000	46.488000	52.696000	16.266000	50.354000	41.617000
O	48.391000	44.651000	51.265000	46.038000	50.495000	55.598000	13.300000	47.523000	44.340000
O	44.732000	44.254000	53.253000	44.668000	46.520000	54.918000	16.740000	49.772000	44.187000
O	46.126000	48.197000	52.082000	46.266000	48.873000	51.656000	15.284000	48.251000	40.581000
O	48.206000	46.671000	52.785000	44.828000	50.453000	53.302000	14.833000	46.457000	42.453000
O	45.759000	46.384000	54.177000	44.197000	47.955000	52.841000	17.134000	47.881000	42.464000
O	47.409000	44.301000	53.595000	43.884000	48.927000	55.237000	15.921000	47.439000	44.722000
O	44.068000	44.445000	50.655000	47.326000	46.636000	55.078000	14.876000	51.432000	43.299000
O	46.242000	43.325000	51.448000	46.027000	48.151000	56.574000	14.443000	49.692000	45.235000
O	44.775000	49.796000	45.283000	52.476000	49.382000	49.733000	11.217000	53.683000	37.196000
O	45.950000	52.000000	46.129000	52.996000	49.329000	47.034000	10.546000	54.176000	34.466000
O	44.339000	52.042000	44.065000	51.633000	47.329000	48.090000	13.037000	54.047000	35.596000
O	46.753000	50.989000	43.816000	54.140000	47.790000	48.700000	11.595000	56.098000	36.069000
O	46.928000	54.548000	46.139000	53.439000	48.912000	44.648000	10.876000	55.002000	31.850000
O	45.752000	52.301000	41.707000	53.328000	45.243000	48.113000	14.061000	56.619000	35.587000
O	48.383000	55.136000	43.955000	55.281000	46.821000	44.658000	11.788000	57.454000	32.084000
O	44.575000	54.439000	45.375000	51.103000	48.052000	45.487000	12.713000	53.614000	33.026000
O	44.495000	54.244000	42.787000	51.505000	45.586000	46.165000	14.802000	54.992000	33.736000
O	45.986000	56.033000	44.141000	52.632000	46.615000	43.968000	13.393000	55.537000	31.542000
O	46.696000	54.920000	42.058000	53.827000	44.926000	45.569000	14.241000	57.396000	33.091000
O	48.275000	52.770000	45.192000	55.491000	48.920000	46.484000	10.075000	56.628000	33.874000

O	48.181000	52.686000	42.624000	55.596000	46.315000	47.072000	12.219000	58.093000	34.568000
C	35.809000	-4.867000	32.494000	28.459000	37.861000	44.845000	-1.259000	29.174000	30.264000
C	35.424000	-7.416000	32.307000	27.777000	40.107000	45.203000	-3.122000	28.383000	31.997000
C	34.804000	-6.016000	32.400000	27.698000	38.975000	44.173000	-2.034000	29.402000	31.562000
C	33.646000	-5.916000	33.332000	28.207000	39.498000	42.760000	-0.956000	29.642000	32.646000
C	35.161000	-0.698000	31.901000	31.852000	36.997000	42.220000	1.267000	30.865000	27.773000
C	36.887000	-0.807000	33.847000	32.116000	34.549000	43.178000	2.744000	28.920000	27.079000
C	35.416000	-0.720000	33.474000	31.149000	35.600000	42.506000	2.650000	30.168000	27.944000
C	34.416000	-1.576000	34.316000	30.483000	35.068000	41.183000	3.116000	30.165000	29.385000
C	30.501000	-5.323000	37.819000	31.415000	41.668000	39.405000	-0.956000	30.674000	36.750000
C	29.040000	-7.456000	37.879000	30.748000	43.707000	38.041000	0.601000	29.334000	38.147000
C	29.303000	-6.110000	37.171000	30.636000	42.113000	38.125000	0.433000	30.006000	36.787000
C	29.398000	-6.219000	35.641000	29.118000	41.784000	38.311000	1.582000	30.995000	36.544000
C	27.064000	-5.003000	28.868000	22.486000	38.143000	36.796000	-1.105000	34.361000	37.415000
C	24.874000	-6.045000	29.859000	22.639000	39.445000	34.839000	-1.668000	36.776000	37.287000
C	26.030000	-5.022000	30.032000	23.526000	38.752000	35.893000	-0.960000	35.624000	36.541000
C	26.680000	-5.142000	31.428000	24.640000	39.606000	36.411000	0.399000	35.981000	35.832000
C	30.254000	-2.609000	27.905000	22.819000	39.625000	40.429000	-2.835000	33.066000	29.952000
C	30.930000	-4.653000	26.480000	21.307000	37.836000	41.219000	-4.441000	34.746000	31.013000
C	31.125000	-3.844000	27.767000	22.590000	38.074000	40.396000	-3.278000	33.722000	31.261000
C	31.171000	-4.735000	29.017000	23.853000	37.370000	40.911000	-2.175000	34.506000	31.909000
C	31.851000	1.670000	28.610000	26.477000	30.841000	39.378000	0.862000	36.402000	26.914000
C	30.772000	2.212000	30.914000	27.609000	32.045000	37.476000	0.546000	33.885000	27.390000
C	31.826000	1.363000	30.159000	27.150000	32.108000	38.921000	0.658000	35.286000	27.948000
C	31.562000	-0.127000	30.082000	26.326000	33.368000	39.320000	1.940000	35.362000	28.771000
C	31.179000	-2.315000	38.656000	33.466000	39.207000	37.889000	5.248000	29.790000	35.086000
C	32.636000	-0.450000	37.370000	33.269000	37.036000	38.829000	5.770000	29.180000	32.597000
C	31.298000	-0.991000	37.883000	33.150000	37.754000	37.467000	6.066000	30.162000	33.808000
C	30.260000	-0.808000	36.815000	31.742000	37.579000	36.802000	5.921000	31.665000	33.476000
C	30.844000	11.023000	32.826000	36.017000	34.898000	37.231000	10.103000	35.034000	35.277000
C	30.702000	12.213000	31.864000	35.426000	33.720000	38.010000	9.579000	33.726000	35.969000
C	30.828000	13.582000	32.595000	36.672000	32.885000	38.454000	9.488000	32.429000	35.105000
C	29.982000	13.660000	33.933000	37.794000	32.641000	37.480000	10.068000	32.218000	33.678000
C	30.217000	12.469000	34.884000	38.180000	33.928000	36.616000	10.234000	33.624000	33.056000
C	30.091000	11.073000	34.244000	36.974000	34.682000	36.051000	10.693000	34.900000	33.821000
C	24.178000	8.830000	38.349000	35.774000	35.837000	27.992000	12.838000	37.310000	26.705000
C	22.699000	8.575000	38.696000	35.620000	35.191000	26.589000	13.432000	37.246000	25.362000
C	22.092000	7.292000	38.217000	36.091000	33.728000	26.433000	12.506000	38.007000	24.364000
C	22.917000	6.165000	37.573000	37.350000	33.483000	27.281000	11.891000	39.308000	24.790000
C	24.433000	6.374000	37.684000	37.251000	33.908000	28.731000	11.285000	39.250000	26.207000
C	25.131000	7.613000	38.256000	36.969000	35.414000	28.854000	12.117000	38.597000	27.235000
C	26.308000	12.395000	38.285000	40.682000	33.258000	33.778000	16.172000	37.165000	30.503000
C	25.716000	13.734000	38.755000	41.733000	32.113000	33.750000	17.212000	36.152000	30.065000
C	25.868000	14.120000	40.254000	41.314000	31.138000	32.647000	16.911000	35.402000	28.738000
C	27.302000	13.580000	40.668000	40.792000	31.669000	31.304000	15.547000	34.738000	28.655000

C	27.756000	12.232000	40.137000	39.815000	32.834000	31.561000	14.457000	35.652000	29.232000
C	27.716000	11.913000	38.686000	40.341000	33.947000	32.414000	14.762000	36.481000	30.506000
C	30.929000	10.168000	42.448000	42.169000	39.765000	34.908000	16.023000	39.714000	33.330000
C	31.839000	10.313000	43.683000	43.312000	40.848000	34.658000	17.286000	40.005000	34.162000
C	33.094000	11.181000	43.514000	44.581000	40.335000	33.945000	17.414000	41.425000	34.834000
C	33.780000	10.803000	42.153000	44.235000	39.645000	32.701000	16.097000	42.159000	35.146000
C	32.872000	10.735000	40.903000	42.960000	38.765000	32.644000	15.007000	41.965000	34.049000
C	31.679000	9.762000	41.139000	41.738000	39.188000	33.448000	14.773000	40.526000	33.605000
C	28.439000	3.773000	40.524000	37.397000	40.490000	28.396000	10.459000	43.122000	28.957000
C	28.197000	3.006000	41.826000	37.710000	41.324000	27.138000	10.267000	44.570000	28.535000
C	29.464000	2.981000	42.732000	38.545000	42.541000	27.471000	10.863000	45.695000	29.460000
C	29.701000	4.437000	43.128000	39.609000	42.442000	28.572000	12.229000	45.315000	30.138000
C	29.928000	5.418000	41.957000	39.278000	41.500000	29.719000	12.211000	43.915000	30.668000
C	28.941000	5.200000	40.829000	38.513000	40.173000	29.433000	11.657000	42.736000	29.845000
C	34.877000	9.613000	36.614000	36.958000	39.902000	37.599000	8.762000	39.029000	36.483000
C	36.155000	9.595000	35.802000	37.145000	40.641000	38.940000	8.123000	39.227000	37.886000
C	36.985000	8.369000	36.192000	38.581000	40.652000	39.521000	9.185000	39.334000	38.974000
C	36.134000	7.153000	36.096000	39.510000	41.075000	38.343000	10.501000	39.816000	38.444000
C	34.701000	7.060000	36.544000	39.493000	39.875000	37.389000	11.149000	39.230000	37.161000
C	33.912000	8.373000	36.521000	38.151000	39.782000	36.652000	10.152000	39.427000	36.014000
C	32.963000	3.970000	35.982000	34.314000	41.831000	33.766000	6.822000	42.024000	31.335000
C	33.451000	2.978000	34.902000	32.822000	42.330000	33.661000	5.380000	42.247000	31.864000
C	32.554000	1.678000	34.827000	31.959000	41.711000	34.836000	5.400000	42.902000	33.248000
C	31.062000	1.925000	34.965000	31.855000	40.231000	34.402000	6.255000	42.116000	34.272000
C	30.696000	2.846000	36.133000	33.218000	39.612000	34.122000	7.617000	41.828000	33.671000
C	31.444000	4.170000	36.011000	34.317000	40.364000	33.231000	7.656000	41.167000	32.328000
C	28.119000	2.650000	33.480000	29.945000	36.688000	33.962000	6.973000	36.039000	33.039000
C	27.414000	5.819000	31.748000	33.470000	34.554000	34.637000	7.369000	34.278000	28.716000
C	25.202000	5.103000	33.921000	31.784000	34.390000	31.814000	5.387000	36.200000	29.777000
C	27.954000	4.023000	34.112000	31.322000	36.760000	33.187000	7.027000	34.992000	31.947000
C	26.862000	0.380000	35.281000	27.966000	35.818000	32.226000	5.993000	37.972000	34.856000
C	25.304000	2.013000	32.959000	29.293000	33.842000	34.630000	5.793000	38.613000	31.601000
C	57.756000	52.335000	55.088000	17.330000	35.192000	43.936000	29.251000	40.015000	33.677000
C	59.797000	53.607000	54.639000	16.288000	36.831000	42.306000	27.879000	38.919000	31.785000
C	58.723000	52.795000	53.971000	16.620000	35.368000	42.577000	27.960000	39.188000	33.343000
C	59.315000	51.690000	53.139000	17.528000	34.988000	41.410000	28.199000	37.939000	34.170000
C	60.668000	47.079000	52.784000	13.982000	32.215000	42.853000	29.254000	33.760000	34.261000
C	62.254000	45.576000	51.554000	12.050000	32.869000	41.166000	31.600000	32.897000	33.483000
C	60.937000	46.386000	51.422000	13.536000	32.592000	41.407000	30.802000	33.991000	34.184000
C	60.983000	47.540000	50.375000	13.979000	31.464000	40.519000	31.414000	34.366000	35.533000
C	59.372000	54.025000	46.792000	17.842000	31.396000	45.314000	23.784000	36.921000	40.261000
C	60.671000	53.439000	48.804000	19.927000	31.600000	46.755000	22.628000	37.727000	38.173000
C	59.499000	54.334000	48.274000	19.336000	31.022000	45.459000	23.975000	37.665000	38.931000
C	58.182000	54.166000	48.892000	20.175000	31.441000	44.229000	25.091000	36.919000	38.149000
C	53.758000	53.882000	47.166000	23.135000	29.502000	42.344000	27.454000	42.108000	40.291000

C	52.916000	51.569000	46.320000	24.021000	28.020000	40.328000	25.460000	40.513000	40.749000
C	52.941000	52.625000	47.428000	23.707000	29.451000	40.922000	26.754000	41.175000	41.333000
C	53.260000	51.875000	48.736000	22.822000	30.341000	40.000000	27.748000	40.068000	41.522000
C	53.811000	48.672000	55.311000	20.117000	35.858000	36.083000	32.465000	43.512000	38.210000
C	55.185000	50.740000	54.765000	19.696000	35.627000	38.645000	33.448000	41.332000	37.379000
C	54.917000	49.243000	54.434000	19.420000	35.109000	37.253000	32.275000	42.033000	38.063000
C	54.489000	49.257000	52.911000	19.786000	33.602000	37.208000	30.872000	41.706000	37.487000
C	57.912000	44.143000	49.394000	13.937000	28.955000	37.237000	34.865000	35.169000	38.197000
C	55.677000	44.749000	48.221000	15.815000	27.270000	36.970000	34.310000	35.923000	40.449000
C	56.402000	44.375000	49.553000	15.227000	28.642000	36.459000	34.799000	36.401000	39.024000
C	56.186000	45.694000	50.388000	16.189000	29.813000	36.333000	33.816000	37.533000	38.570000
C	61.676000	49.505000	44.301000	14.788000	26.441000	44.095000	28.861000	30.950000	40.190000
C	61.335000	48.231000	46.565000	14.275000	27.733000	42.126000	30.785000	32.122000	39.007000
C	60.707000	49.053000	45.431000	15.475000	27.010000	42.769000	29.656000	32.263000	40.076000
C	60.138000	50.288000	46.159000	16.675000	27.905000	43.144000	28.501000	33.254000	39.820000
C	59.117000	40.954000	38.032000	10.713000	26.328000	34.582000	35.977000	39.877000	40.427000
C	60.509000	41.358000	37.437000	9.446000	26.769000	35.362000	36.192000	39.802000	38.902000
C	61.187000	42.494000	38.191000	8.446000	25.699000	35.799000	37.394000	39.082000	38.371000
C	61.000000	42.582000	39.682000	9.068000	24.406000	36.404000	38.635000	39.232000	39.222000
C	59.874000	41.712000	40.339000	10.317000	24.022000	35.586000	38.402000	39.056000	40.709000
C	58.839000	40.866000	39.496000	11.336000	24.981000	34.924000	37.234000	39.963000	41.218000
C	53.586000	40.564000	46.245000	18.553000	26.778000	35.043000	33.104000	41.555000	46.635000
C	53.556000	41.061000	47.684000	18.604000	28.117000	34.340000	31.703000	41.732000	47.217000
C	53.133000	42.516000	47.973000	19.568000	28.282000	33.097000	31.419000	42.763000	48.356000
C	52.814000	43.354000	46.732000	19.961000	26.997000	32.373000	32.538000	42.572000	49.392000
C	53.435000	42.820000	45.393000	19.704000	25.639000	33.186000	33.995000	42.316000	48.843000
C	54.252000	41.533000	45.227000	18.515000	25.544000	34.105000	34.264000	41.419000	47.635000
C	58.828000	39.451000	44.572000	16.046000	28.870000	32.334000	35.187000	45.482000	44.891000
C	59.720000	38.956000	45.726000	16.213000	30.396000	32.660000	34.380000	46.682000	44.312000
C	59.241000	37.703000	46.556000	14.928000	31.182000	33.114000	33.562000	46.482000	42.988000
C	58.533000	36.595000	45.712000	13.985000	30.319000	34.026000	34.034000	45.387000	42.081000
C	57.579000	37.334000	44.809000	13.771000	28.921000	33.361000	34.308000	44.033000	42.930000
C	58.015000	38.453000	43.789000	14.943000	28.076000	32.970000	35.257000	44.150000	44.114000
C	54.489000	34.696000	42.827000	15.217000	26.315000	27.568000	41.901000	44.544000	45.296000
C	53.975000	33.232000	42.946000	15.178000	26.296000	26.022000	42.426000	45.713000	44.390000
C	54.839000	32.438000	41.960000	13.862000	26.000000	25.278000	42.027000	47.103000	45.019000
C	54.900000	33.006000	40.475000	13.090000	24.729000	25.800000	40.476000	47.182000	45.061000
C	55.248000	34.446000	40.346000	13.077000	24.791000	27.371000	39.787000	45.911000	45.667000
C	54.510000	35.355000	41.404000	14.304000	25.167000	28.277000	40.386000	44.516000	45.342000
C	50.174000	40.110000	43.515000	19.322000	21.994000	30.400000	38.472000	42.876000	50.266000
C	49.089000	39.671000	44.583000	20.577000	21.626000	29.675000	38.538000	42.877000	51.828000
C	48.135000	38.486000	44.176000	20.431000	21.529000	28.130000	40.079000	42.850000	52.250000
C	49.228000	37.388000	43.993000	19.393000	22.572000	27.595000	41.145000	42.296000	51.357000
C	50.195000	37.721000	42.896000	18.065000	22.840000	28.372000	40.819000	41.834000	49.949000
C	51.139000	39.004000	43.051000	18.258000	23.003000	29.908000	39.333000	41.760000	49.524000

C	53.732000	37.853000	36.334000	11.251000	21.428000	29.444000	42.219000	41.188000	42.332000
C	53.756000	37.322000	34.866000	10.070000	20.950000	28.646000	43.553000	40.849000	41.860000
C	55.073000	36.594000	34.424000	8.918000	20.477000	29.572000	43.745000	39.499000	41.112000
C	56.295000	37.314000	34.934000	8.635000	21.376000	30.798000	43.116000	38.369000	41.909000
C	56.397000	37.466000	36.477000	9.930000	21.583000	31.618000	41.624000	38.654000	42.187000
C	55.095000	37.981000	37.152000	11.126000	22.097000	30.745000	41.504000	39.967000	43.016000
C	50.109000	41.694000	39.191000	16.853000	18.943000	32.002000	41.383000	37.073000	47.469000
C	48.826000	41.705000	38.373000	17.290000	17.479000	31.943000	41.782000	35.673000	48.197000
C	48.926000	41.672000	36.871000	16.464000	16.661000	32.959000	41.109000	34.399000	47.658000
C	50.215000	41.357000	36.084000	14.969000	16.924000	32.525000	39.579000	34.658000	47.513000
C	51.239000	40.817000	37.102000	14.727000	18.399000	32.962000	39.311000	35.850000	46.595000
C	51.464000	41.458000	38.492000	15.440000	19.496000	32.129000	39.881000	37.146000	47.151000
C	55.050000	46.462000	44.093000	18.160000	24.593000	37.492000	32.596000	34.990000	43.678000
C	53.684000	46.403000	40.462000	17.285000	20.568000	38.032000	35.894000	34.880000	45.996000
C	56.541000	46.594000	40.300000	15.447000	22.748000	39.220000	36.283000	36.127000	43.044000
C	55.170000	45.308000	43.044000	17.784000	23.091000	37.307000	33.478000	36.024000	44.377000
C	57.719000	46.586000	45.285000	17.548000	25.341000	40.746000	29.687000	33.804000	42.967000
C	56.255000	48.894000	43.189000	19.967000	24.239000	39.798000	30.061000	35.878000	45.055000
C	32.993000	8.826000	49.066000	49.923000	20.305000	37.198000	44.417000	13.570000	44.425000
C	31.119000	9.951000	48.027000	47.793000	20.267000	38.569000	44.512000	12.398000	42.264000
C	32.642000	9.988000	48.193000	48.487000	19.762000	37.280000	45.152000	12.414000	43.719000
C	33.406000	11.214000	48.906000	47.559000	20.245000	36.140000	46.720000	12.802000	43.830000
C	33.929000	16.332000	43.209000	43.442000	14.549000	33.472000	47.817000	12.128000	36.354000
C	35.107000	14.325000	42.021000	45.306000	13.275000	34.545000	45.741000	13.437000	36.103000
C	34.784000	14.996000	43.407000	44.844000	14.664000	34.066000	46.522000	12.516000	37.062000
C	34.180000	13.959000	44.308000	44.995000	15.594000	35.263000	46.798000	13.136000	38.462000
C	36.958000	15.868000	50.703000	46.909000	20.154000	31.604000	44.939000	7.710000	42.931000
C	36.695000	16.688000	52.998000	47.067000	22.402000	30.573000	45.774000	5.749000	44.431000
C	35.946000	16.573000	51.652000	46.088000	21.441000	31.230000	46.112000	6.772000	43.313000
C	34.601000	15.799000	51.640000	45.466000	22.098000	32.487000	47.377000	7.589000	43.575000
C	27.498000	17.175000	50.969000	39.192000	22.862000	36.570000	54.341000	8.828000	42.407000
C	27.558000	14.954000	51.946000	40.931000	23.429000	38.310000	53.739000	9.847000	44.687000
C	28.297000	16.295000	51.947000	40.363000	23.760000	36.954000	53.639000	8.633000	43.791000
C	29.794000	16.263000	51.792000	41.531000	23.441000	35.943000	52.217000	8.207000	43.599000
C	26.128000	11.182000	48.823000	44.361000	20.679000	41.998000	53.436000	14.999000	44.769000
C	26.390000	13.479000	48.947000	44.845000	18.871000	40.543000	51.232000	15.480000	43.637000
C	27.146000	12.297000	48.430000	44.610000	20.400000	40.480000	52.128000	14.413000	44.272000
C	28.601000	12.087000	48.967000	43.413000	20.813000	39.523000	52.216000	13.217000	43.351000
C	30.073000	13.493000	42.811000	42.902000	14.854000	38.080000	50.013000	14.800000	37.475000
C	28.727000	12.262000	44.404000	42.615000	16.508000	39.921000	52.028000	15.420000	38.896000
C	28.844000	13.605000	43.771000	41.835000	15.675000	38.885000	51.486000	14.648000	37.746000
C	28.998000	14.871000	44.642000	41.012000	16.555000	38.069000	51.679000	13.189000	38.059000
C	36.985000	17.437000	46.796000	45.290000	17.064000	30.893000	46.492000	6.029000	39.693000
C	36.922000	19.599000	48.322000	43.643000	17.082000	28.971000	45.801000	6.125000	37.282000
C	36.289000	18.251000	47.898000	44.117000	17.797000	30.250000	46.773000	6.605000	38.295000

C	34.801000	18.298000	47.468000	42.938000	17.915000	31.192000	46.723000	8.163000	38.433000
C	37.601000	21.655000	56.265000	38.071000	27.090000	30.247000	54.890000	7.324000	32.670000
C	38.566000	20.465000	56.422000	38.797000	28.321000	30.710000	53.576000	8.117000	32.314000
C	39.099000	19.797000	55.192000	38.624000	29.633000	29.966000	53.281000	8.562000	30.866000
C	37.945000	19.430000	54.236000	37.148000	30.015000	29.720000	54.627000	8.867000	30.250000
C	36.692000	20.344000	54.055000	36.339000	28.800000	29.243000	56.022000	8.656000	30.968000
C	36.466000	21.307000	55.271000	36.596000	27.449000	29.995000	56.127000	8.188000	32.406000
C	32.700000	27.666000	50.978000	34.254000	19.789000	26.910000	62.687000	11.077000	36.952000
C	32.671000	28.382000	49.589000	34.959000	18.537000	26.385000	63.277000	12.347000	37.542000
C	34.057000	28.402000	48.987000	36.108000	18.819000	25.361000	62.362000	12.939000	38.702000
C	34.764000	27.048000	48.808000	37.085000	19.985000	25.623000	60.899000	12.483000	38.933000
C	34.798000	26.212000	50.083000	36.471000	21.087000	26.476000	60.485000	11.219000	38.198000
C	33.451000	26.285000	50.922000	35.223000	20.917000	27.330000	61.164000	10.850000	36.812000
C	36.321000	27.985000	56.192000	35.395000	24.616000	24.691000	62.102000	10.673000	31.260000
C	37.529000	29.000000	56.452000	35.673000	24.983000	23.182000	62.548000	11.909000	30.499000
C	38.797000	28.808000	55.512000	35.408000	26.396000	22.632000	61.499000	11.998000	29.393000
C	39.125000	27.254000	55.410000	34.182000	26.977000	23.402000	59.972000	11.717000	29.675000
C	37.967000	26.283000	55.104000	34.382000	26.908000	24.904000	59.839000	10.347000	30.441000
C	36.495000	26.606000	55.524000	34.415000	25.480000	25.477000	60.656000	10.312000	31.736000
C	33.420000	27.964000	58.023000	30.104000	26.240000	26.404000	64.176000	4.983000	32.741000
C	33.843000	28.659000	59.332000	28.744000	26.132000	25.577000	65.308000	4.448000	31.873000
C	32.595000	29.034000	60.204000	27.525000	26.779000	26.201000	65.910000	5.717000	31.195000
C	31.663000	27.848000	60.249000	27.541000	26.718000	27.743000	65.066000	6.730000	30.436000
C	31.452000	26.959000	59.063000	28.661000	25.902000	28.360000	63.863000	7.100000	31.404000
C	32.651000	26.658000	58.138000	30.076000	26.057000	27.882000	63.138000	5.888000	32.084000
C	27.934000	26.358000	53.802000	29.093000	21.168000	29.944000	63.126000	5.721000	38.562000
C	27.059000	27.498000	53.222000	28.044000	20.119000	29.555000	64.357000	5.468000	39.473000
C	27.432000	28.985000	53.178000	28.036000	19.546000	28.041000	65.488000	6.482000	39.534000
C	28.581000	29.446000	54.052000	29.509000	19.400000	27.548000	65.878000	6.941000	38.116000
C	29.725000	28.333000	53.896000	30.745000	20.177000	28.113000	64.644000	7.288000	37.222000
C	29.388000	26.851000	54.264000	30.543000	20.961000	29.423000	63.459000	6.274000	37.124000
C	32.348000	22.545000	59.887000	31.001000	28.654000	30.785000	58.829000	1.896000	33.329000
C	32.348000	21.843000	61.210000	29.831000	29.556000	31.343000	58.453000	0.702000	32.398000
C	32.952000	20.412000	61.270000	30.375000	30.458000	32.423000	56.976000	0.651000	31.880000
C	32.981000	19.543000	60.034000	31.342000	29.821000	33.491000	56.500000	2.033000	31.349000
C	33.130000	20.272000	58.666000	32.352000	28.724000	32.940000	56.918000	3.224000	32.274000
C	32.397000	21.650000	58.533000	31.738000	27.798000	31.903000	58.303000	3.285000	32.911000
C	28.033000	22.554000	53.574000	32.891000	21.558000	34.310000	58.907000	2.876000	37.881000
C	26.702000	21.775000	53.498000	32.764000	21.183000	35.796000	57.989000	1.911000	38.670000
C	26.642000	20.196000	53.276000	31.619000	21.909000	36.511000	56.529000	1.894000	38.241000
C	27.721000	19.488000	54.078000	31.506000	23.384000	36.125000	55.906000	3.260000	37.903000
C	29.080000	20.260000	53.919000	31.520000	23.591000	34.609000	56.940000	4.433000	37.914000
C	29.119000	21.761000	54.353000	32.762000	22.989000	33.850000	58.437000	4.299000	37.651000
C	29.060000	20.021000	49.750000	36.700000	20.603000	34.619000	53.065000	7.762000	35.824000
C	32.437000	18.892000	50.999000	38.952000	22.796000	33.335000	54.380000	10.585000	37.779000

C	32.883000	20.898000	48.772000	39.207000	21.078000	31.261000	54.788000	7.727000	38.932000
C	30.133000	20.767000	50.621000	36.492000	21.503000	33.375000	54.570000	7.838000	36.237000
C	27.189000	20.411000	47.239000	37.622000	18.366000	36.247000	50.017000	7.017000	36.305000
C	29.858000	21.525000	46.943000	36.900000	17.971000	33.011000	52.116000	5.628000	37.988000
C	50.115000	57.639000	42.722000	54.821000	42.382000	44.278000	12.927000	60.457000	31.239000
C	48.917000	59.669000	41.727000	56.027000	42.764000	42.137000	15.560000	59.800000	30.988000
C	48.847000	58.488000	42.693000	55.580000	43.410000	43.404000	14.016000	59.649000	30.477000
C	47.724000	57.500000	42.252000	54.625000	44.619000	43.035000	13.832000	58.113000	30.324000
C	53.141000	53.505000	43.435000	60.219000	46.574000	45.295000	8.010000	61.070000	33.399000
C	51.861000	55.582000	44.424000	58.525000	45.850000	43.586000	9.858000	60.999000	35.158000
C	51.806000	54.274000	43.508000	58.685000	46.417000	45.057000	8.954000	60.121000	34.223000
C	50.665000	53.310000	43.881000	57.862000	47.563000	45.644000	9.729000	59.332000	33.139000
C	43.352000	52.896000	39.405000	52.170000	40.234000	47.244000	18.975000	56.973000	35.592000
C	44.122000	54.676000	37.539000	52.593000	41.976000	49.193000	18.381000	55.047000	33.974000
C	43.903000	54.284000	39.019000	52.680000	41.620000	47.716000	17.874000	56.048000	35.033000
C	45.189000	54.515000	39.777000	51.982000	42.837000	47.041000	16.698000	56.890000	34.493000
C	42.009000	52.439000	46.488000	48.546000	48.865000	48.472000	14.829000	51.320000	32.378000
C	41.747000	54.904000	46.816000	48.101000	48.969000	45.857000	17.049000	52.285000	33.242000
C	41.479000	53.707000	45.808000	48.124000	48.174000	47.199000	15.714000	51.555000	33.573000
C	42.019000	53.680000	44.310000	48.961000	46.889000	46.948000	14.833000	52.284000	34.527000
C	46.950000	58.524000	46.937000	53.281000	49.293000	41.138000	11.891000	54.891000	28.252000
C	44.712000	59.103000	47.933000	50.799000	49.747000	40.480000	12.010000	52.475000	27.682000
C	45.458000	58.229000	46.871000	51.741000	48.921000	41.283000	12.456000	53.504000	28.725000
C	45.003000	56.736000	46.819000	51.266000	48.812000	42.728000	12.079000	53.217000	30.127000
C	47.869000	49.573000	47.873000	56.585000	50.956000	44.179000	7.844000	56.507000	31.576000
C	49.982000	50.829000	47.397000	54.578000	51.934000	43.024000	7.945000	54.231000	30.555000
C	48.669000	50.852000	48.199000	55.382000	51.945000	44.334000	7.452000	55.034000	31.802000
C	47.961000	52.219000	47.943000	54.632000	51.400000	45.613000	8.240000	54.655000	33.053000
C	46.155000	48.473000	41.928000	57.642000	47.008000	49.588000	10.273000	58.600000	38.010000
C	45.984000	49.541000	39.635000	56.113000	47.571000	51.440000	11.719000	57.413000	39.575000
C	46.925000	49.144000	40.767000	56.677000	46.422000	50.584000	11.618000	58.567000	38.550000
C	47.834000	50.248000	41.363000	55.568000	45.667000	49.855000	12.612000	58.496000	37.373000
C	50.243000	48.908000	51.493000	46.084000	53.059000	51.567000	12.540000	44.921000	40.277000
C	51.297000	50.050000	51.766000	46.208000	54.239000	50.571000	12.372000	43.629000	39.474000
C	50.944000	51.217000	52.727000	47.614000	54.226000	49.864000	12.431000	43.883000	37.950000
C	49.446000	51.425000	52.614000	47.831000	52.883000	49.211000	13.027000	45.233000	37.523000
C	48.643000	50.130000	52.929000	47.639000	51.654000	50.160000	13.341000	46.362000	38.508000
C	48.693000	49.201000	51.797000	46.388000	51.679000	51.123000	13.669000	45.887000	39.925000
C	41.434000	45.749000	48.879000	50.278000	45.164000	53.835000	15.108000	52.977000	39.299000
C	39.971000	46.035000	48.401000	51.247000	44.015000	53.442000	14.912000	54.364000	38.600000
C	39.769000	47.554000	48.366000	51.451000	43.916000	51.893000	14.363000	55.686000	39.250000
C	40.233000	48.201000	49.664000	50.058000	43.778000	51.289000	14.179000	55.522000	40.778000
C	41.623000	47.918000	50.213000	49.106000	44.928000	51.562000	13.955000	54.120000	41.242000
C	41.971000	46.435000	50.128000	48.949000	45.334000	53.080000	14.941000	52.974000	40.816000
C	45.141000	49.511000	55.250000	44.570000	48.083000	49.018000	18.083000	49.677000	38.616000

C	44.709000	50.874000	55.804000	44.472000	47.614000	47.550000	19.385000	50.034000	37.966000
C	43.520000	51.609000	55.123000	43.757000	46.311000	47.154000	20.231000	48.781000	37.608000
C	42.668000	50.655000	54.286000	43.586000	45.419000	48.433000	20.079000	47.749000	38.704000
C	43.526000	49.785000	53.329000	44.243000	45.825000	49.777000	18.716000	47.553000	39.294000
C	44.394000	48.801000	54.071000	45.089000	47.079000	50.057000	18.121000	48.851000	39.859000
C	44.609000	44.612000	56.918000	41.195000	46.671000	55.964000	19.410000	46.662000	45.246000
C	44.922000	45.010000	58.368000	39.736000	46.079000	56.103000	20.617000	46.455000	46.139000
C	45.932000	44.089000	59.080000	38.793000	47.109000	55.349000	21.873000	47.338000	45.849000
C	47.241000	43.988000	58.301000	39.259000	47.744000	53.989000	21.355000	48.722000	45.346000
C	47.126000	43.860000	56.795000	40.737000	48.151000	54.047000	20.043000	48.874000	44.554000
C	45.848000	44.280000	56.010000	41.798000	47.280000	54.647000	18.809000	48.027000	44.964000
C	42.392000	42.027000	52.881000	45.769000	43.906000	56.685000	16.554000	51.071000	47.506000
C	41.470000	40.763000	53.171000	45.570000	42.700000	57.674000	16.653000	52.082000	48.636000
C	41.290000	39.843000	51.956000	46.530000	42.865000	58.817000	17.775000	52.997000	48.106000
C	42.702000	39.735000	51.295000	46.431000	44.234000	59.519000	17.516000	53.912000	46.878000
C	43.620000	40.914000	50.987000	46.510000	45.425000	58.587000	17.126000	52.891000	45.707000
C	43.791000	41.931000	52.252000	45.727000	45.377000	57.277000	16.158000	51.713000	46.036000
C	50.769000	42.952000	52.963000	43.888000	53.201000	55.154000	13.356000	44.059000	44.318000
C	51.970000	42.163000	53.538000	43.078000	54.433000	55.641000	13.287000	42.540000	44.562000
C	52.587000	42.858000	54.731000	41.545000	54.274000	55.416000	14.604000	41.790000	44.526000
C	52.466000	44.423000	54.755000	41.003000	52.825000	55.557000	15.624000	42.658000	45.325000
C	50.924000	44.696000	54.670000	41.854000	51.659000	55.107000	15.836000	44.069000	44.815000
C	50.331000	44.404000	53.296000	43.411000	51.751000	55.435000	14.549000	44.945000	44.793000
C	46.661000	41.404000	48.961000	48.153000	49.933000	59.021000	10.771000	50.424000	46.252000
C	46.932000	39.970000	48.768000	48.125000	50.266000	60.511000	9.682000	50.277000	47.257000
C	48.031000	39.738000	47.696000	46.911000	50.973000	61.069000	10.213000	49.603000	48.539000
C	49.131000	40.806000	47.692000	45.584000	51.098000	60.257000	10.574000	48.171000	48.207000
C	48.872000	42.179000	48.349000	45.689000	50.664000	58.782000	11.846000	48.264000	47.294000
C	47.814000	42.261000	49.490000	47.000000	50.283000	58.037000	11.790000	49.217000	46.068000
C	43.950000	47.255000	44.832000	52.913000	49.497000	52.701000	11.204000	51.093000	37.959000
C	47.253000	44.760000	46.648000	50.509000	50.647000	56.547000	8.268000	50.031000	40.668000
C	47.416000	47.763000	45.399000	51.635000	52.527000	54.308000	10.288000	47.416000	40.127000
C	44.653000	46.228000	45.699000	52.399000	49.494000	54.097000	10.691000	49.703000	38.455000
C	42.978000	49.193000	43.310000	50.974000	47.627000	51.087000	8.724000	52.503000	36.682000
C	42.479000	48.509000	46.664000	50.435000	50.495000	50.907000	9.647000	53.256000	39.555000
H	35.508000	-3.955000	32.049000	28.486000	37.065000	44.145000	-0.509000	29.985000	30.312000
H	36.497000	-5.185000	31.724000	27.858000	37.462000	45.634000	-1.933000	29.323000	29.383000
H	36.268000	-4.699000	33.476000	29.399000	38.211000	45.310000	-0.633000	28.322000	30.231000
H	34.830000	-8.289000	32.330000	27.186000	40.779000	44.567000	-3.378000	29.040000	32.826000
H	36.177000	-7.541000	33.101000	28.847000	40.307000	45.088000	-2.807000	27.433000	32.437000
H	36.043000	-7.302000	31.423000	27.330000	39.803000	46.164000	-3.987000	28.134000	31.448000
H	34.290000	-6.207000	31.519000	26.662000	38.860000	43.838000	-2.765000	30.223000	31.324000
H	33.102000	-6.774000	32.899000	27.373000	40.159000	42.719000	-1.842000	29.903000	33.274000
H	33.768000	-6.046000	34.406000	29.137000	39.998000	42.718000	-0.358000	28.877000	33.009000
H	34.155000	-0.457000	32.136000	31.105000	37.536000	41.663000	1.433000	31.769000	28.242000

H	35.133000	-1.591000	31.345000	32.824000	37.212000	41.747000	0.792000	29.994000	28.286000
H	35.799000	0.011000	31.474000	31.948000	37.490000	43.189000	1.414000	30.839000	26.678000
H	37.424000	-0.140000	33.175000	32.709000	35.075000	43.922000	2.520000	29.062000	26.053000
H	37.108000	-1.807000	33.542000	32.754000	33.993000	42.505000	1.964000	28.282000	27.490000
H	37.037000	-0.590000	34.898000	31.449000	33.938000	43.832000	3.715000	28.494000	26.975000
H	35.044000	0.289000	33.520000	30.372000	35.723000	43.233000	3.149000	30.985000	27.465000
H	35.083000	-2.318000	34.147000	31.275000	35.006000	40.519000	2.455000	29.343000	29.783000
H	34.445000	-1.401000	35.376000	29.947000	34.103000	41.268000	4.144000	29.853000	29.614000
H	30.211000	-5.085000	38.797000	32.427000	42.040000	39.300000	-1.747000	30.083000	37.290000
H	30.692000	-4.493000	37.248000	31.377000	40.635000	39.629000	-1.334000	31.202000	35.812000
H	31.456000	-5.899000	38.017000	30.997000	42.205000	40.131000	-0.653000	31.459000	37.371000
H	28.796000	-7.480000	38.928000	31.804000	43.879000	38.055000	-0.237000	28.686000	38.398000
H	29.690000	-8.307000	37.698000	30.587000	43.958000	39.072000	0.294000	30.161000	38.742000
H	28.137000	-7.726000	37.422000	30.289000	44.512000	37.458000	1.534000	28.907000	38.448000
H	28.464000	-5.465000	37.309000	31.244000	41.755000	37.296000	0.571000	29.204000	36.030000
H	30.299000	-6.758000	35.537000	29.053000	42.381000	39.242000	1.404000	31.718000	37.341000
H	28.590000	-6.698000	35.071000	28.394000	42.241000	37.608000	2.584000	30.644000	36.822000
H	26.914000	-4.945000	27.793000	21.966000	39.049000	37.064000	-0.256000	34.325000	38.027000
H	27.621000	-5.947000	28.940000	23.246000	37.737000	37.495000	-1.005000	33.437000	36.835000
H	27.636000	-4.107000	29.113000	22.059000	37.544000	36.030000	-2.050000	34.387000	38.003000
H	24.757000	-5.919000	28.785000	21.984000	38.682000	34.483000	-2.713000	36.526000	37.525000
H	23.912000	-5.910000	30.362000	23.379000	39.778000	34.130000	-1.594000	37.442000	36.447000
H	25.100000	-7.129000	29.932000	22.160000	40.311000	35.395000	-1.088000	36.922000	38.183000
H	25.671000	-3.968000	29.974000	23.861000	37.839000	35.368000	-1.743000	35.488000	35.741000
H	25.791000	-5.023000	32.087000	25.019000	39.819000	35.394000	-0.033000	36.823000	35.324000
H	27.125000	-6.100000	31.687000	24.413000	40.620000	36.790000	1.218000	36.410000	36.385000
H	29.167000	-2.837000	27.879000	22.697000	39.770000	41.490000	-2.559000	33.775000	29.233000
H	30.523000	-1.871000	27.176000	22.118000	40.164000	39.843000	-3.600000	32.440000	29.512000
H	30.393000	-2.260000	28.884000	23.796000	39.829000	40.077000	-2.022000	32.508000	30.384000
H	31.704000	-5.403000	26.331000	21.221000	36.740000	41.304000	-4.948000	35.275000	31.865000
H	30.804000	-4.138000	25.560000	20.335000	38.332000	41.058000	-5.176000	34.106000	30.449000
H	29.908000	-5.002000	26.758000	21.646000	38.144000	42.179000	-4.266000	35.607000	30.411000
H	32.054000	-3.377000	27.915000	22.292000	37.770000	39.411000	-3.732000	33.022000	31.929000
H	30.351000	-5.193000	28.394000	24.117000	37.649000	42.000000	-2.022000	35.186000	31.055000
H	32.026000	-5.460000	28.958000	23.480000	36.318000	40.919000	-2.345000	35.189000	32.742000
H	31.003000	1.393000	28.016000	25.618000	30.685000	38.746000	1.487000	36.412000	26.076000
H	32.667000	1.177000	28.073000	26.174000	30.975000	40.378000	0.482000	37.416000	27.135000
H	32.000000	2.735000	28.733000	27.198000	30.043000	39.285000	-0.065000	35.945000	26.612000
H	30.987000	3.258000	30.979000	28.300000	31.184000	37.296000	-0.307000	34.046000	26.750000
H	30.711000	1.786000	31.858000	28.127000	32.915000	37.097000	0.352000	33.131000	28.208000
H	29.777000	2.024000	30.414000	26.654000	31.974000	36.974000	1.433000	33.821000	26.796000
H	32.855000	1.525000	30.616000	27.814000	32.104000	39.765000	-0.168000	35.592000	28.528000
H	30.710000	-0.052000	29.509000	25.528000	33.262000	38.588000	2.922000	35.238000	28.262000
H	32.274000	-0.787000	29.612000	26.081000	33.271000	40.349000	1.855000	36.386000	29.057000
H	32.021000	-2.214000	39.339000	34.498000	39.023000	38.194000	5.256000	28.704000	35.321000

H	31.356000	-3.275000	38.145000	32.983000	39.767000	38.719000	4.262000	30.189000	34.876000
H	30.343000	-2.561000	39.279000	33.460000	39.794000	37.018000	5.585000	30.359000	35.963000
H	32.243000	0.407000	36.956000	33.120000	36.004000	38.643000	6.202000	29.247000	31.600000
H	33.038000	-1.023000	36.549000	32.548000	37.512000	39.443000	4.715000	29.422000	32.352000
H	33.410000	-0.247000	38.076000	34.285000	37.168000	39.199000	5.916000	28.211000	32.881000
H	30.794000	-0.319000	38.520000	33.756000	37.257000	36.680000	7.143000	29.911000	33.973000
H	29.288000	-0.988000	37.275000	31.643000	38.254000	35.963000	6.165000	31.927000	34.486000
H	30.244000	0.158000	36.388000	31.745000	36.545000	36.561000	6.680000	31.967000	32.797000
H	30.583000	10.035000	32.527000	35.108000	35.148000	36.689000	10.500000	36.002000	35.609000
H	31.264000	12.212000	31.029000	34.476000	33.907000	38.446000	9.488000	33.577000	37.016000
H	30.734000	14.268000	31.730000	36.769000	32.119000	39.178000	9.084000	31.526000	35.547000
H	29.797000	14.535000	34.435000	38.485000	32.172000	38.114000	9.632000	31.466000	33.078000
H	29.607000	12.503000	35.787000	39.169000	33.860000	36.287000	10.713000	33.759000	32.100000
H	24.521000	9.536000	39.036000	35.299000	36.795000	27.775000	13.564000	36.827000	27.315000
H	22.298000	9.482000	39.062000	34.755000	35.431000	25.906000	13.999000	36.390000	24.883000
H	21.094000	7.120000	38.373000	35.803000	33.245000	25.523000	13.022000	38.109000	23.432000
H	22.350000	5.285000	37.364000	37.618000	32.478000	27.315000	11.238000	39.778000	24.113000
H	25.019000	5.470000	37.769000	38.279000	34.042000	29.112000	10.815000	40.158000	26.576000
H	26.037000	12.313000	37.214000	40.899000	34.157000	34.348000	16.053000	37.601000	31.500000
H	24.781000	13.924000	38.310000	42.182000	31.916000	34.698000	18.199000	36.507000	30.412000
H	25.607000	15.119000	40.601000	42.174000	30.432000	32.583000	17.851000	35.193000	28.250000
H	27.039000	13.665000	41.664000	40.614000	30.969000	30.595000	15.399000	34.235000	27.730000
H	28.485000	11.799000	40.724000	39.157000	33.168000	30.743000	13.504000	35.187000	29.351000
H	30.046000	9.635000	42.687000	41.285000	40.181000	35.287000	16.026000	38.707000	32.961000
H	31.758000	10.455000	44.751000	43.612000	41.389000	35.513000	18.116000	39.481000	33.704000
H	33.832000	11.297000	44.294000	45.419000	41.051000	33.897000	18.283000	41.581000	35.510000
H	34.539000	11.515000	41.865000	45.023000	39.403000	31.941000	16.234000	42.986000	35.778000
H	33.405000	10.568000	40.009000	42.882000	38.752000	31.610000	14.043000	42.300000	34.351000
H	27.602000	3.866000	39.903000	36.864000	39.657000	27.974000	10.013000	42.400000	28.319000
H	27.667000	2.064000	41.924000	37.021000	41.442000	26.392000	9.453000	44.658000	27.810000
H	29.478000	2.132000	43.415000	38.960000	43.188000	26.649000	11.125000	46.401000	28.648000
H	30.266000	4.578000	43.965000	40.350000	43.245000	28.757000	12.805000	46.091000	30.629000
H	29.954000	6.514000	42.089000	39.900000	41.663000	30.616000	13.144000	43.696000	31.193000
H	34.107000	10.362000	36.649000	35.942000	39.760000	37.335000	8.104000	39.014000	35.593000
H	36.675000	10.600000	35.654000	36.278000	40.654000	39.578000	7.069000	38.970000	37.928000
H	37.987000	8.336000	35.827000	38.946000	41.329000	40.285000	9.237000	39.919000	39.899000
H	36.553000	6.116000	36.111000	40.520000	41.409000	38.673000	11.176000	39.780000	39.267000
H	34.108000	6.259000	36.165000	40.200000	39.944000	36.565000	12.222000	39.515000	37.030000
H	33.555000	4.900000	35.779000	35.190000	42.409000	33.469000	6.747000	41.214000	30.647000
H	34.501000	2.849000	34.482000	32.641000	43.446000	33.724000	4.783000	42.904000	31.289000
H	32.868000	0.811000	34.282000	31.101000	42.111000	35.208000	4.418000	43.037000	33.625000
H	30.481000	1.095000	35.306000	31.266000	39.704000	35.173000	6.216000	42.594000	35.208000
H	29.670000	3.150000	36.248000	33.144000	38.577000	33.987000	8.249000	41.247000	34.354000
H	28.888000	2.040000	33.837000	29.576000	37.751000	33.964000	7.930000	36.485000	32.988000
H	28.286000	2.739000	32.403000	30.297000	36.549000	34.969000	6.960000	35.505000	33.976000

H	27.939000	6.762000	31.552000	34.276000	33.869000	34.401000	6.655000	33.561000	28.802000
H	26.535000	5.913000	31.150000	32.672000	34.059000	35.200000	8.387000	33.968000	28.845000
H	28.008000	4.986000	31.379000	33.880000	35.450000	34.981000	7.277000	34.803000	27.788000
H	25.012000	4.045000	34.085000	30.791000	34.276000	32.268000	5.462000	37.095000	30.354000
H	24.629000	5.302000	33.000000	32.203000	33.375000	31.878000	4.534000	35.681000	30.181000
H	24.960000	5.696000	34.793000	31.760000	34.772000	30.813000	5.102000	36.450000	28.722000
H	27.837000	4.025000	35.172000	31.013000	37.082000	32.169000	8.008000	34.683000	32.272000
H	28.947000	4.329000	33.885000	32.038000	37.305000	33.791000	6.282000	34.289000	32.216000
H	25.864000	-0.017000	35.350000	27.828000	36.888000	32.321000	6.469000	38.946000	34.904000
H	27.184000	1.042000	36.108000	27.105000	35.246000	31.807000	6.663000	37.222000	35.253000
H	27.579000	-0.430000	35.217000	28.803000	35.648000	31.682000	5.008000	37.883000	35.359000
H	25.290000	2.181000	31.867000	29.014000	33.987000	35.662000	5.197000	39.485000	31.664000
H	25.190000	2.985000	33.475000	30.361000	33.805000	34.506000	5.225000	37.972000	30.942000
H	24.476000	1.405000	33.349000	28.860000	32.956000	34.194000	6.749000	38.577000	31.126000
H	58.448000	51.844000	55.773000	18.149000	35.829000	44.083000	30.148000	39.424000	33.318000
H	57.426000	53.317000	55.279000	16.592000	35.404000	44.682000	29.251000	41.016000	33.168000
H	57.020000	51.578000	54.840000	17.669000	34.146000	43.977000	29.065000	40.164000	34.693000
H	60.469000	53.134000	55.202000	17.188000	37.483000	42.150000	28.750000	38.410000	31.442000
H	59.479000	54.502000	55.050000	15.685000	37.214000	43.138000	27.942000	39.922000	31.515000
H	60.184000	53.926000	53.691000	15.743000	36.890000	41.357000	27.096000	38.407000	31.161000
H	58.347000	53.445000	53.161000	15.874000	34.620000	42.707000	27.187000	39.790000	33.863000
H	59.925000	52.377000	52.527000	16.762000	35.245000	40.743000	27.308000	37.315000	33.978000
H	59.921000	50.953000	53.630000	18.404000	35.497000	41.246000	29.075000	37.571000	33.565000
H	60.708000	46.242000	53.425000	13.737000	33.072000	43.446000	28.694000	33.772000	33.304000
H	59.750000	47.598000	53.034000	15.029000	31.873000	42.890000	28.958000	34.641000	34.837000
H	61.516000	47.721000	53.010000	13.351000	31.392000	42.877000	29.058000	32.902000	34.861000
H	62.474000	45.262000	50.556000	11.817000	32.975000	40.056000	32.626000	33.157000	33.226000
H	62.086000	44.803000	52.258000	11.590000	33.619000	41.718000	30.981000	32.716000	32.604000
H	63.172000	46.054000	51.734000	11.669000	31.958000	41.611000	31.613000	32.012000	34.067000
H	60.087000	45.741000	51.261000	14.082000	33.424000	40.986000	30.960000	34.906000	33.592000
H	61.775000	47.945000	50.984000	13.571000	30.604000	40.964000	31.259000	33.349000	35.865000
H	61.392000	47.427000	49.345000	13.619000	31.502000	39.460000	32.388000	34.705000	35.751000
H	60.282000	54.479000	46.400000	17.373000	31.005000	46.136000	24.764000	36.907000	40.728000
H	58.479000	54.540000	46.418000	17.417000	30.869000	44.504000	23.430000	35.898000	40.362000
H	59.344000	52.918000	46.598000	17.767000	32.446000	45.213000	23.102000	37.574000	40.778000
H	61.556000	53.278000	48.180000	19.383000	31.185000	47.587000	21.833000	38.167000	38.725000
H	60.549000	52.386000	48.994000	19.692000	32.632000	46.865000	22.643000	36.636000	38.124000
H	60.941000	53.707000	49.814000	20.993000	31.394000	46.767000	22.489000	37.958000	37.128000
H	59.715000	55.404000	48.244000	19.534000	29.962000	45.556000	24.194000	38.610000	39.382000
H	58.602000	54.553000	49.856000	20.004000	32.481000	43.940000	24.811000	35.947000	38.465000
H	57.351000	54.838000	48.635000	21.252000	31.401000	44.240000	24.939000	37.165000	37.171000
H	54.704000	53.441000	46.886000	22.226000	28.850000	42.237000	27.637000	41.293000	39.513000
H	53.973000	54.654000	47.925000	22.803000	30.541000	42.612000	28.413000	42.343000	40.724000
H	53.235000	54.449000	46.316000	23.736000	29.089000	43.209000	26.964000	42.964000	39.811000
H	52.605000	52.258000	45.529000	24.595000	27.608000	41.153000	24.684000	41.351000	40.851000

H	52.210000	50.784000	46.375000	24.569000	28.030000	39.390000	25.187000	39.666000	41.303000
H	53.903000	51.286000	46.076000	23.114000	27.452000	40.277000	25.621000	40.047000	39.771000
H	51.951000	52.881000	47.663000	24.764000	29.617000	40.875000	26.715000	41.645000	42.259000
H	53.132000	52.715000	49.363000	22.922000	31.254000	40.600000	28.573000	40.672000	41.769000
H	52.553000	51.185000	49.217000	23.223000	30.377000	38.988000	27.602000	39.529000	42.421000
H	53.685000	47.739000	54.785000	19.812000	35.324000	35.236000	31.581000	43.710000	38.796000
H	52.946000	49.351000	55.246000	21.157000	35.707000	36.279000	32.477000	44.183000	37.334000
H	54.089000	48.555000	56.339000	19.875000	36.902000	36.028000	33.399000	43.739000	38.744000
H	55.989000	50.732000	53.995000	19.090000	35.073000	39.393000	34.396000	41.255000	37.903000
H	55.613000	51.227000	55.600000	19.339000	36.695000	38.765000	33.693000	41.625000	36.363000
H	54.305000	51.330000	54.428000	20.728000	35.740000	38.915000	32.940000	40.375000	37.277000
H	55.577000	48.501000	54.739000	18.312000	35.066000	37.280000	32.179000	41.798000	39.082000
H	54.396000	48.205000	52.705000	19.254000	33.366000	36.280000	30.285000	42.401000	38.050000
H	53.573000	49.821000	52.673000	20.875000	33.477000	37.160000	30.657000	41.905000	36.438000
H	58.478000	44.869000	48.863000	14.099000	29.074000	38.304000	33.981000	34.620000	38.285000
H	58.058000	43.237000	48.764000	13.257000	28.188000	37.046000	35.756000	34.581000	38.469000
H	58.402000	44.077000	50.396000	13.414000	29.868000	36.856000	34.951000	35.468000	37.156000
H	55.644000	43.938000	47.535000	14.929000	26.707000	36.747000	34.744000	35.107000	41.084000
H	56.455000	45.565000	48.040000	16.258000	27.231000	37.979000	33.278000	35.625000	40.351000
H	54.693000	45.167000	48.391000	16.596000	26.858000	36.340000	34.449000	36.855000	40.976000
H	56.012000	43.542000	50.136000	14.947000	28.572000	35.393000	35.706000	37.046000	39.007000
H	55.144000	45.620000	50.153000	16.738000	29.099000	35.689000	33.975000	38.270000	39.352000
H	56.423000	45.496000	51.448000	15.917000	30.792000	35.920000	34.192000	37.920000	37.644000
H	61.161000	50.053000	43.518000	15.446000	25.776000	44.559000	28.173000	30.943000	41.045000
H	62.085000	48.574000	43.965000	13.838000	25.995000	43.713000	29.545000	30.097000	40.232000
H	62.524000	50.127000	44.597000	14.549000	27.288000	44.693000	28.367000	30.954000	39.256000
H	61.740000	47.271000	46.123000	14.541000	28.070000	41.137000	31.427000	33.020000	38.998000
H	60.491000	48.148000	47.176000	13.742000	28.545000	42.755000	30.015000	32.076000	38.208000
H	62.031000	49.026000	46.947000	13.790000	26.792000	42.096000	31.258000	31.248000	39.343000
H	60.050000	48.359000	44.946000	15.906000	26.128000	42.209000	30.294000	32.495000	40.902000
H	61.021000	50.726000	46.505000	16.104000	28.697000	43.614000	28.132000	32.586000	39.004000
H	59.625000	50.948000	45.472000	17.432000	27.750000	43.905000	27.665000	33.225000	40.568000
H	58.480000	40.290000	37.453000	11.540000	26.964000	34.300000	35.359000	40.638000	40.917000
H	60.294000	41.447000	36.408000	8.914000	27.706000	35.108000	35.235000	39.708000	38.436000
H	62.018000	43.032000	37.851000	7.822000	26.027000	36.628000	37.563000	39.056000	37.343000
H	61.785000	42.955000	40.323000	8.510000	23.726000	36.947000	39.480000	38.718000	38.872000
H	59.789000	41.492000	41.435000	10.873000	23.137000	35.719000	39.219000	39.209000	41.368000
H	54.043000	39.593000	46.357000	18.013000	26.738000	35.909000	33.407000	40.936000	45.818000
H	53.383000	40.272000	48.428000	18.791000	28.770000	35.177000	31.076000	41.570000	46.357000
H	53.277000	42.909000	48.971000	19.823000	29.251000	32.746000	30.384000	42.830000	48.748000
H	52.295000	44.247000	46.827000	20.778000	26.942000	31.722000	32.411000	43.288000	50.165000
H	53.388000	43.651000	44.621000	20.047000	24.803000	32.601000	34.637000	42.545000	49.669000
H	58.025000	40.112000	44.860000	16.921000	28.241000	32.023000	36.001000	45.842000	45.602000
H	60.119000	39.827000	46.217000	17.042000	30.856000	32.157000	34.004000	47.593000	44.793000
H	60.075000	37.258000	47.124000	15.100000	32.187000	33.528000	33.074000	47.283000	42.449000

H	58.118000	35.765000	46.256000	13.031000	30.851000	34.316000	33.417000	45.113000	41.248000
H	56.895000	36.793000	44.247000	13.088000	28.339000	34.011000	34.618000	43.298000	42.214000
H	54.198000	35.410000	43.641000	16.216000	26.636000	27.790000	42.368000	43.797000	44.653000
H	53.714000	32.919000	43.889000	15.680000	27.179000	25.621000	43.498000	45.584000	44.229000
H	54.791000	31.379000	42.234000	13.734000	26.144000	24.233000	42.504000	47.969000	44.536000
H	55.425000	32.427000	39.717000	12.159000	24.666000	25.384000	40.074000	48.106000	45.209000
H	55.312000	34.481000	39.241000	12.370000	24.038000	27.662000	38.670000	46.079000	45.571000
H	51.006000	40.791000	43.816000	19.352000	21.900000	31.489000	37.426000	42.550000	50.244000
H	48.611000	40.469000	45.080000	21.336000	20.931000	29.992000	38.228000	43.854000	52.096000
H	47.468000	38.203000	45.032000	21.151000	21.457000	27.357000	40.306000	43.370000	53.182000
H	48.504000	36.610000	43.902000	19.241000	22.812000	26.532000	42.151000	42.688000	51.555000
H	50.734000	36.770000	42.977000	17.249000	23.377000	27.862000	41.535000	41.141000	49.465000
H	52.928000	38.529000	36.431000	12.071000	21.596000	28.728000	42.117000	42.144000	42.826000
H	52.754000	36.993000	34.569000	10.136000	20.331000	27.764000	43.905000	41.737000	41.325000
H	55.119000	36.495000	33.290000	8.104000	20.098000	28.974000	44.743000	39.245000	40.738000
H	57.019000	36.699000	34.402000	7.862000	20.899000	31.357000	43.653000	37.450000	41.599000
H	57.358000	37.857000	36.800000	9.645000	22.168000	32.473000	41.204000	37.797000	42.751000
H	50.043000	42.153000	40.153000	17.551000	19.526000	31.399000	41.711000	38.032000	47.945000
H	48.162000	42.260000	39.111000	18.328000	17.674000	31.952000	42.772000	35.390000	48.645000
H	48.112000	41.839000	36.218000	16.759000	15.645000	32.628000	41.120000	33.424000	48.151000
H	50.286000	40.735000	35.163000	14.271000	16.213000	32.993000	39.106000	33.756000	47.164000
H	52.046000	40.420000	36.473000	13.693000	18.630000	33.120000	38.398000	36.165000	46.240000
H	55.019000	45.829000	44.955000	17.405000	25.072000	36.865000	32.861000	34.000000	44.155000
H	54.101000	47.046000	44.068000	19.082000	24.666000	36.991000	32.886000	35.007000	42.579000
H	52.975000	46.270000	41.311000	17.618000	20.609000	39.046000	36.942000	35.195000	46.203000
H	53.359000	45.865000	39.646000	18.159000	20.429000	37.366000	35.713000	33.878000	45.677000
H	53.856000	47.499000	40.298000	16.668000	19.742000	37.691000	35.045000	35.261000	46.624000
H	55.924000	47.179000	39.598000	15.040000	21.947000	39.799000	37.296000	35.965000	43.256000
H	57.284000	45.814000	39.909000	14.784000	23.507000	38.841000	36.179000	37.235000	42.900000
H	57.069000	47.211000	40.986000	16.254000	23.297000	39.643000	35.788000	35.502000	42.271000
H	56.161000	45.026000	43.471000	18.031000	22.840000	36.280000	33.272000	36.862000	43.725000
H	54.422000	44.532000	42.993000	18.487000	22.605000	38.052000	33.160000	36.239000	45.361000
H	58.114000	47.290000	46.050000	16.576000	25.662000	40.412000	28.719000	34.002000	42.534000
H	58.469000	46.520000	44.532000	17.915000	25.994000	41.492000	29.382000	33.584000	43.979000
H	57.331000	45.702000	45.732000	17.394000	24.299000	41.105000	30.133000	32.831000	42.630000
H	57.034000	49.582000	43.236000	19.986000	24.249000	40.881000	30.199000	36.878000	44.941000
H	55.310000	49.356000	43.380000	20.872000	24.545000	39.365000	30.499000	35.509000	45.981000
H	56.269000	48.386000	42.200000	19.675000	23.270000	39.424000	28.976000	35.682000	45.049000
H	32.660000	8.012000	48.480000	50.364000	19.641000	37.927000	43.404000	13.827000	44.664000
H	32.584000	8.907000	50.023000	49.948000	21.370000	37.444000	44.959000	14.379000	43.956000
H	34.086000	8.795000	49.096000	50.540000	20.212000	36.335000	44.937000	13.404000	45.331000
H	30.519000	9.098000	47.665000	48.405000	19.784000	39.377000	43.469000	12.122000	42.131000
H	31.062000	10.825000	47.440000	46.851000	19.816000	38.685000	45.228000	11.672000	41.851000
H	30.918000	10.133000	49.120000	47.715000	21.364000	38.574000	44.722000	13.485000	42.243000
H	33.056000	10.034000	47.223000	48.350000	18.721000	37.060000	44.897000	11.480000	44.190000

H	32.829000	11.130000	49.863000	47.454000	21.243000	36.491000	46.501000	13.800000	43.473000
H	34.433000	11.136000	49.059000	47.923000	20.196000	35.106000	47.197000	12.935000	44.806000
H	33.818000	16.937000	44.124000	43.049000	15.407000	32.990000	48.563000	11.507000	36.848000
H	32.905000	16.181000	42.816000	42.952000	14.131000	34.324000	48.306000	13.065000	36.056000
H	34.580000	16.964000	42.602000	43.476000	13.865000	32.653000	47.206000	11.707000	35.579000
H	35.694000	15.091000	41.528000	45.209000	12.462000	33.864000	45.544000	13.099000	35.100000
H	34.299000	14.207000	41.393000	44.765000	12.966000	35.416000	46.207000	14.392000	35.933000
H	35.594000	13.388000	42.141000	46.273000	13.447000	34.843000	44.761000	13.616000	36.592000
H	35.703000	15.391000	43.740000	45.685000	14.919000	33.522000	45.847000	11.674000	36.967000
H	33.424000	13.804000	43.593000	44.465000	15.070000	36.074000	47.592000	13.889000	38.158000
H	34.531000	12.995000	44.459000	45.992000	15.774000	35.579000	45.986000	13.654000	38.936000
H	37.273000	14.856000	51.099000	47.605000	20.413000	32.306000	45.008000	8.474000	43.687000
H	37.767000	16.528000	50.685000	47.471000	19.589000	30.840000	43.927000	7.334000	42.807000
H	36.528000	15.868000	49.720000	46.020000	19.627000	31.961000	45.423000	8.125000	42.073000
H	37.483000	17.344000	52.999000	47.499000	21.892000	29.759000	44.937000	5.110000	44.269000
H	36.927000	15.738000	53.515000	47.820000	22.586000	31.359000	45.506000	6.411000	45.294000
H	35.981000	17.078000	53.753000	46.642000	23.321000	30.229000	46.781000	5.345000	44.477000
H	35.590000	17.487000	51.166000	45.146000	21.182000	30.708000	46.397000	6.239000	42.435000
H	34.825000	14.822000	52.056000	46.208000	22.199000	33.251000	46.691000	8.097000	44.262000
H	33.754000	16.173000	52.311000	44.765000	22.930000	32.394000	48.207000	7.237000	44.092000
H	26.405000	17.191000	50.914000	38.792000	22.967000	37.535000	55.361000	8.946000	42.734000
H	27.785000	16.813000	50.034000	39.843000	21.996000	36.416000	53.996000	9.693000	41.790000
H	28.022000	18.163000	51.112000	38.507000	23.057000	35.731000	54.176000	7.818000	41.984000
H	27.796000	14.423000	52.872000	41.592000	24.105000	38.831000	53.329000	9.493000	45.641000
H	27.434000	14.281000	51.065000	41.300000	22.446000	38.236000	53.240000	10.639000	44.265000
H	26.595000	15.307000	52.227000	39.984000	23.422000	38.894000	54.786000	10.145000	44.645000
H	27.998000	16.886000	52.841000	40.086000	24.815000	36.950000	54.061000	7.737000	44.237000
H	29.910000	17.302000	51.869000	41.041000	23.729000	35.044000	52.243000	7.276000	43.073000
H	30.278000	15.820000	52.684000	42.359000	24.130000	36.188000	51.569000	8.129000	44.402000
H	25.188000	11.449000	48.239000	45.248000	20.259000	42.484000	53.187000	15.633000	45.607000
H	25.930000	10.882000	49.817000	43.522000	20.209000	42.447000	53.983000	15.394000	43.995000
H	26.670000	10.379000	48.360000	44.249000	21.691000	42.386000	54.072000	14.293000	45.209000
H	27.085000	14.245000	48.564000	45.117000	18.726000	39.491000	50.375000	14.916000	43.275000
H	26.336000	13.551000	49.977000	43.859000	18.392000	40.659000	51.836000	15.887000	42.797000
H	25.404000	13.540000	48.534000	45.534000	18.381000	41.231000	51.168000	16.115000	44.473000
H	27.185000	12.154000	47.396000	45.506000	21.032000	40.331000	51.730000	13.982000	45.148000
H	28.278000	12.429000	49.918000	42.710000	20.216000	40.003000	52.894000	13.727000	42.629000
H	29.025000	11.069000	49.083000	43.009000	21.825000	39.492000	52.770000	12.354000	43.778000
H	29.668000	14.370000	42.345000	42.208000	14.156000	37.614000	49.983000	14.278000	36.556000
H	30.349000	12.746000	42.079000	43.342000	14.347000	38.855000	49.873000	15.852000	37.330000
H	30.845000	13.718000	43.586000	43.733000	15.179000	37.412000	49.249000	14.387000	38.224000
H	29.399000	11.865000	45.129000	43.288000	17.037000	39.351000	51.623000	15.028000	39.822000
H	28.707000	11.540000	43.567000	43.130000	15.931000	40.685000	51.967000	16.485000	38.753000
H	27.745000	12.325000	44.805000	41.834000	17.036000	40.432000	53.004000	15.002000	38.784000
H	27.922000	13.728000	43.220000	41.131000	14.946000	39.390000	52.105000	14.905000	36.867000

H	29.097000	15.685000	43.894000	40.416000	15.869000	37.446000	51.155000	12.747000	37.272000
H	28.128000	14.981000	45.246000	40.265000	17.280000	38.412000	52.606000	12.705000	38.284000
H	36.320000	16.730000	46.345000	45.527000	17.476000	31.781000	47.355000	6.397000	40.278000
H	37.967000	17.236000	47.172000	46.106000	17.102000	30.221000	46.482000	4.919000	39.600000
H	36.988000	18.238000	46.001000	45.086000	16.025000	31.077000	45.566000	6.389000	40.208000
H	36.362000	20.024000	49.091000	42.821000	17.662000	28.570000	46.005000	6.709000	36.303000
H	37.116000	20.307000	47.580000	43.366000	16.141000	29.377000	44.862000	6.268000	37.764000
H	37.841000	19.118000	48.620000	44.378000	16.874000	28.190000	45.996000	5.073000	37.130000
H	36.249000	17.611000	48.768000	44.373000	18.817000	30.212000	47.845000	6.629000	38.032000
H	34.615000	18.734000	48.400000	42.654000	18.703000	30.468000	46.846000	8.747000	37.536000
H	34.670000	19.015000	46.750000	42.287000	17.073000	30.999000	45.781000	8.433000	38.934000
H	37.411000	21.981000	57.259000	38.335000	26.096000	30.656000	54.911000	6.887000	33.682000
H	39.377000	20.321000	57.081000	39.843000	28.032000	30.967000	52.738000	7.631000	32.710000
H	39.861000	19.050000	55.323000	39.200000	30.489000	30.241000	52.433000	8.903000	30.297000
H	38.177000	19.018000	53.321000	36.878000	30.868000	29.077000	54.405000	9.151000	29.270000
H	35.969000	20.053000	53.353000	35.278000	29.247000	29.042000	56.948000	9.338000	30.851000
H	31.658000	27.402000	51.135000	33.398000	19.962000	27.638000	63.221000	10.716000	36.099000
H	31.928000	29.102000	49.317000	34.405000	17.561000	26.160000	64.278000	12.634000	37.337000
H	33.866000	28.969000	48.070000	36.559000	17.931000	24.890000	62.831000	13.638000	39.342000
H	35.577000	27.096000	48.182000	38.018000	20.265000	25.060000	60.487000	12.850000	39.829000
H	35.444000	25.328000	49.943000	37.090000	21.918000	26.864000	59.399000	10.964000	38.304000
H	35.330000	28.274000	56.546000	35.320000	23.645000	25.077000	62.194000	9.875000	30.444000
H	37.141000	29.995000	56.528000	36.328000	24.237000	22.834000	62.387000	12.708000	31.257000
H	39.713000	29.330000	55.795000	35.505000	26.643000	21.602000	61.676000	12.913000	28.804000
H	40.094000	27.033000	55.221000	33.894000	27.983000	23.052000	59.308000	11.852000	28.799000
H	38.286000	25.228000	55.040000	33.731000	27.457000	25.642000	58.840000	10.080000	30.798000
H	34.317000	27.562000	57.491000	30.917000	25.922000	25.736000	63.529000	4.387000	33.316000
H	34.650000	29.438000	59.224000	28.668000	26.135000	24.531000	65.878000	4.060000	32.678000
H	32.824000	29.346000	61.203000	26.826000	26.778000	25.356000	66.996000	5.773000	30.937000
H	31.030000	27.692000	61.137000	26.534000	26.938000	28.131000	65.405000	7.555000	29.793000
H	30.797000	26.087000	59.011000	28.588000	26.076000	29.475000	63.204000	7.689000	30.781000
H	27.609000	25.330000	53.899000	29.003000	21.561000	31.004000	62.471000	4.867000	38.652000
H	26.336000	27.018000	52.590000	27.160000	20.605000	30.003000	64.162000	5.098000	40.546000
H	26.721000	29.722000	53.215000	27.201000	18.922000	27.837000	66.284000	6.263000	40.249000
H	28.737000	30.512000	53.791000	29.703000	19.104000	26.549000	66.718000	7.575000	37.963000
H	30.628000	28.604000	54.435000	31.821000	20.002000	28.012000	64.914000	7.456000	36.201000
H	31.705000	23.359000	59.636000	30.605000	27.956000	30.025000	59.753000	1.963000	33.811000
H	32.167000	22.272000	62.241000	29.120000	29.803000	30.583000	58.686000	-0.182000	32.819000
H	32.695000	19.812000	62.119000	29.617000	31.112000	32.820000	56.943000	0.000000	31.031000
H	33.525000	18.643000	59.985000	31.874000	30.459000	34.197000	55.672000	2.397000	30.748000
H	33.167000	19.460000	57.900000	33.014000	28.334000	33.748000	56.663000	4.169000	31.989000
H	27.946000	23.663000	53.757000	33.624000	20.873000	33.848000	59.976000	3.085000	38.017000
H	25.897000	22.341000	53.157000	32.902000	20.195000	36.194000	58.474000	0.969000	38.765000
H	25.628000	19.839000	53.246000	31.590000	21.497000	37.509000	56.124000	0.900000	38.461000
H	27.843000	18.371000	53.995000	30.535000	23.779000	36.386000	54.808000	3.369000	38.004000

H	29.686000	19.873000	54.686000	31.374000	24.597000	34.400000	56.572000	5.441000	37.704000
H	28.238000	20.680000	50.100000	35.704000	20.368000	34.973000	52.778000	7.132000	34.984000
H	28.858000	19.002000	49.919000	37.239000	21.160000	35.421000	52.886000	8.797000	35.495000
H	32.017000	18.791000	51.992000	38.311000	22.937000	34.235000	54.243000	11.142000	36.829000
H	33.424000	18.834000	51.116000	39.169000	23.700000	32.791000	55.166000	11.019000	38.387000
H	31.960000	18.126000	50.418000	39.882000	22.158000	33.456000	53.479000	10.303000	38.298000
H	33.242000	21.896000	48.996000	39.446000	21.198000	30.214000	55.113000	8.174000	39.899000
H	32.164000	20.916000	47.960000	39.020000	19.981000	31.326000	55.125000	6.742000	38.693000
H	33.601000	20.168000	48.589000	40.025000	21.404000	31.866000	53.733000	7.606000	38.969000
H	29.811000	21.783000	50.400000	35.804000	20.971000	32.811000	54.600000	6.867000	36.600000
H	29.908000	20.422000	51.649000	36.197000	22.415000	33.897000	55.105000	8.127000	35.328000
H	27.024000	19.876000	46.338000	38.623000	17.912000	36.442000	49.483000	6.966000	37.232000
H	26.950000	21.468000	46.981000	36.746000	17.728000	36.288000	49.975000	6.167000	35.625000
H	26.500000	20.132000	48.013000	37.581000	19.286000	36.769000	49.796000	7.905000	35.668000
H	29.453000	22.338000	47.511000	37.198000	18.504000	32.126000	51.265000	5.286000	38.651000
H	29.458000	21.634000	45.972000	35.822000	17.766000	33.070000	53.025000	5.849000	38.480000
H	30.969000	21.619000	47.110000	37.455000	17.084000	32.927000	52.500000	4.867000	37.324000
H	51.055000	58.147000	43.124000	55.464000	41.539000	44.634000	11.965000	59.965000	31.099000
H	49.546000	57.109000	43.405000	54.416000	42.897000	45.136000	13.181000	60.217000	32.301000
H	50.439000	57.134000	41.778000	53.867000	41.923000	43.883000	12.886000	61.528000	31.057000
H	47.958000	60.112000	41.511000	56.486000	43.673000	41.722000	16.045000	59.206000	30.152000
H	49.299000	59.117000	40.905000	55.164000	42.371000	41.552000	15.656000	59.299000	31.947000
H	49.580000	60.422000	42.124000	56.679000	41.946000	42.382000	15.944000	60.821000	31.081000
H	48.468000	58.767000	43.639000	56.230000	43.804000	44.218000	13.944000	59.994000	29.470000
H	46.791000	58.030000	42.140000	55.416000	45.199000	42.457000	12.875000	58.226000	29.895000
H	47.953000	57.286000	41.226000	53.834000	44.106000	42.453000	14.647000	57.792000	29.778000
H	53.861000	54.227000	43.059000	60.709000	45.641000	45.107000	7.180000	61.581000	33.877000
H	53.133000	52.597000	42.871000	60.171000	46.882000	46.334000	7.545000	60.331000	32.741000
H	53.292000	53.237000	44.453000	60.811000	47.292000	44.696000	8.647000	61.859000	33.061000
H	52.062000	55.335000	45.491000	58.659000	46.529000	42.754000	10.224000	61.933000	34.802000
H	50.806000	55.788000	44.202000	57.517000	45.871000	44.062000	10.743000	60.500000	35.490000
H	52.527000	56.352000	44.070000	59.125000	44.928000	43.581000	9.261000	61.298000	36.005000
H	51.456000	54.630000	42.574000	58.229000	45.627000	45.615000	8.465000	59.235000	34.639000
H	50.859000	52.599000	43.101000	58.404000	47.543000	46.606000	8.977000	58.661000	32.712000
H	51.008000	52.909000	44.861000	58.242000	48.516000	45.353000	10.266000	59.895000	32.437000
H	44.216000	52.332000	39.301000	51.149000	40.442000	47.584000	19.360000	57.548000	34.767000
H	42.441000	52.489000	39.037000	52.616000	39.476000	47.931000	19.617000	56.351000	36.122000
H	43.141000	53.141000	40.428000	52.265000	39.986000	46.225000	18.345000	57.578000	36.142000
H	44.182000	55.697000	37.302000	52.907000	42.968000	49.327000	17.682000	54.526000	33.335000
H	43.274000	54.311000	37.022000	53.369000	41.266000	49.589000	19.095000	54.390000	34.491000
H	44.938000	54.151000	37.150000	51.570000	41.834000	49.568000	18.965000	55.604000	33.259000
H	43.290000	55.069000	39.310000	53.711000	41.559000	47.510000	17.441000	55.548000	35.834000
H	45.672000	53.787000	39.171000	52.070000	42.648000	46.001000	16.601000	57.674000	35.264000
H	45.699000	55.408000	39.594000	50.943000	42.884000	47.320000	16.854000	57.361000	33.519000
H	43.104000	52.416000	46.372000	49.485000	49.394000	48.421000	14.622000	52.359000	31.983000

H	41.521000	51.626000	45.858000	48.603000	48.183000	49.319000	13.959000	50.794000	32.734000
H	41.695000	52.179000	47.478000	47.825000	49.644000	48.715000	15.384000	50.631000	31.707000
H	41.260000	55.694000	46.216000	47.717000	48.161000	45.267000	17.382000	52.456000	34.264000
H	42.865000	54.882000	46.844000	49.200000	48.996000	45.822000	16.754000	53.133000	32.707000
H	41.233000	54.899000	47.709000	47.731000	49.871000	45.445000	17.689000	51.546000	32.755000
H	40.394000	53.717000	45.959000	47.080000	47.909000	47.341000	15.863000	50.562000	34.021000
H	41.634000	52.702000	44.002000	48.841000	46.655000	47.992000	14.217000	51.427000	34.565000
H	41.693000	54.538000	43.705000	48.426000	46.211000	46.294000	15.330000	52.623000	35.448000
H	47.394000	57.873000	46.162000	53.862000	48.454000	41.562000	12.508000	55.560000	28.833000
H	47.417000	58.134000	47.845000	53.416000	50.228000	41.659000	10.809000	55.051000	28.241000
H	46.979000	59.550000	46.602000	53.173000	49.326000	40.071000	12.344000	54.873000	27.288000
H	45.125000	60.081000	47.869000	51.148000	49.811000	39.454000	12.471000	52.976000	26.765000
H	44.954000	58.839000	48.875000	51.066000	50.658000	40.979000	10.939000	52.617000	27.861000
H	43.626000	59.322000	48.060000	49.738000	49.371000	40.533000	12.384000	51.466000	27.877000
H	45.153000	58.530000	45.850000	51.782000	47.934000	40.842000	13.528000	53.541000	28.590000
H	45.206000	56.212000	47.751000	51.337000	49.860000	42.999000	10.998000	53.175000	30.096000
H	43.964000	56.664000	46.679000	50.206000	48.552000	42.786000	12.492000	52.333000	30.587000
H	47.814000	49.410000	46.744000	56.023000	50.017000	44.280000	8.945000	56.627000	31.715000
H	46.971000	49.556000	48.448000	56.981000	50.955000	45.134000	7.382000	57.077000	32.354000
H	48.505000	48.787000	48.261000	57.331000	50.790000	43.423000	7.641000	56.880000	30.592000
H	49.607000	50.790000	46.380000	54.355000	50.864000	42.796000	8.967000	54.080000	30.335000
H	50.724000	50.083000	47.485000	55.146000	52.315000	42.233000	7.546000	54.509000	29.612000
H	50.274000	51.837000	47.697000	53.654000	52.471000	43.016000	7.633000	53.280000	30.839000
H	48.908000	50.730000	49.263000	55.559000	52.960000	44.449000	6.323000	54.960000	31.881000
H	47.212000	51.955000	48.712000	55.419000	51.691000	46.361000	7.834000	55.368000	33.836000
H	48.318000	53.176000	48.207000	53.748000	51.996000	45.910000	8.061000	53.645000	33.301000
H	46.733000	48.125000	42.721000	58.144000	46.134000	49.147000	10.179000	59.645000	37.552000
H	45.374000	49.018000	42.385000	57.090000	47.617000	48.863000	10.444000	57.765000	37.369000
H	45.633000	47.598000	41.475000	58.492000	47.660000	49.884000	9.481000	58.433000	38.748000
H	45.364000	48.761000	39.283000	56.814000	48.227000	51.988000	10.897000	57.382000	40.333000
H	46.659000	49.958000	38.825000	55.409000	47.181000	52.169000	12.693000	57.278000	39.959000
H	45.332000	50.332000	39.966000	55.521000	48.111000	50.727000	11.625000	56.483000	38.930000
H	47.828000	48.527000	40.478000	57.280000	45.874000	51.282000	11.981000	59.237000	39.309000
H	48.330000	49.659000	42.153000	56.152000	44.985000	49.358000	12.677000	59.410000	36.862000
H	48.628000	50.521000	40.789000	54.885000	45.114000	50.461000	13.448000	58.144000	37.962000
H	50.315000	48.212000	50.662000	45.154000	52.829000	52.169000	12.601000	44.753000	41.333000
H	52.314000	49.708000	51.549000	46.155000	55.241000	50.948000	11.725000	42.806000	39.806000
H	51.788000	51.905000	52.802000	47.756000	55.079000	49.241000	12.188000	42.904000	37.527000
H	49.221000	52.247000	53.295000	48.630000	52.879000	48.448000	13.328000	45.478000	36.488000
H	47.620000	50.417000	53.221000	47.627000	50.742000	49.638000	14.084000	47.110000	38.119000
H	41.788000	44.738000	48.896000	50.084000	45.399000	54.881000	15.404000	52.103000	38.699000
H	39.370000	45.595000	47.590000	52.143000	43.976000	54.045000	15.288000	54.605000	37.632000
H	38.738000	47.901000	48.517000	52.022000	43.091000	51.483000	14.613000	56.780000	38.931000
H	39.908000	49.236000	49.432000	50.376000	43.465000	50.297000	13.505000	56.232000	41.171000
H	41.832000	48.521000	51.093000	48.163000	45.047000	50.975000	13.745000	54.276000	42.275000

H	45.737000	48.678000	55.572000	45.230000	48.932000	49.053000	17.525000	50.667000	38.523000
H	45.279000	51.567000	56.426000	44.584000	48.258000	46.704000	19.176000	50.593000	37.087000
H	43.123000	52.571000	55.479000	44.096000	45.935000	46.144000	21.126000	48.780000	37.056000
H	41.864000	51.162000	53.693000	43.189000	44.402000	48.367000	20.575000	46.821000	38.346000
H	42.781000	49.340000	52.731000	44.012000	44.992000	50.386000	18.755000	46.901000	40.134000
H	43.882000	45.260000	56.362000	41.936000	46.048000	56.421000	18.635000	45.992000	45.600000
H	43.970000	44.874000	58.959000	39.509000	45.510000	56.995000	20.701000	45.403000	46.563000
H	46.053000	44.086000	60.162000	37.740000	46.726000	55.211000	22.678000	47.440000	46.505000
H	47.819000	43.143000	58.773000	38.606000	48.497000	53.518000	22.106000	49.401000	44.964000
H	48.135000	43.958000	56.352000	40.798000	48.792000	53.207000	19.995000	49.939000	44.133000
H	42.332000	42.853000	53.630000	45.121000	43.576000	55.889000	15.823000	50.328000	47.683000
H	40.491000	40.847000	53.606000	45.782000	41.778000	57.244000	17.012000	51.811000	49.644000
H	40.848000	38.888000	52.167000	46.448000	42.139000	59.644000	18.198000	53.712000	48.795000
H	42.569000	39.179000	50.384000	47.248000	44.306000	60.178000	18.357000	54.575000	46.745000
H	44.410000	40.856000	50.237000	46.394000	46.309000	59.164000	17.150000	53.390000	44.763000
H	50.574000	42.389000	52.027000	44.837000	53.385000	55.451000	12.372000	44.526000	44.266000
H	52.088000	41.101000	53.432000	43.225000	55.458000	55.346000	12.380000	42.176000	44.141000
H	53.563000	42.583000	55.109000	41.061000	55.184000	55.815000	14.653000	40.735000	44.648000
H	52.621000	44.740000	55.733000	39.975000	52.501000	55.385000	16.558000	42.229000	45.103000
H	50.674000	45.734000	54.695000	41.457000	50.687000	55.311000	16.766000	44.652000	45.046000
H	45.915000	41.460000	49.753000	49.059000	49.415000	58.793000	10.538000	50.857000	45.297000
H	46.122000	39.238000	48.601000	48.904000	50.344000	61.239000	8.956000	51.046000	47.441000
H	48.238000	38.756000	47.277000	46.807000	51.411000	62.051000	9.303000	49.798000	49.218000
H	49.746000	40.883000	46.746000	44.729000	51.735000	60.573000	10.600000	47.479000	49.047000
H	49.644000	42.920000	48.461000	44.856000	51.151000	58.280000	12.233000	47.372000	47.042000
H	42.983000	46.941000	44.460000	53.646000	48.680000	52.529000	11.465000	50.902000	36.989000
H	44.717000	47.480000	44.149000	53.166000	50.503000	52.302000	12.077000	51.275000	38.544000
H	46.598000	43.927000	46.673000	51.129000	49.885000	56.972000	8.165000	50.022000	41.739000
H	47.643000	44.971000	47.551000	49.550000	50.224000	56.184000	7.686000	49.363000	40.145000
H	48.114000	44.529000	46.033000	50.489000	51.600000	57.055000	8.068000	50.932000	40.197000
H	48.259000	47.869000	45.993000	50.713000	52.959000	54.649000	11.375000	47.341000	40.190000
H	46.740000	48.600000	45.339000	51.859000	52.295000	53.258000	9.877000	47.063000	39.184000
H	47.601000	47.263000	44.467000	52.478000	52.790000	54.944000	9.854000	46.922000	41.007000
H	43.977000	46.111000	46.487000	51.922000	48.515000	54.414000	9.900000	49.536000	37.751000
H	44.543000	45.267000	45.177000	53.161000	49.583000	54.825000	11.410000	48.923000	38.461000
H	43.336000	48.376000	42.725000	51.754000	46.973000	51.511000	8.757000	51.570000	36.222000
H	43.468000	50.099000	42.933000	50.822000	47.494000	50.017000	8.796000	53.274000	35.902000
H	41.917000	49.415000	43.199000	50.058000	47.681000	51.750000	7.881000	52.665000	37.360000
H	42.237000	47.525000	46.373000	50.681000	51.019000	50.020000	8.807000	53.866000	39.512000
H	41.546000	49.057000	46.773000	50.611000	51.250000	51.662000	10.535000	53.836000	39.945000
H	43.014000	48.563000	47.645000	49.436000	50.164000	50.957000	9.409000	52.352000	40.121000

Table S6. Atomic coordinates of systems consisted of four molecules of Ph_7T_8-T_8iBu_7 compound for clusters 4-6.

Atomic coordinates of systems consisted of four molecules of Ph_7T_8-T_8iBu_7 compound									
	Cluster **4**			Cluster **5**			Cluster **6**		
Atom	x	y	z	x	y	z	x	y	z
Si	40.169000	37.325000	33.131000	61.27400	23.11600	34.03700	56.457000	38.185000	34.459000
Si	41.558000	39.727000	29.319000	59.55700	21.70200	36.99100	55.556000	38.508000	38.456000
Si	40.505000	42.179000	30.493000	56.83600	21.93000	35.67300	52.924000	36.970000	39.145000
Si	40.977000	45.093000	30.302000	53.94500	22.09600	36.03100	52.706000	36.853000	42.173000
Si	38.004000	45.511000	30.255000	53.89800	23.78200	33.57700	49.808000	37.418000	42.046000
Si	38.038000	45.538000	33.335000	53.44900	21.51800	31.78600	49.426000	34.450000	41.863000
Si	41.090000	44.995000	33.293000	53.25600	19.78500	34.22400	52.437000	33.818000	41.936000
Si	37.448000	42.511000	30.541000	56.91800	23.52800	33.28800	49.999000	37.436000	39.038000
Si	37.447000	42.664000	33.575000	56.32300	21.16100	31.67700	49.651000	34.421000	38.906000
Si	40.427000	42.118000	33.566000	56.28700	19.49900	34.09500	52.686000	34.033000	38.869000
Si	27.976000	39.480000	37.968000	31.70400	24.94400	48.56100	26.164000	45.297000	44.340000
Si	25.612000	35.878000	38.066000	35.14000	24.87000	45.27600	25.760000	40.603000	43.561000
Si	26.520000	33.089000	38.538000	34.90200	26.30800	42.63000	27.538000	38.329000	44.439000
Si	25.930000	30.213000	38.165000	34.24900	25.64100	39.76000	29.412000	36.108000	43.685000
Si	28.820000	29.699000	37.868000	32.37500	28.11900	39.66400	30.761000	36.372000	46.269000
Si	29.001000	29.242000	40.974000	34.65800	29.69800	38.83100	28.994000	34.318000	47.427000
Si	26.001000	29.847000	41.107000	36.52400	27.24700	38.73500	27.533000	34.165000	44.761000
Si	29.520000	32.495000	38.395000	33.11400	28.70300	42.54200	28.987000	38.598000	47.060000
Si	29.561000	32.119000	41.311000	35.44500	30.43100	41.60700	27.226000	36.553000	48.343000
Si	26.557000	32.777000	41.533000	37.29100	27.94900	41.58300	25.761000	36.289000	45.704000
Si	49.336000	19.453000	36.999000	49.73800	4.12000	49.61400	55.809000	15.493000	37.536000
Si	48.183000	16.422000	39.197000	46.56800	6.53100	49.43700	54.641000	16.678000	41.298000
Si	51.052000	16.799000	40.192000	45.63000	6.60100	52.25000	54.796000	19.732000	41.294000
Si	52.231000	19.428000	41.368000	43.31200	5.30400	53.62800	55.971000	20.364000	43.884000
Si	53.166000	18.073000	43.748000	44.91700	5.06300	56.24900	56.923000	22.891000	42.816000
Si	55.727000	17.583000	42.347000	44.11300	7.72000	57.18300	54.388000	24.440000	43.385000
Si	54.754000	18.848000	39.916000	42.36000	8.00000	54.62200	53.406000	21.793000	44.574000
Si	51.995000	15.454000	42.825000	47.18800	6.48800	54.81500	55.854000	22.293000	40.064000
Si	54.615000	14.962000	41.622000	46.33400	9.15900	55.71800	53.242000	23.726000	40.740000
Si	53.772000	16.147000	39.048000	44.70900	9.31700	53.34100	52.193000	21.101000	41.899000
Si	55.953000	57.336000	51.487000	15.74000	43.58700	43.75200	3.840000	41.404000	51.517000
Si	55.612000	55.108000	48.561000	12.53700	44.52000	41.96300	3.718000	44.243000	48.860000
Si	55.387000	52.803000	46.690000	12.15400	43.91300	39.12000	2.167000	46.465000	47.523000
Si	52.738000	53.259000	45.330000	10.57200	41.61900	37.94100	3.815000	48.281000	45.875000
Si	54.011000	52.392000	42.748000	8.57500	43.48400	36.87500	1.588000	48.704000	43.930000
Si	53.414000	49.585000	43.461000	10.06400	43.66500	34.30000	0.820000	51.084000	45.677000
Si	52.169000	50.348000	46.066000	12.06300	41.62800	35.38800	2.930000	50.485000	47.557000
Si	56.655000	52.005000	44.090000	10.20900	45.92900	37.94100	-0.181000	47.050000	45.604000
Si	56.059000	49.233000	44.861000	11.71500	45.85900	35.35000	-1.025000	49.381000	47.231000
Si	54.852000	49.954000	47.466000	13.66300	43.96900	36.54900	1.233000	48.773000	49.235000

Si	42.867000	36.801000	34.443000	60.81600	25.95000	35.08900	59.415000	38.724000	34.729000
Si	42.583000	36.986000	37.396000	63.61400	27.01800	34.56000	60.757000	36.126000	35.323000
Si	44.605000	34.735000	37.575000	62.66500	29.65500	33.75300	63.028000	36.672000	33.575000
Si	46.801000	36.744000	37.935000	62.76400	30.42400	36.67800	64.309000	37.941000	35.900000
Si	44.795000	39.094000	37.709000	63.80500	27.81700	37.51500	62.048000	37.261000	37.634000
Si	45.068000	34.823000	34.691000	59.77900	28.61300	34.11000	61.752000	39.270000	32.825000
Si	47.212000	36.845000	35.020000	59.82400	29.45200	37.00900	63.174000	40.541000	35.200000
Si	45.075000	38.862000	34.766000	60.99400	27.00600	37.89300	60.941000	39.939000	37.049000
Si	27.022000	41.790000	36.397000	29.60100	26.90200	47.40500	28.649000	46.000000	42.786000
Si	28.517000	42.737000	33.982000	26.80800	26.24000	48.27200	31.064000	45.545000	44.514000
Si	25.912000	42.323000	32.375000	26.11700	29.07900	48.38300	32.615000	45.299000	41.935000
Si	25.144000	45.003000	32.963000	25.23300	29.06800	45.42400	33.415000	48.090000	41.732000
Si	27.550000	45.465000	34.569000	26.05700	26.20600	45.26900	31.873000	48.394000	44.445000
Si	24.551000	41.328000	34.874000	28.99900	29.86800	47.75500	30.226000	45.799000	40.206000
Si	23.610000	44.026000	35.428000	28.09700	30.12100	44.72400	30.902000	48.681000	40.301000
Si	26.045000	44.540000	36.990000	28.73100	27.03900	44.55900	29.276000	48.974000	42.894000
Si	48.073000	20.964000	34.771000	51.41000	2.97900	47.34200	56.721000	12.677000	38.224000
Si	48.641000	23.463000	33.245000	51.83700	5.37800	45.50200	55.005000	10.215000	38.239000
Si	47.509000	22.514000	30.547000	54.36100	4.26800	44.76000	57.364000	8.542000	37.478000
Si	44.653000	23.208000	31.549000	53.09500	2.59800	42.54000	57.981000	8.069000	40.316000
Si	45.918000	24.129000	34.238000	50.64300	3.69600	43.31300	55.752000	9.680000	41.024000
Si	47.097000	19.997000	32.126000	53.94700	1.86800	46.47300	59.037000	11.030000	37.450000
Si	44.204000	20.524000	32.823000	52.74300	0.22100	44.28600	59.698000	10.701000	40.397000
Si	45.088000	21.546000	35.468000	50.16200	1.44000	45.18100	57.216000	12.275000	41.171000
Si	53.198000	58.470000	50.841000	18.90200	43.52800	44.08600	6.449000	40.533000	49.977000
Si	51.685000	59.729000	48.527000	19.62900	44.03600	46.95400	7.272000	38.292000	48.113000
Si	49.454000	57.879000	48.947000	22.06400	42.30300	46.49900	9.561000	40.053000	47.020000
Si	48.271000	59.804000	50.996000	23.51400	44.75800	45.81100	11.333000	38.756000	49.110000
Si	50.580000	61.577000	50.653000	21.07200	46.59900	46.10200	9.064000	37.013000	50.032000
Si	50.871000	56.569000	51.142000	21.39300	41.92300	43.59900	8.638000	42.244000	48.989000
Si	49.801000	58.410000	53.206000	22.91300	44.37900	42.96800	10.580000	40.994000	50.969000
Si	52.085000	60.403000	52.900000	20.46500	46.10400	43.21900	8.322000	39.261000	51.920000
O	41.779000	36.955000	33.214000	60.42500	24.47300	34.40100	57.894000	38.951000	34.288000
O	43.499000	35.335000	34.684000	60.41700	27.08000	33.98300	60.282000	38.744000	33.359000
O	42.338000	37.398000	35.844000	62.43900	26.06500	35.15600	59.719000	37.346000	35.357000
O	44.168000	37.640000	34.099000	60.25300	26.35200	36.56100	59.924000	39.900000	35.742000
O	45.414000	34.395000	36.175000	61.03800	29.65600	33.84900	62.988000	38.166000	33.009000
O	44.378000	39.261000	36.156000	62.61900	26.77200	37.78900	60.853000	38.385000	37.657000
O	47.349000	36.254000	36.467000	61.21800	30.22400	36.49200	64.215000	39.297000	35.056000
O	43.221000	35.498000	37.236000	62.94200	28.05100	33.52700	61.551000	36.261000	33.917000
O	43.733000	37.940000	38.130000	64.21600	27.68300	35.90300	61.799000	36.122000	36.580000
O	45.503000	35.874000	38.359000	63.41300	30.12300	35.17600	63.883000	36.642000	34.985000
O	46.200000	38.240000	37.670000	63.17900	29.27400	37.69500	63.358000	38.023000	37.185000
O	46.066000	35.988000	34.228000	59.29900	28.70700	35.61700	62.116000	40.404000	33.984000
O	46.559000	38.360000	35.106000	60.52700	28.55700	38.11600	62.475000	40.375000	36.664000

O	41.131000	41.277000	29.331000	58.03000	22.18200	36.75000	54.122000	37.738000	38.382000
O	38.986000	42.462000	29.933000	56.75800	23.31000	34.86800	51.555000	37.808000	39.264000
O	41.332000	43.537000	30.591000	55.42100	21.50000	36.30200	53.347000	36.621000	40.693000
O	40.280000	41.662000	32.025000	57.18400	20.70300	34.68200	52.650000	35.581000	38.418000
O	37.146000	44.098000	30.396000	55.38000	23.97800	32.97100	49.248000	37.462000	40.504000
O	41.303000	43.413000	33.208000	54.76600	19.50900	34.70900	53.092000	33.941000	40.442000
O	37.130000	44.215000	33.260000	54.95400	21.82100	31.26200	48.973000	34.598000	40.335000
O	39.437000	45.104000	29.696000	54.05800	23.46500	35.16100	51.356000	37.718000	41.917000
O	40.935000	45.699000	31.838000	53.00200	21.09600	35.14500	52.442000	35.307000	42.608000
O	38.239000	46.025000	31.814000	53.02000	22.66100	32.83700	49.624000	35.878000	42.581000
O	39.557000	45.226000	33.897000	53.37900	20.15500	32.64400	50.832000	33.589000	41.838000
O	37.525000	42.046000	32.070000	57.25900	22.15400	32.51500	49.941000	35.919000	38.351000
O	38.958000	42.543000	34.152000	56.06100	19.78300	32.53700	51.089000	33.691000	38.922000
O	27.859000	40.984000	37.488000	30.93800	26.27500	48.04100	27.117000	45.580000	43.040000
O	26.041000	40.975000	35.369000	29.28000	28.30100	48.04300	29.333000	45.295000	41.450000
O	28.259000	42.425000	35.558000	28.28500	25.97800	47.68900	29.526000	46.121000	44.144000
O	26.157000	42.927000	37.142000	29.78100	27.31400	45.87200	28.579000	47.566000	42.453000
O	24.699000	41.828000	33.317000	27.39700	29.98300	47.95500	31.813000	45.462000	40.495000
O	27.298000	44.940000	36.072000	27.60700	25.98800	44.97400	30.286000	48.615000	44.140000
O	23.984000	44.258000	33.855000	26.55100	30.04900	45.31900	32.434000	48.270000	40.506000
O	27.273000	41.909000	33.251000	26.68300	27.70200	48.93500	31.592000	44.880000	43.085000
O	28.232000	44.303000	33.667000	25.94300	26.25500	46.89600	32.084000	46.796000	44.756000
O	26.039000	43.864000	32.157000	25.23800	28.84600	47.04700	33.408000	46.611000	42.380000
O	26.011000	45.777000	34.084000	25.63800	27.63200	44.71900	32.603000	48.786000	42.970000
O	23.865000	42.457000	35.798000	29.00400	29.89200	46.09500	29.934000	47.374000	40.098000
O	24.619000	44.901000	36.271000	28.10000	28.56600	44.17000	30.359000	49.319000	41.706000
O	25.925000	34.351000	37.631000	34.55000	25.31500	43.82500	27.094000	39.614000	43.533000
O	27.972000	32.728000	37.915000	33.50200	27.09100	42.60300	28.663000	38.684000	45.520000
O	25.594000	31.757000	38.431000	35.05100	25.45500	41.23400	28.105000	37.059000	43.528000
O	26.853000	33.441000	40.032000	36.19600	27.35600	42.62400	26.281000	37.763000	45.292000
O	29.808000	30.950000	38.115000	32.53300	29.03000	41.01700	30.124000	37.487000	47.273000
O	25.713000	31.392000	41.266000	37.09700	27.05300	40.27400	26.496000	35.343000	44.581000
O	29.912000	30.571000	40.925000	34.54200	30.51700	40.24000	28.412000	35.454000	48.487000
O	27.366000	30.085000	37.352000	32.97600	26.62500	40.06500	30.401000	36.865000	44.778000
O	25.960000	29.358000	39.561000	35.11800	26.40800	38.67800	28.904000	34.613000	44.077000
O	28.654000	29.024000	39.381000	33.44300	28.64100	38.61800	30.221000	34.837000	46.425000
O	27.513000	29.485000	41.571000	35.99000	28.74800	38.76200	27.751000	33.998000	46.353000
O	29.542000	32.945000	39.954000	34.49700	29.56900	42.67400	27.542000	38.028000	47.681000
O	28.075000	32.256000	41.907000	36.69200	29.45000	41.25800	26.115000	36.002000	47.255000
O	49.133000	20.369000	35.776000	50.61300	3.08000	48.77400	56.073000	13.911000	37.475000
O	48.203000	20.383000	33.262000	53.06700	2.76300	47.39500	57.666000	11.841000	37.168000
O	48.475000	22.538000	34.600000	51.09400	4.30800	46.50100	55.422000	11.759000	38.554000
O	46.522000	20.822000	35.156000	50.89900	1.63300	46.60800	57.426000	13.008000	39.700000
O	46.843000	21.077000	30.964000	54.60800	2.77300	45.32000	58.701000	9.415000	37.372000
O	45.796000	22.971000	35.328000	49.96200	2.88600	44.52800	55.891000	11.270000	41.157000

O	44.365000	21.556000	31.648000	53.57600	1.29500	43.34500	59.174000	9.174000	40.147000
O	48.611000	22.707000	31.760000	53.44700	5.23700	45.75100	56.193000	9.612000	37.275000
O	47.275000	24.295000	33.359000	51.44000	4.99900	43.95100	55.135000	9.349000	39.569000
O	46.155000	23.364000	30.976000	53.59600	3.89300	43.34500	57.216000	7.768000	38.987000
O	44.836000	23.664000	33.092000	51.54500	2.57100	42.56400	57.079000	8.863000	41.408000
O	45.631000	19.706000	32.851000	53.18900	0.61900	45.82100	59.470000	11.509000	38.959000
O	43.991000	21.340000	34.229000	51.12900	0.55300	44.18400	58.428000	11.188000	41.370000
O	49.756000	16.822000	39.302000	46.17600	5.90500	50.90300	54.680000	18.216000	40.684000
O	51.141000	16.314000	41.720000	46.46100	6.01300	53.46500	55.861000	20.736000	40.566000
O	51.610000	18.357000	40.300000	44.08300	6.14400	52.48000	54.935000	19.570000	42.916000
O	52.232000	15.855000	39.539000	45.64600	8.13700	52.72300	53.350000	20.416000	41.004000
O	52.952000	16.491000	43.702000	46.36100	5.75900	56.03500	56.441000	23.255000	41.299000
O	53.874000	17.796000	39.046000	43.33300	8.56800	53.43900	52.752000	20.859000	43.406000
O	55.198000	16.093000	42.612000	45.63700	8.29200	56.92900	54.139000	24.608000	41.765000
O	51.999000	18.769000	42.789000	44.36000	4.73600	54.75500	56.889000	21.335000	43.037000
O	53.816000	19.626000	40.959000	42.42200	6.42700	54.44100	55.004000	21.396000	44.759000
O	54.609000	18.383000	43.142000	43.96500	6.15200	56.96000	55.812000	23.688000	43.597000
O	55.759000	17.906000	40.717000	43.22300	8.27400	55.95500	53.442000	23.268000	43.921000
O	53.076000	14.620000	41.906000	47.20500	8.06600	54.87900	54.216000	22.698000	39.913000
O	54.847000	15.463000	40.063000	45.16700	9.75600	54.82000	52.270000	22.700000	41.588000
O	54.609000	57.654000	50.649000	17.32000	43.37600	43.56400	4.929000	40.970000	50.327000
O	52.114000	57.315000	50.577000	19.90300	42.22000	44.09800	7.161000	41.586000	48.974000
O	52.755000	59.495000	49.716000	18.78600	44.25900	45.54400	6.555000	39.031000	49.337000
O	52.765000	59.081000	52.278000	19.69200	44.66600	43.18100	7.383000	40.364000	51.221000
O	49.504000	56.926000	50.273000	22.30800	41.79200	44.91500	9.559000	41.404000	47.939000
O	51.833000	61.293000	51.571000	20.16800	46.62000	44.75100	8.154000	37.863000	51.134000
O	48.609000	58.652000	52.075000	23.69000	44.03400	44.38200	11.259000	40.220000	49.709000
O	50.871000	58.261000	48.283000	20.51600	42.68400	46.74500	8.064000	39.453000	47.320000
O	50.830000	60.942000	49.168000	20.67400	45.25600	47.00700	8.491000	37.273000	48.584000
O	48.644000	59.221000	49.518000	22.79500	43.74000	46.83100	10.560000	38.830000	47.671000
O	49.234000	61.003000	51.347000	22.64000	46.10700	45.66000	10.460000	37.841000	50.076000
O	50.766000	57.266000	52.592000	21.88600	43.18500	42.72000	9.450000	41.990000	50.418000
O	50.615000	59.853000	53.402000	22.11000	45.81200	43.12000	9.855000	39.803000	51.811000
O	56.160000	53.822000	47.727000	12.89700	43.93800	40.52300	2.505000	45.033000	48.197000
O	56.046000	52.949000	45.182000	10.82600	44.88600	38.96900	1.229000	46.399000	46.176000
O	53.787000	53.125000	46.561000	11.84400	42.38700	38.61000	3.418000	47.404000	47.193000
O	55.738000	51.312000	47.154000	13.21300	44.57700	38.02900	1.167000	47.307000	48.510000
O	55.564000	51.993000	42.899000	9.09500	45.03500	37.16000	0.199000	48.132000	44.471000
O	53.294000	50.451000	47.319000	13.19100	42.40500	36.18500	2.651000	49.424000	48.756000
O	55.057000	49.441000	43.616000	10.38500	45.21300	34.72400	-0.501000	50.108000	45.920000
O	53.563000	53.376000	43.987000	9.31800	42.63700	38.03500	2.837000	47.880000	44.617000
O	52.100000	51.776000	45.260000	10.90000	41.26600	36.43300	3.648000	49.857000	46.287000
O	53.169000	51.089000	43.034000	9.01900	42.98500	35.40900	1.697000	50.190000	44.564000
O	52.845000	49.440000	44.939000	11.41800	42.74500	34.34700	1.617000	51.237000	47.107000
O	56.865000	50.634000	44.917000	11.33900	46.16300	36.89600	-0.894000	47.824000	46.858000

O	55.264000	48.892000	46.258000	12.94500	44.80300	35.29800	0.104000	49.547000	48.356000
C	35.577000	45.561000	35.961000	53.76500	22.16800	28.53100	46.250000	33.794000	40.965000
C	35.959000	48.052000	36.074000	51.16300	22.37000	28.18900	45.448000	33.390000	43.296000
C	35.979000	46.836000	35.127000	52.37300	22.47300	29.15100	46.585000	34.039000	42.462000
C	37.426000	46.799000	34.661000	52.24500	21.54000	30.36300	47.977000	33.508000	42.643000
C	37.164000	42.232000	36.729000	59.17000	20.00800	31.44300	48.848000	31.047000	38.903000
C	34.743000	41.179000	36.494000	59.44800	20.26200	28.80800	46.631000	31.610000	37.468000
C	35.839000	42.136000	35.978000	58.53600	19.94600	29.99100	47.678000	32.104000	38.486000
C	36.175000	41.702000	34.562000	57.27400	20.83200	30.04600	48.337000	33.409000	37.977000
C	41.726000	45.661000	36.530000	53.16700	16.87000	32.53200	52.246000	32.803000	44.880000
C	43.673000	47.182000	35.927000	51.71700	15.89300	34.56200	54.131000	31.212000	45.044000
C	42.870000	45.980000	35.523000	52.77800	16.90200	34.07800	53.095000	31.808000	44.101000
C	42.290000	46.105000	34.093000	52.20400	18.24300	34.59500	53.601000	32.642000	42.889000
C	44.054000	44.818000	29.406000	51.43500	20.60600	37.96400	54.163000	35.995000	45.316000
C	44.320000	47.101000	28.365000	51.65200	22.40200	39.68700	54.774000	38.523000	45.591000
C	43.429000	46.288000	29.282000	51.88200	22.09600	38.19200	53.888000	37.458000	44.961000
C	41.857000	46.348000	29.224000	53.43200	22.18600	37.78300	53.858000	37.675000	43.401000
C	36.752000	45.397000	27.178000	52.99600	25.72900	30.66000	46.561000	37.893000	43.317000
C	35.477000	47.675000	27.522000	51.90700	27.38000	32.18800	47.340000	39.811000	44.689000
C	36.006000	46.385000	28.157000	52.28300	25.92100	31.99600	47.824000	38.581000	43.896000
C	36.959000	46.792000	29.259000	53.01500	25.43400	33.31700	49.031000	38.858000	42.969000
C	35.084000	39.326000	28.666000	58.64700	26.72500	30.89400	48.671000	41.320000	37.496000
C	37.414000	39.069000	29.885000	57.99800	24.38200	30.20900	49.604000	40.648000	39.738000
C	36.045000	39.825000	29.784000	57.86100	25.52300	31.26000	49.602000	40.387000	38.271000
C	36.271000	41.394000	29.580000	58.04100	24.87400	32.65900	49.283000	38.889000	38.035000
C	42.163000	42.326000	36.336000	57.07700	16.90300	31.91600	53.010000	30.595000	38.934000
C	40.200000	40.653000	36.796000	57.88700	15.43500	33.74500	51.694000	31.309000	36.973000
C	41.540000	40.946000	36.064000	56.95900	16.66800	33.45100	53.099000	31.387000	37.649000
C	41.430000	40.881000	34.567000	57.36400	17.97900	34.12600	53.589000	32.825000	37.832000
C	39.948000	35.844000	38.012000	64.36200	24.97900	32.49500	58.585000	34.688000	36.558000
C	38.659000	35.678000	38.843000	65.30500	23.80200	32.10200	57.696000	33.441000	36.615000
C	38.015000	36.936000	39.325000	66.68800	24.28900	31.71800	58.097000	32.129000	36.020000
C	39.095000	37.812000	39.962000	67.22200	25.49800	32.47600	59.046000	32.207000	34.808000
C	40.276000	38.141000	39.047000	66.25900	26.53200	33.07100	60.197000	33.337000	35.023000
C	41.001000	36.862000	38.468000	64.87100	25.95300	33.52900	59.636000	34.736000	35.419000
C	44.779000	31.966000	34.093000	57.83800	30.35000	32.71900	62.715000	40.428000	30.261000
C	45.195000	30.789000	33.099000	56.77000	30.39600	31.61900	62.385000	41.041000	28.857000
C	44.327000	30.972000	31.807000	55.46100	29.59300	31.90200	61.842000	39.900000	27.962000
C	44.848000	32.291000	31.274000	55.68300	28.30500	32.75900	60.649000	39.240000	28.615000
C	44.732000	33.480000	32.211000	57.07700	28.01100	33.34500	60.894000	38.877000	30.077000
C	45.249000	33.290000	33.615000	58.32400	28.95100	33.14500	61.554000	39.846000	31.015000
C	44.235000	33.716000	40.310000	62.97700	29.72800	30.85200	65.455000	35.646000	32.064000
C	43.936000	32.576000	41.255000	63.92900	29.91300	29.64200	66.049000	34.464000	31.302000
C	43.379000	31.274000	40.718000	64.25000	31.45600	29.59300	65.532000	34.582000	29.893000
C	44.304000	30.830000	39.561000	64.64700	32.13700	30.95200	64.029000	34.686000	29.678000

C	44.372000	31.826000	38.362000	64.01400	31.66600	32.29400	63.310000	35.441000	30.864000
C	44.487000	33.302000	38.826000	63.50300	30.20700	32.20600	63.921000	35.620000	32.277000
C	49.000000	35.469000	39.462000	65.06100	32.10600	37.45700	67.327000	37.500000	35.934000
C	50.263000	35.764000	40.326000	65.41600	33.56000	37.97500	68.585000	36.777000	36.418000
C	49.903000	36.480000	41.618000	64.37200	34.63800	38.24000	68.516000	35.647000	37.436000
C	49.109000	37.776000	41.484000	62.92200	34.33900	38.04900	67.298000	35.956000	38.335000
C	47.899000	37.333000	40.702000	62.71900	32.88500	38.18100	65.971000	36.267000	37.554000
C	48.113000	36.721000	39.258000	63.53900	31.93400	37.33600	65.994000	37.537000	36.721000
C	50.005000	37.242000	34.927000	58.40100	31.16300	38.95300	65.126000	42.628000	33.990000
C	51.438000	37.340000	34.337000	57.22100	31.90800	39.52500	65.967000	43.936000	34.153000
C	51.827000	36.211000	33.371000	56.64000	32.82500	38.42100	65.330000	45.171000	34.946000
C	50.605000	35.527000	32.749000	56.30300	32.15000	37.02400	64.239000	44.679000	35.859000
C	49.333000	35.328000	33.511000	57.53100	31.29800	36.52400	63.379000	43.427000	35.679000
C	48.855000	36.750000	34.078000	58.39600	30.50900	37.54700	64.114000	42.159000	35.137000
C	44.946000	41.114000	39.854000	65.99200	26.26200	38.70200	63.400000	36.367000	40.152000
C	44.808000	42.605000	40.264000	66.86000	25.93800	39.90900	63.829000	35.123000	41.025000
C	45.668000	43.563000	39.465000	66.30300	26.00400	41.35500	62.777000	34.040000	41.161000
C	45.273000	43.280000	37.993000	65.42200	27.27100	41.43000	61.326000	34.353000	40.702000
C	45.586000	41.816000	37.477000	64.52300	27.53800	40.20500	61.125000	35.088000	39.408000
C	44.756000	40.876000	38.348000	65.07200	27.49600	38.80800	62.228000	36.179000	39.126000
C	43.937000	40.746000	32.839000	59.14800	25.51200	39.59100	60.358000	42.427000	38.171000
C	44.016000	42.114000	32.261000	59.00000	24.29200	40.59400	59.538000	43.305000	39.088000
C	44.929000	43.283000	32.799000	59.76000	24.63500	41.84400	58.093000	43.092000	39.212000
C	45.959000	42.858000	33.872000	61.23400	24.83000	41.59800	57.875000	41.650000	39.540000
C	46.190000	41.373000	33.942000	61.50600	25.89100	40.51000	58.624000	40.620000	38.723000
C	45.242000	40.225000	33.565000	60.63400	25.80600	39.28600	60.117000	40.943000	38.328000
C	41.297000	38.834000	30.957000	60.07800	20.85500	35.40400	56.593000	37.383000	37.380000
C	39.215000	37.526000	34.741000	63.01500	23.05200	34.66900	56.453000	37.453000	32.753000
C	39.148000	35.784000	33.104000	61.48700	23.25000	32.24900	55.268000	39.486000	35.012000
C	39.951000	38.348000	31.586000	60.29000	21.61900	34.04700	56.370000	37.039000	35.904000
C	43.246000	39.451000	28.640000	59.62100	20.66400	38.54700	55.448000	39.178000	40.184000
C	40.422000	39.153000	27.993000	60.60800	23.22600	37.46200	55.426000	40.269000	37.865000
C	29.866000	25.906000	40.034000	32.25200	30.88000	36.86100	27.331000	31.154000	48.398000
C	30.689000	25.490000	42.332000	33.67600	32.47100	35.76100	29.026000	31.558000	50.451000
C	29.700000	26.370000	41.510000	33.70600	31.05500	36.44200	28.240000	32.133000	49.216000
C	30.042000	27.862000	41.740000	34.81600	30.95700	37.50300	29.385000	32.821000	48.422000
C	29.872000	35.160000	42.205000	37.19200	32.50900	39.86800	24.781000	38.434000	49.826000
C	31.643000	34.846000	43.842000	37.28700	34.33600	41.67100	25.301000	37.733000	52.105000
C	30.441000	34.271000	43.267000	36.42900	33.23400	40.97900	25.246000	37.216000	50.650000
C	30.691000	32.913000	42.614000	36.00300	32.18900	42.07100	26.590000	36.603000	50.072000
C	23.025000	29.934000	41.818000	38.98900	24.72900	38.76300	24.699000	32.538000	45.358000
C	23.206000	28.042000	43.490000	39.88200	25.51200	36.60500	25.547000	30.585000	43.762000
C	23.593000	28.541000	42.137000	38.61600	25.50100	37.45700	25.411000	32.078000	44.066000
C	25.086000	28.721000	42.239000	38.06800	26.90300	37.74000	26.840000	32.684000	43.964000
C	25.214000	27.227000	36.464000	35.73600	22.76000	38.58600	32.036000	34.786000	42.455000

C	23.698000	27.191000	38.488000	35.01300	22.37400	41.04700	30.563000	33.991000	40.507000
C	24.088000	28.023000	37.252000	34.52600	22.63600	39.61800	31.060000	35.197000	41.318000
C	24.358000	29.492000	37.444000	33.63300	23.87600	39.43100	30.040000	36.154000	41.944000
C	29.974000	26.281000	35.189000	28.46000	28.05300	38.07500	34.811000	35.736000	47.072000
C	27.888000	27.442000	34.952000	29.08100	29.21000	40.28200	33.129000	33.982000	46.794000
C	29.013000	27.216000	35.989000	29.33400	28.00500	39.32800	33.312000	35.509000	47.132000
C	29.664000	28.451000	36.660000	30.68600	28.15400	38.79800	32.560000	36.454000	46.242000
C	29.382000	34.873000	35.625000	32.75200	30.99500	45.33100	27.953000	40.713000	49.409000
C	30.453000	32.759000	35.216000	32.86000	28.56100	46.14000	27.329000	41.537000	46.966000
C	30.542000	34.007000	36.034000	32.08100	29.57800	45.31400	28.425000	41.381000	48.092000
C	30.642000	33.764000	37.551000	31.79600	29.03800	43.88000	29.471000	40.415000	47.450000
C	23.852000	34.733000	44.217000	41.03400	27.37000	43.69200	21.826000	35.720000	43.990000
C	23.777000	34.422000	41.657000	38.80200	28.28900	44.57600	23.965000	36.554000	42.963000
C	24.228000	33.845000	43.020000	39.52600	27.41100	43.56600	23.340000	35.592000	44.006000
C	25.805000	33.796000	42.915000	39.10900	27.82900	42.12500	23.900000	36.039000	45.374000
C	31.403000	42.140000	34.199000	25.23100	23.79900	49.21100	32.633000	43.644000	45.985000
C	32.588000	41.295000	33.663000	24.33000	23.25800	50.34100	32.961000	42.566000	47.013000
C	32.807000	41.157000	32.142000	24.89900	23.28500	51.71000	32.375000	42.790000	48.419000
C	31.477000	40.762000	31.524000	25.50500	24.67800	51.98700	31.022000	43.507000	48.400000
C	30.373000	41.834000	31.809000	26.56900	25.05700	50.94900	30.581000	44.476000	47.270000
C	30.121000	42.014000	33.373000	25.93900	25.17300	49.56600	31.332000	44.415000	45.928000
C	23.064000	38.947000	36.178000	31.45600	30.87500	48.90900	30.761000	45.006000	37.472000
C	21.946000	37.860000	35.965000	32.32100	31.78600	49.80700	30.472000	44.041000	36.347000
C	22.184000	36.967000	34.696000	32.13200	33.26500	49.45800	29.068000	43.727000	35.967000
C	22.772000	37.737000	33.493000	30.65100	33.61200	49.30900	28.120000	43.568000	37.183000
C	23.770000	38.913000	33.755000	29.76000	32.66200	48.34300	28.374000	44.568000	38.360000
C	23.477000	39.828000	34.954000	29.98100	31.16900	48.65500	29.875000	44.813000	38.745000
C	26.755000	41.368000	29.777000	25.61500	29.78800	51.30900	34.590000	43.425000	42.925000
C	26.454000	40.775000	28.338000	24.28600	29.87000	52.10700	35.457000	42.191000	42.886000
C	25.499000	39.584000	28.359000	23.25500	31.02000	51.84600	35.071000	40.957000	42.028000
C	24.276000	39.586000	29.277000	22.92200	31.25000	50.35400	33.926000	41.306000	41.063000
C	24.667000	40.165000	30.675000	24.13700	30.97400	49.37700	33.053000	42.570000	41.175000
C	25.560000	41.496000	30.734000	25.15000	29.85400	49.78200	33.744000	43.820000	41.726000
C	23.393000	45.674000	30.719000	23.29800	31.15900	45.29500	36.071000	47.987000	40.542000
C	22.431000	46.511000	29.879000	21.87000	31.58300	45.15600	37.506000	48.594000	40.589000
C	22.036000	47.822000	30.447000	20.84700	30.54300	45.13100	37.654000	50.141000	40.721000
C	23.030000	48.548000	31.300000	21.13400	29.31800	44.23400	36.793000	50.566000	41.887000
C	23.838000	47.625000	32.243000	22.49200	28.67500	44.66500	35.336000	50.160000	41.605000
C	24.370000	46.284000	31.705000	23.64700	29.80200	44.69800	35.153000	48.651000	41.611000
C	21.317000	44.511000	37.015000	27.77600	31.51400	42.18700	30.758000	49.520000	37.484000
C	19.904000	45.152000	37.224000	28.31100	32.41500	41.06500	30.000000	50.485000	36.609000
C	19.658000	46.623000	36.795000	28.83200	33.80500	41.60200	30.334000	52.001000	36.681000
C	20.153000	46.620000	35.389000	29.40700	33.85400	43.02400	30.312000	52.422000	38.149000
C	21.525000	46.024000	35.058000	28.80500	32.84100	44.03200	30.955000	51.366000	39.109000
C	21.890000	44.627000	35.592000	28.45500	31.43300	43.53200	30.479000	49.899000	38.959000

C	28.829000	47.824000	33.428000	25.35200	24.27400	43.14900	33.936000	48.940000	46.454000
C	29.957000	48.851000	33.487000	24.65000	22.98600	42.59900	34.630000	49.795000	47.485000
C	29.641000	49.870000	34.568000	23.13000	23.23300	42.77400	34.465000	51.342000	47.414000
C	29.201000	49.173000	35.943000	22.85700	23.32000	44.29700	33.152000	51.731000	46.744000
C	28.261000	47.986000	35.837000	23.62800	24.50700	45.01400	32.589000	50.971000	45.526000
C	28.505000	47.060000	34.680000	25.10000	24.80900	44.56600	32.682000	49.479000	45.685000
C	27.556000	45.398000	39.440000	30.53100	25.12700	43.47200	28.733000	51.667000	44.120000
C	27.891000	46.361000	40.631000	31.43900	24.63400	42.38400	28.116000	53.018000	44.140000
C	27.686000	47.848000	40.190000	30.48700	23.95400	41.37600	26.605000	52.789000	44.199000
C	26.311000	48.054000	39.518000	29.31900	24.83800	40.93600	25.969000	51.805000	43.212000
C	25.847000	47.105000	38.402000	28.69200	25.90600	41.87400	26.568000	50.417000	43.276000
C	26.292000	45.566000	38.546000	29.59900	26.27800	43.09300	28.153000	50.479000	43.292000
C	26.590000	37.001000	36.894000	33.42500	25.00500	46.13100	26.564000	42.285000	43.966000
C	28.110000	39.341000	39.798000	30.44800	23.58300	48.27400	24.809000	46.604000	44.041000
C	29.371000	39.096000	36.788000	31.74500	25.13600	50.41800	26.917000	45.415000	45.982000
C	26.712000	38.569000	37.034000	33.31000	24.66100	47.61800	25.642000	43.483000	44.153000
C	26.191000	36.521000	39.772000	36.77000	25.65200	45.63200	24.195000	40.181000	44.408000
C	23.759000	36.175000	38.181000	35.20200	23.05800	45.03800	25.424000	40.766000	41.774000
C	59.841000	16.848000	42.761000	42.86400	8.17700	61.23600	54.165000	27.607000	46.366000
C	58.359000	15.978000	44.488000	41.55900	7.29500	59.38400	53.719000	25.012000	46.485000
C	58.373000	16.551000	43.063000	42.99000	7.28300	59.95400	54.525000	26.176000	45.727000
C	57.497000	17.677000	42.832000	43.66700	8.21800	58.91400	54.135000	26.103000	44.270000
C	54.383000	11.676000	40.477000	45.05600	11.79600	57.14600	53.990000	26.474000	39.141000
C	56.347000	11.123000	41.833000	47.46200	12.73500	57.55200	51.794000	26.654000	37.909000
C	55.677000	12.224000	40.966000	46.56000	11.49900	57.44300	52.743000	25.748000	38.650000
C	55.617000	13.464000	41.801000	47.28100	10.59900	56.39500	52.199000	24.853000	39.760000
C	56.340000	18.258000	37.448000	40.47800	10.61100	54.53900	53.446000	19.082000	46.167000
C	57.016000	20.623000	36.681000	38.34800	9.42300	53.92400	51.421000	19.810000	47.464000
C	55.943000	19.720000	37.426000	39.85800	9.44200	53.76300	52.725000	20.263000	46.859000
C	55.636000	20.087000	38.910000	40.54500	8.22200	54.42800	52.237000	21.355000	45.942000
C	52.266000	22.029000	43.512000	44.51300	3.31300	50.94700	58.744000	20.453000	45.980000
C	53.136000	22.813000	41.096000	43.72000	1.60000	52.86300	57.182000	19.056000	47.295000
C	52.023000	22.300000	42.067000	43.41400	2.81700	51.86900	57.859000	19.255000	46.020000
C	51.391000	21.085000	41.380000	42.60800	3.94600	52.60800	57.020000	19.013000	44.735000
C	53.778000	19.557000	47.701000	45.27100	1.54400	58.61500	60.035000	25.579000	44.213000
C	54.724000	17.422000	46.687000	45.28700	3.79700	59.80500	57.942000	26.043000	42.854000
C	54.233000	18.884000	46.426000	44.65600	3.01000	58.56500	58.631000	25.061000	43.842000
C	53.108000	18.891000	45.406000	44.92300	3.38200	57.04100	58.559000	23.656000	43.285000
C	49.823000	16.516000	45.084000	49.95600	6.95100	57.04600	55.876000	24.548000	37.281000
C	48.318000	15.030000	43.883000	49.40800	4.59100	56.90400	58.080000	24.166000	38.270000
C	49.662000	15.031000	44.631000	49.93200	5.71800	56.05500	56.942000	23.525000	37.529000
C	50.919000	14.520000	44.032000	48.97700	5.87700	54.77700	56.624000	22.242000	38.370000
C	56.243000	14.178000	37.444000	42.25900	10.08500	50.87500	49.215000	22.814000	42.116000
C	55.244000	14.954000	35.160000	44.14800	11.27600	49.45600	47.925000	20.744000	42.364000
C	55.421000	15.177000	36.681000	43.78500	10.36400	50.67700	49.285000	21.351000	42.361000

C	54.063000	15.252000	37.488000	44.56900	10.63500	51.93300	50.442000	20.577000	41.697000
C	50.721000	25.041000	32.022000	51.61600	8.29400	44.90300	53.124000	8.689000	36.482000
C	51.679000	26.218000	32.018000	51.23800	9.77200	45.19900	52.026000	8.494000	35.359000
C	52.408000	26.755000	33.270000	51.08900	10.23100	46.62900	50.779000	9.433000	35.506000
C	52.325000	25.786000	34.488000	50.80200	9.00900	47.61100	51.279000	10.842000	35.948000
C	51.101000	24.808000	34.512000	51.26600	7.61200	47.18800	52.293000	10.925000	37.164000
C	50.261000	24.367000	33.276000	51.21900	7.06100	45.80600	53.539000	10.059000	37.100000
C	46.331000	17.961000	30.185000	55.12100	0.07800	48.42500	59.719000	11.851000	34.670000
C	46.608000	16.756000	29.297000	56.15100	-0.13700	49.55900	60.517000	12.037000	33.439000
C	47.825000	15.933000	29.826000	56.77700	1.13500	50.14900	61.975000	11.609000	33.531000
C	49.232000	16.613000	30.038000	57.41300	1.99900	49.03700	62.568000	11.559000	34.955000
C	48.842000	18.035000	30.584000	56.40800	2.22500	47.86000	61.721000	11.181000	36.188000
C	47.429000	18.483000	31.163000	55.41200	1.19400	47.38900	60.248000	11.620000	36.129000
C	46.988000	22.656000	27.693000	57.12300	4.98100	45.56200	55.678000	6.451000	36.103000
C	47.462000	23.094000	26.304000	58.49600	5.60700	45.28600	55.238000	5.863000	34.741000
C	48.122000	24.476000	26.200000	58.44400	6.78000	44.29200	56.155000	4.676000	34.344000
C	49.218000	24.556000	27.277000	57.34600	6.82200	43.23300	57.662000	5.077000	34.508000
C	48.617000	24.366000	28.678000	56.02500	6.47000	43.76900	58.041000	6.070000	35.683000
C	48.078000	22.941000	28.809000	56.00300	5.07200	44.48100	57.043000	7.141000	36.213000
C	41.874000	23.944000	30.988000	55.35600	2.97500	40.70300	59.576000	6.153000	42.059000
C	40.875000	24.568000	29.998000	55.91000	3.08000	39.22800	60.226000	4.815000	42.265000
C	41.189000	26.031000	29.736000	55.37600	2.18600	38.10800	60.910000	4.270000	41.011000
C	42.593000	26.253000	29.050000	53.95500	1.76300	38.27700	60.184000	4.393000	39.642000
C	43.527000	25.647000	30.151000	53.46000	1.50800	39.69700	59.476000	5.703000	39.428000
C	43.345000	24.161000	30.634000	53.87700	2.55100	40.75800	58.897000	6.464000	40.656000
C	42.691000	18.015000	33.029000	52.34900	-2.76600	44.36600	62.067000	12.021000	41.748000
C	41.423000	17.100000	33.156000	52.88900	-4.21600	44.20800	63.559000	11.955000	42.211000
C	40.072000	17.847000	33.008000	54.40200	-4.45100	44.61100	63.744000	10.895000	43.271000
C	40.076000	19.247000	32.280000	55.27600	-3.35100	44.09100	63.070000	9.606000	42.949000
C	41.315000	20.127000	32.615000	54.75500	-1.88800	44.11200	61.806000	9.635000	42.186000
C	42.718000	19.464000	32.513000	53.25700	-1.55100	43.95800	61.368000	10.773000	41.241000
C	46.458000	26.123000	36.233000	49.18400	5.20700	41.19100	52.948000	8.924000	41.617000
C	46.129000	27.361000	37.078000	48.06600	5.50600	40.09700	51.907000	8.356000	42.657000
C	45.399000	28.511000	36.254000	47.28600	4.21400	39.80700	51.828000	9.080000	44.007000
C	44.397000	27.927000	35.200000	47.16200	2.87500	40.67100	53.236000	8.762000	44.553000
C	44.932000	26.790000	34.328000	48.42400	2.75000	41.53700	54.454000	9.216000	43.699000
C	45.436000	25.622000	35.206000	49.25600	3.96200	42.09500	54.388000	9.023000	42.139000
C	43.086000	21.867000	37.683000	47.62500	0.05500	44.38800	56.756000	12.702000	44.051000
C	43.094000	22.740000	38.916000	46.57500	-1.02900	44.67600	56.401000	13.583000	45.281000
C	43.917000	22.262000	40.178000	46.36200	-1.23900	46.17100	57.631000	14.588000	45.493000
C	45.248000	21.650000	39.675000	47.46400	-1.01100	47.26300	57.980000	15.303000	44.166000
C	45.260000	20.872000	38.331000	48.30600	0.24100	47.02700	58.240000	14.299000	43.075000
C	44.420000	21.434000	37.214000	48.58500	0.52600	45.53100	57.134000	13.330000	42.693000
C	47.282000	17.900000	38.503000	48.40600	6.63000	49.08200	56.143000	15.992000	40.440000
C	49.774000	20.674000	38.328000	50.17200	4.98900	51.24000	53.975000	15.832000	37.072000

C	50.802000	18.394000	36.730000	48.29200	3.00800	49.89000	56.802000	16.199000	36.179000
C	47.658000	18.748000	37.247000	49.39900	5.56700	48.53600	56.585000	16.239000	39.026000
C	47.442000	15.674000	40.769000	46.12400	8.28000	49.48300	52.998000	15.959000	40.813000
C	48.250000	14.989000	38.014000	44.95200	6.14400	48.52500	55.035000	16.641000	43.155000
C	51.086000	47.291000	43.813000	9.87800	45.82300	31.18600	-1.373000	53.800000	46.411000
C	52.094000	45.873000	42.053000	11.01900	43.32700	30.97800	0.936000	54.717000	46.699000
C	52.413000	47.023000	43.006000	9.79300	44.27500	31.26800	0.005000	53.990000	45.765000
C	52.620000	48.334000	42.303000	9.12600	43.78600	32.61100	0.699000	52.792000	45.036000
C	57.617000	45.303000	43.972000	13.78100	47.96500	32.18700	-4.943000	49.916000	48.838000
C	56.286000	46.483000	42.296000	13.47000	45.51300	32.47900	-3.115000	50.270000	50.296000
C	56.502000	46.338000	43.826000	13.57700	46.86100	33.21200	-3.489000	49.641000	48.907000
C	57.070000	47.639000	44.422000	12.26800	47.09100	33.97600	-2.667000	50.021000	47.594000
C	48.352000	49.734000	48.055000	13.67300	37.88700	34.50700	5.899000	53.363000	48.931000
C	49.023000	51.847000	46.536000	14.73900	39.77000	35.86700	3.588000	53.833000	47.882000
C	49.470000	50.705000	47.451000	14.01300	39.38300	34.57200	4.771000	52.807000	47.976000
C	50.465000	49.796000	46.696000	12.64000	40.05900	34.47300	4.229000	51.458000	48.518000
C	53.232000	56.089000	45.854000	8.30100	38.98800	38.23100	5.416000	45.252000	45.625000
C	51.647000	56.483000	43.907000	10.69100	37.88100	37.80200	5.385000	46.336000	43.406000
C	51.773000	56.050000	45.385000	9.73900	38.78400	38.69300	5.913000	46.448000	44.803000
C	51.486000	54.576000	45.654000	10.49500	40.12400	39.06000	5.577000	47.762000	45.376000
C	51.420000	52.802000	40.639000	6.85200	41.04700	36.17600	2.169000	46.181000	41.969000
C	52.484000	54.655000	39.456000	4.60700	41.73500	37.27900	2.326000	47.688000	39.831000
C	52.547000	53.843000	40.739000	6.13800	41.84100	37.28800	1.695000	47.449000	41.225000
C	53.825000	53.047000	41.065000	6.74100	43.24600	37.09500	1.797000	48.704000	42.079000
C	59.648000	52.880000	45.667000	9.26800	49.13900	37.18400	-3.445000	46.656000	45.095000
C	60.307000	50.987000	44.064000	7.42400	47.62500	37.86600	-2.257000	46.028000	42.848000
C	59.721000	52.407000	44.216000	8.63200	48.42300	38.34900	-2.476000	45.808000	44.337000
C	58.384000	52.481000	43.452000	9.65000	47.42500	38.96100	-1.129000	45.587000	45.091000
C	57.622000	48.838000	49.566000	16.36500	42.43800	34.79400	-1.173000	47.796000	51.231000
C	56.782000	51.109000	49.950000	16.19900	45.05200	34.54900	0.870000	46.431000	52.145000
C	56.426000	49.582000	49.993000	16.41700	43.81600	35.48200	0.217000	47.831000	51.864000
C	55.085000	49.453000	49.281000	15.48100	44.00500	36.66000	1.071000	48.882000	51.102000
C	52.882000	61.905000	47.087000	18.16500	45.21900	49.18400	5.348000	38.381000	45.797000
C	54.343000	62.150000	47.263000	16.75700	45.33400	49.93900	4.632000	37.708000	44.622000
C	55.341000	61.277000	46.535000	15.84400	44.11200	50.07400	4.828000	36.203000	44.246000
C	54.852000	59.807000	46.588000	16.61200	42.74000	49.85900	5.306000	35.300000	45.355000
C	53.387000	59.618000	46.176000	17.55500	42.66000	48.68800	6.115000	35.930000	46.551000
C	52.418000	60.400000	46.996000	18.49500	43.83100	48.47300	6.161000	37.491000	46.802000
C	51.141000	53.981000	49.790000	22.31600	39.73200	41.74600	10.107000	44.702000	49.028000
C	51.368000	52.486000	49.822000	21.98200	38.52500	40.82700	10.157000	46.223000	48.866000
C	52.458000	52.017000	50.776000	21.06400	37.40000	41.38900	9.278000	46.878000	47.760000
C	52.329000	52.714000	52.154000	19.94200	37.99300	42.33400	7.944000	46.232000	47.420000
C	52.267000	54.224000	52.010000	20.21400	39.32600	42.99300	8.087000	44.696000	47.475000
C	51.092000	54.747000	51.133000	21.18100	40.41700	42.53000	8.725000	44.064000	48.722000
C	48.631000	58.025000	46.188000	22.16100	41.31500	49.28300	9.992000	39.080000	44.376000

C	48.233000	57.348000	44.873000	22.58200	40.40200	50.44100	9.968000	39.570000	42.858000
C	46.993000	56.416000	44.968000	23.91900	39.65700	50.33000	11.362000	40.248000	42.514000
C	47.087000	55.448000	46.157000	24.55300	39.53900	48.89800	11.777000	41.313000	43.538000
C	47.455000	56.157000	47.473000	24.13900	40.51000	47.73400	11.411000	41.021000	44.994000
C	48.661000	57.155000	47.413000	22.70700	41.10500	47.82300	10.096000	40.287000	45.284000
C	45.327000	60.330000	50.338000	26.36100	44.26400	46.47200	14.205000	38.283000	49.254000
C	43.874000	60.848000	50.473000	26.91800	43.80500	45.20000	15.678000	37.917000	48.924000
C	43.565000	61.603000	51.783000	27.31800	44.94300	44.25800	15.737000	36.495000	48.329000
C	44.884000	62.189000	52.369000	26.45200	46.16400	44.18800	14.560000	36.279000	47.379000
C	45.934000	61.151000	52.644000	25.88200	46.55300	45.56400	13.107000	36.820000	47.613000
C	46.545000	60.541000	51.335000	25.21500	45.31800	46.29800	13.002000	38.206000	48.350000
C	48.142000	58.662000	55.589000	23.71000	44.86500	40.31800	12.615000	41.403000	52.913000
C	46.973000	58.037000	56.439000	24.69200	45.18100	39.16000	13.111000	42.397000	54.008000
C	46.411000	56.610000	56.155000	25.86200	44.15000	39.27800	13.858000	43.500000	53.301000
C	47.501000	55.655000	55.613000	26.48200	43.80000	40.66900	12.949000	44.343000	52.288000
C	48.655000	56.310000	54.758000	25.28100	43.35700	41.64400	12.055000	43.501000	51.466000
C	49.059000	57.818000	54.784000	24.20300	44.48700	41.69000	11.642000	42.088000	51.933000
C	50.738000	64.135000	51.752000	19.77300	48.67800	47.56600	10.475000	34.469000	49.618000
C	50.828000	65.709000	51.848000	19.89300	50.17000	48.05100	10.915000	33.090000	50.175000
C	49.851000	66.436000	50.909000	21.02200	50.37700	49.08600	9.874000	32.180000	50.875000
C	49.730000	65.730000	49.516000	22.39400	49.90300	48.52500	8.400000	32.812000	51.002000
C	49.486000	64.151000	49.667000	22.33800	48.57200	47.78500	8.167000	34.282000	50.576000
C	50.577000	63.401000	50.439000	21.09100	48.30500	46.88400	9.361000	35.204000	50.411000
C	54.446000	61.614000	54.260000	18.28000	47.45900	42.04200	8.271000	40.250000	54.546000
C	54.928000	62.538000	55.400000	17.94900	48.66800	41.20600	7.206000	41.027000	55.406000
C	54.131000	63.792000	55.844000	18.44100	48.75100	39.72400	6.049000	40.239000	56.108000
C	52.747000	63.252000	56.171000	19.95200	48.32000	39.56100	5.887000	38.811000	55.479000
C	52.174000	62.581000	54.867000	20.36300	47.14300	40.48300	6.252000	38.582000	53.917000
C	52.917000	61.350000	54.236000	19.87600	47.22600	41.94900	7.635000	39.211000	53.634000
C	56.407000	54.738000	50.181000	13.99100	45.56000	42.25800	2.944000	44.119000	50.573000
C	55.514000	57.882000	53.221000	15.18100	43.91500	45.49500	4.941000	41.706000	52.977000
C	57.235000	58.311000	50.556000	14.91600	41.89700	43.56200	2.508000	40.110000	51.248000
C	55.995000	55.550000	51.426000	15.33400	44.84900	42.47800	3.655000	43.254000	51.609000
C	53.739000	54.990000	48.686000	11.96800	43.28800	43.27900	5.256000	45.304000	48.850000
C	56.110000	56.550000	47.535000	11.78900	46.21600	41.82000	4.079000	42.868000	47.722000
H	35.843000	44.815000	35.232000	54.34900	22.62600	29.35400	47.097000	34.169000	40.441000
H	34.617000	45.493000	36.317000	53.80300	22.82000	27.70100	45.383000	34.143000	40.486000
H	36.344000	45.551000	36.801000	54.07500	21.16500	28.28800	46.263000	32.713000	40.866000
H	36.120000	48.970000	35.594000	50.39900	22.70300	28.89200	45.749000	33.536000	44.348000
H	36.511000	47.797000	36.930000	50.85000	21.39400	27.90600	45.245000	32.337000	43.077000
H	34.928000	48.017000	36.444000	51.35100	23.01600	27.39400	44.512000	33.930000	43.039000
H	35.370000	47.092000	34.272000	52.52800	23.53300	29.46300	46.628000	35.077000	42.649000
H	37.412000	47.827000	34.346000	51.33500	21.86600	30.83700	48.072000	33.721000	43.717000
H	37.967000	46.670000	35.545000	51.93100	20.52500	30.12500	48.216000	32.509000	42.413000
H	37.727000	42.953000	36.206000	58.35000	19.93500	32.14700	49.308000	31.848000	39.507000

H	37.646000	41.238000	36.690000	59.84000	20.76300	31.78300	49.401000	30.843000	37.996000
H	36.969000	42.589000	37.765000	59.70500	19.12200	31.62900	48.883000	30.156000	39.644000
H	34.418000	41.321000	37.510000	60.32500	19.61700	28.85400	46.076000	30.775000	37.827000
H	35.294000	40.297000	36.457000	59.85500	21.23700	28.75600	47.294000	31.436000	36.622000
H	34.062000	41.328000	35.660000	58.75600	20.13800	27.95400	45.937000	32.475000	37.428000
H	35.364000	43.136000	35.869000	58.08300	18.94200	30.12200	47.152000	32.276000	39.397000
H	36.847000	40.947000	34.967000	57.88200	21.70600	30.12000	48.912000	32.728000	37.258000
H	35.487000	41.372000	33.807000	56.53700	20.87300	29.20200	47.853000	34.158000	37.370000
H	41.946000	45.516000	37.597000	53.35800	15.88900	32.32400	51.899000	32.415000	45.792000
H	41.185000	44.809000	36.114000	54.07900	17.37000	32.24800	51.409000	33.322000	44.354000
H	41.173000	46.548000	36.379000	52.27300	17.06800	31.94200	52.987000	33.517000	45.193000
H	44.162000	47.155000	36.905000	52.13800	14.88900	34.69600	53.474000	30.629000	45.626000
H	43.140000	48.117000	35.920000	50.72500	15.92800	34.26300	54.631000	31.957000	45.637000
H	44.516000	47.335000	35.151000	51.70600	16.37700	35.54500	54.795000	30.456000	44.629000
H	43.480000	45.153000	35.500000	53.80100	16.70500	34.48000	52.498000	30.955000	43.769000
H	42.007000	47.008000	34.615000	51.23000	18.22400	34.07000	54.289000	33.290000	43.372000
H	43.050000	46.339000	33.379000	51.88300	18.04900	35.56800	54.096000	32.127000	42.035000
H	43.980000	44.214000	28.554000	52.12500	20.44200	38.70100	54.988000	35.547000	44.864000
H	43.676000	44.381000	30.330000	51.68500	20.19400	36.97100	53.285000	35.468000	45.054000
H	45.136000	45.043000	29.577000	50.44300	20.39500	38.21800	54.309000	36.014000	46.371000
H	45.313000	46.968000	28.761000	50.63200	22.41500	39.94900	54.767000	38.538000	46.703000
H	44.019000	48.171000	28.584000	52.11600	23.29500	40.10400	54.406000	39.411000	45.130000
H	44.270000	46.718000	27.318000	52.06600	21.47500	40.16300	55.733000	38.181000	45.260000
H	43.689000	46.818000	30.129000	51.18800	22.79800	37.72800	52.886000	37.550000	45.348000
H	41.482000	47.394000	29.381000	53.55600	23.17800	38.19400	53.513000	38.721000	43.257000
H	41.425000	46.115000	28.269000	53.90200	21.44600	38.45100	54.872000	37.452000	43.115000
H	37.538000	46.149000	27.106000	54.03900	25.90200	30.43100	46.307000	38.640000	42.561000
H	36.060000	45.212000	26.369000	52.43900	26.55000	30.17200	46.035000	37.981000	44.251000
H	37.000000	44.426000	27.504000	52.76700	24.68500	30.36700	46.759000	36.913000	42.842000
H	34.778000	48.257000	28.055000	51.35300	27.41500	33.09500	48.081000	40.502000	45.149000
H	35.031000	47.249000	26.612000	51.54300	27.59400	31.20900	46.497000	39.385000	45.323000
H	36.402000	48.299000	27.320000	52.75100	28.02500	32.44900	46.916000	40.258000	43.777000
H	35.300000	45.729000	28.647000	51.34000	25.43100	31.85300	48.161000	37.728000	44.451000
H	37.716000	47.356000	28.722000	53.86700	26.07900	33.29800	48.621000	39.581000	42.260000
H	36.429000	47.244000	30.059000	52.37300	25.82300	34.11100	49.945000	39.187000	43.491000
H	35.617000	39.329000	27.722000	59.69900	26.79700	31.04800	47.789000	40.952000	38.053000
H	34.187000	39.883000	28.537000	58.27100	27.43200	31.48800	48.623000	41.190000	36.445000
H	34.821000	38.228000	28.929000	58.31200	27.03600	29.92900	49.185000	42.210000	37.822000
H	36.887000	38.092000	29.801000	58.20800	24.64800	29.18200	49.942000	41.720000	39.865000
H	37.840000	39.066000	30.912000	57.38000	23.47000	30.21700	50.272000	40.033000	40.294000
H	38.094000	39.216000	29.053000	58.98600	24.12800	30.64300	48.614000	40.351000	39.993000
H	35.510000	39.707000	30.735000	56.81800	25.77600	31.22500	50.603000	40.647000	37.880000
H	36.845000	41.249000	28.632000	59.08200	24.63000	32.67900	48.324000	38.913000	38.535000
H	35.398000	42.069000	29.368000	57.83100	25.66900	33.37200	49.094000	38.726000	37.013000
H	42.060000	42.305000	37.413000	56.56800	16.07400	31.36300	52.601000	29.603000	38.751000

H	41.613000	43.138000	35.865000	58.11000	16.96300	31.66900	52.330000	31.087000	39.583000
H	43.148000	42.420000	35.902000	56.59400	17.86800	31.66700	54.047000	30.611000	39.226000
H	39.889000	39.602000	36.647000	57.89900	15.33400	34.80700	51.649000	31.781000	35.972000
H	39.385000	41.378000	36.798000	58.86600	15.83200	33.39600	50.893000	31.814000	37.524000
H	40.559000	40.680000	37.810000	57.69600	14.45700	33.34500	51.587000	30.232000	36.996000
H	42.417000	40.360000	36.267000	55.99300	16.33300	33.75200	53.877000	30.827000	37.178000
H	42.391000	41.314000	34.249000	57.34400	17.65900	35.15800	54.505000	32.523000	38.286000
H	41.262000	39.988000	33.932000	58.35900	18.28700	33.92500	53.875000	33.482000	37.037000
H	40.526000	34.954000	37.846000	63.32400	25.05100	32.56300	57.878000	35.508000	36.494000
H	38.208000	34.845000	38.238000	64.85500	23.07100	31.48300	56.979000	33.375000	37.464000
H	37.188000	36.648000	39.957000	67.40100	23.49600	31.46100	57.344000	31.393000	35.842000
H	38.849000	38.772000	40.410000	68.19700	25.83000	32.29200	59.307000	31.162000	34.510000
H	40.933000	38.655000	39.738000	66.79300	27.33300	33.54900	61.008000	33.489000	34.248000
H	45.128000	31.827000	35.153000	58.71900	30.79500	32.34200	63.115000	41.249000	30.817000
H	44.553000	29.974000	33.521000	56.57600	31.34500	31.20800	63.371000	41.483000	28.680000
H	44.390000	30.200000	31.027000	54.54000	29.73900	31.32700	61.925000	40.379000	26.997000
H	44.204000	32.417000	30.421000	54.71200	27.83100	33.00400	59.894000	38.590000	28.247000
H	45.066000	34.465000	31.855000	57.09200	27.28000	34.12900	60.140000	38.345000	30.546000
H	44.541000	34.682000	40.761000	62.54600	28.74200	30.85900	65.977000	35.510000	33.027000
H	43.659000	33.173000	42.087000	63.67500	29.39000	28.77600	67.053000	34.468000	30.894000
H	43.272000	30.539000	41.516000	64.83300	31.61400	28.78000	65.910000	33.840000	29.223000
H	43.890000	29.912000	39.311000	65.25900	33.04300	31.05700	63.823000	34.411000	28.657000
H	45.056000	31.456000	37.643000	64.67400	31.98000	33.12700	62.266000	35.311000	30.948000
H	49.322000	35.252000	38.458000	65.74800	31.62300	36.77300	67.556000	38.339000	35.220000
H	50.921000	36.536000	39.949000	66.42900	33.67800	38.27800	69.476000	36.748000	35.783000
H	50.855000	36.653000	42.069000	64.59600	35.66300	38.10300	69.329000	35.246000	37.990000
H	48.987000	38.313000	42.357000	62.23900	34.96400	38.64700	67.442000	36.537000	39.214000
H	47.501000	38.282000	40.309000	61.63300	32.90800	37.99600	65.065000	36.391000	38.061000
H	50.042000	38.131000	35.529000	59.23100	30.86300	39.58100	65.693000	41.772000	33.675000
H	52.312000	37.692000	34.870000	57.63700	32.44800	40.31500	66.815000	44.053000	33.495000
H	52.800000	36.314000	32.929000	55.91600	33.55400	38.53200	65.884000	46.033000	35.113000
H	50.867000	34.722000	32.106000	55.78200	32.70300	36.27500	63.866000	45.621000	36.273000
H	48.712000	34.682000	32.922000	57.52500	30.71300	35.57800	62.746000	43.432000	36.596000
H	44.276000	40.467000	40.436000	66.45100	26.38900	37.72800	64.242000	36.824000	39.663000
H	45.000000	42.640000	41.313000	67.58700	25.13300	39.74500	64.837000	35.185000	41.409000
H	45.788000	44.526000	39.991000	66.76700	25.46200	42.11800	62.888000	33.369000	42.038000
H	44.303000	43.648000	37.857000	65.03100	27.52300	42.37900	60.571000	33.566000	40.736000
H	45.434000	41.641000	36.434000	63.71200	28.29600	40.22300	60.214000	35.044000	38.835000
H	43.142000	40.279000	32.322000	58.52400	25.09600	38.82000	61.382000	42.773000	37.994000
H	43.061000	42.576000	31.897000	57.97900	24.16700	40.89700	59.596000	44.376000	38.969000
H	44.899000	44.291000	32.435000	59.82300	23.74200	42.43800	57.498000	43.741000	39.771000
H	46.978000	43.274000	33.740000	61.85000	24.71200	42.49600	56.832000	41.365000	39.577000
H	46.930000	41.006000	34.541000	62.55000	26.01600	40.40500	58.490000	39.555000	38.869000
H	41.946000	37.944000	30.905000	61.02300	20.36500	35.72200	57.585000	37.758000	37.514000
H	41.634000	39.671000	31.538000	59.45000	20.06600	35.01800	56.440000	36.535000	38.001000

H	39.301000	38.481000	35.271000	62.95200	22.72300	35.67900	57.198000	36.677000	32.758000
H	39.728000	36.831000	35.474000	63.37500	24.05900	34.74400	56.836000	38.157000	31.991000
H	38.114000	37.385000	34.794000	63.64500	22.40000	34.15000	55.422000	37.196000	32.647000
H	38.135000	36.190000	32.910000	60.64600	23.85600	31.97300	54.794000	40.144000	34.251000
H	39.222000	35.166000	34.066000	61.47500	22.22200	31.77000	55.922000	40.030000	35.701000
H	39.514000	35.388000	32.208000	62.52800	23.63400	32.06900	54.457000	38.961000	35.550000
H	39.260000	39.173000	31.637000	60.75900	20.92800	33.33800	57.229000	36.349000	35.847000
H	39.740000	37.750000	30.703000	59.33000	22.03900	33.62800	55.489000	36.463000	35.737000
H	43.845000	39.483000	29.544000	60.53400	20.78000	39.05800	55.764000	38.440000	40.903000
H	43.318000	40.262000	27.922000	59.72800	19.65300	38.07600	54.359000	39.351000	40.312000
H	43.498000	38.513000	28.104000	58.81900	20.86100	39.26500	56.056000	40.105000	40.199000
H	40.762000	39.469000	27.007000	60.52900	23.53500	38.49400	56.207000	40.861000	38.301000
H	39.417000	39.387000	28.293000	60.16200	23.94400	36.78400	54.481000	40.755000	37.941000
H	40.568000	38.094000	27.976000	61.68000	23.07400	37.21100	55.604000	40.189000	36.772000
H	30.926000	25.875000	39.840000	31.97500	31.78700	37.37500	27.933000	30.282000	48.022000
H	29.550000	24.909000	39.886000	31.64800	30.83500	35.97000	26.569000	30.788000	48.988000
H	29.441000	26.460000	39.246000	32.27000	29.99100	37.43900	26.897000	31.631000	47.534000
H	31.748000	25.483000	41.937000	33.31500	33.31800	36.35100	29.533000	30.743000	50.038000
H	30.425000	24.427000	42.234000	32.95100	32.17800	35.04800	28.234000	31.100000	51.038000
H	30.611000	25.698000	43.357000	34.65000	32.79800	35.27500	29.609000	32.216000	51.014000
H	28.636000	26.268000	41.656000	33.97800	30.23400	35.76900	27.572000	32.822000	49.738000
H	29.628000	27.871000	42.745000	35.84600	31.09300	37.15000	29.671000	33.449000	49.236000
H	31.048000	28.305000	41.747000	34.69300	31.95200	37.96100	30.228000	32.416000	47.984000
H	29.582000	36.113000	42.644000	37.67600	33.09900	39.10800	24.082000	39.052000	50.275000
H	29.056000	34.471000	41.864000	36.69100	31.71200	39.37400	24.508000	38.058000	48.821000
H	30.560000	35.182000	41.365000	38.00500	32.03700	40.34000	25.732000	38.976000	49.803000
H	31.907000	34.124000	44.580000	36.72200	34.75400	42.44400	25.797000	36.976000	52.808000
H	31.518000	35.910000	44.222000	37.40900	35.10800	40.92000	24.267000	37.659000	52.420000
H	32.420000	34.758000	43.131000	38.20600	33.97600	42.14500	25.651000	38.767000	52.219000
H	29.903000	34.278000	44.156000	35.51400	33.66200	40.60600	24.480000	36.475000	50.969000
H	31.523000	33.255000	41.998000	37.07200	32.16400	42.31600	27.240000	37.413000	50.312000
H	31.108000	32.174000	43.247000	35.39200	32.55500	42.90500	27.088000	35.730000	50.560000
H	21.987000	30.005000	41.620000	38.22000	24.84000	39.48500	24.772000	33.636000	45.375000
H	23.446000	30.112000	40.794000	39.81700	25.29500	39.07000	25.247000	32.254000	46.309000
H	23.384000	30.493000	42.730000	39.17400	23.68900	38.45000	23.642000	32.282000	45.468000
H	22.106000	27.806000	43.370000	40.28900	24.53700	36.36200	24.575000	30.272000	43.642000
H	23.297000	28.838000	44.201000	40.58200	26.13200	37.18900	25.971000	29.922000	44.452000
H	23.621000	27.121000	43.905000	39.52100	25.96600	35.68100	25.931000	30.382000	42.803000
H	23.403000	27.864000	41.308000	37.91400	25.00800	36.77900	24.730000	32.427000	43.343000
H	24.778000	29.400000	42.980000	39.02200	27.01500	38.31800	27.255000	31.890000	44.611000
H	25.837000	28.122000	42.649000	38.02000	27.69500	36.97200	27.248000	32.529000	42.926000
H	26.043000	27.005000	37.095000	36.31400	23.54300	39.07000	31.599000	34.480000	43.380000
H	25.662000	27.733000	35.556000	35.39200	23.00300	37.52900	32.309000	35.836000	42.498000
H	24.471000	26.406000	36.251000	36.32100	21.85200	38.53500	32.836000	34.194000	42.058000
H	23.343000	26.140000	38.394000	35.68700	21.49400	41.17700	31.476000	33.374000	40.156000

H	22.894000	27.688000	39.078000	34.22100	22.16300	41.78500	29.933000	34.396000	39.661000
H	24.583000	27.151000	39.140000	35.57900	23.25000	41.26900	29.867000	33.367000	41.135000
H	23.295000	28.155000	36.581000	33.89300	21.85400	39.25900	31.490000	35.854000	40.606000
H	24.508000	29.533000	36.406000	33.44600	23.91200	38.37800	30.878000	36.896000	42.066000
H	23.602000	30.144000	37.814000	32.62000	23.64000	39.72800	29.330000	36.386000	41.179000
H	30.811000	26.055000	35.941000	28.83100	27.50500	37.16700	35.500000	36.527000	47.332000
H	30.224000	27.029000	34.419000	28.40800	29.13000	37.98500	35.073000	35.396000	46.097000
H	29.394000	25.479000	34.712000	27.44100	27.79600	38.34000	35.087000	35.014000	47.829000
H	26.994000	27.913000	35.315000	28.10100	29.14100	40.66500	33.735000	33.618000	47.638000
H	27.641000	26.425000	34.572000	29.24300	30.03400	39.71400	33.707000	33.989000	45.864000
H	28.205000	28.071000	34.126000	29.70500	29.13000	41.14400	32.208000	33.472000	46.759000
H	28.595000	26.514000	36.674000	29.27800	27.06800	39.82900	32.911000	35.662000	48.116000
H	30.217000	27.718000	37.227000	30.77700	27.28800	38.13300	32.817000	37.487000	46.520000
H	30.401000	28.886000	36.058000	30.73500	29.02600	38.14900	32.740000	36.533000	45.190000
H	28.410000	34.378000	35.626000	33.68300	31.04500	44.76600	27.532000	39.701000	49.410000
H	29.682000	34.924000	34.603000	32.98200	31.49800	46.34700	27.227000	41.399000	49.906000
H	29.339000	35.902000	36.093000	32.01800	31.57900	44.83700	28.852000	40.846000	50.085000
H	30.360000	33.162000	34.197000	33.11600	29.03600	47.01600	26.724000	42.115000	47.607000
H	29.537000	32.149000	35.378000	33.70300	28.30300	45.53200	26.875000	40.641000	46.622000
H	31.139000	31.942000	35.184000	32.26800	27.60000	46.33300	27.548000	42.106000	46.152000
H	31.534000	34.448000	35.915000	31.17800	30.03000	45.71000	28.909000	42.282000	48.387000
H	31.474000	33.078000	37.433000	31.24800	28.12700	44.10500	29.656000	40.934000	46.534000
H	31.004000	34.556000	38.199000	31.12300	29.60600	43.23300	30.426000	40.269000	47.971000
H	24.153000	34.061000	45.020000	41.84700	26.83700	43.14100	21.570000	35.138000	44.828000
H	22.769000	34.898000	44.119000	40.96800	27.01900	44.70100	21.802000	35.318000	43.014000
H	24.375000	35.661000	44.159000	41.30200	28.40000	43.91700	21.459000	36.751000	43.871000
H	24.023000	33.887000	40.764000	37.78100	28.00500	44.40000	25.133000	36.562000	42.994000
H	24.156000	35.443000	41.651000	38.94600	29.41200	44.64300	23.552000	37.533000	43.230000
H	22.719000	34.539000	41.858000	39.09200	27.83100	45.55500	23.638000	36.305000	41.975000
H	23.805000	32.856000	43.108000	39.20200	26.42700	43.41900	23.463000	34.539000	43.618000
H	26.169000	34.795000	42.595000	39.46100	28.85000	41.91800	23.530000	37.075000	45.552000
H	26.263000	33.441000	43.826000	39.62400	27.09400	41.47700	23.475000	35.401000	46.081000
H	31.436000	42.215000	35.286000	24.67000	24.14400	48.43100	33.012000	43.457000	45.031000
H	33.439000	41.909000	33.908000	23.62400	22.38700	50.22700	33.974000	42.436000	47.280000
H	32.992000	42.065000	31.540000	24.10500	22.79300	52.25100	32.628000	42.102000	49.208000
H	31.587000	40.729000	30.453000	25.75800	24.80700	53.02500	30.629000	43.828000	49.352000
H	29.511000	41.488000	31.252000	27.04200	25.91600	51.40600	29.767000	45.133000	47.129000
H	23.102000	39.471000	37.153000	31.71000	29.85400	49.01000	31.850000	45.052000	37.665000
H	21.459000	37.261000	36.715000	33.26700	31.25300	49.86900	31.208000	44.192000	35.525000
H	21.640000	36.165000	34.248000	32.52200	33.91200	50.26800	28.908000	43.049000	35.152000
H	22.973000	37.297000	32.518000	30.72600	34.70300	49.12900	27.123000	43.338000	36.869000
H	23.901000	39.339000	32.786000	28.79100	33.11400	48.04800	27.681000	44.744000	39.112000
H	27.438000	42.107000	30.047000	26.00900	28.82700	51.48300	35.296000	44.212000	43.187000
H	27.241000	40.654000	27.645000	24.30700	29.75800	53.21400	36.035000	41.953000	43.779000
H	25.658000	39.184000	27.344000	22.42800	31.04600	52.52100	35.726000	40.156000	41.821000

H	23.629000	38.697000	29.115000	22.38600	32.17500	50.04900	33.729000	40.758000	40.120000
H	23.812000	40.227000	31.318000	24.01700	30.89700	48.28300	32.669000	42.738000	40.182000
H	23.611000	44.636000	30.597000	24.06300	31.93800	45.20900	36.119000	46.938000	40.329000
H	21.644000	46.023000	29.318000	21.61700	32.57300	45.37200	38.215000	48.156000	39.896000
H	21.534000	48.390000	29.622000	19.89700	31.09800	44.95000	38.708000	50.196000	40.818000
H	22.694000	49.383000	31.817000	20.30200	28.67100	44.17200	36.838000	51.612000	42.072000
H	24.876000	47.879000	32.657000	22.82200	27.74500	44.22300	34.851000	50.805000	42.374000
H	21.114000	43.526000	37.343000	26.99800	30.80800	41.95500	30.323000	48.578000	37.244000
H	19.350000	44.954000	38.176000	27.69500	32.33500	40.20100	30.032000	50.344000	35.487000
H	18.629000	46.888000	36.696000	28.77500	34.59500	40.87000	29.643000	52.673000	36.119000
H	19.965000	47.617000	35.085000	29.95500	34.66100	43.49000	30.885000	53.314000	38.227000
H	21.824000	45.866000	34.014000	29.42000	32.76400	44.92900	30.625000	51.763000	40.038000
H	28.877000	47.077000	32.652000	26.24700	24.59800	42.74400	33.969000	47.862000	46.763000
H	30.261000	49.306000	32.569000	24.86500	22.76100	41.57900	35.633000	49.594000	47.817000
H	30.297000	50.727000	34.639000	22.86800	22.31300	42.28100	35.116000	51.808000	48.098000
H	28.833000	50.100000	36.286000	23.13700	22.35400	44.70500	33.294000	52.813000	46.620000
H	28.099000	47.463000	36.785000	23.60800	24.38000	46.09200	31.574000	51.346000	45.418000
H	27.877000	44.338000	39.509000	31.18600	25.68400	44.14800	29.798000	51.849000	43.921000
H	28.838000	46.133000	41.079000	32.07600	23.80400	42.78700	28.264000	53.899000	44.774000
H	27.728000	48.496000	41.045000	31.00500	23.28200	40.64900	25.928000	53.651000	44.041000
H	26.411000	49.126000	39.286000	28.76300	24.39400	40.16000	24.883000	51.780000	43.208000
H	25.049000	47.572000	37.862000	28.12900	26.68500	41.45000	26.179000	49.697000	42.560000
H	27.509000	36.433000	36.763000	32.98900	25.99600	45.80200	26.768000	42.431000	44.980000
H	25.944000	36.990000	36.007000	32.76800	24.26000	45.68000	27.438000	42.411000	43.359000
H	28.532000	40.285000	39.960000	30.72600	22.65700	48.76000	23.942000	46.108000	43.653000
H	28.704000	38.508000	40.149000	30.63300	23.45500	47.25000	25.144000	47.388000	43.324000
H	27.095000	39.379000	40.195000	29.44700	23.96100	48.45300	24.544000	46.886000	45.052000
H	29.203000	38.034000	36.824000	32.74100	25.40700	50.48400	26.341000	45.061000	46.833000
H	30.115000	39.549000	37.438000	31.48600	24.34700	51.05100	27.077000	46.483000	46.207000
H	29.388000	39.576000	35.777000	31.19700	26.06000	50.67800	27.831000	44.959000	45.801000
H	27.344000	38.805000	36.129000	33.77100	23.66200	47.56200	25.185000	43.689000	43.188000
H	25.754000	39.028000	36.989000	33.98200	25.23700	48.22300	25.088000	43.265000	45.075000
H	25.966000	35.874000	40.571000	37.27300	26.00600	44.78500	24.146000	40.539000	45.400000
H	25.722000	37.500000	39.991000	37.30700	24.89600	46.12100	24.060000	39.087000	44.373000
H	27.233000	36.717000	39.708000	36.69400	26.36300	46.44000	23.305000	40.398000	43.826000
H	23.269000	35.698000	37.302000	35.00300	22.44900	45.95500	24.808000	40.018000	41.254000
H	23.559000	37.242000	38.139000	36.06900	22.97100	44.41500	26.393000	40.907000	41.299000
H	23.408000	35.739000	39.067000	34.39200	22.94400	44.32100	24.774000	41.569000	41.726000
H	60.112000	15.818000	43.044000	42.54400	7.58900	61.98400	54.484000	27.246000	47.293000
H	60.284000	17.659000	43.339000	42.29000	9.10400	61.19900	53.150000	27.980000	46.445000
H	59.952000	16.973000	41.662000	43.78100	8.59500	61.67000	54.864000	28.317000	45.847000
H	59.101000	15.233000	44.761000	40.93300	6.68400	60.01000	54.091000	24.991000	47.517000
H	57.332000	15.721000	44.934000	41.63500	6.74000	58.49100	53.732000	24.111000	45.959000
H	58.659000	16.973000	44.812000	41.14700	8.25500	59.25500	52.651000	25.199000	46.508000
H	58.108000	15.731000	42.426000	43.46300	6.33400	59.95900	55.619000	26.232000	45.627000

H	57.695000	18.430000	43.573000	42.82200	8.92100	58.83700	53.102000	26.187000	44.636000
H	58.021000	17.955000	41.933000	44.55700	8.47600	59.45600	54.405000	26.864000	43.517000
H	53.867000	12.486000	39.978000	44.54400	10.82700	57.10000	54.417000	25.673000	39.684000
H	53.798000	11.429000	41.398000	45.08100	12.39800	56.27400	53.909000	27.299000	39.805000
H	54.501000	10.812000	39.885000	44.57900	12.21800	57.98600	54.645000	26.770000	38.352000
H	56.444000	10.394000	41.072000	46.90200	13.38500	58.30100	52.395000	27.147000	37.129000
H	55.685000	10.815000	42.643000	47.75600	13.28900	56.67700	51.695000	27.162000	38.903000
H	57.162000	11.741000	42.057000	48.30900	12.24500	58.02800	50.798000	26.288000	37.586000
H	56.176000	12.470000	40.076000	46.34100	11.09100	58.39400	53.086000	25.037000	37.930000
H	55.181000	12.835000	42.637000	47.11100	11.34900	55.58400	52.067000	25.707000	40.391000
H	56.552000	13.860000	42.094000	48.31800	10.28000	56.53700	51.322000	24.263000	39.713000
H	57.224000	18.151000	38.036000	40.28700	10.23600	55.49900	52.887000	18.622000	45.354000
H	56.458000	17.923000	36.453000	40.10500	11.51700	54.23400	53.465000	18.274000	46.922000
H	55.585000	17.636000	37.881000	41.60400	10.61100	54.43600	54.423000	19.401000	45.783000
H	56.934000	20.282000	35.665000	37.91800	10.41800	53.70800	51.525000	19.024000	48.153000
H	58.014000	20.490000	37.059000	37.81700	9.05200	54.84400	50.804000	19.452000	46.670000
H	56.690000	21.598000	36.943000	38.06300	8.65000	53.23800	50.818000	20.613000	47.884000
H	54.982000	19.812000	36.926000	40.25400	9.46100	52.76000	53.522000	20.430000	47.587000
H	56.597000	20.121000	39.502000	40.28000	8.16900	55.48800	51.368000	21.059000	45.298000
H	55.050000	21.015000	39.071000	40.08400	7.36200	53.96800	51.881000	22.170000	46.565000
H	52.518000	22.847000	44.177000	45.18500	2.60800	50.47200	59.392000	20.517000	46.849000
H	53.048000	21.344000	43.636000	45.16700	3.81900	51.62000	58.238000	21.406000	45.743000
H	51.332000	21.706000	43.799000	44.05700	4.09300	50.33400	59.344000	20.506000	45.087000
H	52.516000	23.034000	40.251000	42.94700	1.39000	53.58500	57.050000	18.096000	46.834000
H	53.888000	22.148000	40.703000	44.46900	2.15100	53.34600	56.255000	19.377000	47.714000
H	53.679000	23.738000	41.406000	44.13200	0.67700	52.37200	57.853000	18.862000	48.090000
H	51.315000	23.110000	42.029000	42.78100	2.24700	51.21200	58.552000	18.402000	46.020000
H	50.628000	20.779000	42.061000	42.41800	4.38400	51.64300	57.856000	18.961000	44.099000
H	50.905000	21.265000	40.435000	41.64300	3.73500	53.05600	56.464000	18.059000	44.721000
H	54.354000	19.687000	48.591000	45.38300	1.27800	59.59800	59.970000	26.514000	44.672000
H	52.953000	18.826000	47.965000	46.30200	1.87800	58.27200	60.603000	25.735000	43.330000
H	53.333000	20.462000	47.327000	45.01100	0.68500	57.99900	60.584000	24.863000	44.868000
H	55.108000	16.891000	45.859000	45.05700	3.05900	60.53600	56.972000	25.612000	42.812000
H	53.913000	16.874000	47.156000	44.78200	4.78200	59.98000	58.502000	26.010000	41.967000
H	55.522000	17.492000	47.416000	46.38100	3.68600	59.71500	57.873000	26.954000	43.355000
H	54.858000	19.678000	46.072000	43.67200	3.01600	58.92600	58.027000	24.942000	44.718000
H	52.420000	18.112000	45.794000	45.92600	2.92900	57.04500	58.880000	23.764000	42.263000
H	52.688000	19.835000	45.212000	44.34900	2.73400	56.43700	59.164000	22.927000	43.896000
H	50.752000	16.658000	45.664000	50.36400	7.97000	56.89500	55.216000	23.959000	36.631000
H	48.935000	16.870000	45.586000	50.65100	6.53000	57.75100	56.133000	25.394000	36.658000
H	49.837000	16.906000	44.068000	48.97200	7.03000	57.48800	55.549000	24.859000	38.259000
H	48.312000	15.788000	43.117000	48.32000	4.70100	57.08300	57.479000	24.314000	39.146000
H	47.527000	15.254000	44.587000	49.89800	4.58000	57.82100	58.628000	25.105000	38.018000
H	48.206000	14.074000	43.363000	49.60000	3.74300	56.25000	58.719000	23.273000	38.451000
H	49.492000	14.343000	45.522000	51.01200	5.47500	55.97200	57.339000	23.336000	36.574000

H	51.584000	14.696000	44.878000	49.50500	6.69600	54.37300	55.872000	21.960000	37.616000
H	50.911000	13.441000	43.751000	49.20300	4.95400	54.34200	57.316000	21.410000	38.483000
H	56.135000	14.597000	38.438000	42.05600	9.39000	51.69100	50.023000	23.408000	42.493000
H	57.317000	13.995000	37.293000	41.74800	9.82000	49.97900	48.365000	22.961000	42.729000
H	55.756000	13.196000	37.367000	41.95900	11.13100	50.98900	49.029000	22.900000	41.092000
H	54.632000	15.853000	34.909000	45.23800	11.04300	49.42900	47.716000	19.850000	42.790000
H	54.763000	14.059000	34.968000	44.13100	12.37800	49.47400	47.499000	20.885000	41.401000
H	56.211000	14.896000	34.761000	43.73100	10.97100	48.44900	47.563000	21.535000	42.978000
H	56.017000	16.127000	36.773000	44.06000	9.34900	50.44900	49.478000	21.209000	43.375000
H	53.840000	16.020000	36.819000	45.43300	10.48100	51.27800	50.480000	19.605000	42.211000
H	53.324000	14.494000	37.439000	44.58600	11.55000	52.42300	50.381000	20.308000	40.693000
H	50.162000	24.746000	31.147000	51.70000	7.90000	43.92100	53.979000	7.996000	36.451000
H	51.878000	26.719000	31.108000	51.48800	10.63000	44.63500	51.710000	7.555000	34.931000
H	53.356000	27.260000	33.253000	50.69400	11.21300	46.79900	50.137000	9.358000	34.647000
H	52.806000	26.060000	35.412000	50.88300	9.26000	48.64300	50.387000	11.384000	35.994000
H	50.904000	24.118000	35.313000	51.02700	6.74300	47.75100	52.414000	12.026000	37.243000
H	45.296000	18.099000	30.007000	54.38300	-0.68200	48.11000	58.672000	12.128000	34.745000
H	45.711000	16.255000	28.900000	55.72500	-0.93200	50.22200	59.841000	12.315000	32.654000
H	47.845000	15.047000	29.151000	57.40700	1.06000	51.05200	62.783000	11.922000	32.932000
H	50.182000	16.667000	29.488000	57.84500	2.91300	49.51600	63.602000	11.113000	35.116000
H	49.784000	18.139000	31.159000	56.74200	2.94600	47.15600	62.129000	11.159000	37.245000
H	46.869000	21.577000	27.657000	57.06900	4.10600	46.33200	54.951000	7.263000	36.326000
H	46.619000	22.719000	25.750000	59.26900	5.41800	45.96200	54.191000	5.806000	34.650000
H	48.463000	24.789000	25.215000	59.50500	6.95300	44.07700	55.804000	4.431000	33.328000
H	49.886000	25.431000	27.259000	57.76300	7.56200	42.52300	58.323000	4.340000	34.073000
H	49.384000	24.404000	29.483000	55.59000	6.70800	42.79900	59.043000	6.366000	35.591000
H	41.678000	22.949000	31.264000	55.55600	3.88400	41.22600	59.200000	6.680000	42.906000
H	39.822000	24.403000	30.214000	56.86600	3.42200	38.95800	60.832000	4.596000	43.099000
H	40.701000	26.308000	28.799000	55.82400	2.29900	37.11000	61.259000	3.275000	41.303000
H	42.821000	27.309000	28.895000	53.75800	1.06200	37.47700	60.526000	4.012000	38.667000
H	44.429000	26.102000	29.923000	52.42600	1.22100	39.78600	59.010000	5.797000	38.441000
H	43.431000	17.274000	33.297000	51.39300	-2.56300	43.99900	62.462000	12.776000	41.094000
H	41.332000	16.302000	33.895000	52.16900	-4.96800	44.48400	64.259000	12.759000	42.513000
H	39.201000	17.183000	32.855000	54.74400	-5.41300	44.26300	64.631000	10.664000	43.841000
H	39.103000	19.703000	32.326000	56.34000	-3.48400	44.27000	63.289000	8.702000	43.447000
H	41.423000	21.062000	31.997000	55.41200	-1.15400	43.71000	61.347000	8.672000	41.894000
H	46.544000	25.372000	37.027000	49.58200	6.12200	41.58200	52.756000	9.971000	41.481000
H	46.769000	27.644000	37.912000	48.12800	6.26500	39.31900	51.032000	8.177000	42.112000
H	44.732000	29.279000	36.649000	46.37000	4.14600	39.27000	50.993000	9.070000	44.677000
H	43.773000	28.626000	34.617000	46.74400	2.08400	40.04900	53.255000	8.664000	45.574000
H	44.195000	26.432000	33.668000	48.00100	2.01800	42.18200	55.266000	9.529000	44.311000
H	42.418000	22.059000	36.861000	47.70700	0.12900	43.33200	55.986000	11.963000	44.057000
H	42.051000	22.979000	38.871000	45.80000	-1.00600	43.91400	56.227000	13.259000	46.288000
H	43.918000	22.739000	41.177000	45.53700	-1.93600	46.41700	57.300000	15.483000	46.009000
H	46.065000	21.444000	40.366000	47.39300	-1.12900	48.31600	58.425000	16.268000	44.186000

H	46.204000	20.614000	37.811000	49.05300	0.25900	47.78100	58.645000	14.730000	42.187000
H	46.221000	17.631000	38.509000	48.41300	7.56800	48.55200	57.068000	16.245000	41.018000
H	47.579000	18.702000	39.145000	48.88700	6.72500	50.03400	56.073000	14.901000	40.401000
H	50.430000	20.101000	38.927000	50.86000	5.74100	51.23800	53.919000	15.270000	36.106000
H	48.897000	21.010000	38.897000	50.48400	4.07200	51.79600	53.807000	16.874000	36.897000
H	50.306000	21.489000	37.791000	49.19800	5.29200	51.63600	53.321000	15.423000	37.853000
H	50.879000	18.138000	35.743000	47.52700	3.75700	50.14800	57.723000	15.587000	36.269000
H	50.630000	17.631000	37.389000	48.74500	2.50500	50.79700	57.042000	17.238000	36.310000
H	51.685000	18.900000	37.036000	48.25100	2.43800	48.93900	56.180000	15.914000	35.303000
H	47.358000	18.014000	36.535000	49.26900	5.29400	47.47700	56.381000	17.278000	38.927000
H	46.849000	19.456000	37.356000	50.36300	6.11700	48.64200	57.650000	16.003000	38.904000
H	46.518000	15.218000	40.523000	45.09800	8.32000	49.97400	53.022000	15.740000	39.726000
H	47.224000	16.455000	41.479000	46.14200	8.72100	48.45200	52.244000	16.698000	41.043000
H	48.184000	15.083000	41.257000	46.78300	8.84100	50.06500	52.819000	15.208000	41.487000
H	48.370000	14.053000	38.500000	44.70300	6.75600	47.70600	54.030000	16.799000	43.456000
H	48.924000	15.295000	37.281000	44.30600	6.46500	49.38300	55.561000	17.572000	43.306000
H	47.240000	14.882000	37.615000	45.01100	5.08100	48.54800	55.555000	15.741000	43.483000
H	50.871000	46.467000	44.417000	8.83400	46.25900	31.27800	-2.128000	53.597000	45.686000
H	51.006000	48.229000	44.393000	10.41200	46.23700	32.05000	-1.375000	53.103000	47.226000
H	50.431000	47.474000	43.020000	10.33300	46.11600	30.19100	-1.612000	54.855000	46.549000
H	52.870000	45.706000	41.419000	10.97700	42.22700	30.83500	1.617000	54.906000	45.886000
H	51.347000	46.249000	41.462000	11.80400	43.55000	31.71000	1.337000	54.024000	47.462000
H	51.833000	44.916000	42.568000	11.45900	43.72200	30.06700	0.717000	55.713000	47.265000
H	53.261000	46.767000	43.621000	9.14200	44.13100	30.45100	-0.129000	54.776000	44.993000
H	53.414000	47.921000	41.727000	8.44900	44.58300	32.89600	-0.162000	52.709000	44.408000
H	51.939000	48.823000	41.611000	8.71000	42.77200	32.52100	1.593000	53.060000	44.567000
H	57.232000	44.432000	43.488000	14.71700	47.89900	31.70700	-5.300000	49.700000	49.834000
H	58.049000	44.982000	44.872000	13.70200	48.81100	32.89200	-5.557000	49.549000	48.054000
H	58.165000	46.015000	43.361000	12.98300	48.06000	31.51700	-4.970000	50.923000	48.618000
H	57.171000	46.965000	41.892000	12.54300	45.22600	32.01200	-3.194000	51.351000	49.973000
H	55.522000	47.250000	42.393000	13.70300	44.64300	33.12000	-2.027000	50.235000	50.309000
H	55.899000	45.470000	41.845000	14.05800	45.38700	31.58200	-3.543000	50.048000	51.297000
H	55.674000	45.899000	44.334000	14.24400	46.84300	34.09200	-3.522000	48.652000	49.341000
H	57.310000	47.278000	45.476000	12.23200	48.00400	34.47700	-3.321000	49.480000	46.933000
H	57.876000	48.163000	43.903000	11.44200	46.99600	33.27300	-2.733000	50.912000	46.964000
H	47.724000	49.200000	47.354000	13.24400	37.58000	35.50100	5.467000	53.700000	49.861000
H	47.685000	50.272000	48.729000	14.66100	37.59500	34.33800	6.134000	54.179000	48.259000
H	49.037000	49.022000	48.547000	13.17900	37.74500	33.56900	6.699000	52.656000	49.181000
H	49.813000	52.466000	46.457000	14.96000	40.81700	35.89700	3.085000	53.151000	47.220000
H	48.386000	52.614000	46.884000	15.61400	39.26500	36.20700	3.936000	54.718000	47.370000
H	48.578000	51.308000	45.722000	13.88100	39.59800	36.49100	2.778000	54.100000	48.608000
H	49.986000	50.959000	48.391000	14.70800	39.51600	33.74000	5.203000	52.459000	47.003000
H	50.761000	49.138000	47.520000	12.13100	40.22300	33.54100	4.959000	50.843000	48.107000
H	50.008000	49.184000	45.905000	12.03400	39.39700	35.19900	4.100000	51.462000	49.594000
H	53.762000	55.353000	45.179000	8.49300	39.52800	37.35100	4.363000	45.504000	45.595000

H	53.234000	55.787000	46.876000	7.83300	39.60600	38.98500	5.967000	45.402000	46.597000
H	53.781000	57.006000	45.680000	7.73300	38.09700	38.07800	5.573000	44.277000	45.184000
H	50.643000	56.319000	43.777000	11.61200	37.73800	38.37200	5.569000	47.247000	42.867000
H	52.287000	55.783000	43.349000	11.04900	38.30100	36.89600	4.337000	46.282000	43.675000
H	51.863000	57.492000	43.889000	10.20600	36.96300	37.56300	5.825000	45.488000	42.903000
H	51.172000	56.645000	45.971000	9.68400	38.10200	39.58500	6.961000	46.403000	44.651000
H	51.411000	54.641000	46.735000	9.77300	40.54600	39.73800	6.054000	47.404000	46.314000
H	50.539000	54.211000	45.320000	11.48200	40.24700	39.46200	6.127000	48.665000	45.132000
H	51.594000	52.482000	41.619000	6.49200	40.04500	36.28200	1.722000	46.113000	42.952000
H	51.549000	51.942000	39.891000	7.91200	40.96100	36.20100	3.248000	46.383000	42.166000
H	50.592000	53.439000	40.476000	6.64000	41.56000	35.23500	2.068000	45.291000	41.441000
H	51.518000	55.005000	39.684000	4.65000	40.69700	37.53300	1.977000	46.972000	39.089000
H	52.529000	54.227000	38.454000	4.13200	41.91900	36.29200	3.451000	47.607000	40.014000
H	53.179000	55.457000	39.574000	4.33800	42.27100	38.13500	2.199000	48.674000	39.365000
H	52.554000	54.700000	41.497000	6.14900	41.84300	38.36100	0.720000	47.347000	40.966000
H	53.532000	52.271000	40.352000	6.26900	43.37400	36.13600	2.888000	48.685000	42.022000
H	54.806000	53.344000	40.744000	6.46800	44.09500	37.71500	1.580000	49.770000	41.802000
H	59.251000	52.258000	46.415000	9.55200	48.56000	36.31400	-2.868000	47.597000	45.116000
H	59.082000	53.774000	45.903000	10.12500	49.79200	37.41400	-3.486000	46.129000	46.019000
H	60.695000	53.035000	45.992000	8.53400	49.88400	36.80500	-4.484000	46.713000	44.732000
H	59.536000	50.301000	44.449000	7.82800	46.86600	37.19500	-1.830000	46.998000	42.429000
H	61.255000	50.815000	44.580000	6.63800	48.12700	37.29400	-3.212000	45.984000	42.369000
H	60.571000	50.933000	43.012000	7.15600	46.77900	38.59600	-1.539000	45.293000	42.414000
H	60.179000	53.214000	43.680000	8.34400	49.17600	39.08400	-3.017000	44.871000	44.616000
H	58.349000	53.561000	43.663000	10.59700	47.90900	38.93700	-1.448000	45.284000	46.017000
H	58.535000	52.407000	42.369000	9.24100	47.06600	39.90000	-0.471000	44.806000	44.738000
H	57.893000	47.899000	50.062000	16.57800	41.69000	35.57600	-1.651000	48.779000	51.122000
H	57.705000	48.758000	48.490000	15.40300	42.24200	34.45200	-1.002000	47.392000	50.197000
H	58.420000	49.489000	49.745000	17.08900	42.47000	33.99200	-1.842000	47.192000	51.775000
H	57.681000	51.433000	50.415000	17.04200	44.89000	33.86600	0.171000	45.772000	52.781000
H	55.903000	51.459000	50.553000	16.49500	45.78000	35.32200	1.728000	46.620000	52.718000
H	56.821000	51.445000	48.961000	15.35600	45.18500	33.88900	1.226000	45.912000	51.205000
H	56.459000	49.832000	51.057000	17.44200	44.06500	35.79000	0.132000	48.385000	52.783000
H	54.932000	48.433000	49.414000	15.85600	43.08200	37.12100	0.413000	49.773000	51.259000
H	54.221000	50.018000	49.577000	15.63400	44.88400	37.30400	2.057000	48.896000	51.605000
H	52.473000	62.642000	47.774000	18.96300	45.87600	48.95500	5.190000	39.468000	45.830000
H	54.569000	63.199000	47.309000	16.28500	46.28600	50.03500	4.596000	38.245000	43.672000
H	56.253000	61.145000	47.070000	15.06100	44.17000	50.76900	4.285000	35.542000	43.554000
H	55.535000	59.555000	45.757000	15.98700	41.84300	49.93500	5.468000	34.185000	45.228000
H	53.450000	60.348000	45.407000	17.75900	41.62700	48.59200	6.792000	35.430000	47.170000
H	50.445000	54.187000	48.984000	22.96900	40.58000	41.41700	10.560000	44.334000	49.898000
H	51.394000	52.209000	48.748000	22.92400	38.40900	40.23300	11.074000	46.776000	48.873000
H	52.743000	50.957000	50.649000	20.75400	36.60300	40.62900	9.097000	47.906000	47.899000
H	53.308000	52.578000	52.598000	19.17000	37.37500	42.56300	7.420000	46.626000	46.564000
H	52.129000	54.712000	52.975000	19.42600	39.67500	43.59700	7.079000	44.424000	47.351000

H	49.586000	58.482000	46.146000	21.07100	41.45400	49.24900	9.039000	38.641000	44.455000
H	48.247000	57.809000	43.944000	22.08600	40.60200	51.41100	9.587000	38.751000	42.232000
H	47.004000	55.727000	44.130000	24.20800	39.06300	51.11400	11.116000	40.599000	41.492000
H	46.407000	54.634000	46.194000	25.57200	39.16700	48.68500	11.084000	42.033000	43.120000
H	47.470000	55.441000	48.243000	24.33100	40.24700	46.67200	11.683000	41.758000	45.700000
H	45.393000	59.853000	49.379000	26.14100	43.29600	46.99000	14.035000	39.065000	49.945000
H	43.064000	60.312000	50.006000	27.52800	42.87200	45.14500	16.346000	38.253000	49.766000
H	42.698000	62.184000	51.625000	27.61500	44.76100	43.25700	16.618000	35.942000	48.167000
H	44.776000	62.708000	53.335000	26.67400	46.98300	43.57600	14.744000	35.270000	46.961000
H	46.541000	61.467000	53.468000	25.15700	47.28100	45.50600	12.317000	36.540000	46.956000
H	48.580000	59.616000	55.964000	22.91500	45.57800	40.39600	12.378000	40.389000	53.259000
H	46.328000	58.479000	57.178000	24.22100	45.28600	38.13300	13.695000	41.607000	54.543000
H	45.623000	56.056000	56.758000	26.56600	44.31900	38.43600	14.608000	44.017000	53.754000
H	47.369000	54.643000	55.353000	27.11200	42.97400	40.98100	13.114000	45.327000	51.905000
H	49.442000	55.735000	54.329000	25.61300	42.87400	42.56800	11.286000	43.924000	50.840000
H	51.479000	63.753000	52.406000	18.99700	48.79000	46.86300	11.366000	35.157000	49.542000
H	51.057000	66.135000	52.824000	19.17300	50.44400	48.77900	11.885000	32.874000	49.786000
H	49.891000	67.518000	50.917000	20.90500	51.21400	49.71800	10.294000	31.496000	51.594000
H	49.236000	66.392000	48.786000	23.27000	50.00400	49.15100	7.799000	32.266000	51.608000
H	49.274000	63.597000	48.777000	23.22900	48.42600	47.22100	7.456000	34.980000	50.953000
H	55.019000	60.778000	53.994000	18.31900	47.72700	43.12000	8.943000	40.875000	53.977000
H	55.919000	62.899000	55.309000	17.01100	49.19500	41.35500	7.514000	41.678000	56.260000
H	54.688000	64.513000	56.416000	18.04300	49.60400	39.22200	5.720000	40.513000	57.062000
H	51.940000	63.909000	56.427000	20.50600	48.47300	38.62200	5.202000	38.092000	55.866000
H	51.104000	62.604000	54.788000	21.41600	46.84600	40.39400	5.979000	37.690000	53.402000
H	56.125000	53.701000	50.171000	13.88000	46.30400	41.46200	2.662000	45.138000	50.824000
H	57.494000	54.869000	50.059000	13.57800	45.92800	43.20900	2.077000	43.437000	50.278000
H	56.258000	57.993000	53.907000	15.70300	43.27000	46.17300	5.380000	42.636000	53.230000
H	54.794000	57.231000	53.609000	14.11900	43.70000	45.44300	5.564000	40.851000	52.807000
H	55.009000	58.799000	52.974000	15.33700	44.95100	45.70100	4.265000	41.466000	53.769000
H	58.252000	58.058000	50.750000	15.51300	41.24400	42.86700	2.212000	39.961000	52.311000
H	57.095000	59.287000	51.025000	14.05900	42.11600	42.98200	3.230000	39.367000	50.893000
H	56.980000	58.327000	49.488000	14.64000	41.62800	44.54000	1.731000	40.453000	50.645000
H	54.972000	55.295000	51.730000	15.33500	44.41500	41.41400	4.632000	43.748000	51.588000
H	56.871000	55.104000	51.960000	16.13300	45.63900	42.62800	3.340000	43.432000	52.662000
H	53.689000	56.000000	48.833000	12.76300	42.69000	43.57800	5.195000	45.855000	47.954000
H	53.561000	54.406000	49.612000	11.16900	42.68200	42.85100	6.071000	44.621000	48.975000
H	53.283000	54.589000	47.779000	11.58300	44.00500	44.00800	5.163000	46.075000	49.666000
H	57.055000	56.297000	47.006000	12.16900	46.76100	40.97000	3.103000	42.360000	47.587000
H	56.168000	57.338000	48.218000	12.09400	46.66000	42.82700	4.839000	42.219000	48.225000
H	55.264000	56.682000	46.772000	10.69700	46.19700	41.80700	4.435000	43.377000	46.794000

Table S7. Atomic coordinates of systems consisted of four molecules of Ph_7T_8-T_8iBu_7 compound for clusters 7 and 8.

Atomic coordinates of systems consisted of four molecules of Ph_7T_8-T_8iBu_7 compound						
	Cluster **7**			Cluster **8**		
Atom	x	y	z	x	y	z
Si	32.703000	33.740000	36.861000	41.384000	42.182000	41.577000
Si	30.607000	35.803000	33.040000	38.528000	41.403000	43.985000
Si	29.073000	37.846000	34.736000	36.685000	42.224000	46.298000
Si	28.450000	40.697000	34.492000	34.326000	43.865000	47.305000
Si	25.928000	40.384000	36.263000	33.556000	41.863000	49.363000
Si	27.578000	40.904000	38.645000	35.435000	43.186000	51.339000
Si	29.968000	41.295000	37.016000	36.270000	45.195000	49.305000
Si	26.755000	37.462000	36.546000	35.898000	40.227000	48.394000
Si	28.304000	38.092000	39.066000	37.672000	41.519000	50.443000
Si	30.671000	38.350000	37.208000	38.529000	43.493000	48.428000
Si	16.656000	25.187000	38.935000	15.978000	30.956000	47.823000
Si	19.866000	27.496000	39.669000	12.215000	32.418000	48.711000
Si	20.585000	30.345000	39.878000	13.657000	35.075000	48.576000
Si	23.047000	31.993000	39.915000	15.670000	36.730000	46.911000
Si	21.690000	34.287000	39.010000	14.124000	39.115000	47.841000
Si	21.174000	35.043000	41.927000	16.272000	39.523000	50.007000
Si	22.587000	32.585000	42.906000	17.818000	37.090000	49.030000
Si	19.129000	32.730000	39.047000	12.435000	37.500000	49.783000
Si	18.533000	33.475000	41.982000	14.751000	37.798000	51.786000
Si	19.960000	31.087000	42.756000	15.902000	35.248000	50.490000
Si	36.297000	23.146000	30.869000	35.197000	23.970000	43.381000
Si	36.167000	21.339000	34.160000	33.575000	20.566000	43.315000
Si	39.201000	21.783000	34.009000	36.407000	20.113000	42.372000
Si	40.186000	24.585000	34.324000	38.988000	20.651000	43.841000
Si	41.410000	23.722000	36.907000	39.035000	17.670000	44.600000
Si	43.858000	23.170000	35.154000	40.663000	17.124000	42.214000
Si	42.536000	23.949000	32.631000	40.332000	19.896000	41.281000
Si	40.628000	20.938000	36.604000	36.447000	17.223000	43.300000
Si	43.000000	20.339000	34.842000	38.183000	16.556000	40.816000
Si	41.613000	21.251000	32.304000	37.765000	19.419000	39.838000
Si	51.516000	53.265000	52.518000	50.905000	52.501000	52.373000
Si	54.522000	51.239000	50.786000	47.845000	53.378000	49.875000
Si	56.757000	49.503000	50.020000	46.707000	55.141000	47.697000
Si	55.172000	47.403000	48.392000	45.355000	53.225000	45.790000
Si	57.565000	47.023000	46.561000	42.894000	54.984000	45.869000
Si	58.872000	45.193000	48.320000	43.734000	56.737000	43.613000
Si	56.483000	45.511000	50.175000	46.234000	55.113000	43.569000
Si	59.098000	49.158000	48.139000	44.156000	56.847000	47.812000
Si	60.218000	47.198000	50.119000	44.980000	58.706000	45.625000
Si	57.913000	47.610000	51.927000	47.606000	57.040000	45.600000

Si	35.502000	34.678000	36.572000	42.090000	42.659000	38.690000
Si	36.936000	34.994000	39.073000	44.144000	43.972000	36.836000
Si	39.478000	34.050000	37.710000	43.160000	42.513000	34.438000
Si	40.189000	36.953000	37.152000	41.070000	44.611000	33.948000
Si	37.552000	37.857000	38.460000	42.164000	46.136000	36.315000
Si	37.958000	33.675000	35.087000	41.120000	41.166000	36.266000
Si	38.740000	36.604000	34.507000	39.021000	43.111000	35.470000
Si	36.111000	37.523000	35.847000	40.089000	44.659000	37.795000
Si	14.683000	26.156000	37.035000	15.048000	28.059000	47.238000
Si	13.313000	28.657000	36.763000	13.086000	26.242000	48.513000
Si	14.968000	29.650000	34.411000	11.274000	26.482000	46.088000
Si	12.932000	28.293000	32.713000	12.512000	24.049000	45.061000
Si	11.261000	27.395000	35.067000	14.402000	23.836000	47.340000
Si	16.370000	26.954000	34.603000	13.346000	28.071000	44.704000
Si	14.336000	25.535000	32.816000	14.594000	25.565000	43.617000
Si	12.725000	24.848000	35.207000	16.436000	25.738000	45.954000
Si	34.692000	24.158000	28.521000	33.891000	26.537000	44.376000
Si	34.347000	26.751000	27.101000	34.662000	28.908000	46.102000
Si	33.658000	25.440000	24.533000	33.051000	30.894000	44.611000
Si	30.738000	25.316000	25.233000	30.772000	30.222000	46.499000
Si	31.313000	26.650000	27.745000	32.495000	28.218000	48.087000
Si	33.803000	22.811000	25.946000	32.065000	28.470000	42.990000
Si	30.961000	22.719000	26.734000	29.741000	27.918000	44.779000
Si	31.708000	24.100000	29.291000	31.629000	25.995000	46.340000
Si	49.940000	51.793000	50.392000	49.003000	50.169000	53.134000
Si	48.990000	51.800000	47.578000	46.672000	49.532000	54.980000
Si	48.636000	48.762000	47.679000	45.491000	47.533000	53.099000
Si	45.848000	49.284000	48.635000	47.095000	45.480000	54.693000
Si	46.106000	52.365000	48.533000	48.310000	47.421000	56.563000
Si	49.495000	48.896000	50.617000	47.886000	48.072000	51.256000
Si	46.690000	49.340000	51.533000	49.418000	45.923000	52.787000
Si	47.080000	52.281000	51.283000	50.516000	48.024000	54.699000
O	34.169000	33.909000	36.152000	42.347000	42.183000	40.246000
O	36.685000	33.627000	36.147000	42.033000	41.433000	37.571000
O	35.665000	35.178000	38.075000	43.267000	43.736000	38.249000
O	35.691000	35.994000	35.656000	40.613000	43.241000	38.474000
O	39.209000	33.839000	36.153000	41.854000	41.552000	34.838000
O	36.291000	37.703000	37.445000	41.407000	45.637000	37.628000
O	39.991000	36.416000	35.589000	40.035000	43.434000	34.262000
O	38.014000	33.886000	38.459000	43.942000	42.706000	35.821000
O	37.603000	36.485000	39.320000	43.496000	45.251000	36.032000
O	39.944000	35.589000	37.998000	42.532000	43.892000	33.848000
O	38.869000	37.813000	37.557000	41.132000	45.648000	35.135000
O	38.026000	35.152000	34.395000	39.605000	41.861000	36.342000
O	37.568000	37.533000	35.214000	39.130000	44.443000	36.443000

O	29.519000	36.947000	33.430000	37.131000	41.853000	44.799000
O	27.535000	37.580000	35.106000	35.795000	41.043000	46.976000
O	29.407000	39.378000	34.358000	35.699000	43.514000	46.467000
O	29.904000	37.471000	36.086000	37.895000	42.433000	47.376000
O	25.923000	38.850000	36.857000	34.659000	40.691000	49.318000
O	30.760000	39.870000	36.714000	37.465000	44.744000	48.262000
O	27.319000	39.405000	39.185000	36.265000	41.790000	51.253000
O	26.947000	40.245000	34.984000	33.650000	42.496000	47.876000
O	29.041000	41.593000	35.701000	34.899000	44.750000	48.565000
O	26.832000	41.254000	37.291000	34.123000	42.951000	50.444000
O	29.163000	41.185000	38.417000	36.428000	44.376000	50.737000
O	27.797000	37.242000	37.751000	37.292000	40.601000	49.128000
O	29.843000	38.408000	38.582000	38.371000	42.906000	49.992000
O	15.143000	25.343000	38.316000	15.942000	29.310000	47.789000
O	15.831000	26.936000	36.187000	13.683000	28.384000	46.277000
O	13.478000	27.177000	37.337000	14.469000	26.988000	48.240000
O	14.135000	24.974000	36.039000	16.131000	27.304000	46.147000
O	16.018000	28.428000	34.083000	11.945000	27.247000	44.804000
O	11.735000	26.039000	35.710000	15.655000	24.849000	47.100000
O	13.982000	27.080000	32.373000	13.055000	25.016000	43.890000
O	14.631000	29.260000	35.979000	11.983000	26.938000	47.504000
O	12.032000	28.573000	35.713000	13.242000	24.685000	48.108000
O	13.543000	29.532000	33.591000	11.501000	24.900000	46.050000
O	11.757000	27.429000	33.490000	13.822000	23.523000	45.881000
O	15.527000	25.739000	33.896000	14.382000	27.091000	44.006000
O	13.025000	24.892000	33.591000	15.707000	25.051000	44.693000
O	20.690000	28.801000	39.328000	12.787000	33.796000	48.072000
O	20.149000	31.488000	38.853000	12.653000	36.337000	48.667000
O	22.064000	30.705000	40.280000	14.946000	35.494000	47.666000
O	19.749000	30.572000	41.280000	14.379000	34.824000	50.031000
O	20.078000	34.083000	39.050000	13.134000	38.810000	49.127000
O	21.628000	31.222000	42.940000	17.125000	35.652000	49.363000
O	19.639000	34.618000	41.688000	15.166000	39.246000	51.149000
O	22.383000	32.857000	38.751000	14.443000	37.751000	47.017000
O	23.182000	32.687000	41.413000	16.976000	37.405000	47.667000
O	21.899000	34.871000	40.530000	15.633000	39.421000	48.515000
O	21.607000	33.917000	43.034000	17.408000	38.308000	50.078000
O	18.402000	32.719000	40.516000	13.425000	37.267000	50.987000
O	19.233000	32.497000	43.083000	15.741000	36.579000	51.372000
O	35.984000	23.827000	29.454000	34.972000	25.556000	43.708000
O	34.760000	23.617000	26.963000	33.471000	27.700000	43.290000
O	34.467000	25.772000	28.411000	34.482000	27.327000	45.642000
O	33.241000	23.597000	29.066000	32.515000	25.911000	44.967000
O	33.383000	23.801000	24.739000	32.200000	30.013000	43.525000
O	31.756000	25.721000	29.046000	32.539000	26.743000	47.462000

O	30.821000	23.731000	25.463000	29.974000	29.403000	45.351000
O	34.651000	25.950000	25.701000	34.321000	29.925000	44.813000
O	32.748000	27.127000	27.047000	33.616000	29.070000	47.390000
O	32.170000	25.939000	24.737000	32.120000	30.935000	45.978000
O	30.413000	25.923000	26.656000	31.170000	28.999000	47.565000
O	32.506000	22.256000	26.785000	30.813000	27.640000	43.559000
O	30.673000	23.517000	28.090000	30.273000	26.966000	46.041000
O	37.641000	21.605000	33.572000	34.937000	20.789000	42.441000
O	39.536000	21.695000	35.631000	36.161000	18.822000	43.381000
O	39.406000	23.381000	33.591000	37.693000	20.930000	42.857000
O	40.287000	20.924000	33.224000	36.594000	19.430000	40.941000
O	41.498000	22.160000	37.196000	37.815000	16.738000	44.031000
O	41.570000	22.830000	31.983000	38.956000	20.389000	40.456000
O	43.546000	21.632000	35.571000	39.365000	16.224000	41.848000
O	40.266000	24.164000	35.897000	38.692000	19.271000	44.698000
O	41.653000	24.796000	33.704000	40.218000	20.234000	42.829000
O	42.756000	23.972000	36.037000	40.402000	17.673000	43.743000
O	43.752000	23.318000	33.532000	40.765000	18.336000	41.156000
O	41.573000	20.056000	35.632000	36.772000	16.775000	41.689000
O	42.853000	20.877000	33.326000	38.331000	17.872000	39.838000
O	51.176000	52.493000	51.144000	49.538000	51.652000	52.721000
O	50.335000	50.261000	50.250000	48.011000	49.511000	51.998000
O	49.340000	52.521000	48.979000	47.994000	50.224000	54.449000
O	48.667000	51.894000	51.341000	50.145000	49.079000	53.514000
O	48.785000	48.230000	49.245000	46.621000	47.306000	51.931000
O	46.780000	53.052000	49.855000	49.569000	48.359000	56.014000
O	46.336000	48.750000	50.091000	47.965000	45.266000	53.288000
O	49.386000	50.199000	47.604000	45.845000	49.054000	53.659000
O	47.388000	51.982000	47.521000	47.039000	48.312000	56.047000
O	47.063000	49.229000	47.534000	45.918000	46.557000	54.301000
O	45.515000	50.841000	48.838000	48.193000	46.044000	55.756000
O	48.296000	49.253000	51.671000	49.148000	47.201000	51.774000
O	46.261000	50.924000	51.361000	50.152000	46.577000	54.041000
O	56.144000	50.882000	50.572000	47.326000	54.764000	49.117000
O	57.633000	49.587000	48.700000	45.167000	55.555000	47.946000
O	55.583000	48.460000	49.583000	46.629000	54.031000	46.510000
O	57.744000	49.053000	51.204000	47.382000	56.525000	47.134000
O	58.785000	48.005000	46.985000	43.060000	56.315000	46.752000
O	56.697000	46.673000	51.321000	47.295000	55.719000	44.601000
O	59.939000	46.336000	48.767000	43.883000	57.896000	44.760000
O	56.246000	47.705000	47.158000	43.944000	53.783000	46.396000
O	55.597000	45.918000	48.901000	45.418000	53.866000	44.322000
O	57.726000	45.559000	47.216000	43.189000	55.369000	44.317000
O	57.982000	44.983000	49.684000	45.272000	56.376000	43.316000
O	59.749000	48.623000	49.533000	45.000000	57.996000	47.128000

O	59.190000	46.803000	51.341000	46.383000	57.999000	45.210000
C	25.548000	40.571000	41.448000	35.298000	41.484000	54.433000
C	25.723000	43.065000	41.891000	33.669000	43.625000	55.015000
C	25.802000	41.958000	40.778000	34.353000	42.628000	53.994000
C	27.125000	42.061000	40.056000	35.045000	43.655000	53.035000
C	30.057000	39.063000	41.901000	38.963000	42.250000	53.109000
C	29.619000	36.849000	43.122000	39.246000	39.464000	53.828000
C	29.031000	37.820000	42.043000	38.669000	40.707000	53.038000
C	28.593000	37.253000	40.739000	38.681000	40.376000	51.495000
C	31.974000	42.024000	39.690000	33.770000	46.961000	50.787000
C	32.648000	44.123000	38.495000	35.231000	48.904000	51.073000
C	32.362000	42.618000	38.298000	35.218000	47.375000	50.952000
C	31.133000	42.620000	37.332000	36.239000	46.968000	49.912000
C	30.434000	40.870000	31.667000	31.575000	43.580000	45.217000
C	29.471000	43.014000	31.072000	31.729000	45.945000	44.699000
C	29.927000	42.130000	32.313000	32.576000	44.686000	45.080000
C	28.599000	41.751000	32.921000	33.241000	45.124000	46.411000
C	24.977000	43.584000	35.223000	33.116000	39.724000	51.904000
C	22.961000	42.464000	34.308000	30.658000	40.148000	51.841000
C	24.388000	42.258000	34.676000	32.041000	40.680000	51.458000
C	24.330000	41.043000	35.633000	32.089000	41.027000	49.939000
C	25.468000	33.120000	36.478000	37.322000	36.153000	47.519000
C	27.633000	34.401000	35.674000	38.243000	38.409000	46.989000
C	26.344000	34.458000	36.484000	37.303000	37.640000	47.880000
C	25.698000	35.896000	36.316000	35.979000	38.288000	48.306000
C	33.715000	39.070000	38.638000	39.798000	45.037000	45.560000
C	32.588000	37.319000	39.960000	40.345000	46.516000	47.537000
C	33.190000	37.628000	38.585000	40.540000	45.118000	46.863000
C	32.303000	37.535000	37.353000	40.288000	44.059000	47.896000
C	34.931000	35.075000	41.233000	46.878000	44.790000	36.317000
C	34.384000	34.566000	42.549000	48.348000	44.740000	36.838000
C	35.446000	34.197000	43.645000	48.745000	45.361000	38.269000
C	36.563000	33.330000	43.109000	47.557000	45.051000	39.249000
C	37.016000	33.581000	41.617000	46.162000	45.186000	38.692000
C	36.149000	34.316000	40.591000	45.873000	44.365000	37.430000
C	39.171000	32.682000	32.563000	39.758000	38.782000	35.310000
C	40.088000	31.626000	31.891000	39.561000	37.314000	35.620000
C	39.694000	30.225000	32.070000	40.784000	36.470000	35.378000
C	38.549000	29.850000	33.004000	42.037000	37.067000	36.051000
C	38.479000	30.915000	34.179000	42.188000	38.543000	36.085000
C	38.246000	32.387000	33.739000	40.873000	39.319000	36.207000
C	42.301000	33.409000	38.289000	43.721000	40.932000	32.097000
C	43.437000	32.408000	38.640000	44.610000	39.738000	31.535000
C	43.065000	30.915000	38.550000	46.097000	39.971000	31.620000
C	41.536000	30.475000	38.603000	46.664000	40.924000	32.641000

C	40.675000	31.495000	37.947000	45.675000	42.066000	33.030000
C	40.854000	32.953000	38.347000	44.253000	41.580000	33.369000
C	41.804000	38.778000	38.859000	40.479000	44.400000	31.089000
C	43.226000	39.232000	39.201000	39.779000	45.100000	29.952000
C	44.233000	38.117000	39.609000	40.691000	46.302000	29.528000
C	44.361000	37.078000	38.427000	41.223000	47.259000	30.599000
C	42.961000	36.630000	37.952000	41.532000	46.510000	31.861000
C	41.829000	37.680000	37.778000	40.716000	45.307000	32.252000
C	39.273000	38.636000	32.475000	36.611000	43.777000	33.890000
C	39.642000	38.966000	31.027000	35.328000	43.512000	33.094000
C	40.942000	38.162000	30.594000	34.922000	42.072000	32.873000
C	40.784000	36.682000	30.699000	35.692000	41.032000	33.778000
C	39.990000	36.196000	31.971000	37.126000	41.288000	34.055000
C	39.071000	37.095000	32.755000	37.341000	42.706000	34.696000
C	36.433000	40.586000	38.727000	43.545000	48.353000	37.487000
C	35.839000	41.725000	39.564000	43.783000	49.808000	37.693000
C	36.736000	42.175000	40.692000	42.549000	50.768000	37.782000
C	37.458000	40.988000	41.352000	41.442000	50.309000	36.882000
C	37.969000	39.844000	40.487000	41.215000	48.810000	36.701000
C	36.945000	39.301000	39.403000	42.469000	47.894000	36.538000
C	34.886000	40.288000	35.666000	37.616000	44.468000	39.123000
C	33.912000	41.245000	34.930000	36.313000	45.011000	39.638000
C	32.876000	40.759000	33.901000	35.918000	46.420000	39.138000
C	32.853000	39.271000	33.631000	37.257000	47.339000	39.330000
C	33.811000	38.204000	34.236000	38.437000	46.955000	38.389000
C	34.813000	38.724000	35.258000	38.799000	45.488000	38.757000
C	31.867000	35.427000	34.427000	38.607000	42.565000	42.515000
C	32.804000	34.026000	38.769000	42.179000	43.194000	42.948000
C	32.559000	31.833000	36.588000	41.581000	40.378000	41.908000
C	31.529000	34.603000	35.698000	39.562000	42.477000	41.269000
C	31.381000	36.088000	31.419000	38.280000	39.681000	43.452000
C	29.668000	34.223000	32.716000	39.833000	41.796000	45.213000
C	22.099000	36.855000	45.043000	19.623000	40.018000	50.710000
C	20.774000	38.672000	44.135000	19.091000	42.521000	51.352000
C	21.006000	37.135000	43.983000	18.571000	41.068000	51.153000
C	21.411000	36.788000	42.542000	17.218000	41.031000	50.322000
C	15.381000	31.805000	42.557000	13.859000	40.194000	54.058000
C	14.534000	33.938000	43.684000	13.962000	38.705000	56.077000
C	15.778000	33.183000	43.293000	13.734000	38.748000	54.558000
C	16.807000	34.087000	42.504000	14.671000	37.794000	53.728000
C	22.157000	33.361000	46.098000	20.240000	36.091000	51.148000
C	24.614000	32.722000	46.641000	21.891000	35.924000	49.118000
C	23.425000	32.614000	45.650000	20.451000	35.894000	49.682000
C	23.871000	32.822000	44.184000	19.681000	36.838000	48.737000
C	26.284000	30.839000	41.242000	18.300000	35.225000	45.564000

C	24.727000	29.012000	40.495000	16.143000	34.094000	44.890000
C	25.541000	30.261000	40.055000	17.031000	35.332000	44.742000
C	24.712000	31.358000	39.402000	16.074000	36.517000	45.127000
C	22.888000	37.693000	36.695000	14.173000	42.105000	44.775000
C	22.692000	37.660000	39.268000	14.682000	39.708000	44.259000
C	22.204000	37.009000	37.902000	14.590000	40.731000	45.353000
C	22.440000	35.485000	37.799000	13.718000	40.523000	46.593000
C	15.590000	32.454000	38.718000	10.815000	36.953000	52.850000
C	16.728000	30.362000	38.173000	10.089000	35.310000	50.885000
C	16.551000	31.807000	37.742000	10.167000	36.754000	51.469000
C	17.829000	32.600000	37.582000	10.675000	37.747000	50.356000
C	18.066000	28.916000	45.916000	17.200000	32.491000	53.326000
C	19.212000	31.178000	46.132000	15.258000	34.075000	53.544000
C	18.587000	30.142000	45.170000	16.102000	33.183000	52.534000
C	19.355000	29.741000	43.903000	16.840000	33.981000	51.448000
C	12.514000	31.330000	37.806000	12.215000	27.997000	50.814000
C	11.810000	32.029000	38.973000	11.714000	28.044000	52.190000
C	12.329000	31.845000	40.445000	12.154000	27.136000	53.313000
C	12.961000	30.401000	40.716000	12.372000	25.704000	52.844000
C	13.724000	29.869000	39.479000	13.100000	25.714000	51.462000
C	12.895000	29.864000	38.156000	12.553000	26.561000	50.269000
C	18.872000	26.862000	33.032000	14.429000	30.422000	43.635000
C	20.346000	26.669000	32.602000	14.415000	31.653000	42.814000
C	21.049000	25.541000	33.377000	13.342000	32.578000	43.434000
C	20.441000	25.297000	34.748000	11.931000	31.981000	43.553000
C	18.909000	25.272000	34.830000	11.998000	30.690000	44.379000
C	18.218000	26.559000	34.396000	13.126000	29.708000	43.913000
C	16.386000	31.797000	33.006000	8.870000	28.318000	45.757000
C	17.017000	33.234000	32.813000	7.386000	28.377000	45.306000
C	16.485000	34.275000	33.816000	6.434000	27.515000	46.117000
C	15.973000	33.693000	35.125000	6.977000	26.120000	46.443000
C	14.998000	32.506000	34.863000	8.523000	26.036000	46.870000
C	15.746000	31.300000	34.298000	9.443000	26.917000	46.012000
C	12.696000	30.315000	30.578000	10.830000	21.473000	44.643000
C	11.905000	31.044000	29.475000	9.904000	20.678000	43.695000
C	10.975000	30.177000	28.603000	10.514000	20.505000	42.268000
C	10.758000	28.659000	28.938000	11.369000	21.577000	41.613000
C	11.111000	28.142000	30.368000	12.329000	22.186000	42.689000
C	12.242000	28.949000	31.090000	11.674000	22.686000	44.031000
C	15.609000	23.094000	31.551000	13.625000	25.369000	40.995000
C	16.280000	22.502000	30.285000	13.726000	24.997000	39.495000
C	15.191000	22.427000	29.242000	15.002000	25.327000	38.729000
C	14.121000	23.512000	29.052000	16.147000	24.792000	39.684000
C	13.898000	24.465000	30.234000	16.111000	25.599000	40.981000
C	14.836000	24.461000	31.389000	14.884000	25.134000	41.805000

C	8.621000	26.783000	36.349000	15.048000	20.851000	47.310000
C	7.158000	27.092000	36.542000	15.289000	19.483000	48.157000
C	7.080000	28.626000	36.399000	15.996000	19.593000	49.567000
C	7.288000	29.005000	34.884000	15.443000	20.816000	50.319000
C	8.706000	28.517000	34.513000	15.167000	22.167000	49.593000
C	9.377000	27.331000	35.116000	14.811000	22.175000	48.104000
C	13.082000	21.835000	35.219000	19.048000	25.578000	47.153000
C	12.511000	20.687000	36.127000	20.456000	25.037000	47.251000
C	11.081000	20.144000	35.700000	21.282000	25.137000	45.947000
C	10.165000	21.320000	35.273000	20.514000	25.442000	44.680000
C	10.794000	22.653000	34.715000	19.005000	25.788000	44.676000
C	12.143000	23.088000	35.230000	18.175000	25.443000	45.881000
C	18.954000	26.981000	38.092000	13.055000	31.459000	47.363000
C	16.898000	23.285000	38.865000	17.566000	31.242000	47.007000
C	16.486000	24.716000	40.769000	16.001000	31.136000	49.730000
C	18.187000	25.673000	38.054000	14.534000	31.638000	46.985000
C	18.483000	28.122000	40.800000	10.390000	32.270000	48.294000
C	21.191000	26.382000	40.272000	12.989000	32.064000	50.354000
C	47.307000	24.022000	37.848000	43.903000	14.292000	41.655000
C	44.822000	23.574000	38.312000	41.934000	15.220000	40.236000
C	45.974000	23.378000	37.308000	43.013000	15.487000	41.287000
C	45.493000	23.854000	35.894000	42.191000	16.013000	42.449000
C	42.296000	17.292000	34.223000	36.191000	16.331000	38.318000
C	44.664000	16.314000	34.751000	37.098000	14.225000	37.225000
C	43.825000	17.510000	34.151000	37.432000	15.447000	38.155000
C	44.026000	18.813000	34.938000	37.785000	15.123000	39.554000
C	44.883000	23.727000	30.238000	41.202000	20.937000	37.872000
C	44.790000	26.187000	29.845000	43.051000	22.062000	38.919000
C	43.958000	24.945000	30.190000	41.603000	21.669000	39.183000
C	43.427000	25.230000	31.591000	41.725000	20.704000	40.397000
C	36.886000	24.873000	35.127000	40.766000	20.682000	46.612000
C	38.072000	26.441000	36.765000	41.585000	22.780000	45.436000
C	37.791000	26.108000	35.287000	40.433000	22.017000	46.043000
C	39.082000	26.068000	34.429000	39.225000	21.990000	45.113000
C	41.184000	26.366000	40.223000	41.398000	15.822000	47.559000
C	41.590000	27.036000	37.717000	40.401000	14.697000	45.524000
C	40.880000	26.168000	38.711000	40.977000	16.033000	46.117000
C	41.329000	24.624000	38.577000	39.841000	17.125000	46.125000
C	39.217000	22.025000	39.826000	36.005000	13.717000	42.972000
C	37.566000	20.488000	38.732000	36.020000	14.338000	45.557000
C	39.035000	20.648000	39.213000	35.349000	14.475000	44.191000
C	40.081000	20.046000	38.181000	35.209000	16.020000	44.072000
C	43.985000	19.872000	30.091000	37.668000	18.103000	36.428000
C	42.218000	19.991000	28.179000	37.525000	20.628000	35.790000
C	42.682000	20.493000	29.561000	37.845000	19.553000	36.880000

C	41.603000	20.371000	30.687000	37.130000	19.963000	38.172000
C	35.437000	29.301000	26.149000	37.267000	28.887000	47.379000
C	36.358000	30.528000	26.223000	38.726000	29.405000	47.270000
C	37.829000	30.287000	26.191000	38.948000	30.921000	47.159000
C	38.136000	29.008000	27.035000	38.080000	31.520000	46.074000
C	37.042000	28.072000	27.511000	36.603000	30.996000	46.201000
C	35.534000	28.247000	27.209000	36.387000	29.482000	46.279000
C	33.887000	20.663000	23.955000	32.311000	27.508000	40.312000
C	34.517000	19.755000	22.832000	32.741000	28.052000	38.953000
C	36.001000	19.799000	22.451000	31.552000	28.843000	38.239000
C	36.798000	20.664000	23.464000	30.761000	29.867000	39.069000
C	36.090000	21.813000	24.222000	30.758000	29.733000	40.635000
C	34.692000	21.621000	24.859000	31.801000	28.658000	41.175000
C	34.390000	26.920000	22.187000	34.106000	33.449000	44.788000
C	34.973000	27.036000	20.770000	34.418000	34.774000	44.080000
C	34.315000	26.123000	19.706000	33.294000	35.645000	43.469000
C	34.175000	24.661000	20.228000	32.187000	34.716000	42.933000
C	33.658000	24.571000	21.682000	31.973000	33.301000	43.602000
C	34.137000	25.629000	22.732000	33.238000	32.517000	43.978000
C	29.000000	27.448000	24.128000	29.050000	32.654000	46.367000
C	27.613000	27.740000	23.491000	27.874000	33.480000	46.797000
C	27.391000	27.292000	22.064000	27.537000	33.630000	48.306000
C	27.905000	25.776000	21.965000	27.863000	32.396000	49.122000
C	29.283000	25.449000	22.537000	28.778000	31.256000	48.535000
C	29.452000	25.929000	23.981000	29.486000	31.387000	47.147000
C	28.453000	21.451000	26.424000	28.040000	26.436000	42.888000
C	27.568000	20.171000	26.187000	26.643000	25.988000	42.433000
C	27.643000	19.003000	27.261000	25.510000	25.898000	43.430000
C	29.132000	18.837000	27.528000	25.529000	27.199000	44.316000
C	30.120000	20.014000	27.845000	26.832000	27.504000	44.895000
C	29.939000	21.150000	26.802000	28.154000	27.469000	44.065000
C	29.357000	28.948000	27.289000	32.556000	29.379000	50.849000
C	28.094000	29.714000	27.837000	32.551000	29.354000	52.420000
C	28.149000	30.153000	29.298000	31.604000	28.229000	52.923000
C	29.125000	29.460000	30.297000	31.845000	26.942000	52.176000
C	30.325000	28.851000	29.597000	31.867000	26.999000	50.628000
C	30.156000	28.085000	28.242000	32.633000	28.131000	49.907000
C	30.915000	22.181000	31.177000	30.232000	23.409000	46.585000
C	30.255000	21.816000	32.557000	29.957000	21.993000	47.235000
C	30.970000	22.404000	33.800000	29.692000	22.239000	48.773000
C	30.999000	23.889000	33.516000	30.878000	22.967000	49.372000
C	31.652000	24.314000	32.174000	31.400000	24.319000	48.749000
C	31.011000	23.674000	30.895000	31.145000	24.433000	47.245000
C	35.108000	22.719000	33.455000	33.645000	21.851000	44.705000
C	37.097000	24.642000	31.634000	36.437000	23.529000	44.652000

C	37.946000	22.190000	30.686000	34.908000	23.810000	41.527000
C	34.877000	22.967000	31.962000	33.711000	23.295000	44.276000
C	35.812000	21.430000	36.036000	33.256000	18.918000	44.245000
C	35.697000	19.751000	33.309000	32.582000	20.804000	41.805000
C	59.222000	42.033000	50.133000	44.916000	58.056000	40.468000
C	61.111000	41.372000	48.607000	42.541000	59.296000	40.386000
C	60.366000	42.596000	49.262000	43.621000	58.530000	41.200000
C	59.834000	43.584000	48.195000	42.907000	57.520000	42.064000
C	63.331000	45.280000	52.153000	44.509000	62.568000	43.774000
C	63.218000	44.996000	49.597000	42.758000	60.782000	43.529000
C	62.464000	45.308000	50.900000	44.217000	61.061000	43.951000
C	61.888000	46.747000	50.762000	44.532000	60.527000	45.379000
C	55.205000	41.826000	51.584000	48.456000	54.786000	39.791000
C	55.348000	43.876000	53.209000	49.647000	55.564000	41.720000
C	55.877000	43.112000	52.011000	48.258000	55.329000	41.265000
C	55.634000	43.909000	50.682000	47.168000	54.538000	42.038000
C	52.569000	49.987000	47.843000	44.194000	51.032000	48.247000
C	53.107000	48.718000	45.665000	43.276000	50.346000	45.925000
C	52.631000	48.571000	47.131000	44.480000	50.485000	46.789000
C	53.401000	47.390000	47.872000	45.491000	51.323000	46.010000
C	59.226000	46.780000	43.816000	41.304000	52.642000	44.016000
C	57.249000	47.723000	42.368000	39.674000	52.073000	45.960000
C	57.641000	46.913000	43.597000	40.952000	52.716000	45.539000
C	56.940000	47.390000	44.888000	41.248000	54.125000	46.101000
C	62.259000	49.570000	46.829000	41.171000	55.921000	49.482000
C	60.236000	49.909000	45.083000	43.412000	55.400000	50.714000
C	61.046000	50.455000	46.349000	42.364000	56.433000	50.231000
C	60.044000	50.577000	47.548000	43.178000	57.332000	49.316000
C	55.925000	49.453000	54.256000	49.239000	60.012000	46.624000
C	55.971000	46.924000	54.814000	50.163000	58.090000	47.817000
C	56.751000	48.209000	54.682000	49.901000	58.679000	46.433000
C	57.982000	47.894000	53.787000	49.276000	57.769000	45.314000
C	49.996000	52.469000	44.910000	44.852000	50.610000	57.030000
C	50.658000	53.522000	43.997000	44.117000	51.790000	57.584000
C	52.068000	53.888000	44.476000	44.124000	53.168000	57.020000
C	52.163000	54.274000	45.922000	45.373000	53.543000	56.139000
C	51.493000	53.111000	46.819000	45.706000	52.255000	55.260000
C	50.040000	52.800000	46.413000	45.944000	50.889000	55.994000
C	49.634000	46.375000	51.920000	48.920000	48.788000	48.789000
C	50.568000	45.214000	52.384000	48.593000	48.974000	47.266000
C	51.643000	44.582000	51.556000	48.505000	47.676000	46.501000
C	52.456000	45.749000	51.113000	47.402000	46.719000	47.131000
C	51.636000	46.904000	50.495000	47.694000	46.613000	48.562000
C	50.432000	47.475000	51.237000	47.894000	47.906000	49.404000
C	49.222000	48.123000	44.903000	43.441000	47.978000	51.030000

C	50.005000	47.126000	43.874000	42.063000	48.392000	50.564000
C	49.723000	45.624000	44.042000	41.316000	48.932000	51.817000
C	49.918000	45.220000	45.474000	41.186000	47.878000	52.947000
C	49.266000	45.997000	46.599000	42.607000	47.457000	53.312000
C	49.185000	47.567000	46.373000	43.783000	47.295000	52.400000
C	43.683000	48.873000	46.625000	46.874000	42.739000	55.652000
C	42.686000	47.944000	45.845000	46.013000	41.503000	56.080000
C	41.782000	47.172000	46.834000	44.571000	41.275000	55.277000
C	42.346000	46.912000	48.267000	43.866000	42.531000	54.758000
C	43.351000	47.964000	48.904000	44.875000	43.600000	54.168000
C	44.368000	48.396000	47.907000	46.105000	43.921000	55.091000
C	46.226000	47.041000	53.382000	51.842000	44.791000	51.627000
C	45.555000	46.337000	54.580000	52.610000	43.406000	51.484000
C	44.056000	46.502000	54.462000	51.942000	42.333000	50.703000
C	43.523000	47.987000	54.358000	50.657000	42.712000	49.894000
C	44.329000	48.818000	53.370000	49.871000	43.837000	50.648000
C	45.817000	48.486000	52.954000	50.399000	44.543000	51.949000
C	43.802000	52.786000	46.691000	48.526000	48.638000	59.223000
C	42.602000	53.582000	46.236000	48.609000	48.427000	60.767000
C	42.515000	55.120000	46.383000	49.685000	47.555000	61.380000
C	43.615000	55.845000	47.216000	50.099000	46.410000	60.396000
C	44.760000	55.019000	47.867000	49.965000	46.632000	58.880000
C	44.762000	53.452000	47.720000	48.693000	47.290000	58.374000
C	45.635000	52.991000	53.685000	53.407000	47.496000	54.345000
C	45.574000	53.808000	55.011000	54.843000	47.420000	54.798000
C	45.745000	55.306000	54.879000	55.207000	48.683000	55.639000
C	47.004000	55.585000	54.131000	54.213000	48.894000	56.742000
C	47.335000	54.786000	52.870000	52.805000	49.053000	56.153000
C	46.716000	53.383000	52.675000	52.298000	47.980000	55.325000
C	54.396000	51.976000	52.537000	49.615000	53.958000	49.943000
C	49.995000	53.349000	53.669000	52.438000	51.516000	52.999000
C	51.977000	54.893000	51.819000	50.706000	53.961000	53.550000
C	53.000000	52.245000	53.280000	50.613000	52.990000	50.614000
C	53.542000	49.669000	51.191000	47.352000	51.966000	48.770000
C	54.116000	52.669000	49.601000	46.678000	53.312000	51.323000
H	25.571000	39.873000	40.610000	35.532000	41.141000	53.439000
H	24.643000	40.270000	41.878000	34.656000	40.787000	54.916000
H	26.294000	40.488000	42.201000	36.104000	41.797000	55.109000
H	25.750000	43.823000	41.087000	33.142000	44.417000	54.548000
H	26.594000	43.174000	42.521000	34.330000	44.234000	55.648000
H	24.764000	43.346000	42.348000	32.976000	42.995000	55.533000
H	25.046000	42.004000	39.989000	33.543000	42.234000	53.404000
H	26.844000	42.988000	39.590000	34.131000	44.200000	52.869000
H	27.970000	42.077000	40.727000	35.661000	44.367000	53.607000
H	29.770000	39.805000	41.170000	38.046000	42.467000	52.656000

H	31.017000	38.649000	41.631000	39.816000	42.397000	52.449000
H	30.164000	39.520000	42.872000	38.850000	42.606000	54.146000
H	30.013000	37.263000	44.045000	39.048000	39.484000	54.865000
H	30.369000	36.358000	42.515000	40.291000	39.476000	53.506000
H	28.806000	36.122000	43.192000	38.732000	38.592000	53.471000
H	28.244000	38.451000	42.421000	37.607000	40.593000	53.241000
H	29.633000	36.961000	40.707000	39.756000	40.373000	51.416000
H	27.984000	36.371000	40.828000	38.422000	39.362000	51.171000
H	32.652000	41.999000	40.538000	33.410000	47.597000	51.583000
H	31.929000	40.973000	39.372000	33.437000	45.987000	50.790000
H	31.133000	42.479000	40.106000	33.669000	47.472000	49.817000
H	33.414000	44.265000	39.219000	34.360000	49.393000	51.564000
H	31.832000	44.721000	38.791000	35.041000	48.943000	50.000000
H	32.913000	44.523000	37.543000	36.142000	49.390000	51.348000
H	33.219000	42.151000	37.877000	35.626000	47.262000	51.929000
H	30.453000	43.147000	38.007000	35.651000	47.584000	49.229000
H	31.244000	43.191000	36.444000	37.293000	47.316000	49.887000
H	29.505000	40.521000	31.171000	30.824000	44.024000	45.809000
H	30.807000	40.119000	32.339000	32.027000	42.656000	45.467000
H	31.275000	41.119000	31.089000	31.019000	43.427000	44.286000
H	30.368000	43.264000	30.526000	31.424000	45.846000	43.642000
H	29.144000	44.039000	31.372000	32.436000	46.666000	44.954000
H	28.674000	42.593000	30.420000	30.917000	46.120000	45.383000
H	30.741000	42.580000	32.865000	33.361000	44.353000	44.474000
H	28.323000	42.740000	33.211000	33.996000	45.597000	45.723000
H	27.805000	41.363000	32.293000	32.564000	45.709000	47.042000
H	24.404000	43.751000	36.104000	33.145000	38.706000	51.487000
H	24.849000	44.250000	34.417000	33.087000	39.630000	52.987000
H	25.987000	43.384000	35.406000	33.971000	40.441000	51.627000
H	22.464000	41.590000	33.887000	29.863000	40.796000	51.477000
H	22.916000	43.274000	33.647000	30.756000	40.025000	52.947000
H	22.451000	42.727000	35.250000	30.846000	39.189000	51.370000
H	25.105000	42.079000	33.880000	31.989000	41.665000	51.853000
H	23.957000	41.811000	36.323000	32.144000	40.024000	49.639000
H	23.700000	40.249000	35.797000	31.167000	41.435000	49.527000
H	24.850000	33.152000	35.556000	36.829000	35.983000	46.643000
H	24.822000	33.299000	37.339000	36.822000	35.814000	48.363000
H	26.184000	32.326000	36.550000	38.327000	35.798000	47.492000
H	28.072000	33.436000	35.878000	39.259000	37.920000	46.779000
H	28.187000	35.155000	36.137000	38.250000	39.470000	47.308000
H	27.653000	34.467000	34.612000	37.684000	38.367000	46.065000
H	26.828000	34.212000	37.409000	37.994000	37.625000	48.704000
H	25.405000	36.041000	35.292000	35.348000	38.025000	47.473000
H	24.816000	36.044000	36.938000	35.617000	37.779000	49.221000
H	34.276000	39.117000	39.509000	40.151000	45.709000	44.798000

H	32.910000	39.700000	38.821000	38.722000	44.933000	45.541000
H	34.184000	39.451000	37.715000	40.123000	44.126000	45.072000
H	32.403000	36.316000	39.811000	41.046000	46.758000	48.279000
H	31.676000	37.804000	40.208000	39.391000	46.769000	47.945000
H	33.296000	37.438000	40.760000	40.753000	47.127000	46.714000
H	33.978000	36.928000	38.237000	41.628000	44.972000	46.595000
H	33.019000	37.805000	36.586000	40.814000	43.205000	47.479000
H	32.092000	36.493000	37.302000	40.766000	44.256000	48.864000
H	34.124000	35.385000	40.645000	46.985000	44.145000	35.480000
H	33.822000	35.317000	43.081000	49.209000	44.677000	36.101000
H	35.104000	34.117000	44.696000	49.745000	44.811000	38.309000
H	37.327000	33.142000	43.821000	47.849000	45.431000	40.208000
H	37.891000	33.166000	41.256000	45.810000	44.907000	39.632000
H	39.233000	33.671000	32.050000	38.948000	39.449000	35.389000
H	40.277000	31.747000	30.860000	38.783000	37.009000	34.907000
H	39.746000	29.447000	31.364000	40.789000	35.398000	35.513000
H	38.769000	28.854000	33.372000	42.957000	36.581000	35.826000
H	37.683000	30.693000	34.969000	42.889000	38.846000	36.856000
H	42.325000	34.444000	38.594000	42.668000	41.011000	32.125000
H	44.434000	32.797000	38.771000	44.615000	39.170000	30.575000
H	43.744000	30.080000	38.686000	46.755000	39.191000	31.203000
H	41.088000	29.516000	38.277000	47.604000	41.504000	32.483000
H	39.653000	31.257000	38.085000	46.153000	42.549000	33.786000
H	41.161000	39.358000	38.169000	39.886000	43.532000	31.342000
H	43.293000	39.872000	40.064000	39.754000	44.549000	29.054000
H	45.174000	38.561000	39.853000	40.179000	46.862000	28.796000
H	45.255000	36.398000	38.406000	41.493000	48.282000	30.592000
H	42.960000	35.964000	37.100000	41.910000	47.241000	32.690000
H	40.261000	38.960000	32.940000	36.942000	44.756000	34.109000
H	39.667000	40.050000	31.117000	35.184000	44.230000	32.335000
H	41.445000	38.487000	29.699000	33.862000	42.149000	32.620000
H	41.622000	36.248000	30.089000	35.377000	40.004000	33.549000
H	39.904000	35.110000	31.866000	37.562000	40.387000	34.498000
H	35.846000	40.329000	37.903000	44.494000	47.859000	37.408000
H	35.513000	42.691000	39.240000	44.681000	50.212000	38.179000
H	36.474000	43.036000	41.249000	43.268000	51.582000	37.844000
H	38.141000	41.335000	42.155000	40.739000	51.097000	36.881000
H	38.515000	39.015000	40.964000	40.460000	48.293000	36.102000
H	35.686000	40.573000	36.290000	38.005000	43.549000	39.423000
H	33.813000	42.291000	35.313000	35.581000	44.173000	39.761000
H	32.145000	41.322000	33.353000	35.012000	46.792000	39.580000
H	32.218000	39.030000	32.702000	37.142000	48.413000	39.105000
H	33.806000	37.148000	34.039000	39.187000	47.664000	38.352000
H	32.685000	34.898000	33.947000	37.640000	42.530000	41.916000
H	32.254000	36.283000	34.939000	38.951000	43.499000	42.961000

H	33.720000	33.483000	38.911000	41.679000	44.173000	42.797000
H	32.024000	33.506000	39.308000	43.184000	43.384000	42.710000
H	32.992000	35.043000	38.963000	41.970000	42.646000	43.817000
H	33.026000	31.415000	35.733000	42.584000	40.212000	42.360000
H	31.514000	31.689000	36.615000	41.397000	39.812000	40.996000
H	33.072000	31.323000	37.439000	40.938000	39.957000	42.609000
H	30.827000	35.272000	36.232000	39.243000	41.674000	40.623000
H	31.071000	33.695000	35.474000	39.318000	43.410000	40.807000
H	30.492000	36.301000	30.863000	37.423000	39.671000	42.818000
H	31.750000	35.065000	31.344000	38.038000	38.984000	44.227000
H	32.105000	36.935000	31.488000	39.212000	39.289000	43.042000
H	28.928000	34.101000	33.474000	40.565000	42.270000	44.594000
H	30.491000	33.562000	32.842000	40.148000	40.839000	45.661000
H	29.336000	34.192000	31.720000	39.458000	42.444000	45.954000
H	22.764000	37.627000	44.636000	20.107000	40.081000	49.778000
H	21.801000	37.198000	46.057000	20.295000	39.811000	51.563000
H	22.665000	35.924000	45.037000	18.823000	39.287000	50.673000
H	21.642000	39.242000	43.798000	19.312000	42.852000	50.395000
H	20.458000	38.786000	45.168000	19.946000	42.519000	51.967000
H	19.981000	38.989000	43.438000	18.352000	43.215000	51.818000
H	20.072000	36.644000	44.278000	18.357000	40.620000	52.140000
H	20.450000	37.225000	42.182000	16.667000	41.756000	50.892000
H	22.259000	37.298000	42.168000	17.533000	41.427000	49.362000
H	14.710000	31.531000	43.338000	13.409000	41.043000	54.563000
H	16.084000	31.141000	42.276000	13.640000	40.193000	52.952000
H	14.793000	32.181000	41.712000	14.928000	40.273000	54.091000
H	14.813000	34.743000	44.296000	13.742000	37.659000	56.270000
H	13.937000	33.199000	44.252000	13.137000	39.336000	56.450000
H	14.096000	34.365000	42.847000	14.917000	38.885000	56.547000
H	16.368000	32.883000	44.129000	12.770000	38.302000	54.467000
H	16.326000	34.111000	41.568000	15.349000	38.568000	54.071000
H	17.004000	35.000000	43.085000	14.974000	36.895000	54.205000
H	21.700000	33.039000	46.999000	21.022000	35.404000	51.563000
H	21.608000	33.222000	45.189000	19.238000	35.693000	51.421000
H	22.555000	34.374000	46.169000	20.424000	37.114000	51.493000
H	24.200000	32.347000	47.533000	22.551000	35.333000	49.720000
H	25.079000	33.657000	46.894000	22.148000	36.940000	49.237000
H	25.345000	31.960000	46.278000	21.864000	35.602000	48.055000
H	23.153000	31.581000	45.536000	20.116000	34.876000	49.484000
H	24.017000	33.924000	44.333000	20.061000	37.783000	49.072000
H	24.682000	32.378000	43.688000	19.704000	36.665000	47.701000
H	25.551000	31.197000	41.958000	17.927000	35.301000	46.523000
H	26.967000	31.615000	40.864000	18.867000	36.114000	45.331000
H	26.825000	30.038000	41.740000	18.973000	34.357000	45.363000
H	25.487000	28.513000	41.013000	16.768000	33.202000	44.886000

H	24.134000	28.346000	39.842000	15.246000	33.974000	44.242000
H	24.070000	29.664000	41.097000	15.881000	34.340000	45.872000
H	26.286000	29.944000	39.295000	17.340000	35.281000	43.642000
H	25.346000	32.199000	39.342000	16.722000	37.388000	44.883000
H	24.436000	30.972000	38.413000	15.130000	36.533000	44.558000
H	22.414000	37.202000	35.907000	14.448000	42.809000	45.528000
H	23.915000	37.343000	36.721000	13.048000	42.152000	44.752000
H	22.851000	38.757000	36.554000	14.591000	42.651000	43.908000
H	22.119000	37.253000	40.062000	14.926000	38.671000	44.581000
H	22.536000	38.742000	39.176000	15.541000	40.104000	43.692000
H	23.785000	37.460000	39.316000	13.741000	39.802000	43.788000
H	21.179000	37.265000	37.906000	15.562000	40.887000	45.735000
H	21.966000	35.290000	36.838000	13.966000	41.508000	47.013000
H	23.468000	35.150000	37.524000	12.630000	40.429000	46.333000
H	16.029000	32.326000	39.726000	11.835000	36.527000	52.831000
H	14.633000	31.979000	38.524000	10.417000	36.351000	53.671000
H	15.559000	33.548000	38.534000	10.765000	38.008000	53.059000
H	15.716000	29.993000	38.224000	9.785000	34.381000	51.364000
H	17.219000	30.038000	39.025000	11.163000	35.125000	50.672000
H	17.231000	30.038000	37.321000	9.479000	35.330000	49.977000
H	16.050000	31.818000	36.794000	9.170000	36.967000	51.688000
H	18.174000	31.869000	36.892000	10.084000	37.342000	49.483000
H	17.636000	33.441000	36.975000	10.308000	38.722000	50.470000
H	17.433000	28.357000	45.222000	17.630000	31.662000	52.786000
H	17.654000	29.014000	46.893000	16.844000	32.035000	54.252000
H	18.914000	28.316000	46.143000	18.056000	33.070000	53.608000
H	19.385000	32.089000	45.609000	14.386000	34.637000	53.288000
H	20.135000	30.757000	46.561000	15.933000	34.860000	53.861000
H	18.610000	31.525000	46.980000	14.837000	33.581000	54.434000
H	17.613000	30.517000	44.945000	15.363000	32.548000	52.174000
H	20.244000	29.311000	44.422000	17.399000	34.575000	52.157000
H	18.645000	29.063000	43.517000	17.642000	33.577000	50.826000
H	11.681000	31.374000	37.096000	11.588000	28.669000	50.210000
H	11.370000	32.993000	38.760000	11.120000	28.918000	52.335000
H	11.549000	32.216000	41.133000	12.050000	27.577000	54.247000
H	13.242000	30.800000	41.684000	12.510000	24.927000	53.616000
H	14.364000	29.027000	39.707000	13.451000	24.756000	50.935000
H	18.241000	27.460000	32.401000	15.065000	29.705000	43.155000
H	20.645000	26.682000	31.541000	15.390000	32.039000	42.571000
H	22.094000	25.497000	33.301000	13.376000	33.454000	42.809000
H	21.023000	24.549000	35.301000	11.157000	32.683000	43.987000
H	18.636000	25.392000	35.861000	11.151000	30.039000	44.216000
H	17.255000	31.155000	32.806000	9.300000	28.735000	44.822000
H	17.367000	33.515000	31.849000	7.523000	27.733000	44.441000
H	17.120000	35.182000	33.862000	6.104000	27.927000	47.011000

H	15.623000	34.631000	35.591000	6.362000	25.546000	47.148000
H	14.305000	32.465000	35.672000	8.825000	24.964000	46.818000
H	13.489000	30.794000	31.059000	10.511000	21.840000	45.611000
H	12.020000	32.094000	29.559000	9.252000	19.923000	44.089000
H	10.493000	30.736000	27.850000	10.075000	19.964000	41.442000
H	9.829000	28.225000	28.571000	11.785000	21.087000	40.695000
H	10.977000	27.088000	30.492000	12.634000	23.020000	42.060000
H	16.330000	23.445000	32.326000	12.650000	25.177000	41.445000
H	16.738000	21.497000	30.363000	12.791000	25.099000	39.030000
H	15.466000	21.832000	28.419000	14.872000	24.943000	37.745000
H	13.361000	23.234000	28.315000	17.112000	24.849000	39.041000
H	13.285000	25.374000	30.092000	16.990000	25.354000	41.539000
H	8.928000	25.779000	36.727000	14.899000	20.803000	46.233000
H	6.877000	26.623000	37.522000	15.505000	18.575000	47.563000
H	6.007000	28.508000	36.308000	15.977000	18.581000	49.972000
H	7.301000	30.046000	34.568000	16.018000	20.809000	51.200000
H	9.130000	29.117000	33.717000	14.818000	22.912000	50.297000
H	14.101000	22.222000	35.402000	18.612000	25.264000	48.099000
H	13.285000	19.968000	36.346000	20.823000	25.171000	48.201000
H	10.423000	19.408000	36.158000	22.269000	24.803000	45.605000
H	9.224000	21.217000	34.806000	21.077000	25.541000	43.733000
H	10.239000	23.583000	34.693000	18.420000	25.947000	43.772000
H	18.647000	27.840000	37.498000	12.859000	30.395000	47.277000
H	19.842000	26.592000	37.604000	12.658000	32.061000	46.548000
H	15.981000	22.634000	38.910000	17.366000	31.481000	45.997000
H	17.579000	22.904000	39.664000	18.160000	30.382000	47.130000
H	17.354000	23.158000	37.867000	18.121000	32.100000	47.363000
H	16.631000	23.620000	40.587000	16.934000	31.586000	50.033000
H	15.465000	24.949000	41.128000	16.087000	30.197000	50.042000
H	17.330000	25.232000	41.222000	15.213000	31.743000	50.116000
H	17.776000	25.462000	37.073000	14.754000	31.031000	46.134000
H	18.944000	24.910000	38.272000	14.819000	32.679000	46.800000
H	19.049000	28.896000	41.390000	9.642000	31.982000	49.045000
H	18.068000	27.339000	41.409000	10.313000	31.808000	47.366000
H	17.870000	28.595000	40.067000	10.345000	33.305000	48.000000
H	20.755000	25.419000	40.092000	14.002000	32.412000	50.283000
H	21.449000	26.580000	41.332000	12.910000	30.958000	50.425000
H	22.008000	26.482000	39.564000	12.480000	32.444000	51.227000
H	47.395000	23.722000	38.820000	44.551000	14.074000	40.858000
H	47.012000	25.076000	37.705000	43.409000	13.415000	42.146000
H	47.935000	23.723000	37.090000	44.493000	14.755000	42.456000
H	45.115000	23.300000	39.324000	42.460000	14.865000	39.312000
H	43.817000	23.169000	38.094000	41.428000	16.154000	40.114000
H	44.707000	24.640000	38.382000	41.353000	14.425000	40.764000
H	46.373000	22.342000	37.171000	43.697000	16.148000	40.846000

H	45.041000	24.774000	36.175000	41.671000	15.135000	42.803000
H	46.300000	23.885000	35.179000	42.530000	16.520000	43.393000
H	42.048000	18.267000	33.918000	36.438000	17.269000	38.786000
H	42.180000	17.110000	35.265000	35.222000	15.892000	38.742000
H	41.796000	16.534000	33.638000	36.136000	16.483000	37.300000
H	44.374000	15.460000	34.240000	36.860000	14.713000	36.287000
H	44.363000	16.049000	35.728000	36.241000	13.618000	37.621000
H	45.758000	16.426000	34.665000	38.067000	13.785000	37.117000
H	43.785000	17.641000	33.103000	38.291000	15.999000	37.704000
H	43.760000	18.420000	35.941000	36.987000	14.524000	39.878000
H	45.013000	19.293000	34.944000	38.697000	14.620000	39.414000
H	45.464000	23.987000	31.097000	41.930000	20.110000	37.801000
H	45.526000	23.663000	29.408000	41.043000	21.342000	36.874000
H	44.383000	22.821000	30.542000	40.223000	20.555000	38.149000
H	45.073000	25.904000	28.835000	42.966000	22.635000	38.036000
H	45.656000	26.350000	30.392000	43.811000	21.342000	38.714000
H	44.162000	27.055000	29.741000	43.368000	22.928000	39.583000
H	43.020000	24.742000	29.582000	41.065000	22.598000	39.085000
H	44.399000	25.386000	32.064000	42.535000	20.024000	40.302000
H	42.903000	26.194000	31.751000	42.000000	21.449000	41.038000
H	36.118000	25.063000	35.876000	41.489000	21.042000	47.246000
H	37.417000	23.993000	35.456000	41.189000	20.217000	45.740000
H	36.634000	24.683000	34.107000	39.953000	20.152000	47.120000
H	38.670000	27.316000	36.530000	40.914000	23.664000	45.452000
H	38.689000	25.707000	37.248000	41.942000	22.408000	44.444000
H	37.246000	26.647000	37.440000	42.419000	22.727000	46.157000
H	36.899000	26.745000	35.110000	40.110000	22.620000	46.873000
H	38.435000	26.006000	33.566000	38.644000	21.656000	45.945000
H	39.738000	26.898000	34.325000	38.759000	22.984000	44.925000
H	40.759000	27.323000	40.484000	41.983000	14.925000	47.467000
H	42.166000	26.196000	40.563000	40.371000	15.671000	47.835000
H	40.561000	25.656000	40.692000	41.941000	16.778000	47.770000
H	41.126000	26.689000	36.806000	39.967000	14.991000	44.593000
H	42.701000	26.991000	37.776000	39.571000	14.319000	46.181000
H	41.082000	27.942000	38.114000	41.173000	13.957000	45.320000
H	39.809000	26.323000	38.568000	41.827000	16.278000	45.527000
H	42.356000	24.875000	38.849000	39.021000	16.714000	46.647000
H	40.868000	24.016000	39.396000	40.133000	18.074000	46.558000
H	40.265000	22.244000	40.111000	35.493000	14.086000	42.102000
H	38.537000	22.009000	40.619000	35.852000	12.641000	42.955000
H	39.058000	22.760000	39.045000	37.058000	13.968000	42.909000
H	37.116000	21.080000	37.936000	36.951000	14.782000	45.427000
H	37.210000	20.812000	39.702000	36.196000	13.299000	45.636000
H	37.316000	19.446000	38.468000	35.510000	14.700000	46.447000
H	38.875000	19.943000	39.976000	34.308000	14.208000	44.266000

H	40.969000	20.032000	38.782000	34.649000	15.988000	43.158000
H	39.722000	19.009000	37.994000	34.577000	16.467000	44.837000
H	44.249000	20.173000	31.094000	37.960000	17.567000	37.352000
H	44.866000	20.092000	29.592000	38.351000	17.994000	35.625000
H	43.886000	18.796000	29.953000	36.717000	17.551000	36.328000
H	41.305000	20.546000	27.950000	37.759000	21.660000	36.124000
H	41.979000	18.908000	28.146000	36.560000	20.420000	35.354000
H	42.866000	20.231000	27.412000	38.372000	20.239000	35.196000
H	42.981000	21.524000	29.804000	38.866000	19.788000	37.016000
H	41.116000	21.262000	30.150000	37.391000	20.974000	37.954000
H	40.925000	19.536000	30.527000	36.111000	20.013000	38.455000
H	34.389000	29.718000	25.996000	37.198000	27.786000	47.351000
H	36.390000	30.978000	25.222000	39.349000	29.169000	48.087000
H	38.160000	29.883000	25.208000	39.942000	31.160000	46.999000
H	38.941000	28.330000	27.053000	38.095000	32.627000	46.108000
H	37.094000	27.484000	28.407000	36.135000	31.240000	45.256000
H	32.858000	20.557000	24.217000	33.132000	27.181000	40.888000
H	33.832000	19.105000	22.454000	33.329000	27.449000	38.294000
H	36.508000	18.940000	22.112000	31.777000	29.338000	37.294000
H	37.581000	21.006000	22.772000	30.206000	30.626000	38.542000
H	36.638000	22.217000	25.112000	31.177000	30.714000	40.882000
H	34.976000	27.608000	22.800000	34.912000	33.046000	45.275000
H	35.132000	28.019000	20.349000	34.871000	35.476000	44.806000
H	34.921000	26.175000	18.808000	33.350000	36.524000	42.807000
H	33.892000	23.830000	19.679000	31.309000	35.125000	42.471000
H	33.398000	23.588000	22.073000	31.156000	32.839000	43.016000
H	29.018000	27.662000	25.162000	29.150000	32.551000	45.298000
H	27.054000	28.665000	23.605000	27.723000	34.391000	46.230000
H	26.383000	27.555000	21.701000	26.674000	34.092000	48.758000
H	27.736000	25.540000	20.962000	27.366000	32.161000	50.048000
H	29.445000	24.393000	22.423000	28.438000	30.321000	48.978000
H	28.344000	22.304000	25.794000	28.779000	26.611000	42.147000
H	26.693000	20.257000	25.582000	27.030000	25.042000	42.222000
H	27.237000	18.110000	26.842000	24.632000	25.678000	42.905000
H	29.253000	17.890000	28.060000	24.641000	27.414000	44.850000
H	31.100000	19.931000	28.320000	26.757000	28.442000	45.467000
H	29.016000	28.445000	26.387000	33.052000	30.243000	50.517000
H	27.621000	30.368000	27.190000	32.493000	30.387000	52.845000
H	27.217000	30.187000	29.740000	31.432000	28.053000	54.004000
H	29.483000	30.197000	30.984000	31.060000	26.402000	52.690000
H	31.038000	28.439000	30.303000	32.145000	25.999000	50.313000
H	30.507000	21.422000	30.482000	30.333000	23.450000	45.463000
H	30.193000	20.719000	32.801000	29.070000	21.585000	46.757000
H	31.988000	22.082000	33.928000	29.376000	21.382000	49.338000
H	31.620000	24.304000	34.370000	30.950000	23.060000	50.425000

H	31.795000	25.384000	32.329000	32.100000	24.886000	49.309000
H	34.088000	22.607000	33.880000	32.790000	21.537000	45.357000
H	35.580000	23.657000	33.819000	34.562000	21.615000	45.255000
H	37.586000	24.286000	32.545000	35.936000	23.512000	45.616000
H	36.239000	25.280000	31.872000	37.304000	24.169000	44.576000
H	37.767000	25.085000	30.884000	36.752000	22.507000	44.502000
H	37.683000	21.148000	30.890000	34.441000	24.694000	41.179000
H	38.714000	22.523000	31.366000	34.333000	22.869000	41.671000
H	38.328000	22.529000	29.708000	35.958000	23.808000	41.284000
H	34.364000	22.046000	31.822000	32.835000	23.559000	43.694000
H	34.037000	23.711000	31.829000	33.654000	24.011000	45.151000
H	35.783000	22.402000	36.416000	34.097000	18.817000	44.890000
H	36.736000	21.007000	36.524000	33.374000	18.042000	43.613000
H	34.900000	20.968000	36.356000	32.358000	19.038000	44.821000
H	36.226000	19.535000	32.421000	31.581000	21.262000	42.058000
H	34.733000	19.997000	32.840000	32.531000	19.950000	41.204000
H	35.478000	18.920000	33.970000	33.152000	21.349000	41.124000
H	59.377000	41.249000	50.880000	45.295000	59.030000	40.366000
H	58.800000	42.912000	50.600000	45.532000	57.476000	41.009000
H	58.602000	41.611000	49.362000	44.608000	57.598000	39.556000
H	61.821000	41.963000	48.001000	41.711000	59.359000	41.082000
H	60.323000	40.933000	48.153000	42.314000	58.557000	39.691000
H	61.706000	40.736000	49.351000	42.925000	60.193000	39.939000
H	60.982000	42.996000	50.083000	44.137000	59.261000	41.896000
H	60.763000	43.876000	47.771000	42.290000	58.335000	42.305000
H	59.321000	43.029000	47.386000	42.328000	56.832000	41.531000
H	63.820000	44.231000	52.303000	44.474000	62.987000	42.750000
H	62.754000	45.591000	52.999000	45.450000	62.801000	44.212000
H	64.020000	46.160000	51.953000	43.684000	62.902000	44.420000
H	64.058000	45.631000	49.728000	42.035000	61.499000	43.847000
H	62.679000	45.061000	48.601000	42.549000	59.899000	44.021000
H	63.514000	43.992000	49.824000	42.781000	60.613000	42.456000
H	61.693000	44.627000	51.222000	44.939000	60.500000	43.415000
H	61.751000	46.928000	51.841000	45.457000	61.093000	45.218000
H	62.571000	47.572000	50.388000	44.040000	60.910000	46.226000
H	54.164000	42.096000	51.297000	49.014000	53.868000	39.917000
H	55.094000	41.118000	52.358000	49.136000	55.491000	39.310000
H	55.779000	41.364000	50.801000	47.415000	54.618000	39.410000
H	55.789000	44.800000	53.467000	49.677000	56.023000	42.652000
H	55.679000	43.062000	53.819000	50.303000	56.056000	41.024000
H	54.348000	43.958000	53.164000	49.966000	54.553000	41.766000
H	56.977000	43.068000	52.236000	47.984000	56.319000	41.093000
H	56.029000	43.035000	50.038000	46.518000	54.744000	41.236000
H	54.524000	44.003000	50.590000	47.264000	53.485000	42.103000
H	53.545000	50.426000	47.919000	43.726000	51.966000	48.485000

H	52.084000	50.013000	48.807000	45.091000	50.807000	48.831000
H	51.867000	50.364000	47.030000	43.488000	50.246000	48.360000
H	52.928000	47.835000	45.088000	43.368000	49.847000	44.988000
H	54.035000	49.132000	45.472000	42.850000	51.347000	45.797000
H	52.440000	49.526000	45.390000	42.593000	49.810000	46.610000
H	51.640000	48.221000	46.817000	44.825000	49.516000	46.933000
H	52.810000	47.425000	48.787000	46.435000	51.365000	46.610000
H	53.204000	46.445000	47.366000	45.548000	51.084000	44.946000
H	59.395000	46.046000	44.564000	42.297000	53.052000	43.797000
H	59.712000	47.713000	44.027000	40.512000	53.045000	43.441000
H	59.210000	46.422000	42.820000	41.293000	51.589000	43.724000
H	57.801000	47.274000	41.528000	39.782000	51.122000	45.452000
H	57.638000	48.670000	42.660000	38.982000	52.676000	45.410000
H	56.201000	47.769000	42.048000	39.720000	52.084000	47.006000
H	57.396000	45.931000	43.406000	41.629000	51.990000	46.004000
H	57.052000	48.480000	44.911000	40.625000	54.529000	45.327000
H	55.856000	47.230000	44.800000	40.929000	54.335000	47.128000
H	61.739000	48.679000	47.160000	41.516000	55.325000	48.686000
H	62.591000	50.084000	47.714000	40.583000	56.640000	49.074000
H	62.983000	49.476000	45.996000	40.753000	55.327000	50.322000
H	59.601000	49.049000	45.256000	43.788000	54.895000	49.875000
H	61.101000	49.729000	44.451000	42.928000	54.650000	51.369000
H	59.546000	50.576000	44.603000	44.233000	55.824000	51.237000
H	61.610000	51.376000	46.159000	42.087000	57.071000	50.999000
H	60.619000	50.944000	48.473000	42.517000	58.033000	48.874000
H	59.233000	51.207000	47.371000	43.877000	57.817000	50.001000
H	56.837000	50.103000	54.324000	49.055000	60.477000	45.668000
H	55.454000	49.287000	53.304000	48.309000	59.908000	47.046000
H	55.074000	49.767000	54.887000	49.987000	60.444000	47.269000
H	55.062000	46.990000	55.400000	50.469000	58.928000	48.444000
H	56.548000	46.064000	55.170000	50.919000	57.360000	47.875000
H	55.623000	46.624000	53.869000	49.202000	57.680000	48.187000
H	57.183000	48.511000	55.687000	50.724000	59.315000	46.180000
H	58.503000	48.855000	53.908000	49.471000	58.543000	44.585000
H	58.733000	47.129000	53.990000	49.979000	56.977000	45.050000
H	50.519000	51.569000	45.102000	45.063000	49.793000	57.731000
H	50.762000	53.245000	42.938000	43.387000	51.554000	58.328000
H	52.298000	54.449000	43.643000	43.687000	53.881000	57.649000
H	53.175000	54.528000	45.934000	45.368000	54.504000	55.573000
H	51.665000	53.599000	47.787000	46.589000	52.486000	54.751000
H	48.670000	46.538000	52.491000	48.973000	49.793000	49.138000
H	49.891000	44.402000	52.561000	49.378000	49.572000	46.887000
H	51.203000	44.503000	50.638000	48.436000	47.747000	45.401000
H	52.790000	45.765000	50.079000	47.310000	45.676000	46.852000
H	52.146000	47.763000	50.126000	46.968000	45.848000	48.805000

H	49.163000	49.111000	44.464000	44.151000	47.769000	50.202000
H	50.042000	47.471000	42.841000	42.023000	49.051000	49.718000
H	50.176000	44.809000	43.477000	40.347000	49.298000	51.656000
H	49.756000	44.168000	45.713000	40.768000	48.475000	53.708000
H	49.153000	45.480000	47.549000	42.722000	47.214000	54.372000
H	44.416000	49.444000	46.078000	47.074000	42.248000	54.725000
H	42.223000	48.590000	45.102000	46.514000	40.557000	56.082000
H	41.115000	46.328000	46.595000	44.023000	40.398000	55.622000
H	41.717000	46.692000	49.033000	42.823000	42.350000	54.428000
H	43.736000	47.649000	49.837000	44.458000	44.658000	54.038000
H	47.281000	46.871000	53.246000	52.299000	45.326000	52.519000
H	45.595000	45.268000	54.855000	52.930000	42.760000	52.350000
H	43.384000	46.000000	55.097000	52.429000	41.416000	50.333000
H	42.477000	48.148000	54.457000	50.226000	41.898000	49.252000
H	43.997000	49.813000	53.139000	48.819000	43.648000	50.609000
H	43.754000	51.755000	46.969000	47.586000	49.037000	58.912000
H	41.709000	53.118000	45.763000	48.549000	49.393000	61.221000
H	41.653000	55.688000	45.988000	49.873000	47.382000	62.476000
H	43.443000	56.869000	47.489000	51.067000	46.099000	60.730000
H	45.484000	55.641000	48.407000	50.121000	45.698000	58.284000
H	45.591000	51.941000	53.857000	52.916000	46.766000	53.649000
H	45.110000	53.454000	55.876000	55.296000	47.079000	53.911000
H	45.750000	56.115000	55.594000	56.226000	48.636000	55.912000
H	47.534000	56.534000	54.108000	54.374000	49.945000	56.892000
H	48.252000	54.964000	52.368000	52.170000	49.277000	56.913000
H	55.101000	51.282000	53.151000	49.678000	53.967000	48.870000
H	54.874000	52.923000	52.527000	49.745000	55.010000	50.241000
H	49.114000	53.447000	53.044000	53.211000	52.237000	53.146000
H	50.036000	54.274000	54.180000	52.673000	50.743000	52.307000
H	50.083000	52.570000	54.346000	52.321000	51.075000	53.962000
H	50.988000	55.212000	51.612000	49.628000	54.109000	53.588000
H	52.632000	54.695000	50.955000	51.284000	54.808000	53.143000
H	52.504000	55.467000	52.588000	51.202000	53.550000	54.499000
H	52.583000	51.278000	53.440000	50.358000	52.067000	50.149000
H	53.265000	52.622000	54.243000	51.641000	53.385000	50.303000
H	52.566000	49.987000	51.556000	48.254000	51.814000	48.161000
H	54.080000	49.285000	52.074000	46.486000	52.366000	48.232000
H	53.431000	49.070000	50.278000	47.189000	51.156000	49.466000
H	53.901000	52.240000	48.628000	46.286000	54.277000	51.566000
H	54.778000	53.491000	49.613000	47.289000	52.914000	52.068000
H	53.203000	53.023000	50.001000	45.863000	52.654000	51.022000

Table S8. Molecule interatomic distances of each of Ph_7T_8-T_8iBu_7 structure from eight obtained clusters.

	Molecule interatomic distance along the axis [Å]											
	1st molecule			2nd molecule			3rd molecule			4th molecule		
	x	y	z	x	y	z	x	y	z	x	y	z
Cluster **1**	24.50	13.46	13.80	25.16	15.22	16.69	28.14	13.54	12.44	24.61	18.40	19.22
Cluster **2**	23.21	13.85	14.54	27.27	15.77	14.50	24.26	13.92	12.43	25.83	14.41	16.31
Cluster **3**	23.39	15.07	13.62	28.44	16.81	14.20	24.63	15.77	13.12	25.58	13.26	16.28
Cluster **4**	24.28	14.14	14.15	26.53	14.92	13.70	27.72	13.65	15.66	23.40	15.95	16.06
Cluster **5**	24.79	13.74	16.77	28.07	14.68	15.14	24.03	14.32	16.75	24.05	14.99	15.52
Cluster **6**	26.03	13.78	16.14	26.57	16.43	15.83	25.02	14.24	14.99	25.77	13.50	14.32
Cluster **7**	24.45	13.51	14.04	27.29	14.11	13.60	23.67	12.59	13.94	24.47	13.34	13.69
Cluster **8**	26.01	15.95	14.85	27.61	16.61	15.76	26.18	13.15	14.35	27.62	16.33	16.74
